# Intestinal Microbiota Is Influenced by Gender and Body Mass Index

**DOI:** 10.1371/journal.pone.0154090

**Published:** 2016-05-26

**Authors:** Carmen Haro, Oriol A. Rangel-Zúñiga, Juan F. Alcalá-Díaz, Francisco Gómez-Delgado, Pablo Pérez-Martínez, Javier Delgado-Lista, Gracia M. Quintana-Navarro, Blanca B. Landa, Juan A. Navas-Cortés, Manuel Tena-Sempere, José C. Clemente, José López-Miranda, Francisco Pérez-Jiménez, Antonio Camargo

**Affiliations:** 1 Lipids and Atherosclerosis Unit, GC9 Nutrigenomics. IMIBIC/Reina Sofia University Hospital/University of Cordoba, Cordoba, Spain; 2 CIBER Fisiopatología de la Obesidad y Nutrición (CIBEROBN), Instituto de Salud Carlos III, Cordoba, Spain; 3 Instituto de Agricultura Sostenible (IAS), Consejo Superior de Investigaciones Científicas (CSIC), Cordoba, Spain; 4 Department of Cell Biology, Physiology, and Immunology, IMIBIC/Reina Sofia University Hospital/University of Cordoba, Cordoba, Spain; 5 Department of Genetics and Genomic Sciences, Icahn School of Medicine at Mount Sinai, New York, NY 10029, United States of America; 6 Immunology Institute, Icahn School of Medicine at Mount Sinai, New York, NY 10029, United States of America; Institute of Agrochemistry and Food Technology, National Research Council (IATA-CSIC), SPAIN

## Abstract

Intestinal microbiota changes are associated with the development of obesity. However, studies in humans have generated conflicting results due to high inter-individual heterogeneity in terms of diet, age, and hormonal factors, and the largely unexplored influence of gender. In this work, we aimed to identify differential gut microbiota signatures associated with obesity, as a function of gender and changes in body mass index (BMI). Differences in the bacterial community structure were analyzed by 16S sequencing in 39 men and 36 post-menopausal women, who had similar dietary background, matched by age and stratified according to the BMI. We observed that the abundance of the *Bacteroides* genus was lower in men than in women (P<0.001, Q = 0.002) when BMI was > 33. In fact, the abundance of this genus decreased in men with an increase in BMI (P<0.001, Q<0.001). However, in women, it remained unchanged within the different ranges of BMI. We observed a higher presence of *Veillonella* (84.6% vs. 47.2%; X^2^ test P = 0.001, Q = 0.019) and *Methanobrevibacter* genera (84.6% vs. 47.2%; X^2^ test P = 0.002, Q = 0.026) in fecal samples in men compared to women. We also observed that the abundance of *Bilophila* was lower in men compared to women regardless of BMI (P = 0.002, Q = 0.041). Additionally, after correcting for age and sex, 66 bacterial taxa at the genus level were found to be associated with BMI and plasma lipids. Microbiota explained at *P* = 0.001, 31.17% variation in BMI, 29.04% in triglycerides, 33.70% in high-density lipoproteins, 46.86% in low-density lipoproteins, and 28.55% in total cholesterol. Our results suggest that gut microbiota may differ between men and women, and that these differences may be influenced by the grade of obesity. The divergence in gut microbiota observed between men and women might have a dominant role in the definition of gender differences in the prevalence of metabolic and intestinal inflammatory diseases.

## Introduction

Gut microbiota acts collectively as an organ fully integrated in host’s metabolism, and is involved in energy extraction from nutrients, regulating innate and adaptative immunity, and participating in the control of the energy balance [1]. However, several studies have proposed that changes in intestinal microbiota may trigger the pathogenic mechanisms which are involved in the development of obesity and insulin resistance [2–4], both associated with high cardiovascular risk [5]. Moreover, studies in animal models have shown that obesity is associated with an increase in the *Firmicutes/Bacteroidetes* ratio [6]. Studies with gnotobiotic mice colonized with the microbiota of lean or obese twins have also shown that this phenotype is also transmissible [7]. In contrast, studies in humans have yielded conflicting results, which may be explained by the inter-individual heterogeneity to which the gut microbiota is exposed. Fundamentally, this comes from different environmental factors such as diet, host metabolism, and hormonal factors [8]. In fact, gut microbiota composition seems to be more influenced by ambient and dietary cues than by genetic factors [9, 10].

In line with this, studies in humans have shown that gut microbiota seems to have coevolved with dietary habit [11, 12]. Recent research indicates that changes in gut microbiota composition may occur after dietary interventions [13–15]. In addition, it has been shown that microbial exposure and sex hormones exert potent effects on autoimmune diseases, which is more prevalent in women than in men [16]. Nevertheless, other studies have also linked gut microbiota to phospholipid metabolism and cardiovascular risk [17]. Overall, the incidence of metabolic diseases and their co-morbidities is sexually dimorphic and varies depending on gonadal status; e.g., increases after menopause [18]. Likewise, sex hormones are thought to play an important role in the development of cardiovascular diseases [19–21].

In addition, alteration of the intestinal microbiota has been demonstrated to be a key player in the protracted course of inflammation in inflammatory bowel diseases (IBD) [22]. This disease is more prevalent in females than in males and has the highest rates in developed Western parts of the world [23]. Therefore, the latter suggests that environmental exposures may be contributing to the pathogenesis of IBD [24].

The influence of gut microbiota in the incidence of metabolic and intestinal diseases is rather complex due to the inter-individual heterogeneity. Furthermore, the proportion of Firmicutes/Bacteroidetes in lean and obese humans has yielded contradictory results, as well as studies describing gender-related differences in the gut microbiome [25, 26]. In order to clarify this question, our objective was to identify the gut microbiota signatures associated with obesity as a function of changes in gender and BMI.

## Materials and Methods

### Study Subjects

This current study was conducted in a subgroup of 75 patients (39 men and 36 women) within the CORDIOPREV study (Clinical Trials.gov.Identifier: NCT00924937), an ongoing prospective, randomized, opened, and controlled trial in patients with coronary heart disease (CHD), who had their last coronary event over six months before enrolling in two different dietary models (Mediterranean and low-fat) over a period of five years, in addition to conventional treatment for CHD [27].

We analyzed the baseline fecal samples of 75 patients (39 men and 36 women), who were also divided into three groups, according to their BMI: 13 men and 13 women with BMI < 30; 13 men and 10 women with 30 ≤ BMI ≤ 33; and 13 men and 13 women with BMI > 33. The metabolic characteristics of the subjects in the study are shown in S1 Table.

### Ethics, consent and permissions

The trial protocol and all amendments were approved by the Reina University Hospital Ethics Committee, following the Helsinki declaration and good clinical practice. However, all patients gave written informed consent to participate in the study.

### Diet assessment

We performed a validated 14-item questionnaire to assess adherence to the Mediterranean Diet [28] and a similar 9-point score to assess adherence to low-fat diet at baseline before the start of the dietary intervention (and yearly follow-up visits, in the original study). Fiber intake was calculated using the Spanish food composition tables and through a validated food frequency questionnaire [29].

### Clinical plasma parameters

Blood was collected in tubes containing EDTA to give a final concentration of 0.1% EDTA. The plasma was separated from the red cells by centrifugation at 1500 X g for 15 min at 4°C. Analytes determined in frozen samples were analyzed centrally by the laboratory investigators of the Lipid and Atherosclerosis Unit at the Reina Sofia University Hospital, who were unaware of the interventions. Lipid variables were assessed with a DDPPII Hitachi modular analyzer (Roche) using specific reagents (Boehringer-Mannheim). Plasma triglycerides (TG) and cholesterol concentrations were assayed by enzymatic procedures [30, 31]. High-Density Lipoprotein—cholesterol (HDL-c) was measured by the precipitation of a plasma aliquot with dextran sulphate-Mg2+, as described by Warnick et al. [32]. Low-Density Lipoprotein—cholesterol (LDL-c) was calculated using the following formula: plasma cholesterol–(HDL-C + large Triglyceride-Rich Lipoproteins-Cholesterol (TRL-C) + small TRL-C). Also, glucose determination was performed using the hexokinase method.

### DNA extraction from fecal samples

To collect the fecal samples, we gave the patients a box with carbonic snow and a sterile plastic bottle with a screw cap to keep the frozen sample. Once it was delivered to the laboratory staff, the sample was stored at -80°C until microbial DNA was extracted. This was performed using the QIAamp DNA kit Stool Mini Kit Handbook (Qiagen, Hilden, Germany) following the manufacturer’s instructions. This protocol is optimized for a 180–220 mg sample. DNA was quantified using a Nanodrop ND-1000 v3.5.2 spectrophotometer (Nanodrop Technology^®^, Cambridge, UK) and the samples were stored at -20°C.

### Sequencing the V4 16S microbial rRNA on the Illumina MiSeq

Sample preparation was performed similarly to that described by Costello et al. [33]. Briefly, the 75 samples were amplified in triplicates by polymerase chain reaction (PCR) to generate an amplification library (modified from Sarah Owens, Argonne National Labs), with each sample being amplified in 3 replicate 25 μL PCR reactions. The PCR experimental condition for the 515–806 bp region of the 16S rRNA gene, and the sequencing procedures with the Illumina platform has been described by Caporaso et al. [34].

### Upstream informatics analysis of the 16S sequences

The obtained 16S rRNA sequences were analyzed using QIIME with default parameters unless indicated otherwise [34]. Briefly, raw sequencing data was de- multiplexed and low quality reads were discarded. Reads were clustered using a closed-reference OTU picking protocol that assigned reads to reference sequences from Greengenes v13-8 [35]. Taxonomy was assigned to the OTUs against the Greengenes v13-8 preclustered at 97% identity. Differences between bacterial communities were calculated in QIIME using rarefaction curves of alpha-diversity indexes including estimates of community richness (such as the Chao1 estimator, Good´s coverage, the observed number of OTUs present in each sample and Phylogenetic diversity (PD) or the length of the phylogenetic branch observed in each sample). Due to the unequal size of our library per sample and with the purpose to retain all samples each library was sub-sampled to an even sequencing depth of exactly 2000 sequences per sample (the lowest number of reads obtained for any of the 75 samples analyzed) to mitigate biases arising from different depths of sequence across samples. Beta diversity was estimated using weighted and unweighted UniFrac distance [36]. Beta-diversity distance matrices were built after sub-sampling all the samples to an even depth of 2000 sequences per sample, which is the same with the depth provided to script alpha_rarefaction.py. Relative taxonomic abundance was measured as the proportion of reads over the total in each sample assigned to a given taxonomy.

### Statistical analysis

All the data presented in this study are expressed as mean±SEM. PASW statistical software, version 20.0 (IBM Inc., Chicago, IL, USA) and R software, version 3.0.2 (R Foundation for Statistical Computing, http://www.R-project.org/) were used for statistical analyses of data. The normal distribution of variables was assessed using the Kolmogorov-Smirnov test. The statistical differences in the main metabolic variables between groups were evaluated using One-way ANOVA. In order to assess whether specific differences occurred in some bacterial taxa between genders and bacterial species, we compared the abundance of taxa present at least in the 75% of the human fecal DNA samples from men and at least in 75% of the total human fecal DNA samples from women. The specific differences in bacterial taxa between genders and bacterial species were evaluated by the Mann-Whitney *U* test. The specific differences in bacterial taxa by BMI were evaluated by the Kruskal-Wallis test. EPIDAT program v4.1 was used to evaluate power and sample size calculations (Epidat: programa para análisis epidemiológico de datos. Versión 4.1, octubre 2014. Consellería de Sanidad, Xunta de Galicia, España; Organización Panamericana de la salud (OPS-OMS); Universidad CES, Colombia.). Furthermore, we also analyzed the frequency of occurrence of taxa identified at least in 25% of the total human fecal DNA samples from men and at least in 25% of the total human fecal DNA samples from women. The X^2^ test was applied to establish differences in bacterial prevalence between the studied groups. Results were adjusted by False Discovery Rate (FDR) using Benjamini and Hochberg method. FDR adjusted p-value (or q-value) of 0.05 was considered statistically significant.

A two part model for association analysis between BMI and lipids with either OTUs or taxonomic (at the genus level) units adjusting for age and gender was performed as described by Fu et al. [37]. This approach overcomes the problem of an ab non-normal distribution, which is a feature of the majority of gut bacteria OTUs or taxa. Briefly, the first part describes a binomial analysis that tests for the association of detecting a microbe (represented by an OTU or taxonomy) with a trait. The second part of the quantitative analysis tests for association between the lipid level and the abundance of bacteria, but only for the subjects where that microbe is present. To further combine the effect of both binary and quantitative analysis, a meta *P* value was derived using an unweighted *Z* method. Then, a final association *P* value per microbe-trait pair was assigned from the minimum of *P* values from binary analysis, quantitative analysis, and meta-analysis [37].

Finally, the proportion of variation in BMI and lipids could be explained by the gut microbiome, based on significantly associated OTUs identified in the two-part model at a certain *P* value (ranging from 0.001 to 0.1). Also, the risk (*rm*) of the gut microbiome on BMI or lipids for each individual using an additive model was estimated according to Fu et al. [37]. The variation in BMI and lipids explained by the gut microbiome was represented as the squared correlation coefficient between the traits and *rm*, after correcting for age and gender.

## Results

### Baseline characteristic of the study participants

No statistical significant differences in age, BMI, glucose, TG, LDL-c, total cholesterol and systolic blood pressure were observed between men and women. However, women had higher HDL-c levels than men (*P = 0*.*022*) and men higher diastolic blood pressure (*P = 0*.*035*) (S1 Table). In addition, we did not find any differences in the diet (S2 Table) and in macronutrients intake (S3 Table) between men and women.

### Gender and gut microbiota

For the bacterial community analyses of the 75 samples, after screening our data for poor quality sequences, we recovered 1,296,641 high-quality 16S rRNA gene sequences with 706,186 and 590,455 sequences for men and women, respectively, with an average of 17,469 sequences per sample (Min-2015; Max-33,319). There were no significant differences in bacterial diversity between males and females with any of the alpha diversity estimators used and at a rarefaction level of 2,000 sequences per sample. With this depth we reached satisfactory coverage of the diversity for all samples since all Good’s coverage values ranged between 98.43 and 99.77 (data not shown). Similarly, Principal Coordinate Analysis (PCoA) or UPGMA clustering based on unweighted and weighted UniFrac distances did not show significant differences in microbiota composition between men and women (S1 and S2 Figs).

Although diversity and overall community composition were not significantly different between males and females, we investigated whether the relative abundance of specific taxa might differ among groups. Regardless of BMI, we did not find any differences at phylum level between men and women. However, at the genera level, we observed that the abundance of the *Bilophila* genus was higher in women than in men (*P = 0*.*002*, *Q = 0*.*041*). Higher presence of *Veillonella* (33/39, 84.6% vs. 17/36, 47.2%; X^2^ test *P = 0*.*001*, *Q = 0*.*019*) and *Methanobrevibacter* genera (33/39, 84.6% vs. 17/36, 47.2%; X^2^ test *P = 0*.*002*, *Q = 0*.*026*) was observed in fecal samples from men compared to women.

In addition, at the bacterial species level, we observed that the abundance of *Bacteroides caccae* was higher in women than in men (*P = 0*.*009*, *Q = 0*.*035*). On the other hand, the abundance of *Bacteroides plebeius* was higher in men than in women (*P = 0*.*001*, *Q = 0*.*006*). Moreover, we observed a higher presence of *Coprococcus catus* (30/39, 76.9% vs. 13/36, 36.1%; X^2^ test *P<0*.*001*, *Q = 0*.*011*) in fecal samples from men compared to women.

### Gender differences in the gut microbiota are influenced by BMI

**Fig 1:**
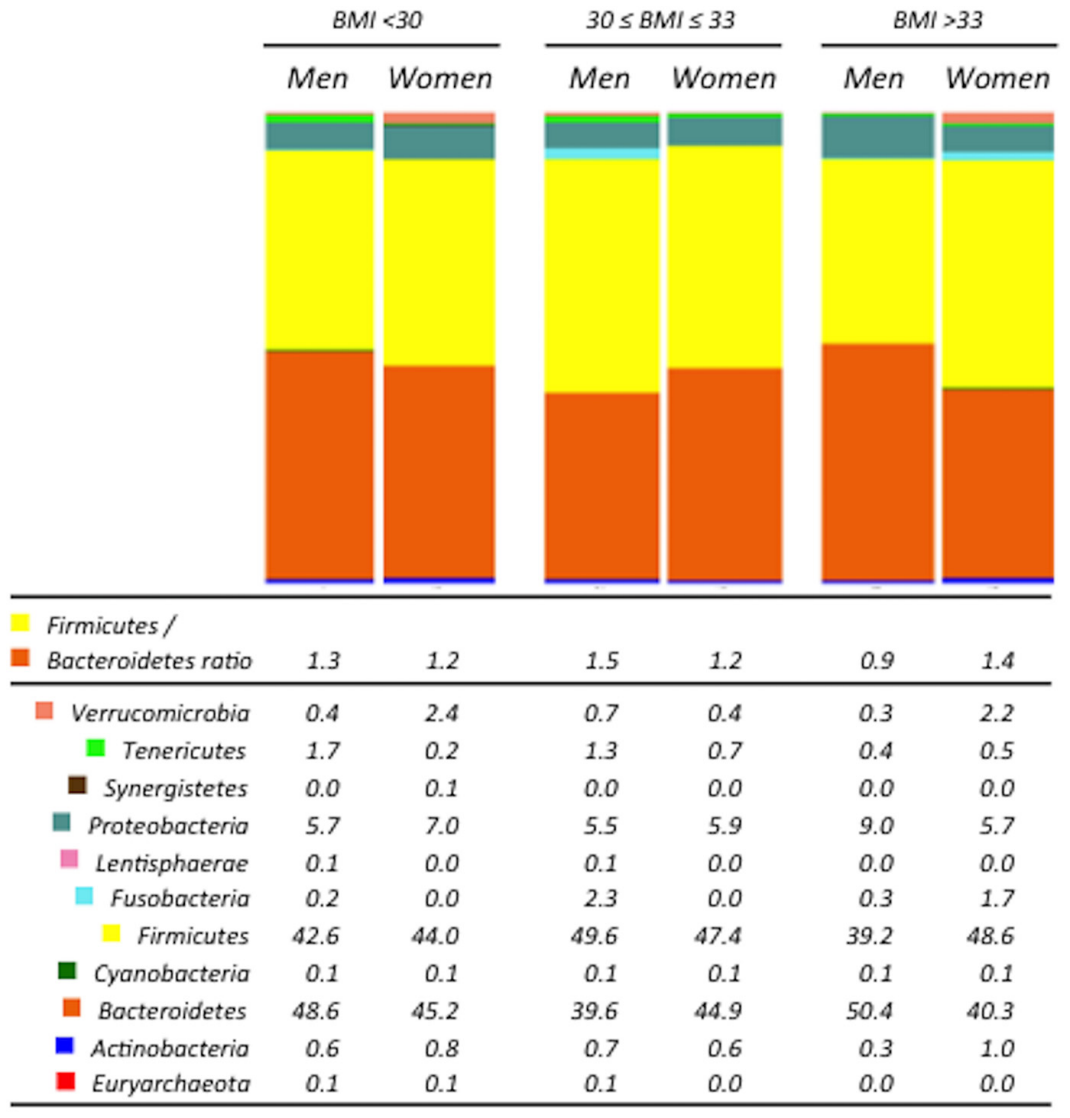
Gender differences in the gut microbiota at the phylum level. The abundance of the bacterial phyla was obtained by analyzing the 16S rRNA sequences using QIIME. *Firmicutes*/*Bacteroidetes* ratio was calculated dividing the abundance of *Firmicutes* and *Bacteroidetes* for each subject. **BMI**: body mass index.

In order to assess whether specific differences in bacterial taxa between genders were influenced by BMI, we stratified both men and women into three groups each: BMI < 30; 30 ≤ BMI ≤ 33; and BMI > 33. Similar to the results on the non-stratified data, both alpha and beta diversity were not significantly different between males and females in any of the BMI groups (S1 and S2 Figs). No differences were observed at phyla level and *Firmicutes/Bacteriodetes* ratio between men and women when considered independently of the BMI. However, when we stratified men and women according to their BMI, we observed that men had higher *Firmicutes/Bacteriodetes* ratio under a BMI of 33. By contrast, men had a significantly lower *Firmicutes/Bacteriodetes* ratio than women in the BMI > 33 group (*P = 0*.*018*) (Fig 1).

**Fig 2:**
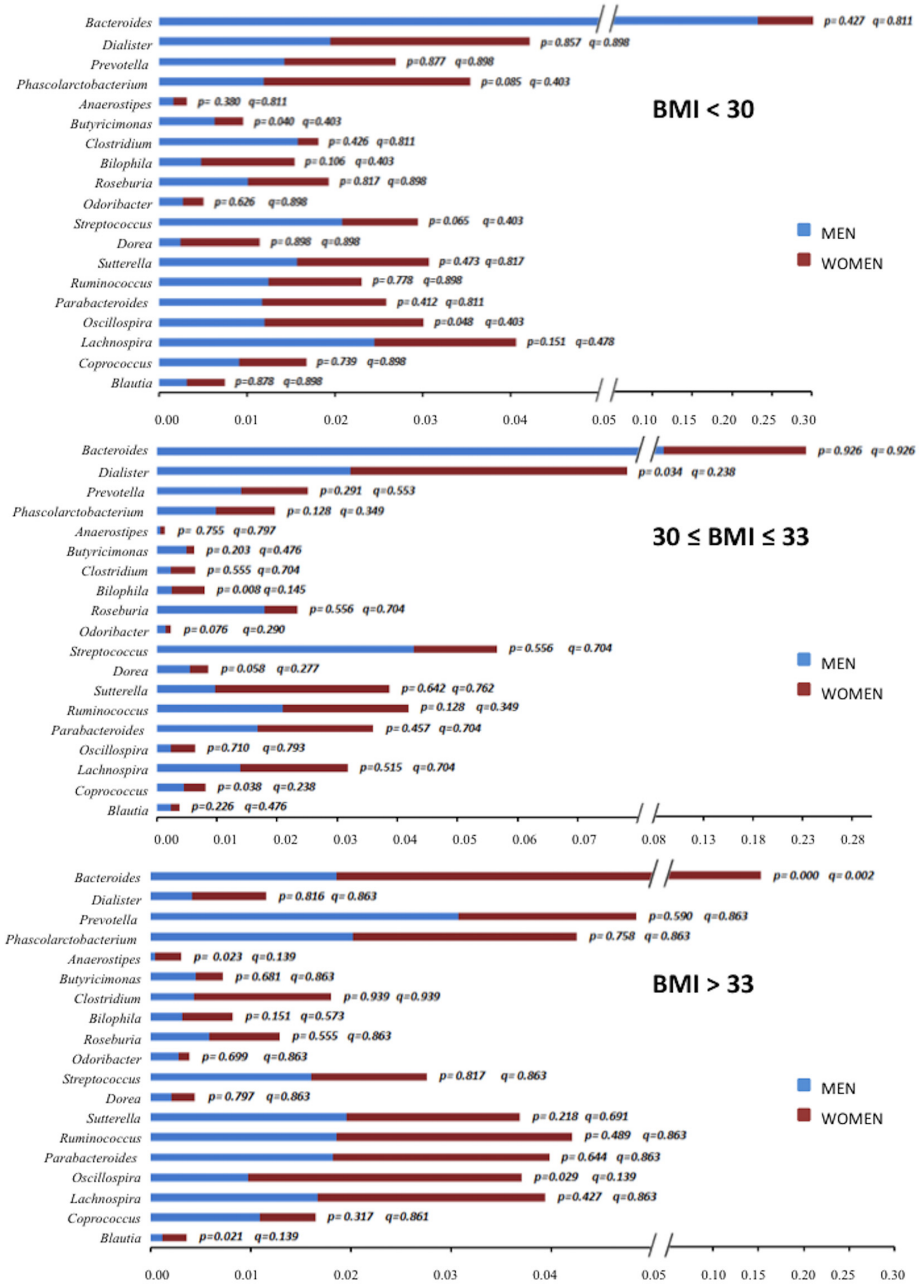
Gender differences in the gut microbiota at different BMI ranges at the genus level. The abundance of the bacterial phyla was obtained by analyzing the 16S rRNA sequences using QIIME. Bars show the comparison of the abundance of the different bacterial species between men and women at different BMI ranges by the Mann-Whitney *U* test (*P-value*). *Q-value*: False Discovery Rate (FDR) using Benjamini and Hochberg method. **BMI:** body mass index.

At genera level, we observed a significantly higher abundance of the *Bacteroides* genus in women than in men (*P<0*.*001*, *Q = 0*.*002*) with a BMI > 33, whereas we did not find any difference between genders in the abundance of this genus when the BMI was < 33 (Fig 2). This was consistent with the decrease in the abundance of *Bacteroides* genus in men with the increase of the BMI (*P<0*.*001*, *Q<0*.*001*), whereas in women, it remained unchanged in the different ranges of BMI (Table 1).

**Table 1:** Gender differences in the gut microbiota by BMI at genus level.

	*Men*					*Women*				
	*BMI < 30*	*30 ≤ BMI ≤ 33*	*BMI > 33*	*P-value*	*Q-value*	*BMI < 30*	*30 ≤ BMI ≤ 33*	*BMI > 33*	*P-value*	*Q-value*
***Bacteroides***	*0*.*232 ± 0*.*040*	*0*.*151 ± 0*.*025*	*0*.*019 ± 0*.*005*	*<0*.*001*	*<0*.*001*	*0*.*179 ± 0*.*039*	*0*.*165 ± 0*.*045*	*0*.*134 ± 0*.*022*	*0*.*919*	*0*.*952*
***Dialister***	*0*.*019 ± 0*.*004*	*0*.*018 ± 0*.*009*	*0*.*004 ± 0*.*002*	*0*.*008*	*0*.*053*	*0*.*023 ± 0*.*006*	*0*.*055 ± 0*.*015*	*0*.*007 ± 0*.*003*	*0*.*018*	*0*.*333*
***Prevotella***	*0*.*014 ± 0*.*008*	*0*.*014 ± 0*.*004*	*0*.*031 ± 0*.*012*	*0*.*712*	*0*.*798*	*0*.*013 ± 0*.*005*	*0*.*010 ± 0*.*004*	*0*.*018 ± 0*.*007*	*0*.*893*	*0*.*952*
***Phascolarctobacterium***	*0*.*012 ± 0*.*004*	*0*.*017 ± 0*.*003*	*0*.*020 ± 0*.*005*	*0*.*229*	*0*.*435*	*0*.*024 ± 0*.*005*	*0*.*011 ± 0*.*003*	*0*.*022 ± 0*.*006*	*0*.*181*	*0*.*619*
***Anaerostipes***	*0*.*002 ± 0*.*001*	*0*.*001 ± 0*.*000*	*0*.*000 ± 0*.*000*	*0*.*047*	*0*.*226*	*0*.*002 ± 0*.*001*	*0*.*001 ± 0*.*000*	*0*.*003 ± 0*.*001*	*0*.*854*	*0*.*952*
***Butyricimonas***	*0*.*006 ± 0*.*001*	*0*.*004 ± 0*.*001*	*0*.*005 ± 0*.*001*	*0*.*171*	*0*.*406*	*0*.*003 ± 0*.*001*	*0*.*002 ± 0*.*000*	*0*.*003 ± 0*.*001*	*0*.*228*	*0*.*619*
***Clostridium***	*0*.*016 ± 0*.*011*	*0*.*004 ± 0*.*001*	*0*.*004 ± 0*.*001*	*0*.*960*	*0*.*960*	*0*.*002 ± 0*.*000*	*0*.*004 ± 0*.*001*	*0*.*014 ± 0*.*008*	*0*.*732*	*0*.*952*
***Bilophila***	*0*.*005 ± 0*.*001*	*0*.*003 ± 0*.*001*	*0*.*003 ± 0*.*001*	*0*.*756*	*0*.*798*	*0*.*011 ± 0*.*003*	*0*.*007 ± 0*.*002*	*0*.*005 ± 0*.*001*	*0*.*586*	*0*.*952*
***Roseburia***	*0*.*010 ± 0*.*002*	*0*.*015 ± 0*.*005*	*0*.*006 ± 0*.*001*	*0*.*259*	*0*.*448*	*0*.*009 ± 0*.*002*	*0*.*007 ± 0*.*002*	*0*.*007 ± 0*.*002*	*0*.*797*	*0*.*952*
***Odoribacter***	*0*.*003 ± 0*.*001*	*0*.*003 ± 0*.*000*	*0*.*003 ± 0*.*001*	*0*.*145*	*0*.*393*	*0*.*002 ± 0*.*001*	*0*.*002 ± 0*.*000*	*0*.*001 ± 0*.*000*	*0*.*115*	*0*.*619*
***Streptococcus***	*0*.*021 ± 0*.*005*	*0*.*018 ± 0*.*012*	*0*.*016 ± 0*.*006*	*0*.*117*	*0*.*369*	*0*.*009 ± 0*.*003*	*0*.*012 ± 0*.*004*	*0*.*012 ± 0*.*002*	*0*.*624*	*0*.*952*
***Dorea***	*0*.*002 ± 0*.*000*	*0*.*005 ± 0*.*002*	*0*.*002 ± 0*.*001*	*0*.*202*	*0*.*426*	*0*.*009 ± 0*.*004*	*0*.*002 ± 0*.*001*	*0*.*002 ± 0*.*001*	*0*.*228*	*0*.*619*
***Sutterella***	*0*.*016 ± 0*.*003*	*0*.*016 ± 0*.*003*	*0*.*020 ± 0*.*003*	*0*.*601*	*0*.*798*	*0*.*015 ± 0*.*005*	*0*.*026 ± 0*.*009*	*0*.*017 ± 0*.*004*	*0*.*744*	*0*.*952*
***Ruminococcus***	*0*.*012 ± 0*.*003*	*0*.*021 ± 0*.*006*	*0*.*019 ± 0*.*005*	*0*.*589*	*0*.*798*	*0*.*011 ± 0*.*002*	*0*.*014 ± 0*.*007*	*0*.*024 ± 0*.*006*	*0*.*186*	*0*.*619*
***Parabacteroides***	*0*.*012 ± 0*.*002*	*0*.*017 ± 0*.*005*	*0*.*018 ± 0*.*006*	*0*.*679*	*0*.*798*	*0*.*014 ± 0*.*003*	*0*.*023 ± 0*.*006*	*0*.*022 ± 0*.*006*	*0*.*641*	*0*.*952*
***Oscillospira***	*0*.*012 ± 0*.*003*	*0*.*010 ± 0*.*001*	*0*.*010 ± 0*.*002*	*0*.*732*	*0*.*798*	*0*.*018 ± 0*.*003*	*0*.*010 ± 0*.*001*	*0*.*027 ± 0*.*009*	*0*.*042*	*0*.*403*
***Lachnospira***	*0*.*025 ± 0*.*007*	*0*.*011 ± 0*.*004*	*0*.*017 ± 0*.*006*	*0*.*076*	*0*.*287*	*0*.*016 ± 0*.*006*	*0*.*017 ± 0*.*006*	*0*.*023 ± 0*.*006*	*0*.*541*	*0*.*952*
***Coprococcus***	*0*.*009 ± 0*.*003*	*0*.*009 ± 0*.*001*	*0*.*011 ± 0*.*003*	*0*.*494*	*0*.*781*	*0*.*008 ± 0*.*002*	*0*.*005 ± 0*.*001*	*0*.*006 ± 0*.*001*	*0*.*952*	*0*.*952*
***Blautia***	*0*.*003 ± 0*.*001*	*0*.*003 ± 0*.*001*	*0*.*001 ± 0*.*000*	*0*.*007*	*0*.*053*	*0*.*004 ± 0*.*002*	*0*.*002 ± 0*.*000*	*0*.*002 ± 0*.*000*	*0*.*726*	*0*.*952*

**BMI:** body mass index. **Rows**: comparison of the abundance of the different bacterial genera between different BMI ranges in men and women together and in men and women separately by the Kruskal-Wallis test. **Q-value**: False Discovery Rate (FDR) using Benjamini and Hochberg method.

In addition, we observed that the abundance of *B*. *plebeius* was higher in BMI > 33 group in men than in women (*P = 0*.*005*, *Q = 0*.*041*) (Table 2). Thus, this was in line with the trend to increase in the abundance of this bacterial species observed with the BMI in men (*P = 0*.*021*, *Q = 0*.*055*).

**Table 2:** Gender differences in the gut microbiota at bacterial species level.

*Bacterial species*	*Men (m)*	*Women (w)*	*P-value (m/w)*	*Q-value (m/w)*
***B*. *uniformis***				
*All range BMI*	0.0228±0.0036	0.0253±0.0040	0.722	0.747
*BMI < 30*	0.0282±0.0061	0.0285±0.0084	0.719	0.956
*30 ≤ BMI ≤ 33*	0.0252±0.0079	0.0278±0.0074	0.495	0.660
*BMI > 33*	0.0151±0.0035	0.0201±0.0046	0.608	0.857
*P-value (BMI)*	0.201	0.626		
*Q-value (BMI)*	0.268	0.715		
***F*. *prausnitzii***				
*All range BMI*	0.0209±0.0032	0.0236±0.0057	0.270	0.539
*BMI < 30*	0.0242±0.0056	0.0274±0.0105	0.504	0.806
*30 ≤ BMI ≤ 33*	0.0223±0.0072	0.0107±0.0025	0.120	0.241
*BMI > 33*	0.0162±0.0036	0.0297±0.0114	0.857	0.857
*P-value (BMI)*	0.526	0.552		
*Q-value (BMI)*	0.602	0.715		
***B*. *ovatus***				
*All range BMI*	0.0048±0.0009	0.0049±0.0012	0.440	0.586
*BMI < 30*	0.0058±0.0020	0.0079±0.0028	0.836	0.956
*30 ≤ BMI ≤ 33*	0.0065±0.0013	0.0040±0.0015	0.097	0.241
*BMI > 33*	0.0022±0.0005	0.0025±0.0005	0.750	0.857
*P-value (BMI)*	0.016	0.369		
*Q-value (BMI)*	0.055	0.590		
***P*. *distasonis***				
*All range BMI*	0.0080±0.0012	0.0115±0.0023	0.438	0.586
*BMI < 30*	0.0060±0.0012	0.0120±0.0033	0.268	0.630
*30 ≤ BMI ≤ 33*	0.0103±0.0020	0.0103±0.0039	0.382	0.611
*BMI > 33*	0.0076±0.0026	0.0120±0.0048	0.291	0.775
*P-value (BMI)*	0.155	0.783		
*Q-value (BMI)*	0.248	0.783		
***P*. *copri***				
*All range BMI*	0.0916±0.0210	0.0732±0.0137	0.264	0.539
*BMI < 30*	0.0336±0.0256	0.0583±0.0211	0.013	0.106
*30 ≤ BMI ≤ 33*	0.0809±0.0275	0.0792±0.0230	0.756	0.864
*BMI > 33*	0.1603±0.0458	0.0836±0.0271	0.426	0.851
*P-value (BMI)*	0.021	0.310		
*Q-value (BMI)*	0.055	0.590		
***B*. *caccae***				
*All range BMI*	0.0061±0.0011	0.0100±0.0016	0.009	0.035
*BMI < 30*	0.0081±0.0024	0.0123±0.0032	0.315	0.630
*30 ≤ BMI ≤ 33*	0.0072±0.002	0.0118±0.0029	0.066	0.241
*BMI >33*	0.0029±0.0006	0.0062±0.0017	0.063	0.252
*P-value (BMI)*	0.099	0.069		
*Q-value (BMI)*	0.199	0.275		
***H*. *parainfluenzae***				
*All range BMI*	0.0052±0.0013	0.0041±0.0009	0.747	0.747
*BMI < 30*	0.0036±0.0015	0.0034±0.0015	0.958	0.958
*30 ≤ BMI ≤ 33*	0.0067±0.0032	0.0036±0.0006	0.949	0.949
*BMI > 33*	0.0052±0.0019	0.0052±0.0020	0.733	0.857
*P-value (BMI)*	0.885	0.367		
*Q-value (BMI)*	0.885	0.590		
***B*. *plebeius***				
*All range BMI*	0.0310±0.0041	0.0129±0.0027	0.001	0.006
*BMI < 30*	0.0221±0.0079	0.0085±0.0017	0.230	0.630
*30 ≤ BMI ≤ 33*	0.0242±0.0055	0.0081±0.0042	0.054	0.241
*BMI > 33*	0.0467±0.0063	0.0208±0.0062	0.005	0.041
*P-value (BMI)*	0.015	0.067		
*Q-value (BMI)*	0.055	0.275		

**BMI:** body mass index. **Rows**: comparison of the abundance of the different bacterial species between men and women with different BMI range or all BMI ranges together by the Mann-Whitney *U* test. **Columns**: comparison of the abundance of the different bacterial species between different BMI ranges in men and women separately by the Kruskal-Wallis test. **Q-value**: False Discovery Rate (FDR) using Benjamini and Hochberg method.

We also observed a higher presence of *C*. *catus* (10/13, 76.9% vs. 1/13, 7.7%; X^2^ test *P<0*.*001*, *Q = 0*.*011*), in fecal samples from men compared to women when the BMI was < 30. In fact, the prevalence of *C*. *catus* tended to increase with the BMI in women (1/13, 7.7% BMI<30; 5/10, 50.0% 30<BMI>33; 7/13, 53.9% BMI>33; X^2^ test *P = 0*.*028*, *Q = 0*.*873*), whereas it remained unchanged in men at the different BMI ranges.

Moreover, we observed a higher presence of *Bifidobacterium adolescentis* (10/13, 76.9% vs. 3/13, 23.1%; X^2^ test *P = 0*.*006*, *Q = 0*.*047*), *Eubacterium biforme* (10/13, 76.9% vs. 3/13, 23.1%; X^2^ test *P = 0*.*006*, *Q = 0*.*047*), and *Oxalobacter formigenes* (11/13, 84.6% vs. 3/13, 23.1%; X^2^ test *P = 0*.*002*, *Q = 0*.*026*) in fecal samples from men compared to women when the BMI was < 30. However, the prevalence of these bacterial species was not statistically different between men and women when the BMI was > 30.

### Relationship between gut microbiota and plasma lipid levels

In addition, we study the relationship between gut microbiota, BMI, and plasma lipid levels. After adjusting for age and sex, a total of 1428 associated OTUs were detected at FDR = 0.05. Out of them, 299 OTUs were associated with BMI, 312 with TG, 335 with HDL, 276 with LDL-c, and 223 with total cholesterol (S4a to S4e Table; S3A Fig). None of the OTUs was shared by all five traits, 1 to 3 OTUS were shared by four traits (7 OTUs in total), 1 to 9 OTUs were shared by three traits (41 OTUs in total), and 13 to 62 were shared by two traits (289 OTUs in total) (S3A Fig). Across traits, 726 associations (50.24%) were detected by binary analysis (presence/absence); 521 associations (36%) were detected by the quantitative model, and 723 associations (50.03%) were detected by the meta-analysis of binary and quantitative analyses (S4a to S4e Table).

At the genus taxonomy level, we identified 90 significant associations for 66 unique taxonomies at FDR = 0.05: 23 were associated with BMI, 18 with triglycerides, 17 with HDL, 14 with LDL, and 18 with cholesterol (S5a to S5e Table; S3B Fig). Also, none of the OTUs was shared by five or four traits, only 3 OTUS were shared by three traits, and 17 (1 to 4) OTUs were shared by two traits (S3B Fig).

Furthermore, we then estimated the proportion of variation in the metabolic traits that was explained by the microbiome. The OTUs identified at *P* = 0.001 level explained 31.17% variation in BMI, 29.04% in TG, 33.70% in HDL, 46.86% in LDL-c, and 28.55% in total cholesterol. As the significance level increases, the risk model included a higher number of OTUs increasing the proportion of the explained variance. Thus, the OTUs identified at *P* = 0.05 explained 64.06% variation in TG, 55.97% in HDL, 68.98% in LDL-c, and 58.97% in total cholesterol. However, this is with the exception of BMI for which the proportion of the explained variation decreased with the increase in the significance level, being estimated in 19.40% at *P* = 0.05 (S4 Fig).

## Discussion

Our study shows that the gut microbiota differs in men and women at the bacterial phyla level (*Firmicutes/Bacteriodetes* ratio), at the genus level (*Bacteroides*, *Bilophila*, *Veillonella*, *and Methanobrevibacter*), and at the species level (*B*. *plebeius*, *B*. *caccae*, *C*. *catus*). In fact, our results suggest that in this cohort of age- and diet-matched obese subjects, microbiota composition can be affected by gender in a BMI-specific manner.

Our study reveals that the *Firmicutes*/*Bacteroidetes* ratio, which has a great importance in the development of obesity [2], changed with the BMI and between genders. Notably, previous estimations of the proportion of *Firmicutes*/*Bacteroidetes* in lean and obese humans have yielded contradictory results. As reported in mice, several studies in humans found that this ratio is increased in obesity [38, 39]. However, others did not confirm these observations [40], or even show that the relative abundance of *Firmicutes* was reduced in obese subjects [41]. Furthermore, some studies reported differences in gut microbiota composition by age group [42, 43], and that gut microbiota may also differ between sexes in animal models [16]. Moreover, previous study testing for a relatively reduced number of taxa by fluorescent in situ hybridization showed that the abundance for *Bacteriodes-Prevotella* group (together *Bacteroidetes phylum)* was higher in men [25]. However, a recent study performed by NGS (Next Generation Sequencing) Roche 454 platform testing for the global pattern of the bacterial community showed that women was characterized by a lower abundance of *Bacteroidetes* [26]. The latter study also analyzed the gut microbiome according to BMI in women and men separately, but failed to find differences as the cohort studied had a 25.0±4.06 Kg/m^2^ (mean±SD) as the mean of BMIand therefore including include lean and mostly overweight people, but not obese people. This was such that a presumably too short range of BMI was analyzed. Our approach by NGS Illumina platform also test for the global pattern of the bacterial community which confirmed the lower abundance of *Bacteroidetes* in women as compared with men when BMI is around 25 Kg/m^2^ as was previously shown [26]. Consequently, this also showed differences at bacterial phyla, genus, and species levels between men and women. Thus, these differences were influenced by the grade of obesity as the BMI cohort which was included in the current work ranged from 23.44 to 41.88. Therefore, the conflicting results in term of intestinal bacterial proportions might be explained by the men/women ratio, range of age, and the grade of obesity in the different cohorts studied. In addition, in other human-associated microbial habitats such as the skin surface, various differences between genders have also been observed [44]. Additionally, diet modulates significantly the composition of the microbiota. Although the microbiota is generally highly stable over an extended period of time in the absence of significant perturbations [45], dietary interventions can induce quick changes in composition [13, 15].

In this study, we comparatively analyzed the microbiota of lean and obese men and women under a similar nutritional background, matched by age (with a mean age of 60 years) and stratified according to BMI. A higher proportion of *Firmicutes* was found in women regardless of the BMI. Interestingly, a higher proportion of *Firmicutes* was found in men under a BMI of 33, whereas a lower proportion was detected when BMI was > 33. Thus, this reflects a potential sexual dimorphism in gut microbiota composition that is variably influenced by BMI. In addition, we observed that the abundance of the *Bacteroides* genus was lower in men than in women when BMI was > 33. This is presumably a consequence of the decrease in the abundance of this genus in men with the increase of the BMI. Nevertheless, in women, it remained unchanged in the different ranges of BMI. Gender differences in fat distribution have been previously reported, and these are related to the differences in sex hormone levels [46]. Yet, little is still known about the cellular and molecular mechanisms underlying this phenomenon. The differences observed herein regarding microbiota architecture may stem from the actual differences in sex hormone levels in elder men and women. On the other hand, it might reflect the residual influence of the dramatic differences in sex steroid profiles early in life between sexes, which may have a persistent effect on gut microbiota over time.

The composition of the gut microbiota may determine how excess energy is stored in the body, and this effect might be sex dependent. Animal experiments have provided solid proof for this phenomenon, as the sexual dimorphism in total body fat content seen in rodents (males exhibiting higher fat content than females) has shown a fade away in germ free animals, thus suggesting a role for the gut microbiota [47]. Thus, it is plausible that the gender-related differences found in our study regarding bacterial composition may have an impact on how men and women differentially store excess energy.

In addition, increasing evidence suggests that the intestinal microbiota may play a role in the development of IBD [48, 49]. Moreover, the prevalence of IBD has been shown to be higher in females and with increasing age, and most common in Caucasians as compared with other ethnicities [50, 51]. In this context, it is tempting to hypothesize that the differences in gut microbiota composition reported here might contribute in determining gender differences in the prevalence of IBD. In fact, our study showed that *Bilophila* and *Blautia*, two IBD-related genera, were more abundant in women than in men. *Bilophila wadsworthia*, a sulphite-reducing bacteria, has been associated with an increased incidence of colitis [52], and emerges under pathological conditions such as appendicitis and other intestinal inflammatory disorders [52]. Moreover, the genus *Blautia*, recently reorganized to refer to several misclassified species belonging to the *Clostridium* cluster XIVa, which according to our study is more abundant in women than in men under obesity condition, display a high incidence in patients with IBD [53, 54]. Of note, the difference in the abundance of *Bilophila* and *Blautia* between men and women was influenced by the BMI. Thus, while the differences in *Bilophila* were more evident below 33 of BMI, we observed a significant trend of the differences in *Blautia* only above 33 of BMI. These findings support the possibility that obesity, by influencing microbiota composition, might influence the development of IBD. Although admittedly, the pathogenic role of overweight in IBD remain poorly understood.

In a recent study by Fu et al. [37], it has been shown that gut microbiota composition has little effect on LDL or TC levels. However, it makes a significant contribution to the individual variance seen in BMI and to the blood levels of triglycerides and HDL. In our study, we also found that gut microbiota has a significant contribution to the individual variance seen in BMI, triglycerides, and HDL. Thus, as a difference, we found that it also contributed to the variance seen in LDL and cholesterol.

Our study shows gender-related differences analyzing the full microbiome by using a NGS method. Our experimental design included a broad BMI range, including lean, overweight, obese, and morbidly obese people which allowed us to find out gut microbiota signatures associated with obesity as a function of changes in gender and the BMI. Consequently, this could open a new hypothesis to be tested in bigger populations as one limitation of this study is the reduced sample size, although large enough to detect relative gender-related changes in gut microbiota. However, small differences in taxa with high inter-individual variability may not have been detected, or we may not have had a sufficient sample size to detect small differences in taxa between groups. Further investigations in larger populations are needed to confirm these results and extend the knowledge about the gender differences in the gut microbiota.

In conclusion, our results suggest that gut microbiota may differ between men and women, and that these differences may be influenced by the grade of obesity. Thus, these results might be relevant for the proper understanding of the basis of gender differences in the prevalence of metabolic and intestinal inflammatory diseases. Further studies will however be needed to unveil the specific mechanisms, such as sex steroid milieu, gonadal status, or genetic factors, underlying this phenomenon, and to what extent this may play a role in the sexual dimorphism in cardiovascular disease.

## Supporting Information

S1 Fig Evaluation of microbial diversity using a variety of alpha diversity metrics. Rarefaction curves were generated using phylogenetic metrics (a,d,g,j) and non-phylogenetic metrics (b,c,e,f,h,i,k,l). Horizontal and vertical axes represent rarefaction depth and alpha diversity values, respectively. Error bars correspond to standard deviation for alpha diversity values at each rarefaction depth. Rarefaction curves for gut microbiome richness estimated by gender in all subject (a,b,c), in subject with a BMI lower than 30 (d,e,f), in subject with BMI equal or greater than 30 and equal or less than 33 (g,h,i), and in subject with a BMI greater than 33 (j,k.l).
(PPTX)

S2 Fig Evaluation of microbial diversity through beta-diversity, including unweighted and weighted UniFrac measures. 3D PCoA Plots were generated using quantitative measures (unweighted unifrac) and qualitative measures (weighted unifrac). Proportion of variance explained by each principal coordinate axis is denoted in the corresponding axis label. Beta diversity was estimated by gender in all subject (a,b), in subject with a BMI lower than 30 (c,d), in subject with BMI equal or greater than 30 and equal or less than 33 (e,f), and in subject with a BMI greater than 33 (g,h).
(PPTX)

S3 Fig The number of OTUs (a) or taxonomies (b) associated with TG, HDL, LDL, cholesterol and BMI at FD < 0.05, and their overlaps with each other. BMI indicates body mass index; TG, triglycerides; HDL, high-density lipoprotein; LDL, low-density lipoprotein; and TC, total cholesterol.
(PPTX)

S4 Fig Contribution of the gut microbiome to body mass index and lipids. Variation explained by gut microbes at different levels of significance. BMI indicates body mass index; TG, triglycerides; HDL, high-density lipoprotein; LDL, low-density lipoprotein; and TC, total cholesterol.
(PPTX)

S1 Table Metabolic characteristic of the participants in the study. Values correspond to the mean±SEM of the main metabolic variables. The statistical differences between groups were evaluated by One-way ANOVA. N, 39 men and 36 women. BMI < 30 group, 13 men and 13 women; 30 ≤ BMI ≤ 33 group, 13 men and 10 women; and BMI > 33 group, 13 men and 13 women.
(DOCX)

S2 Table Dietary assessment of the participant in the study. Values correspond to the mean±SEM of a 14-item questionnaire to assess adherence to the Mediterranean Diet and a 9-point score to assess adherence to low-fat diet. Fiber intake was calculated using the Spanish food composition tables. The statistical differences between groups were evaluated by One-way ANOVA. N, 39 men and 36 women. BMI < 30 group, 13 men and 13 women; 30 ≤ BMI ≤ 33 group, 13 men and 10 women; and BMI > 33 group, 13 men and 13 women.
(DOCX)

S3 Table Macronutrients intake of the participant in the study. Values correspond to the mean±SEM. Macronutrient percentage from total energy (E) intake was calculated using the Spanish food composition tables and food frequency questionnaires. The statistical differences between groups were evaluated by One-way ANOVA. N, 39 men and 36 women. BMI < 30 group, 13 men and 13 women; 30 < BMI < 33 group, 13 men and 10 women; and BMI > 33 group, 13 men and 13 women.
(DOCX)

S4 Table OTUs associated with Body mass index (a), triglycerides (b); HDL, high-density lipoprotein (c); LDL, low-density lipoprotein (d); and TC, total cholesterol (e) at FDR < 0.05 level. (PDF)

S5 Table Taxonomies associated with Body mass index (a), triglycerides (b); HDL, high-density lipoprotein (c); LDL, low-density lipoprotein (d); and TC, total cholesterol (e) at FDR < 0.05 level. (PDF)
