# Supplementary material for: Intestinal Microbiota Is Influenced by Gender and Body Mass Index
Source: PLoS One. 2016 May 26;11(5):e0154090. doi: 10.1371/journal.pone.0154090 (PMC4881937; doi:10.1371/journal.pone.0154090)
Supplement: S1 Fig — Rarefaction curves were generated using phylogenetic metrics (a,d,g,j) and non-phylogenetic metrics (b,c,e,f,h,i,k,l). Horizontal and vertical axes represent rarefaction depth and alpha diversity values, respectively. Error bars correspond to standard deviation for alpha diversity values at each rarefaction depth. Rarefaction curves for gut microbiome richness estimated by gender in all subject (a,b,c), in subject with a BMI lower than 30 (d,e,f), in subject with BMI equal or greater than 30 and equal or less than 33 (g,h,i), and in subject with a BMI greater than 33 (j,k.l). (PPTX) [file pone.0154090.s001.pptx]

## Slide 1
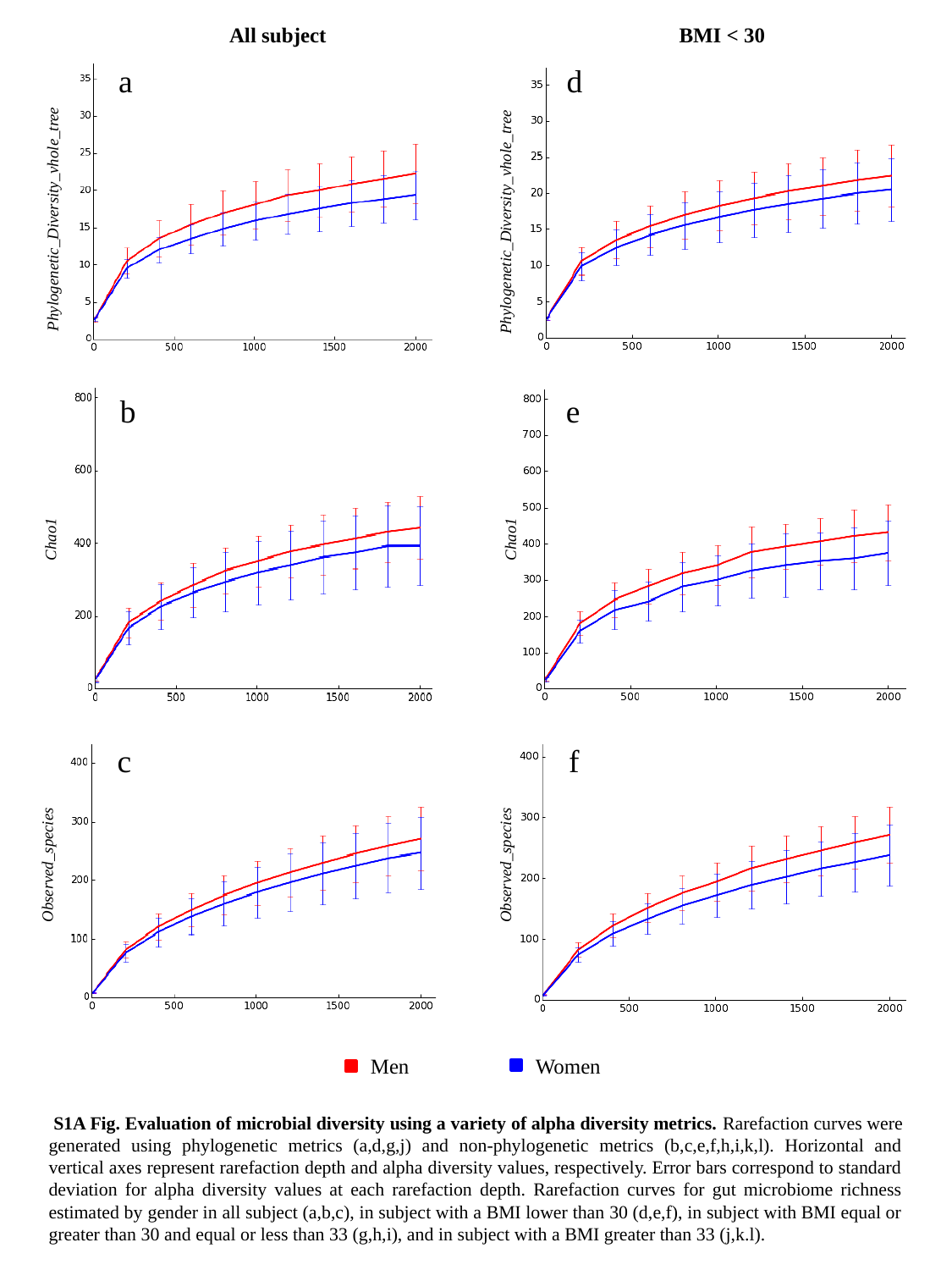

All subject
BMI < 30
a
d
Phylogenetic_Diversity_vhole_tree
Phylogenetic_Diversity_vhole_tree
b
e
Chao1
Chao1
c
f
Observed_species
Observed_species
Men
Women
 S1A Fig. Evaluation of microbial diversity using a variety of alpha diversity metrics. Rarefaction curves were generated using phylogenetic metrics (a,d,g,j) and non-phylogenetic metrics (b,c,e,f,h,i,k,l). Horizontal and vertical axes represent rarefaction depth and alpha diversity values, respectively. Error bars correspond to standard deviation for alpha diversity values at each rarefaction depth. Rarefaction curves for gut microbiome richness estimated by gender in all subject (a,b,c), in subject with a BMI lower than 30 (d,e,f), in subject with BMI equal or greater than 30 and equal or less than 33 (g,h,i), and in subject with a BMI greater than 33 (j,k.l).

## Slide 2
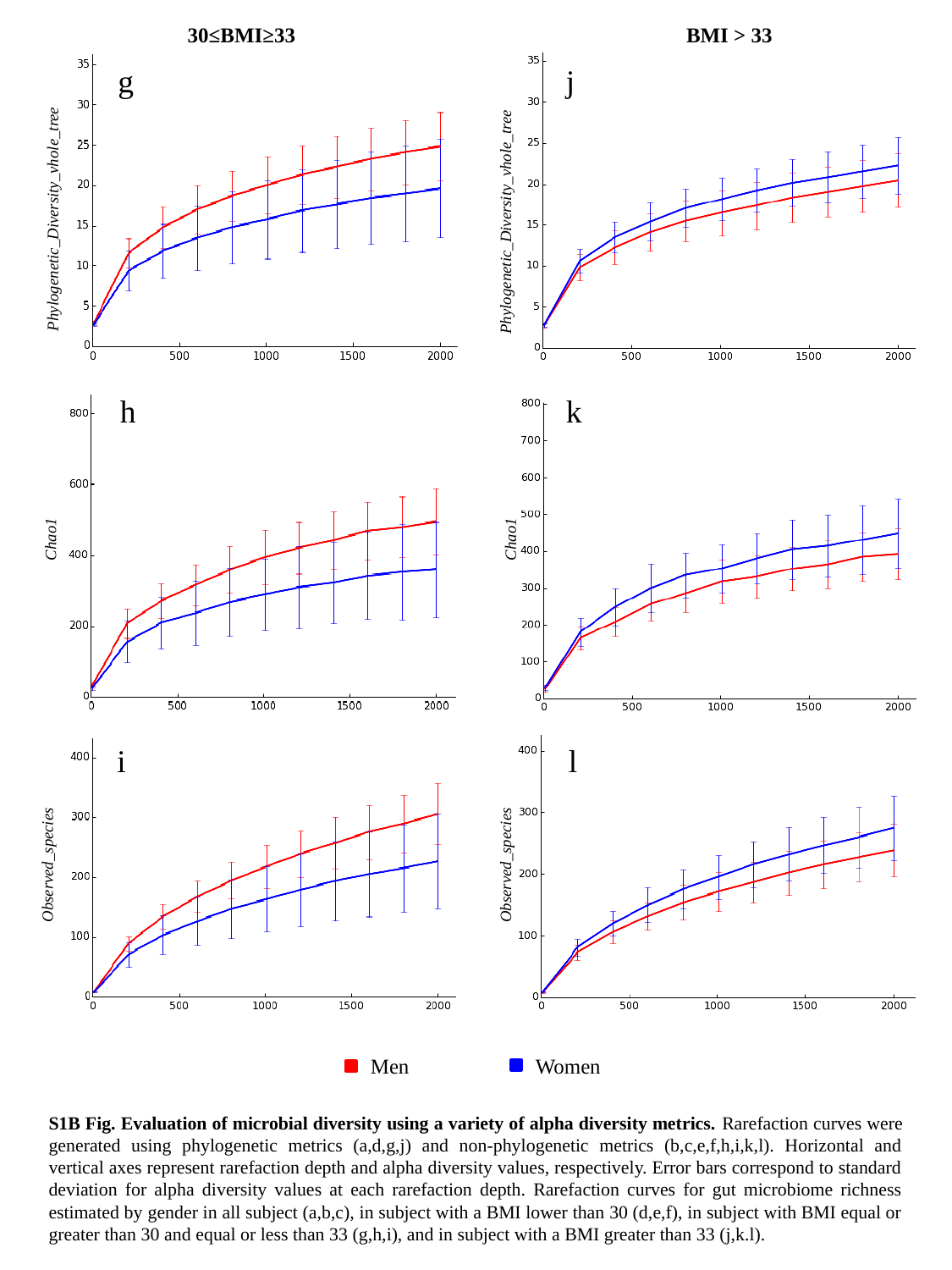

30≤BMI≥33
BMI > 33
g
j
Phylogenetic_Diversity_vhole_tree
Phylogenetic_Diversity_vhole_tree
h
k
Chao1
Chao1
i
l
Observed_species
Observed_species
Men
Women
S1B Fig. Evaluation of microbial diversity using a variety of alpha diversity metrics. Rarefaction curves were generated using phylogenetic metrics (a,d,g,j) and non-phylogenetic metrics (b,c,e,f,h,i,k,l). Horizontal and vertical axes represent rarefaction depth and alpha diversity values, respectively. Error bars correspond to standard deviation for alpha diversity values at each rarefaction depth. Rarefaction curves for gut microbiome richness estimated by gender in all subject (a,b,c), in subject with a BMI lower than 30 (d,e,f), in subject with BMI equal or greater than 30 and equal or less than 33 (g,h,i), and in subject with a BMI greater than 33 (j,k.l).
