# Supplementary material for: Intestinal Microbiota Is Influenced by Gender and Body Mass Index
Source: PLoS One. 2016 May 26;11(5):e0154090. doi: 10.1371/journal.pone.0154090 (PMC4881937; doi:10.1371/journal.pone.0154090)
Supplement: S2 Fig — 3D PCoA Plots were generated using quantitative measures (unweighted unifrac) and qualitative measures (weighted unifrac). Proportion of variance explained by each principal coordinate axis is denoted in the corresponding axis label. Beta diversity was estimated by gender in all subject (a,b), in subject with a BMI lower than 30 (c,d), in subject with BMI equal or greater than 30 and equal or less than 33 (e,f), and in subject with a BMI greater than 33 (g,h). (PPTX) [file pone.0154090.s002.pptx]

## Slide 1
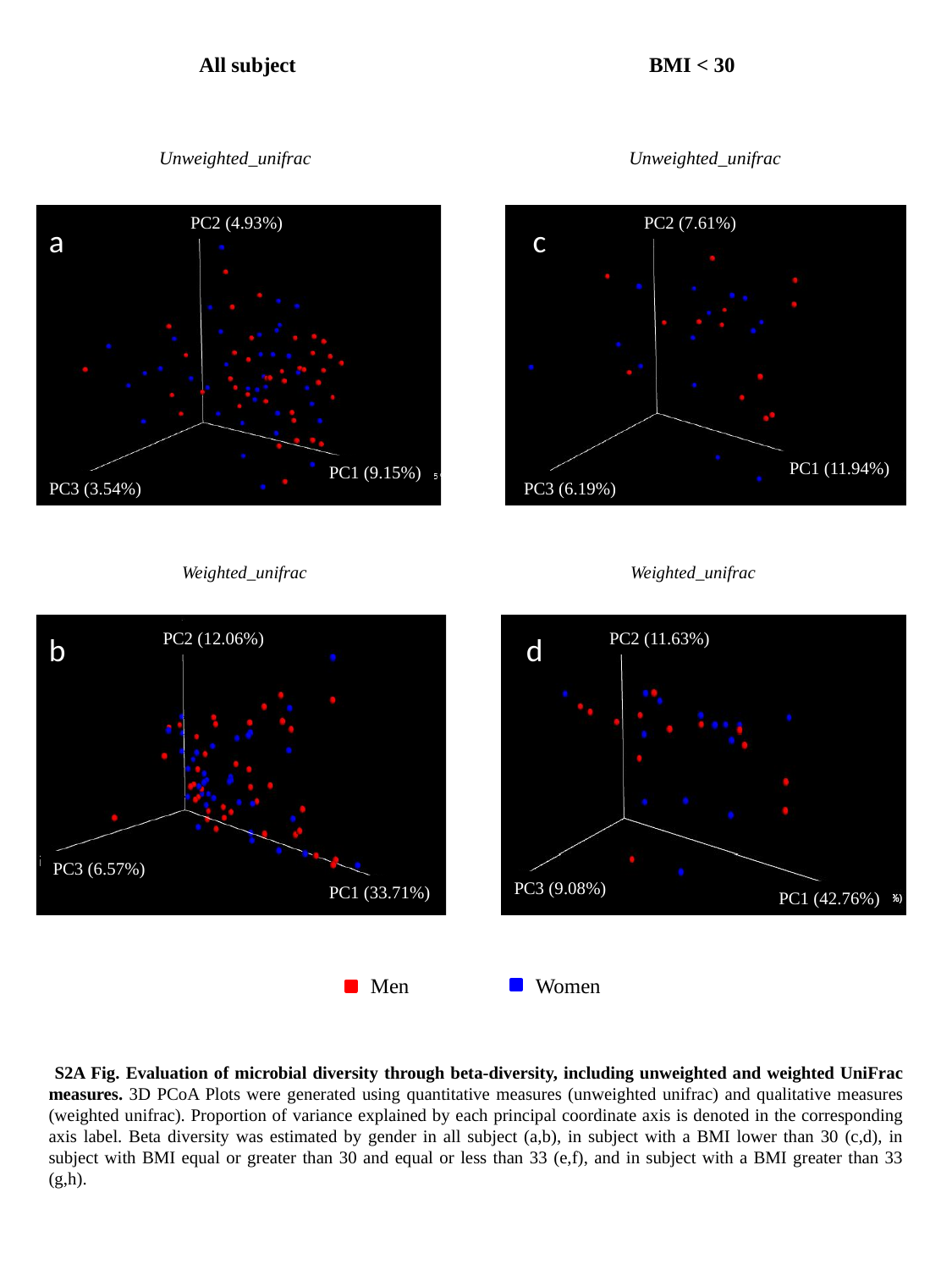

All subject
BMI < 30
Unweighted_unifrac
Unweighted_unifrac
PC2 (4.93%)
PC2 (7.61%)
a
c
c
d
PC1 (11.94%)
PC1 (9.15%)
PC3 (3.54%)
PC3 (6.19%)
Weighted_unifrac
Weighted_unifrac
PC2 (12.06%)
PC2 (11.63%)
b
d
PC3 (6.57%)
PC3 (9.08%)
PC1 (33.71%)
PC1 (42.76%)
Men
Women
 S2A Fig. Evaluation of microbial diversity through beta-diversity, including unweighted and weighted UniFrac measures. 3D PCoA Plots were generated using quantitative measures (unweighted unifrac) and qualitative measures (weighted unifrac). Proportion of variance explained by each principal coordinate axis is denoted in the corresponding axis label. Beta diversity was estimated by gender in all subject (a,b), in subject with a BMI lower than 30 (c,d), in subject with BMI equal or greater than 30 and equal or less than 33 (e,f), and in subject with a BMI greater than 33 (g,h).

## Slide 2
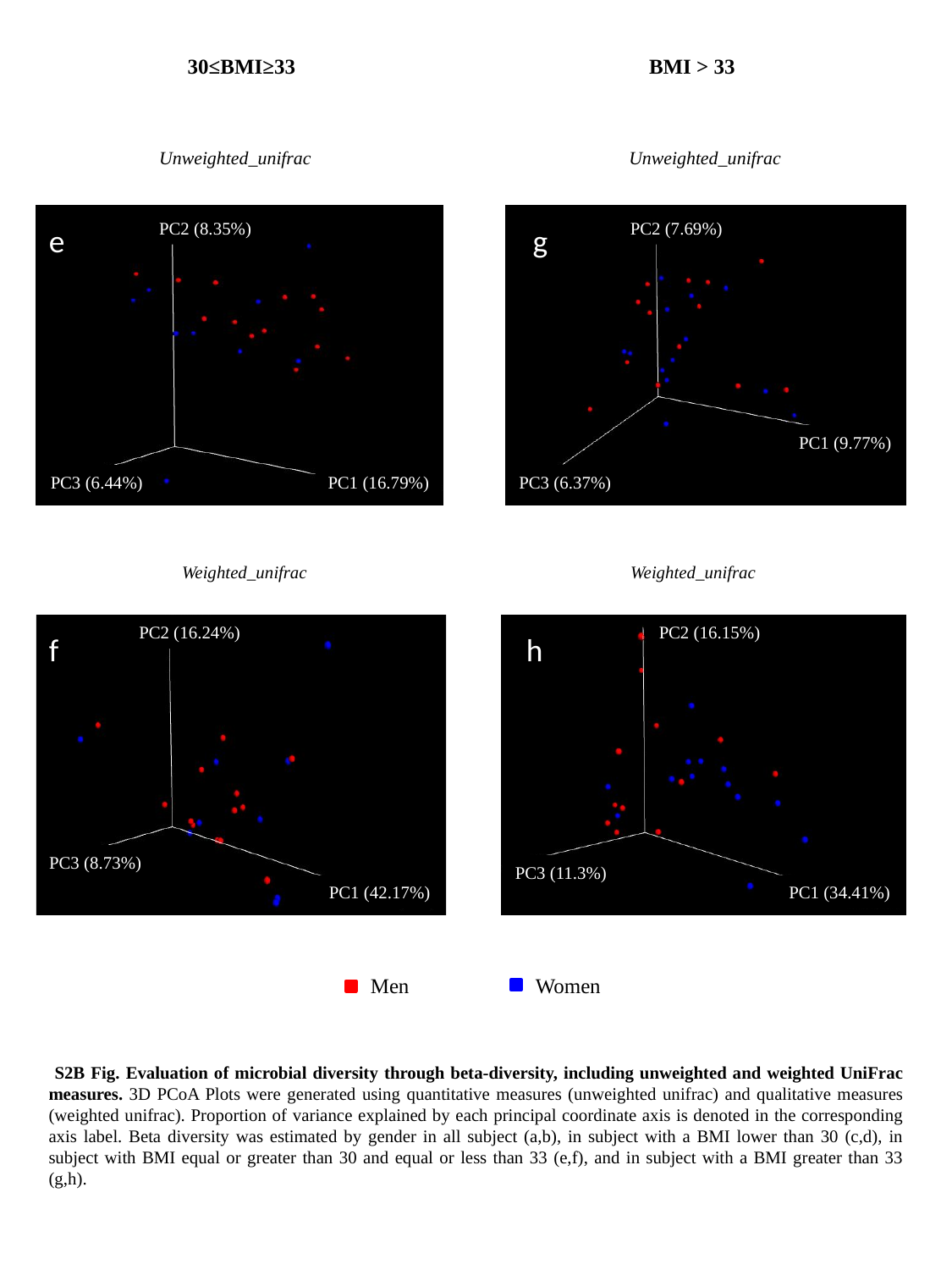

30≤BMI≥33
BMI > 33
Unweighted_unifrac
Unweighted_unifrac
PC2 (8.35%)
PC2 (7.69%)
e
g
PC1 (9.77%)
PC3 (6.44%)
PC1 (16.79%)
PC3 (6.37%)
Weighted_unifrac
Weighted_unifrac
PC2 (16.24%)
PC2 (16.15%)
f
h
PC3 (8.73%)
PC3 (11.3%)
PC1 (42.17%)
PC1 (34.41%)
Men
Women
 S2B Fig. Evaluation of microbial diversity through beta-diversity, including unweighted and weighted UniFrac measures. 3D PCoA Plots were generated using quantitative measures (unweighted unifrac) and qualitative measures (weighted unifrac). Proportion of variance explained by each principal coordinate axis is denoted in the corresponding axis label. Beta diversity was estimated by gender in all subject (a,b), in subject with a BMI lower than 30 (c,d), in subject with BMI equal or greater than 30 and equal or less than 33 (e,f), and in subject with a BMI greater than 33 (g,h).
