# Supplementary material for: Intestinal Microbiota Is Influenced by Gender and Body Mass Index
Source: PLoS One. 2016 May 26;11(5):e0154090. doi: 10.1371/journal.pone.0154090 (PMC4881937; doi:10.1371/journal.pone.0154090)
Supplement: S3 Fig — BMI indicates body mass index; TG, triglycerides; HDL, high-density lipoprotein; LDL, low-density lipoprotein; and TC, total cholesterol. (PPTX) [file pone.0154090.s003.pptx]

## Slide 1
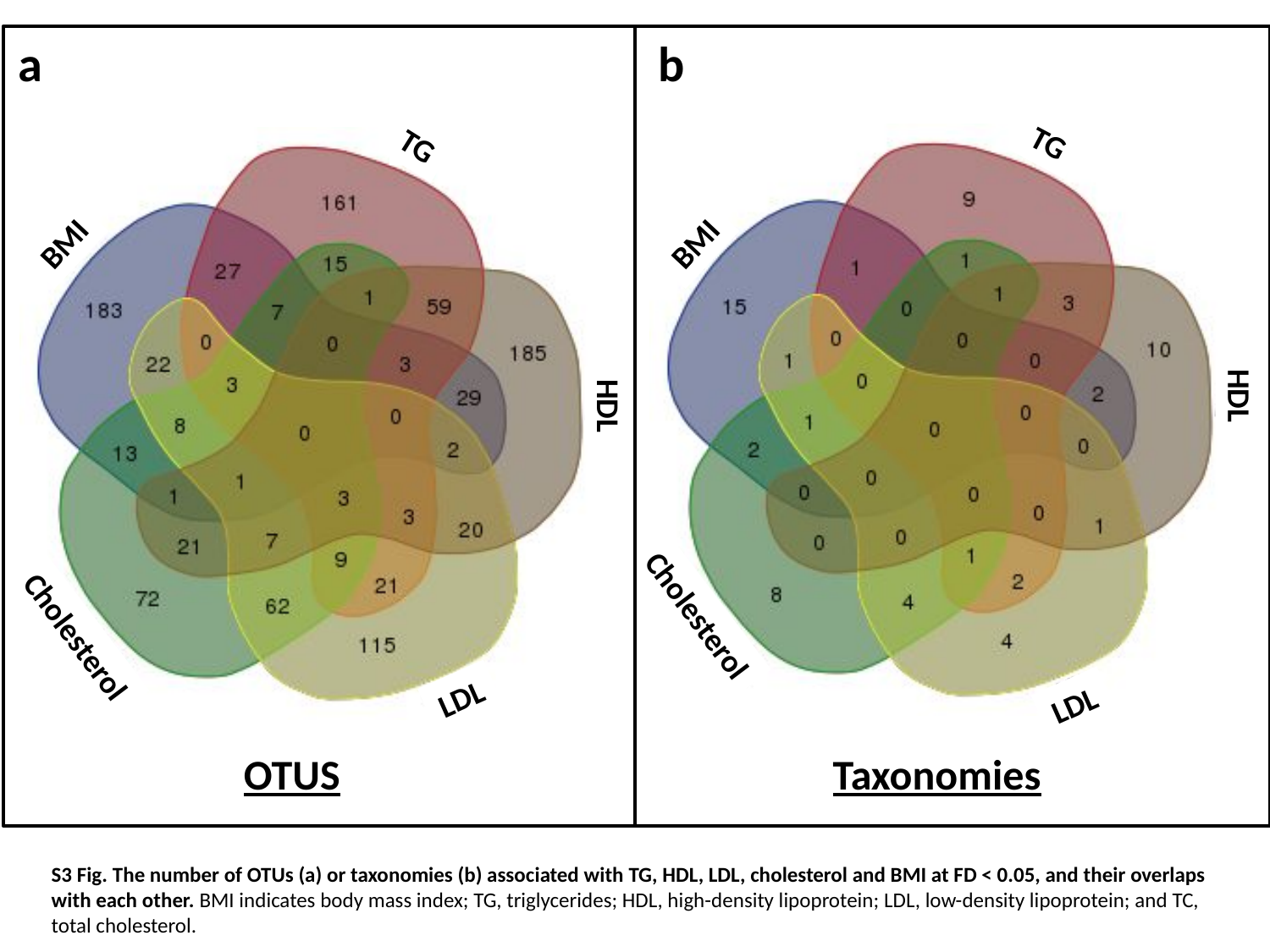

a
b
TG
TG
TG
BMI
BMI
HDL
HDL
Cholesterol
Cholesterol
LDL
LDL
OTUS
Taxonomies
S3 Fig. The number of OTUs (a) or taxonomies (b) associated with TG, HDL, LDL, cholesterol and BMI at FD < 0.05, and their overlaps with each other. BMI indicates body mass index; TG, triglycerides; HDL, high-density lipoprotein; LDL, low-density lipoprotein; and TC, total cholesterol.
