# Supplementary material for: Intestinal Microbiota Is Influenced by Gender and Body Mass Index
Source: PLoS One. 2016 May 26;11(5):e0154090. doi: 10.1371/journal.pone.0154090 (PMC4881937; doi:10.1371/journal.pone.0154090)
Supplement: S4 Fig — Variation explained by gut microbes at different levels of significance. BMI indicates body mass index; TG, triglycerides; HDL, high-density lipoprotein; LDL, low-density lipoprotein; and TC, total cholesterol. (PPTX) [file pone.0154090.s004.pptx]

## Slide 1
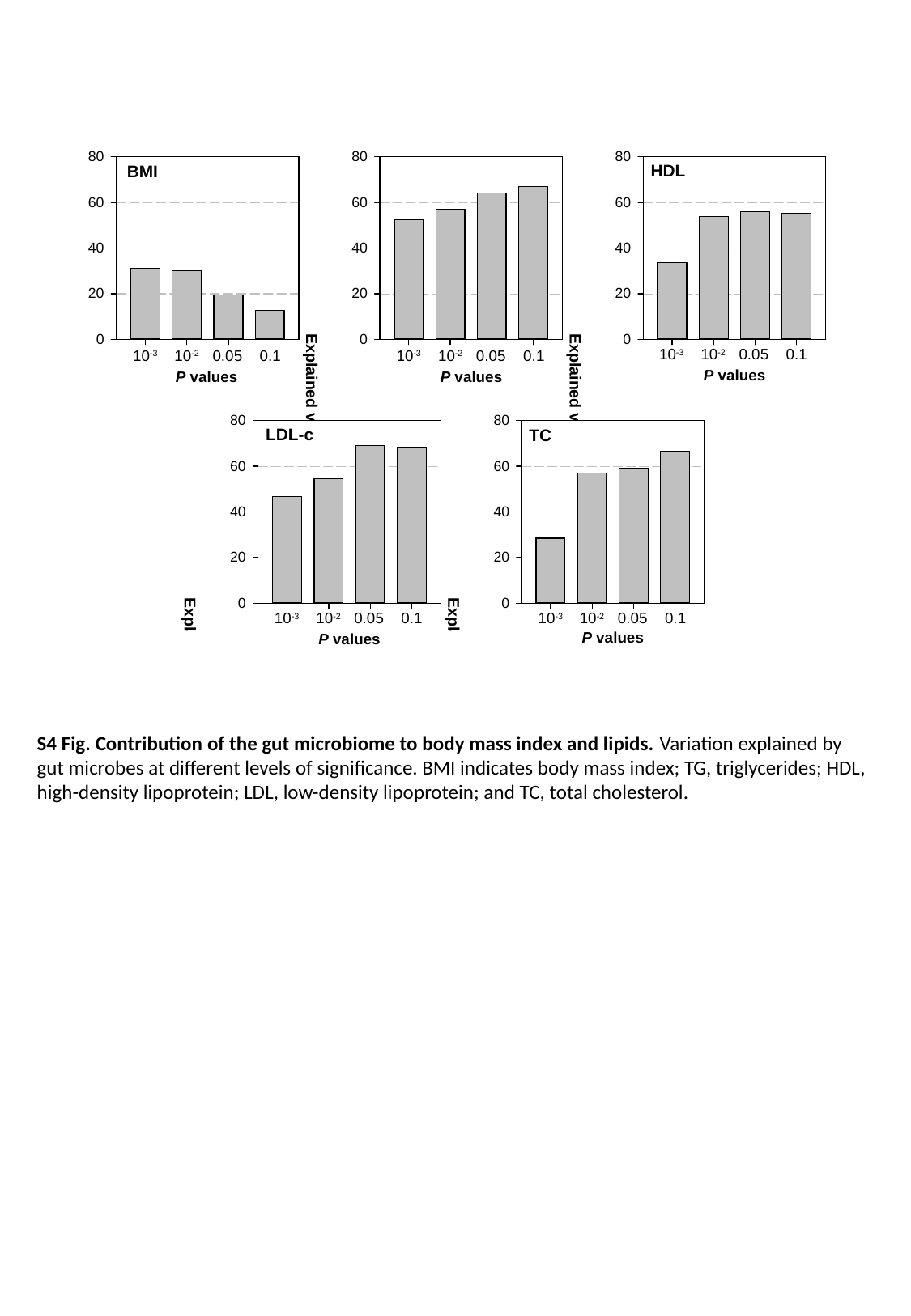

HDL
BMI
10-3
10-2
0.05
0.1
10-3
10-2
0.05
0.1
10-3
10-2
0.05
0.1
P values
P values
P values
LDL-c
TC
10-3
10-2
0.05
0.1
10-3
10-2
0.05
0.1
P values
P values
S4 Fig. Contribution of the gut microbiome to body mass index and lipids. Variation explained by gut microbes at different levels of significance. BMI indicates body mass index; TG, triglycerides; HDL, high-density lipoprotein; LDL, low-density lipoprotein; and TC, total cholesterol.
