# Supplementary material for: Intestinal Microbiota Is Influenced by Gender and Body Mass Index
Source: PLoS One. 2016 May 26;11(5):e0154090. doi: 10.1371/journal.pone.0154090 (PMC4881937; doi:10.1371/journal.pone.0154090)
Supplement: S1 Table — Values correspond to the mean±SEM of the main metabolic variables. The statistical differences between groups were evaluated by One-way ANOVA. N, 39 men and 36 women. BMI < 30 group, 13 men and 13 women; 30 ≤ BMI ≤ 33 group, 13 men and 10 women; and BMI > 33 group, 13 men and 13 women. (DOCX) [file pone.0154090.s005.docx]

| ***Age (y)*** | ***All Subjects*** | ***BMI < 30*** | ***30 ≤ BMI ≤ 33*** | ***BMI > 33*** |
| --- | --- | --- | --- | --- |
| *Men* | 61.15±1.27 | 63.23±1.98 | 58.92±2.42 | 61.31±2.17 |
| *Women* | 60.31±1.40 | 60.15±2.63 | 62.40±2.31 | 58.85±2.32 |
| *P-value* | 0.654 | 0.359 | 0.322 | 0.447 |
| ***BMI (kg/m2)*** | ***All Subjects*** | ***BMI < 30*** | ***30 ≤ BMI ≤ 33*** | ***BMI > 33*** |
| *Men* | 31.44±0.60 | 27.58±0.58 | 31.41±0.28 | 35.33±0.69 |
| *Women* | 31.75±0.90 | 27.03±0.84 | 31.40±0.30 | 36.73±1.35 |
| *P-value* | 0.772 | 0.596 | 0.974 | 0.362 |
| ***Glucose (mg/dl)*** | ***All Subjects*** | ***BMI < 30*** | ***30 ≤ BMI ≤ 33*** | ***BMI > 33*** |
| *Men* | 129.33±7.67 | 132.28±13.78 | 138.69±16.72 | 116.91±8.31 |
| *Women* | 111.46±6.66 | 106.08±7.07 | 98.40±8.05 | 128.17±15.70 |
| *P-value* | 0.088 | 0.102 | 0.620 | 0.536 |
| ***TG (mg/dl)*** | ***All Subjects*** | ***BMI < 30*** | ***30 ≤ BMI ≤ 33*** | ***BMI > 33*** |
| *Men* | 136.50±11.03 | 124.85±21.00 | 143.69±18.35 | 141.33±18.97 |
| *Women* | 154.75±12.63 | 135.31±17.67 | 168.30±24.90 | 163.77±23.82 |
| *P-value* | 0.281 | 0.706 | 0.424 | 0.480 |
| ***HDL-c (mg/dl)*** | ***All Subjects*** | ***BMI < 30*** | ***30 ≤ BMI ≤ 33*** | ***BMI > 33*** |
| *Men* | 39.26±1.16 | 39.85±2.05 | 40.77±1.92 | 37.15±2.07 |
| *Women* | 44.17±1.80 | 46.17±3.64 | 46.50±3.06 | 40.54±2.51 |
| *P-value* | 0.022 | 0.137 | 0.113 | 0.309 |
| ***LDL-c (mg/dl)*** | ***All Subjects*** | ***BMI < 30*** | ***30 ≤ BMI ≤ 33*** | ***BMI > 33*** |
| *Men* | 86.53±3.47 | 76.62±4.24 | 95.31±6.04 | 87.75±6.77 |
| *Women* | 87.22±4.29 | 94.15±9.42 | 87.10±7.55 | 80.38±4.37 |
| *P-value* | 0.900 | 0.102 | 0.400 | 0.376 |
| ***Total Cholesterol (mg/dl)*** | ***All Subjects*** | ***BMI < 30*** | ***30 ≤ BMI ≤ 33*** | ***BMI > 33*** |
| *Men* | 155.13±4.48 | 141.85±5.18 | 165.15±7.90 | 158.38±8.82 |
| *Women* | 159.85±3.58 | 159.45±7.61 | 167.70±5.97 | 154.15±4.93 |
| *P-value* | 0.422 | 0.062 | 0.810 | 0.679 |
| ***Systolic pressure (mm Hg)*** | ***All Subjects*** | ***BMI < 30*** | ***30 ≤ BMI ≤ 33*** | ***BMI > 33*** |
| *Men* | 139.69±2.81 | 141.38±4.67 | 138.08±5.30 | 139.62±4.96 |
| *Women* | 140.92±2.93 | 139.08±4.84 | 144.40±6.10 | 140.08±4.81 |
| *P-value* | 0.764 | 0.735 | 0.442 | 0.947 |
| ***Diastolic pressure (mm Hg)*** | ***All Subjects*** | ***BMI < 30*** | ***30 ≤ BMI ≤ 33*** | ***BMI > 33*** |
| *Men* | 80.08±1.56 | 79.62±2.56 | 81.62±3.39 | 79.00±2.20 |
| *Women* | 74.94±1.83 | 75.31±3.69 | 73.33±2.40 | 75.69±2.97 |
| *P-value* | 0.035 | 0.347 | 0.087 | 0.380 |

**S1 Table. Metabolic characteristic of the participants in the study.** Values correspond to the mean±SEM of the main metabolic variables. The statistical differences between groups were evaluated by One-way ANOVA. N, 39 men and 36 women. BMI < 30 group, 13 men and 13 women; 30 ≤ BMI ≤ 33 group, 13 men and 10 women; and BMI > 33 group, 13 men and 13 women.
