# Supplementary material for: Intestinal Microbiota Is Influenced by Gender and Body Mass Index
Source: PLoS One. 2016 May 26;11(5):e0154090. doi: 10.1371/journal.pone.0154090 (PMC4881937; doi:10.1371/journal.pone.0154090)
Supplement: S2 Table — Values correspond to the mean±SEM of a 14-item questionnaire to assess adherence to the Mediterranean Diet and a 9-point score to assess adherence to low-fat diet. Fiber intake was calculated using the Spanish food composition tables. The statistical differences between groups were evaluated by One-way ANOVA. N, 39 men and 36 women. BMI < 30 group, 13 men and 13 women; 30 ≤ BMI ≤ 33 group, 13 men and 10 women; and BMI > 33 group, 13 men and 13 women. (DOCX) [file pone.0154090.s006.docx]

| ***Mediterranean diet score*** | ***All Subjects*** | ***BMI < 30*** | ***30 ≤ BMI ≤ 33*** | ***BMI > 33*** |
| --- | --- | --- | --- | --- |
| *Men* | 8.41±0.30 | 8.46±0.50 | 8.15±0.53 | 8.62±0.55 |
| *Women* | 7.83±0.27 | 8.31±0.57 | 7.70±0.33 | 7.46±0.40 |
| *P-value* | 0.157 | 0.841 | 0.508 | 0.103 |
| ***Low-fat diet score*** | ***All Subjects*** | ***BMI < 30*** | ***30 ≤ BMI ≤ 33*** | ***BMI > 33*** |
| *Men* | 3.77±0.24 | 4.23±0.44 | 3.92±0.42 | 3.15±0.37 |
| *Women* | 4.11±0.28 | 3.85±0.54 | 4.50±0.37 | 4.08±0.51 |
| *P-value* | 0.360 | 0.587 | 0.328 | 0.158 |
| ***Fiber intake (g/d)*** | ***All Subjects*** | ***BMI < 30*** | ***30 ≤ BMI ≤ 33*** | ***BMI > 33*** |
| *Men* | 24.59±1.06 | 24.25±2.02 | 23.80±1.55 | 25.72±2.01 |
| *Women* | 24.67±1.34 | 24.36±1.80 | 24.12±3.01 | 25.40±2.45 |
| *P-value* | 0.961 | 0.967 | 0.919 | 0.922 |

**S2 Table. Dietary assessment of the participant in the study.** Values correspond to the mean±SEM of a 14-item questionnaire to assess adherence to the Mediterranean Diet and a 9-point score to assess adherence to low-fat diet. Fiber intake was calculated using the Spanish food composition tables. The statistical differences between groups were evaluated by One-way ANOVA. N, 39 men and 36 women. BMI < 30 group, 13 men and 13 women; 30 ≤ BMI ≤ 33 group, 13 men and 10 women; and BMI > 33 group, 13 men and 13 women.
