# Supplementary material for: Intestinal Microbiota Is Influenced by Gender and Body Mass Index
Source: PLoS One. 2016 May 26;11(5):e0154090. doi: 10.1371/journal.pone.0154090 (PMC4881937; doi:10.1371/journal.pone.0154090)
Supplement: S3 Table — Values correspond to the mean±SEM. Macronutrient percentage from total energy (E) intake was calculated using the Spanish food composition tables and food frequency questionnaires. The statistical differences between groups were evaluated by One-way ANOVA. N, 39 men and 36 women. BMI < 30 group, 13 men and 13 women; 30 < BMI < 33 group, 13 men and 10 women; and BMI > 33 group, 13 men and 13 women. (DOCX) [file pone.0154090.s007.docx]

| ***Total energy (Kcal)*** | ***All Subjects*** | ***BMI < 30*** | ***30 ≤ BMI ≤ 33*** | ***BMI > 33*** |
| --- | --- | --- | --- | --- |
| *Men* | 2274.73±74.73 | 2279.81±151.85 | 2196.30±92.75 | 2348.09±142.95 |
| *Women* | 1888.88±57.95 | 1850.11±87.98 | 1982.05±115.75 | 1855.99±104.30 |
| *P-value* | <0.001 | 0.022 | 0.158 | 0.010 |
| ***Proteins (% of E)*** | ***All Subjects*** | ***BMI < 30*** | ***30 ≤ BMI ≤ 33*** | ***BMI > 33*** |
| *Men* | 18.22±0.52 | 18.11±0.82 | 18.80±1.01 | 17.77±0.89 |
| *Women* | 18.97±0.49 | 19.72±0.99 | 18.87±0.75 | 18.30±0.76 |
| *P-value* | 0.301 | 0.222 | 0.957 | 0.654 |
| ***Carbohydrates (% of E)*** | ***All Subjects*** | ***BMI < 30*** | ***30 ≤ BMI ≤ 33*** | ***BMI > 33*** |
| *Men* | 40.91±1.35 | 38.49±2.93 | 41.33±2.04 | 42.92±1.93 |
| *Women* | 44.50±1.17 | 43.21±2.05 | 44.72±2.64 | 45.62±1.62 |
| *P-value* | 0.050 | 0.199 | 0.313 | 0.295 |
| ***Fat (% of E)*** | ***All Subjects*** | ***BMI < 30*** | ***30 ≤ BMI ≤ 33*** | ***BMI > 33*** |
| *Men* | 37.52±1.26 | 38.43±2.89 | 38.19±1.77 | 35.94±1.82 |
| *Women* | 35.97±0.97 | 36.43±1.51 | 35.74±2.48 | 35.67±1.31 |
| *P-value* | 0.337 | 0.546 | 0.418 | 0.907 |

**S3 Table. Macronutrients intake of the participant in the study.** Values correspond to the mean±SEM. Macronutrient percentage from total energy (E) intake was calculated using the Spanish food composition tables and food frequency questionnaires. The statistical differences between groups were evaluated by One-way ANOVA. N, 39 men and 36 women. BMI < 30 group, 13 men and 13 women; 30 < BMI < 33 group, 13 men and 10 women; and BMI > 33 group, 13 men and 13 women.
