# Supplementary material for: Intestinal Microbiota Is Influenced by Gender and Body Mass Index
Source: PLoS One. 2016 May 26;11(5):e0154090. doi: 10.1371/journal.pone.0154090 (PMC4881937; doi:10.1371/journal.pone.0154090)
Supplement: S4 Table — (PDF) [file pone.0154090.s008.pdf]

Table S4a. Associated taxonomies with Body mass index (BMI) at FDR < 0.05 level [a]

	Summary of OTUs reads			Binary model				Quantitative model				Meta analysis		Final association	
OTU	No. Absent	No. Present	mean counts in presents	Estimate	s.e.	*t* value	*P* value	Estimate	s.e.	*t* value	*P* value	Meta z value	*Meta P* value	*z*	*P value*
4212012	64	11	1.18	1.18371	1.52943	0.77395	0.44149	13.27102	1.92120	6.90768	**0.00012**	3.25878	**0.00112**	3.83894	**0.00012**
188348	65	10	4.90	5.29614	1.45878	3.63051	**0.00053**	2.72684	1.22619	2.22382	0.06153	3.77371	**0.00016**	3.77371	**0.00016**
4094259	51	24	6.17	-0.82249	1.15194	-0.71401	0.47753	1.86710	0.43689	4.27359	**0.00034**	2.03236	**0.04212**	3.58447	**0.00034**
4386018	57	18	11.89	4.24149	1.15012	3.68788	**0.00044**	0.33440	0.81125	0.41220	0.68602	2.77310	**0.00555**	3.51750	**0.00044**
165118	35	40	75.98	3.61296	0.99069	3.64691	**0.00050**	-0.12879	0.33146	-0.38856	0.69983	2.18922	**0.02858**	3.48157	**0.00050**
1952	7	68	202.57	6.45276	1.80900	3.56704	**0.00065**	0.27777	0.24021	1.15636	0.25177	3.22251	**0.00127**	3.41124	**0.00065**
4305923	60	15	42.80	2.93900	1.29645	2.26696	**0.02640**	1.15966	0.38415	3.01874	**0.01069**	3.37506	**0.00074**	3.37506	**0.00074**
183439	24	51	94.39	3.72449	1.06210	3.50672	**0.00079**	-0.20164	0.24648	-0.81806	0.41736	1.80093	0.07171	3.35790	**0.00079**
4227110	71	4	11.75	7.83335	2.24623	3.48733	**0.00084**	-2.09345	1.82709	-1.14579	0.45681	1.83608	0.06635	3.34071	**0.00084**
3134492	13	62	139.89	4.53815	1.31139	3.46057	**0.00091**	0.04086	0.24156	0.16914	0.86627	2.46452	**0.01372**	3.31696	**0.00091**
182656	64	11	2.55	-0.42446	1.51451	-0.28026	0.78008	-3.18090	0.66621	-4.77461	**0.00140**	-2.45635	**0.01404**	-3.19459	**0.00140**
4420570	64	11	16.27	4.73987	1.43516	3.30267	**0.00149**	-0.11364	0.56197	-0.20222	0.84479	2.10734	**0.03509**	3.17600	**0.00149**
839964	40	35	12.34	3.36831	1.02351	3.29093	**0.00155**	-0.13990	0.46583	-0.30033	0.76587	2.02775	**0.04259**	3.16546	**0.00155**
182911	39	36	7.17	3.27313	1.00115	3.26936	**0.00165**	0.74047	0.62182	1.19081	0.24222	3.05154	**0.00228**	3.14609	**0.00165**
213566	37	38	25.39	3.23748	1.00138	3.23302	**0.00185**	0.21706	0.40638	0.53414	0.59662	2.57575	**0.01000**	3.11339	**0.00185**
182603	71	4	2.00	5.80901	2.28794	2.53898	**0.01328**	6.28148	0.58718	10.69769	0.05934	3.08431	**0.00204**	3.08431	**0.00204**
229228	67	8	75.88	3.54541	1.68508	2.10400	**0.03887**	1.57907	0.48188	3.27692	**0.02203**	3.07974	**0.00207**	3.07974	**0.00207**
4321810	71	4	7.75	-7.10250	2.23202	-3.18209	**0.00216**	-1.89414	2.93973	-0.64432	0.63562	-2.50407	**0.01228**	-3.06745	**0.00216**
4378683	52	23	15.17	-3.46660	1.08996	-3.18049	**0.00217**	-0.29298	0.64184	-0.45647	0.65297	-2.48593	**0.01292**	-3.06600	**0.00217**
181432	34	41	60.73	3.16982	1.00887	3.14195	**0.00244**	-0.32718	0.35245	-0.92831	0.35911	1.49488	0.13494	3.03115	**0.00244**
227697	70	5	34.20	0.88382	2.14621	0.41181	0.68171	1.33111	0.06889	19.32277	**0.00267**	2.41391	**0.01578**	3.00365	**0.00267**
174516	43	32	22.66	3.10525	1.04540	2.97039	**0.00404**	0.59400	0.42570	1.39533	0.17351	2.99536	**0.00274**	2.99536	**0.00274**
2654263	56	19	5.68	3.56723	1.16986	3.04928	**0.00321**	1.89119	1.40638	1.34473	0.19747	2.99518	**0.00274**	2.99518	**0.00274**
356827	69	6	35.67	0.22292	1.99023	0.11201	0.91113	1.56080	0.17094	9.13053	**0.00278**	2.19418	**0.02822**	2.99143	**0.00278**
302049	58	17	3.94	-3.68755	1.20392	-3.06295	**0.00308**	0.38525	0.61742	0.62397	0.54268	-1.66220	0.09647	-2.95947	**0.00308**
197761	68	7	2.71	2.55979	1.82439	1.40309	0.16489	4.99352	0.91195	5.47562	**0.00541**	2.94872	**0.00319**	2.94872	**0.00319**
4457427	25	50	41.32	0.03437	1.13869	0.03018	0.97600	0.89259	0.29069	3.07058	**0.00355**	2.08322	**0.03723**	2.91604	**0.00355**
177222	43	32	5.00	-1.74233	1.06438	-1.63694	0.10601	-0.88400	0.34026	-2.59802	**0.01458**	-2.87015	**0.00410**	-2.87015	**0.00410**
158722	64	11	3.91	0.92343	1.51876	0.60802	0.54509	4.61883	1.17022	3.94698	**0.00425**	2.44929	**0.01431**	2.85869	**0.00425**
4429981	61	14	38.07	3.82990	1.29883	2.94873	**0.00430**	0.15568	0.77070	0.20199	0.84361	2.15847	**0.03089**	2.85526	**0.00430**
307113	63	12	1.67	-4.07104	1.38137	-2.94711	**0.00432**	1.04041	1.14275	0.91044	0.38632	-1.40535	0.15992	-2.85377	**0.00432**
4045882	63	12	1.67	-4.07104	1.38137	-2.94711	**0.00432**	1.04041	1.14275	0.91044	0.38632	-1.40535	0.15992	-2.85377	**0.00432**
188079	51	24	5.08	2.55770	1.10864	2.30707	**0.02393**	1.50412	0.80515	1.86814	0.07576	2.85253	**0.00434**	2.85253	**0.00434**
179291	68	7	11.29	0.20829	1.84245	0.11305	0.91030	-0.87150	0.14974	-5.82007	**0.00434**	-1.93726	0.05271	-2.85235	**0.00434**
366147	62	13	6.31	3.95561	1.34499	2.94100	**0.00440**	-0.53398	0.81869	-0.65224	0.52895	1.56877	0.11670	2.84819	**0.00440**
167950	70	5	4.80	5.96385	2.02914	2.93910	**0.00442**	-0.17127	2.85008	-0.06009	0.95754	1.97510	**0.04826**	2.84645	**0.00442**
338301	59	16	11.13	-1.34893	1.31826	-1.02326	0.30961	1.85743	0.54159	3.42958	**0.00448**	1.29129	0.19660	2.84219	**0.00448**
3799784	70	5	1.40	0.32812	2.15060	0.15257	0.87916	6.44509	0.43697	14.74943	**0.00457**	2.11300	**0.03460**	2.83621	**0.00457**
4387246	70	5	6.60	5.97544	2.04157	2.92689	**0.00458**	2.88063	1.97320	1.45988	0.28175	2.76596	**0.00568**	2.83526	**0.00458**
320915	69	6	19.33	-0.44244	1.97606	-0.22390	0.82347	-2.27599	0.29909	-7.60971	**0.00471**	-2.15620	**0.03107**	-2.82624	**0.00471**
4428929	69	6	1.83	4.03243	1.91627	2.10431	**0.03884**	3.89414	1.26576	3.07652	0.05428	2.82164	**0.00478**	2.82164	**0.00478**
179508	63	12	1.67	2.17952	1.43812	1.51553	0.13402	4.23522	1.36835	3.09513	**0.01282**	2.81931	**0.00481**	2.81931	**0.00481**
1614788	32	43	7.74	3.00910	1.03487	2.90771	**0.00484**	-0.85397	0.40207	-2.12393	**0.03991**	0.53952	0.58953	2.81768	**0.00484**
4472174	70	5	3.20	-5.34952	2.17440	-2.46023	**0.01629**	-1.62037	0.61095	-2.65221	0.11761	-2.80534	**0.00503**	-2.80534	**0.00503**
4357713	55	20	2.90	3.31381	1.14659	2.89014	**0.00509**	-0.70073	0.90806	-0.77167	0.45090	1.44790	0.14765	2.80155	**0.00509**
363430	67	8	71.88	-0.11196	1.73495	-0.06453	0.94872	-0.50663	0.10825	-4.68001	**0.00543**	-2.01133	**0.04429**	-2.78014	**0.00543**
147702	30	45	27.76	1.94792	1.07011	1.82030	0.07287	0.86086	0.39029	2.20568	**0.03293**	2.77649	**0.00549**	2.77649	**0.00549**
188676	22	53	17.13	1.41192	1.16990	1.20688	0.23143	1.01935	0.35951	2.83537	**0.00659**	2.76734	**0.00565**	2.76734	**0.00565**
186772	35	40	5.33	2.90910	1.02040	2.85092	**0.00568**	0.50955	0.55488	0.91830	0.36441	2.59686	**0.00941**	2.76551	**0.00568**
189548	70	5	2.60	5.72272	2.08610	2.74327	**0.00767**	-4.39082	0.33377	-13.15518	**0.00573**	-0.06840	0.94547	-2.76291	**0.00573**
187180	33	42	14.98	1.54183	1.06927	1.44194	0.15365	-1.00692	0.34567	-2.91298	**0.00590**	-0.93812	0.34818	-2.75344	**0.00590**
4391326	69	6	14.17	-5.33730	1.88204	-2.83591	**0.00593**	0.05655	0.64273	0.08799	0.93543	-1.88846	0.05896	-2.75169	**0.00593**
847934	67	8	1.88	4.66489	1.64624	2.83365	**0.00597**	-0.26458	2.67134	-0.09904	0.92495	1.87766	0.06043	2.74961	**0.00597**
2700883	18	57	11.93	1.97753	1.24813	1.58440	0.11749	-0.95597	0.33571	-2.84756	**0.00622**	-0.82775	0.40781	-2.73602	**0.00622**
183457	55	20	10.75	2.41614	1.17725	2.05236	**0.04377**	1.23952	0.62674	1.97772	0.06440	2.73342	**0.00627**	2.73342	**0.00627**
267718	64	11	2.82	-2.98471	1.47242	-2.02708	**0.04636**	-2.93536	1.38583	-2.11812	0.06702	-2.70373	**0.00686**	-2.70373	**0.00686**
2272791	64	11	2.82	-2.98471	1.47242	-2.02708	**0.04636**	-2.93536	1.38583	-2.11812	0.06702	-2.70373	**0.00686**	-2.70373	**0.00686**
197624	43	32	5.97	-2.86551	1.02988	-2.78236	**0.00689**	0.22237	0.60356	0.36844	0.71522	-1.65284	0.09836	-2.70232	**0.00689**
187388	62	13	2.62	3.73970	1.34581	2.77876	**0.00695**	0.41188	2.09437	0.19666	0.84803	2.04398	**0.04096**	2.69899	**0.00695**
207487	47	28	5.14	2.92705	1.05388	2.77742	**0.00698**	0.44096	0.82750	0.53288	0.59882	2.27960	**0.02263**	2.69775	**0.00698**
182647	63	12	29.67	-2.33134	1.43793	-1.62131	0.10932	-0.76240	0.28888	-2.63913	**0.02695**	-2.69652	**0.00701**	-2.69652	**0.00701**
194297	53	22	35.55	-1.28784	1.16778	-1.10281	0.27378	-0.87342	0.28922	-3.01994	**0.00704**	-2.67935	**0.00738**	-2.69478	**0.00704**
4467347	71	4	3.50	4.51960	2.33792	1.93317	0.05715	-6.97488	0.07929	-87.96415	**0.00724**	-0.55406	0.57954	-2.68574	**0.00724**
299302	66	9	3.33	-4.31559	1.56760	-2.75299	**0.00747**	-0.06452	1.84304	-0.03501	0.97321	-1.91538	0.05544	-2.67518	**0.00747**
3530697	37	38	6.55	2.82965	1.03043	2.74610	**0.00761**	-0.28976	0.55311	-0.52388	0.60366	1.52004	0.12850	2.66880	**0.00761**
346253	69	6	2.67	-3.36047	1.95590	-1.71812	0.09007	-2.06861	0.59181	-3.49540	**0.03961**	-2.65362	**0.00796**	-2.65362	**0.00796**
189450	66	9	2.44	-4.32220	1.58378	-2.72904	**0.00798**	0.10965	0.98529	0.11128	0.91502	-1.80051	0.07178	-2.65301	**0.00798**
347243	71	4	2.25	-0.27906	2.41506	-0.11555	0.90833	-2.24329	0.02838	-79.03470	**0.00805**	-1.95510	0.05057	-2.64978	**0.00805**
196061	68	7	5.57	3.87536	1.86405	2.07900	**0.04118**	4.46167	1.99053	2.24145	0.08848	2.64824	**0.00809**	2.64824	**0.00809**
180215	70	5	2.00	5.60382	2.06372	2.71540	**0.00828**	1.36171	3.62512	0.37563	0.74329	2.09861	**0.03585**	2.64038	**0.00828**
161423	6	69	36.12	-5.10888	1.88283	-2.71341	**0.00833**	-0.03913	0.28604	-0.13680	0.89161	-1.96209	**0.04975**	-2.63854	**0.00833**
4368216	18	57	399.56	2.45075	1.22200	2.00552	**0.04867**	0.25780	0.14391	1.79140	0.07883	2.63680	**0.00837**	2.63680	**0.00837**
4451906	44	31	4.68	1.53600	1.08983	1.40939	0.16302	-2.00576	0.70703	-2.83689	**0.00837**	-0.87795	0.37997	-2.63658	**0.00837**
2953981	67	8	3.50	-0.86319	1.73213	-0.49834	0.61977	2.47816	0.58813	4.21362	**0.00838**	1.51336	0.13019	2.63640	**0.00838**
279048	70	5	26.80	-5.61723	2.07793	-2.70329	**0.00856**	0.78834	1.36483	0.57761	0.62189	-1.51036	0.13095	-2.62915	**0.00856**
185763	3	72	91.36	-0.14715	2.73972	-0.05371	0.95731	0.68401	0.25277	2.70604	**0.00857**	1.82088	0.06863	2.62864	**0.00857**
814442	50	25	69.36	1.80770	1.12916	1.60093	0.11377	0.78838	0.34598	2.27872	**0.03275**	2.62806	**0.00859**	2.62806	**0.00859**

Table S4a. Associated taxonomies with Body mass index (BMI) at FDR < 0.05 level [a] (Cont.)

	Summary of OTUs reads			Binary model				Quantitative model				Meta analysis		Final association	
OTU	No. Absent	No. Present	mean counts in presents	Estimate	s.e.	*t* value	*P* value	Estimate	s.e.	*t* value	*P* value	Meta z value	*Meta P* value	*z*	*P value*
193644	59	16	2.25	0.54398	1.30575	0.41661	0.67820	3.01894	0.98078	3.07810	**0.00881**	2.14553	**0.03191**	2.61932	**0.00881**
186559	68	7	2.29	2.77604	1.87488	1.48065	0.14306	6.11929	1.75793	3.48097	**0.02533**	2.61687	**0.00887**	2.61687	**0.00887**
4403113	71	4	1.75	6.10114	2.27387	2.68315	**0.00904**	-5.01366	0.79608	-6.29796	0.10025	0.68365	0.49420	2.61048	**0.00904**
179760	67	8	2.75	4.44370	1.66469	2.66938	**0.00939**	0.46870	1.44088	0.32529	0.75813	2.05459	**0.03992**	2.59769	**0.00939**
4383924	68	7	2.29	-4.69991	1.76138	-2.66831	**0.00941**	0.94467	1.05375	0.89648	0.42067	-1.26674	0.20525	-2.59670	**0.00941**
2699610	45	30	2.03	2.78080	1.04443	2.66250	**0.00956**	0.62498	0.98588	0.63393	0.53146	2.27482	**0.02292**	2.59130	**0.00956**
197988	42	33	14.00	1.68549	1.06088	1.58877	0.11649	1.04809	0.47858	2.19000	**0.03644**	2.58921	**0.00962**	2.58921	**0.00962**
184426	46	29	5.07	2.80765	1.05562	2.65971	**0.00963**	0.09584	0.64757	0.14800	0.88349	1.93412	0.05310	2.58871	**0.00963**
4438983	49	26	30.96	-0.44387	1.13651	-0.39056	0.69728	-1.12546	0.39977	-2.81525	**0.00982**	-2.10089	**0.03565**	-2.58212	**0.00982**
197460	68	7	1.86	0.37402	1.84209	0.20304	0.83967	2.11479	0.45795	4.61792	**0.00990**	1.96698	**0.04919**	2.57942	**0.00990**
322835	38	37	7.84	1.35967	1.06246	1.27973	0.20475	1.65720	0.66344	2.49790	**0.01749**	2.57692	**0.00997**	2.57692	**0.00997**
4326573	70	5	11.00	5.40927	2.05529	2.63188	**0.01038**	-1.42962	1.91698	-0.74577	0.53355	1.37195	0.17008	2.56283	**0.01038**
4354235	10	65	52.98	3.97183	1.51090	2.62878	**0.01047**	0.09018	0.26262	0.34339	0.73246	2.05188	**0.04018**	2.55995	**0.01047**
327218	29	46	7.09	2.06293	1.07324	1.92214	0.05854	1.06951	0.60459	1.76898	0.08399	2.55944	**0.01048**	2.55944	**0.01048**
195294	53	22	2.59	1.80765	1.20818	1.49618	0.13898	2.50751	1.08815	2.30437	**0.03266**	2.55680	**0.01056**	2.55680	**0.01056**
186888	51	24	8.71	-2.89134	1.10528	-2.61593	**0.01083**	-0.15710	0.66074	-0.23777	0.81437	-1.96773	**0.04910**	-2.54799	**0.01083**
366451	66	9	4.89	1.08229	1.64550	0.65773	0.51281	3.03207	0.83338	3.63829	**0.01086**	2.26397	**0.02358**	2.54727	**0.01086**
182142	70	5	1.60	5.39933	2.06729	2.61179	**0.01095**	-0.19330	5.23494	-0.03692	0.97390	1.77584	0.07576	2.54413	**0.01095**
4350477	57	18	5.28	-3.16682	1.21481	-2.60683	**0.01110**	0.13757	0.84575	0.16266	0.87296	-1.68264	0.09245	-2.53951	**0.01110**
841907	52	23	37.48	-2.89859	1.11508	-2.59946	**0.01132**	0.06219	0.35625	0.17456	0.86318	-1.66899	0.09512	-2.53264	**0.01132**
313593	25	50	6.04	2.83083	1.08934	2.59866	**0.01134**	-0.70017	0.52193	-1.34150	0.18621	0.85561	0.39222	2.53190	**0.01134**
191660	69	6	1.67	4.91088	1.89013	2.59817	**0.01136**	-5.72879	1.19539	-4.79241	**0.01728**	0.10663	0.91508	2.53145	**0.01136**
193161	71	4	1.25	-3.27708	2.35336	-1.39251	0.16805	-14.03829	0.64380	-21.80544	**0.02918**	-2.51702	**0.01184**	-2.51702	**0.01184**
177515	14	61	56.00	3.40074	1.31714	2.58191	**0.01186**	-0.20488	0.32163	-0.63701	0.52663	1.33156	0.18300	2.51628	**0.01186**
4232048	66	9	1.89	0.42156	1.66417	0.25332	0.80075	3.62250	1.03070	3.51460	**0.01260**	1.94259	0.05207	2.49485	**0.01260**
321063	68	7	2.43	4.32975	1.78212	2.42955	**0.01761**	2.38980	1.78193	1.34113	0.25097	2.49019	**0.01277**	2.49019	**0.01277**
2829179	38	37	331.81	2.70483	1.06660	2.53594	**0.01338**	0.23528	0.22186	1.06049	0.29640	2.48728	**0.01287**	2.48728	**0.01287**
4456702	63	12	1.92	-0.37885	1.46176	-0.25918	0.79624	-6.07388	1.97268	-3.07900	**0.01316**	-1.93577	0.05290	-2.47937	**0.01316**
4484075	38	37	4.03	2.25627	1.04077	2.16788	**0.03347**	1.01713	0.72557	1.40184	0.17003	2.47379	**0.01337**	2.47379	**0.01337**
187751	45	30	57.10	0.82696	1.09118	0.75786	0.45101	0.58955	0.22314	2.64209	**0.01354**	2.27897	**0.02267**	2.46922	**0.01354**
180771	61	14	26.00	2.76157	1.33939	2.06181	**0.04284**	0.89786	0.56987	1.57555	0.14343	2.46669	**0.01364**	2.46669	**0.01364**
3443092	68	7	1.71	3.09503	1.88560	1.64141	0.10507	4.15042	1.63068	2.54521	0.06363	2.45756	**0.01399**	2.45756	**0.01399**
3785400	69	6	56.67	-3.22392	1.93797	-1.66355	0.10055	-0.70250	0.25114	-2.79729	0.06801	-2.45166	**0.01422**	-2.45166	**0.01422**
4301511	33	42	11.90	1.66505	1.06256	1.56702	0.12149	0.95054	0.48087	1.97673	0.05517	2.45086	**0.01425**	2.45086	**0.01425**
194215	57	18	4.33	1.46236	1.26517	1.15586	0.25156	1.54079	0.59768	2.57794	**0.02100**	2.44274	**0.01458**	2.44274	**0.01458**
2423305	57	18	7.61	-0.35599	1.26202	-0.28208	0.77869	-2.03083	0.73683	-2.75618	**0.01470**	-1.92379	0.05438	-2.43963	**0.01470**
1522739	71	4	2.25	-3.11914	2.35569	-1.32409	0.18966	-3.25676	0.16729	-19.46738	**0.03267**	-2.43786	**0.01477**	-2.43786	**0.01477**
179583	67	8	1.75	-0.37365	1.73444	-0.21543	0.83004	2.50601	0.69359	3.61311	**0.01533**	1.56259	0.11815	2.42449	**0.01533**
199279	55	20	1.95	-0.20446	1.23433	-0.16565	0.86890	-1.69420	0.63328	-2.67530	**0.01598**	-1.82044	0.06869	-2.40943	**0.01598**
270094	23	52	35.77	1.76807	1.14326	1.54651	0.12636	0.58372	0.30352	1.92315	0.06028	2.40934	**0.01598**	2.40934	**0.01598**
188047	12	63	20.89	3.49434	1.41705	2.46593	**0.01605**	0.31878	0.34067	0.93574	0.35316	2.35911	**0.01832**	2.40780	**0.01605**
189356	67	8	2.50	3.33224	1.72170	1.93544	0.05686	2.18251	1.22496	1.78170	0.13490	2.40375	**0.01623**	2.40375	**0.01623**
189877	70	5	2.40	5.31492	2.16467	2.45530	**0.01649**	2.26269	6.79115	0.33318	0.77068	1.90163	0.05722	2.39783	**0.01649**
326626	56	19	6.32	2.42131	1.19792	2.02126	**0.04697**	-2.02396	0.75736	-2.67240	**0.01669**	-0.28775	0.77354	-2.39350	**0.01669**
171559	61	14	1.57	0.80844	1.37471	0.58808	0.55832	-4.85406	1.72505	-2.81387	**0.01685**	-1.27604	0.20194	-2.38993	**0.01685**
194001	50	25	3.64	2.65948	1.09225	2.43485	**0.01738**	1.01235	1.06522	0.95037	0.35225	2.33972	**0.01930**	2.37863	**0.01738**
4469576	29	46	37.48	-1.84447	1.08061	-1.70687	0.09216	-0.56929	0.33307	-1.70923	0.09462	-2.37281	**0.01765**	-2.37281	**0.01765**
196371	69	6	3.33	-1.04039	1.99530	-0.52142	0.60367	-3.59689	0.75903	-4.73883	**0.01782**	-2.04247	**0.04110**	-2.36936	**0.01782**
177032	70	5	2.20	-3.75968	2.20948	-1.70161	0.09314	-3.65150	1.21213	-3.01245	0.09479	-2.36861	**0.01786**	-2.36861	**0.01786**
4410097	68	7	4.14	-3.20432	1.80202	-1.77818	0.07960	-2.54222	1.24918	-2.03511	0.11156	-2.36474	**0.01804**	-2.36474	**0.01804**
354850	62	13	2.77	0.44971	1.44839	0.31049	0.75708	-2.41990	0.85712	-2.82331	**0.01806**	-1.45319	0.14617	-2.36443	**0.01806**
4362300	70	5	2.60	1.07810	2.17201	0.49636	0.62115	4.93291	0.67380	7.32100	**0.01815**	2.02002	**0.04338**	2.36252	**0.01815**
194373	68	7	9.43	4.32862	1.79688	2.40896	**0.01856**	-0.58165	0.95461	-0.60930	0.57523	1.26850	0.20462	2.35429	**0.01856**
195937	27	48	9.63	2.59412	1.08004	2.40188	**0.01889**	-0.86391	0.44131	-1.95761	0.05650	0.31144	0.75547	2.34764	**0.01889**
158711	71	4	36.00	0.56522	2.39650	0.23585	0.81422	0.41946	0.01292	32.47746	**0.01960**	1.81655	0.06929	2.33400	**0.01960**
192244	58	17	5.65	2.94711	1.23888	2.37884	**0.02002**	-0.56350	0.90197	-0.62474	0.54219	1.21371	0.22486	2.32595	**0.02002**
1992	14	61	12.31	-1.38668	1.36911	-1.01284	0.31453	-0.82306	0.35135	-2.34255	**0.02261**	-2.32341	**0.02016**	-2.32341	**0.02016**
3203801	45	30	7.33	-1.61979	1.07734	-1.50351	0.13708	-0.99999	0.53477	-1.86994	0.07237	-2.32180	**0.02024**	-2.32180	**0.02024**
759816	68	7	5.43	-3.58119	1.79231	-1.99808	**0.04949**	-5.84242	3.75454	-1.55610	0.19467	-2.30607	**0.02111**	-2.30607	**0.02111**
4300690	42	33	100.88	0.73970	1.08021	0.68477	0.49569	-0.68648	0.28208	-2.43363	**0.02112**	-1.14875	0.25066	-2.30587	**0.02112**
185597	55	20	4.15	2.82025	1.19641	2.35726	**0.02113**	-1.06295	0.95894	-1.10847	0.28310	0.87132	0.38358	2.30561	**0.02113**
189708	67	8	2.25	0.35494	1.73802	0.20422	0.83876	4.30512	1.29985	3.31201	**0.02119**	1.77343	0.07616	2.30452	**0.02119**
4409280	16	59	162.36	1.37326	1.29732	1.05854	0.29335	0.58237	0.25718	2.26446	**0.02744**	2.30238	**0.02131**	2.30238	**0.02131**
3943182	17	58	10.81	-2.64395	1.25613	-2.10484	**0.03880**	-0.39565	0.33018	-1.19827	0.23595	-2.29917	**0.02150**	-2.29917	**0.02150**
3474081	70	5	2.00	2.44971	2.12748	1.15147	0.25335	-2.83956	0.42764	-6.64013	**0.02194**	-0.81262	0.41644	-2.29146	**0.02194**
730939	71	4	2.50	-1.57077	2.37819	-0.66049	0.51105	11.28613	0.38976	28.95683	**0.02198**	1.15511	0.24805	2.29078	**0.02198**
2148365	51	24	7.92	-2.59118	1.10688	-2.34098	**0.02201**	-0.21287	0.79136	-0.26899	0.79056	-1.80725	0.07072	-2.29026	**0.02201**
4480359	28	47	8.09	2.49700	1.06786	2.33831	**0.02215**	-0.05023	0.52469	-0.09572	0.92418	1.55038	0.12105	2.28775	**0.02215**
175922	69	6	2.67	-3.16682	1.94133	-1.63126	0.10720	-6.18878	2.68938	-2.30119	0.10487	-2.28577	**0.02227**	-2.28577	**0.02227**
146554	20	55	91.85	1.53237	1.20040	1.27655	0.20586	0.57057	0.28390	2.00977	**0.04966**	2.28248	**0.02246**	2.28248	**0.02246**
111135	70	5	11.00	-0.27657	2.14717	-0.12881	0.89787	0.74552	0.11415	6.53115	**0.02265**	1.52094	0.12827	2.27929	**0.02265**
311950	43	32	19.56	2.20664	1.05652	2.08860	**0.04028**	0.59773	0.50018	1.19502	0.24176	2.27791	**0.02273**	2.27791	**0.02273**
189292	64	11	6.00	3.41102	1.46566	2.32729	**0.02277**	-0.78963	1.21611	-0.64931	0.53433	1.17093	0.24163	2.27735	**0.02277**
368236	67	8	2.13	-1.74632	1.72487	-1.01243	0.31472	-2.47027	0.79614	-3.10279	**0.02677**	-2.27706	**0.02278**	-2.27706	**0.02278**
195465	41	34	3.88	0.91119	1.07966	0.84396	0.40149	-1.40900	0.58815	-2.39567	**0.02281**	-1.01650	0.30939	-2.27652	**0.02281**
289734	19	56	17.04	-0.09343	1.23150	-0.07587	0.93973	-0.82653	0.35264	-2.34383	**0.02287**	-1.66256	0.09640	-2.27561	**0.02287**
4458576	23	52	72.92	2.61906	1.12687	2.32420	**0.02294**	0.00514	0.30655	0.01678	0.98668	1.62007	0.10522	2.27443	**0.02294**

Table S4a. Associated taxonomies with Body mass index (BMI) at FDR < 0.05 level [a] (Cont.)

	Summary of OTUs reads			Binary model				Quantitative model				Meta analysis		Final association	
OTU	No. Absent	No. Present	mean counts in presents	Estimate	s.e.	*t* value	*P* value	Estimate	s.e.	*t* value	*P* value	Meta z value	*Meta P* value	*z*	*P value*
363442	70	5	1.60	4.81240	2.07152	2.32312	**0.02300**	-5.75146	3.09677	-1.85725	0.20440	0.71014	0.47762	2.27341	**0.02300**
3544699	68	7	4.43	-4.12458	1.77573	-2.32275	**0.02302**	-1.68556	1.78519	-0.94419	0.39853	-2.20427	**0.02751**	-2.27306	**0.02302**
187924	46	29	5.93	2.46750	1.06246	2.32244	**0.02304**	-1.37151	0.68744	-1.99509	0.05661	0.25914	0.79553	2.27277	**0.02304**
179319	67	8	2.50	-3.93430	1.69674	-2.31874	**0.02325**	2.58751	1.67415	1.54557	0.18287	-0.66278	0.50747	-2.26927	**0.02325**
211750	71	4	14.25	4.34059	2.32791	1.86459	0.06632	1.62204	0.44496	3.64533	0.17045	2.26773	**0.02335**	2.26773	**0.02335**
302333	63	12	2.50	1.24252	1.45756	0.85247	0.39678	-3.03195	1.11513	-2.71893	**0.02365**	-1.00083	0.31691	-2.26278	**0.02365**
47365	70	5	6.00	0.40116	2.16074	0.18566	0.85323	1.32934	0.20837	6.37964	**0.02370**	1.73025	0.08358	2.26196	**0.02370**
181140	59	16	3.06	-0.90595	1.30813	-0.69255	0.49082	1.97458	0.77120	2.56039	**0.02372**	1.11200	0.26614	2.26161	**0.02372**
4301298	44	31	50.84	0.64005	1.09457	0.58475	0.56054	-0.76522	0.32021	-2.38978	**0.02383**	-1.18638	0.23547	-2.25983	**0.02383**
198449	20	55	15.67	-1.01631	1.21113	-0.83915	0.40416	-0.76039	0.32669	-2.32760	**0.02386**	-2.18749	**0.02871**	-2.25937	**0.02386**
158217	66	9	3.67	3.70550	1.60688	2.30603	**0.02399**	0.84191	2.00813	0.41925	0.68963	1.87852	0.06031	2.25727	**0.02399**
189793	71	4	1.25	3.90422	2.37044	1.64704	0.10391	11.34500	2.14122	5.29837	0.11876	2.25299	**0.02426**	2.25299	**0.02426**
3926480	22	53	23.74	2.61708	1.13732	2.30109	**0.02428**	-0.37098	0.31678	-1.17109	0.24711	0.77443	0.43868	2.25260	**0.02428**
199190	69	6	2.00	3.41440	1.94475	1.75570	0.08339	3.59704	1.84765	1.94682	0.14673	2.25037	**0.02443**	2.25037	**0.02443**
4425571	30	45	21.82	2.44928	1.06638	2.29681	**0.02454**	-0.08789	0.34188	-0.25707	0.79838	1.40934	0.15873	2.24855	**0.02454**
275237	42	33	229.64	2.39206	1.04159	2.29655	**0.02456**	-0.09576	0.24420	-0.39213	0.69774	1.31517	0.18845	2.24831	**0.02456**
329313	70	5	1.20	0.64305	2.18224	0.29468	0.76909	-4.88817	0.78182	-6.25227	**0.02464**	-1.38129	0.16719	-2.24700	**0.02464**
198183	16	59	11.10	2.95129	1.28689	2.29335	**0.02475**	-0.26743	0.37831	-0.70691	0.48255	1.09113	0.27522	2.24529	**0.02475**
363646	58	17	13.29	2.83368	1.23684	2.29107	**0.02489**	-0.03179	0.55117	-0.05769	0.95481	1.54606	0.12209	2.24313	**0.02489**
174638	66	9	18.22	3.65503	1.59636	2.28961	**0.02498**	-0.19769	0.69955	-0.28260	0.78698	1.39407	0.16330	2.24175	**0.02498**
3887769	55	20	1.95	1.25025	1.21369	1.03012	0.30640	2.11529	0.90633	2.33390	**0.03213**	2.23840	**0.02520**	2.23840	**0.02520**
1000547	51	24	2.75	2.09717	1.12130	1.87029	0.06551	1.50036	1.09645	1.36837	0.18566	2.23821	**0.02521**	2.23821	**0.02521**
3251419	63	12	4.67	-3.22263	1.41065	-2.28450	**0.02529**	-0.61673	1.33946	-0.46043	0.65613	-1.89659	0.05788	-2.23692	**0.02529**
175373	70	5	1.60	-4.83026	2.11722	-2.28142	**0.02548**	1.03933	1.36105	0.76362	0.52488	-1.13007	0.25845	-2.23400	**0.02548**
4446669	60	15	12.40	-2.09376	1.31798	-1.58861	0.11653	-1.07164	0.62558	-1.71302	0.11240	-2.23233	**0.02559**	-2.23233	**0.02559**
4463108	70	5	4.80	0.52223	2.15575	0.24225	0.80928	-2.77526	0.45415	-6.11090	**0.02575**	-1.40617	0.15968	-2.22998	**0.02575**
4396297	33	42	7.98	2.37548	1.04473	2.27377	**0.02596**	-0.96391	0.47032	-2.04949	**0.04718**	0.17119	0.86407	2.22676	**0.02596**
688923	63	12	3.08	0.22256	1.48564	0.14981	0.88133	4.23127	1.59176	2.65823	**0.02612**	1.67844	0.09326	2.22440	**0.02612**
192437	26	49	16.29	2.46691	1.08727	2.26890	**0.02627**	0.27343	0.44793	0.61042	0.54459	1.99974	**0.04553**	2.22216	**0.02627**
197539	69	6	4.50	-1.49488	1.96640	-0.76021	0.44961	1.35457	0.33397	4.05598	**0.02701**	1.02910	0.30343	2.21143	**0.02701**
193763	63	12	3.17	0.88719	1.48485	0.59750	0.55205	3.49114	1.32360	2.63762	**0.02702**	1.98409	**0.04725**	2.21124	**0.02702**
816702	48	27	33.30	2.43313	1.07862	2.25578	**0.02712**	-0.03957	0.39577	-0.09997	0.92120	1.49257	0.13555	2.20974	**0.02712**
193873	65	10	1.10	2.85822	1.55592	1.83700	0.07034	7.95997	5.47236	1.45458	0.18911	2.20826	**0.02723**	2.20826	**0.02723**
184990	66	9	4.89	-1.68044	1.64473	-1.02171	0.31034	-1.10093	0.40593	-2.71210	**0.03501**	-2.20814	**0.02723**	-2.20814	**0.02723**
185814	24	51	7.24	2.51825	1.11956	2.24932	**0.02755**	-0.53974	0.40819	-1.32229	0.19234	0.63634	0.52455	2.20362	**0.02755**
3475269	67	8	2.38	-1.80463	1.72210	-1.04793	0.29818	-2.89091	1.03444	-2.79467	**0.03823**	-2.20101	**0.02774**	-2.20101	**0.02774**
308322	69	6	4.00	0.72569	1.97573	0.36730	0.71447	-2.50120	0.62343	-4.01203	**0.02779**	-1.29711	0.19459	-2.20025	**0.02779**
368490	59	16	12.25	1.10087	1.30605	0.84290	0.40208	1.11135	0.44876	2.47646	**0.02779**	2.14825	**0.03169**	2.20017	**0.02779**
198754	70	5	2.80	3.96227	2.09578	1.89060	0.06270	2.37909	1.31360	1.81112	0.21182	2.19901	**0.02788**	2.19901	**0.02788**
3946926	69	6	3.17	4.32411	1.92719	2.24374	**0.02792**	-0.21009	2.34592	-0.08956	0.93428	1.49616	0.13461	2.19834	**0.02792**
234443	67	8	6.88	2.12644	1.73491	1.22568	0.22431	2.16106	0.88425	2.44396	0.05837	2.19773	**0.02797**	2.19773	**0.02797**
191148	71	4	2.50	4.26313	2.33001	1.82966	0.07144	6.27986	1.95612	3.21038	0.19224	2.19674	**0.02804**	2.19674	**0.02804**
2530636	61	14	176.79	0.30809	1.37636	0.22384	0.82352	0.75212	0.29747	2.52834	**0.02806**	1.71086	0.08711	2.19650	**0.02806**
176269	9	66	145.11	0.43719	1.64749	0.26537	0.79148	0.43873	0.19524	2.24717	**0.02814**	1.73927	0.08199	2.19532	**0.02814**
363389	65	10	304.50	3.41274	1.52613	2.23620	**0.02844**	0.03187	0.32816	0.09712	0.92535	1.61566	0.10617	2.19119	**0.02844**
4433737	62	13	7.54	0.71895	1.42096	0.50596	0.61443	-1.24564	0.48863	-2.54922	**0.02890**	-1.18869	0.23456	-2.18481	**0.02890**
1835779	59	16	2.38	0.79107	1.30860	0.60451	0.54740	1.86683	0.76037	2.45517	**0.02893**	1.97006	**0.04883**	2.18443	**0.02893**
182653	70	5	8.80	-4.66877	2.09579	-2.22769	**0.02903**	0.20035	0.42913	0.46687	0.68651	-1.25832	0.20828	-2.18312	**0.02903**
164259	66	9	3.33	3.55504	1.60008	2.22179	**0.02944**	-0.40275	2.07998	-0.19363	0.85285	1.40859	0.15896	2.17753	**0.02944**
2831841	54	21	3.67	1.03230	1.18882	0.86835	0.38809	1.31375	0.55603	2.36274	**0.02960**	2.14853	**0.03167**	2.17539	**0.02960**
176108	64	11	2.18	0.39567	1.52815	0.25892	0.79643	-5.30171	2.00659	-2.64215	**0.02961**	-1.35570	0.17520	-2.17521	**0.02961**
361702	71	4	2.00	-4.84593	2.33321	-2.07694	**0.04138**	-5.12441	2.63994	-1.94111	0.30285	-2.17088	**0.02994**	-2.17088	**0.02994**
199677	54	21	2.90	-0.22591	1.19633	-0.18883	0.85075	-1.69433	0.72054	-2.35148	**0.03029**	-1.66485	0.09594	-2.16629	**0.03029**
178183	71	4	1.25	-1.25179	2.38028	-0.52590	0.60057	15.34949	0.74591	20.57833	**0.03091**	1.15586	0.24774	2.15820	**0.03091**
4094866	57	18	5.89	-2.67099	1.21434	-2.19954	**0.03105**	-0.62953	0.71924	-0.87527	0.39523	-2.12598	**0.03350**	-2.15642	**0.03105**
588471	64	11	315.64	-0.55796	1.51512	-0.36826	0.71376	-0.65723	0.25172	-2.61096	**0.03108**	-1.78390	0.07444	-2.15600	**0.03108**
4421070	52	23	2.96	2.26007	1.15937	1.94940	0.05514	1.41776	1.21794	1.16407	0.25810	2.15572	**0.03111**	2.15572	**0.03111**
4305372	70	5	27.60	-1.21748	2.14394	-0.56787	0.57189	-0.78765	0.14285	-5.51383	**0.03135**	-1.92180	0.05463	-2.15256	**0.03135**
4278525	48	27	3.67	-2.26326	1.08473	-2.08648	**0.04048**	-0.59482	0.58923	-1.00949	0.32281	-2.14787	**0.03172**	-2.14787	**0.03172**
193778	66	9	3.00	1.88940	1.63297	1.15703	0.25108	3.38041	1.45818	2.31824	0.05959	2.14361	**0.03206**	2.14361	**0.03206**
258099	64	11	4.64	3.21945	1.47293	2.18575	**0.03209**	-1.67410	1.72358	-0.97129	0.35985	0.86810	0.38534	2.14332	**0.03209**
2331530	71	4	1.50	-1.01006	2.38413	-0.42366	0.67308	-0.81432	0.04125	-19.74055	**0.03222**	-1.81272	0.06987	-2.14165	**0.03222**
194129	71	4	2.00	-0.45280	2.39683	-0.18892	0.85069	5.46473	0.27753	19.69075	**0.03230**	1.38056	0.16742	2.14064	**0.03230**
4452400	71	4	2.50	-5.04780	2.31312	-2.18225	**0.03235**	-2.03554	9.30343	-0.21879	0.86287	-1.63534	0.10198	-2.14000	**0.03235**
573110	67	8	2.88	-2.25023	1.71461	-1.31238	0.19356	-1.78425	0.83198	-2.14457	0.08483	-2.13792	**0.03252**	-2.13792	**0.03252**
4315974	68	7	2.71	3.01408	1.80834	1.66677	0.09991	-4.69667	1.46471	-3.20655	**0.03270**	-0.34682	0.72872	-2.13580	**0.03270**
197540	69	6	4.33	-4.18345	1.92522	-2.17297	**0.03307**	-0.97879	2.09938	-0.46623	0.67282	-1.80557	0.07099	-2.13118	**0.03307**
4329572	69	6	3.00	4.15493	1.91259	2.17240	**0.03312**	-2.09920	1.80300	-1.16428	0.32848	0.81563	0.41471	2.13065	**0.03312**
4412540	23	52	179.63	2.12686	1.13670	1.87108	0.06540	-0.50253	0.22955	-2.18917	**0.03338**	-0.20151	0.84030	-2.12751	**0.03338**
184342	68	7	1.86	3.59109	1.80301	1.99172	0.05020	-4.53746	1.42970	-3.17372	**0.03374**	-0.11661	0.90717	-2.12320	**0.03374**
209475	70	5	2.40	4.50800	2.08268	2.16452	**0.03374**	-1.65411	3.95890	-0.41782	0.71666	1.24467	0.21325	2.12315	**0.03374**
182255	42	33	4.58	1.52074	1.08288	1.40435	0.16451	-1.52637	0.68726	-2.22094	**0.03405**	-0.51579	0.60600	-2.11948	**0.03405**
180999	35	40	7.23	2.24833	1.04256	2.15655	**0.03438**	0.04691	0.58245	0.08053	0.93625	1.55250	0.12054	2.11558	**0.03438**
1046624	71	4	34.25	-0.53296	2.39660	-0.22238	0.82465	-1.08484	0.05882	-18.44235	**0.03449**	-1.65174	0.09859	-2.11435	**0.03449**
168071	30	45	10.31	2.29478	1.06653	2.15162	**0.03478**	-0.03537	0.47409	-0.07461	0.94088	1.44018	0.14982	2.11089	**0.03478**
4390319	49	26	4.85	-1.15249	1.11730	-1.03149	0.30576	-1.34852	0.65304	-2.06499	0.05038	-2.10783	**0.03505**	-2.10783	**0.03505**
178708	50	25	2.28	2.37874	1.10968	2.14362	**0.03544**	-0.62700	1.21803	-0.51477	0.61185	1.12843	0.25914	2.10328	**0.03544**

Table S4a. Associated taxonomies with Body mass index (BMI) at FDR < 0.05 level [a] (Cont.)

	Summary of OTUs reads			Binary model				Quantitative model				Meta analysis		Final association	
OTU	No. Absent	No. Present	mean counts in presents	Estimate	s.e.	*t* value	*P* value	Estimate	s.e.	*t* value	*P* value	Meta z value	*Meta P* value	*z*	*P value*
184925	44	31	20.10	2.26155	1.05596	2.14171	**0.03560**	0.34729	0.49075	0.70768	0.48500	1.97972	**0.04773**	2.10146	**0.03560**
110192	60	15	20.60	-2.78505	1.30157	-2.13976	**0.03576**	0.42000	0.48975	0.85758	0.40792	-0.89948	0.36840	-2.09960	**0.03576**
308544	62	13	3.15	-2.46808	1.39302	-1.77175	0.08067	-1.07461	0.82531	-1.30206	0.22209	-2.09857	**0.03585**	-2.09857	**0.03585**
1726408	70	5	40.80	-3.88068	2.10091	-1.84714	0.06884	-1.79682	1.12478	-1.59749	0.25125	-2.09786	**0.03592**	-2.09786	**0.03592**
577170	14	61	25.00	-0.41519	1.38814	-0.29910	0.76573	-0.68982	0.32135	-2.14666	**0.03601**	-1.69333	0.09039	-2.09676	**0.03601**
330469	49	26	7.15	1.46921	1.11305	1.31998	0.19102	-1.06352	0.47785	-2.22562	**0.03613**	-0.55716	0.57742	-2.09551	**0.03613**
4399109	53	22	48.32	2.43712	1.14303	2.13216	**0.03641**	-0.31419	0.51856	-0.60590	0.55175	1.05870	0.28973	2.09237	**0.03641**
179826	60	15	2.20	-2.81273	1.32039	-2.13023	**0.03657**	-1.39380	1.58240	-0.88081	0.39572	-2.07877	**0.03764**	-2.09053	**0.03657**
218710	67	8	3.75	-2.70719	1.71509	-1.57845	0.11884	-2.70149	1.65583	-1.63151	0.16371	-2.08762	**0.03683**	-2.08762	**0.03683**
158415	64	11	104.45	2.82740	1.50078	1.88395	0.06361	0.28307	0.24261	1.16679	0.27690	2.08047	**0.03748**	2.08047	**0.03748**
1820776	64	11	40.09	3.11897	1.47180	2.11916	**0.03753**	-0.61859	0.57961	-1.06726	0.31700	0.76321	0.44534	2.07999	**0.03753**
122049	54	21	13.14	1.88374	1.17350	1.60523	0.11282	0.67765	0.48177	1.40659	0.17658	2.07677	**0.03782**	2.07677	**0.03782**
842594	70	5	2.60	-2.86921	2.14027	-1.34058	0.18427	-3.23578	1.16522	-2.77696	0.10890	-2.07244	**0.03822**	-2.07244	**0.03822**
178151	71	4	3.25	-4.07035	2.33554	-1.74279	0.08564	-1.17728	0.43574	-2.70182	0.22567	-2.07213	**0.03825**	-2.07213	**0.03825**
336559	19	56	226.68	2.52453	1.19640	2.11010	**0.03833**	0.06934	0.20970	0.33067	0.74220	1.69727	0.08965	2.07136	**0.03833**
289452	68	7	1.00	-3.77236	1.78818	-2.10961	**0.03837**	0.00000	0.00000	0.00000	1.00000	-1.46434	0.14310	-2.07089	**0.03837**
334336	45	30	6.80	1.18768	1.08547	1.09416	0.27753	1.06640	0.55554	1.91958	0.06554	2.07003	**0.03845**	2.07003	**0.03845**
4424598	51	24	3.33	2.35244	1.11805	2.10405	**0.03887**	-1.73924	1.06467	-1.63359	0.11725	0.35297	0.72411	2.06559	**0.03887**
184114	25	50	16.10	2.34300	1.11674	2.09808	**0.03941**	-0.19876	0.38336	-0.51848	0.60655	1.09241	0.27465	2.05990	**0.03941**
190490	65	10	5.00	-1.62254	1.56411	-1.03735	0.30304	-1.17713	0.52581	-2.23869	0.06019	-2.05719	**0.03967**	-2.05719	**0.03967**
195805	67	8	11.75	-2.28720	1.71636	-1.33258	0.18687	-1.13928	0.59315	-1.92073	0.11282	-2.05452	**0.03993**	-2.05452	**0.03993**
113542	45	30	17.70	-1.30428	1.08400	-1.20321	0.23284	-0.65992	0.37170	-1.77543	0.08710	-2.05342	**0.04003**	-2.05342	**0.04003**
185583	22	53	26.92	2.44084	1.16824	2.08933	**0.04021**	0.00494	0.33680	0.01468	0.98835	1.46100	0.14402	2.05156	**0.04021**
2341726	69	6	2.00	4.04615	1.93934	2.08636	**0.04049**	2.29635	4.02793	0.57011	0.60850	1.81085	0.07016	2.04873	**0.04049**
179695	71	4	2.50	-2.60565	2.36955	-1.09964	0.27515	1.54608	0.09866	15.67005	**0.04057**	0.67642	0.49877	2.04788	**0.04057**
4364405	41	34	10.06	-0.72159	1.09568	-0.65857	0.51227	-1.04422	0.48876	-2.13647	**0.04064**	-1.91093	0.05601	-2.04716	**0.04064**
4434579	62	13	83.77	2.86908	1.37671	2.08401	**0.04071**	0.39284	0.47891	0.82027	0.43118	2.00370	**0.04510**	2.04648	**0.04071**
4346374	70	5	2.40	4.37709	2.10059	2.08374	**0.04073**	-9.62428	4.72320	-2.03766	0.17847	0.49551	0.62024	2.04623	**0.04073**
216710	28	47	7.55	0.26297	1.12599	0.23355	0.81600	1.31078	0.62517	2.09667	**0.04181**	1.60382	0.10875	2.03545	**0.04181**
175612	70	5	65.20	2.38008	2.21210	1.07594	0.28555	-1.76498	0.37282	-4.73417	**0.04184**	-0.68390	0.49404	-2.03513	**0.04184**
2298935	20	55	31.00	2.43613	1.17654	2.07058	**0.04198**	0.11246	0.31250	0.35986	0.72041	1.69111	0.09082	2.03368	**0.04198**
1945397	66	9	9.00	-3.31040	1.60219	-2.06617	**0.04241**	-0.17723	1.70936	-0.10368	0.92080	-1.50535	0.13223	-2.02947	**0.04241**
4333652	67	8	2.13	0.69519	1.73325	0.40109	0.68954	-2.65548	0.98279	-2.70199	**0.04268**	-1.15069	0.24986	-2.02680	**0.04268**
4457064	59	16	11.19	-2.73032	1.32370	-2.06264	**0.04276**	0.29097	0.94391	0.30826	0.76277	-1.21922	0.22276	-2.02609	**0.04276**
3648884	62	13	5.62	-2.82765	1.37547	-2.05577	**0.04343**	-0.51233	1.25193	-0.40923	0.69100	-1.70910	0.08743	-2.01953	**0.04343**
4460786	61	14	5.00	-1.10821	1.37029	-0.80874	0.42133	-1.49831	0.65685	-2.28105	**0.04346**	-1.99646	**0.04588**	-2.01930	**0.04346**
189007	67	8	2.00	-0.98619	1.73488	-0.56845	0.57150	-7.39027	2.75546	-2.68205	**0.04371**	-1.82623	0.06782	-2.01683	**0.04371**
4445673	49	26	34.08	2.24509	1.09393	2.05232	**0.04377**	0.04805	0.31897	0.15065	0.88157	1.53104	0.12576	2.01624	**0.04377**
1736067	67	8	3.38	3.52071	1.71649	2.05111	**0.04390**	-0.56620	1.98127	-0.28578	0.78651	1.23337	0.21744	2.01509	**0.04390**
181466	69	6	3.17	-1.91269	1.96122	-0.97525	0.33270	-4.42934	1.50382	-2.94539	0.06025	-2.01360	**0.04405**	-2.01360	**0.04405**
4460021	54	21	8.24	-0.49680	1.19418	-0.41602	0.67863	-1.84935	0.85438	-2.16455	**0.04411**	-1.71642	0.08609	-2.01306	**0.04411**
193129	50	25	5.52	0.62969	1.14732	0.54884	0.58481	-1.27098	0.59662	-2.13031	**0.04457**	-1.03400	0.30114	-2.00866	**0.04457**
189208	55	20	3.80	0.53183	1.22099	0.43557	0.66445	-2.07842	0.95904	-2.16718	**0.04471**	-1.11268	0.26585	-2.00734	**0.04471**
1146771	69	6	2.83	-2.99570	1.94728	-1.53841	0.12833	-4.52146	2.66476	-1.69676	0.18831	-2.00557	**0.04490**	-2.00557	**0.04490**
354334	66	9	2.89	0.89699	1.64555	0.54510	0.58737	-6.66461	2.63811	-2.52628	**0.04490**	-1.03442	0.30094	-2.00555	**0.04490**
185575	14	61	187.34	2.82522	1.38650	2.03767	**0.04526**	0.03489	0.26164	0.13337	0.89436	1.50969	0.13112	2.00224	**0.04526**
583134	71	4	2.50	-4.73459	2.32651	-2.03506	**0.04553**	0.75144	1.36740	0.54954	0.68010	-1.12248	0.26166	-1.99975	**0.04553**
114348	67	8	7.25	1.72984	1.72293	1.00401	0.31874	4.89145	2.10018	2.32906	0.06729	1.99884	**0.04563**	1.99884	**0.04563**
2774254	69	6	13.50	-3.90064	1.91989	-2.03170	**0.04588**	-0.77310	0.87221	-0.88637	0.44070	-1.95695	0.05035	-1.99654	**0.04588**
845396	69	6	4.00	3.54972	1.92945	1.83976	0.06993	3.20869	2.64739	1.21202	0.31225	1.99609	**0.04592**	1.99609	**0.04592**
198248	56	19	23.58	0.75399	1.23875	0.60867	0.54466	-1.08786	0.50268	-2.16411	**0.04593**	-0.98308	0.32557	-1.99607	**0.04593**
180826	45	30	4.87	2.16642	1.06713	2.03014	**0.04604**	0.38402	0.72907	0.52673	0.60268	1.77880	0.07527	1.99505	**0.04604**
4434334	51	24	9.75	2.26822	1.11790	2.02899	**0.04616**	-0.12231	0.69743	-0.17537	0.86247	1.28744	0.19794	1.99395	**0.04616**
3211875	25	50	7.50	-0.22260	1.13740	-0.19571	0.84539	-1.06646	0.52118	-2.04622	**0.04635**	-1.54657	0.12197	-1.99218	**0.04635**
25461	70	5	1.60	-1.34377	2.14117	-0.62758	0.53226	15.08178	3.37368	4.47042	**0.04657**	0.96564	0.33422	1.99018	**0.04657**
4472721	4	71	28.08	-0.06419	2.43931	-0.02632	0.97908	-0.71691	0.35412	-2.02450	**0.04685**	-1.42404	0.15444	-1.98767	**0.04685**
184534	66	9	14.67	-2.23082	1.63543	-1.36405	0.17680	-1.14200	0.68155	-1.67561	0.14483	-1.98605	**0.04703**	-1.98605	**0.04703**
125270	61	14	3.50	-2.71407	1.34532	-2.01742	**0.04738**	-0.33335	1.00853	-0.33053	0.74721	-1.63003	0.10310	-1.98289	**0.04738**
2932557	61	14	2.79	2.40449	1.42134	1.69171	0.09503	-2.14154	0.96296	-2.22392	**0.04803**	-0.21751	0.82781	-1.97706	**0.04803**
2119418	32	43	269.07	1.37740	1.07161	1.28535	0.20279	0.37847	0.24449	1.54802	0.12949	1.97265	**0.04854**	1.97265	**0.04854**
1139021	55	20	43.05	1.37799	1.20764	1.14106	0.25763	-0.95896	0.45177	-2.12265	**0.04877**	-0.59296	0.55320	-1.97060	**0.04877**
158625	70	5	2.80	-4.19743	2.09648	-2.00213	**0.04904**	0.59973	2.34912	0.25530	0.82235	-1.23300	0.21757	-1.96826	**0.04904**
519880	70	5	3.80	2.48061	2.15851	1.14922	0.25427	-27.04683	6.23030	-4.34118	**0.04918**	-0.58476	0.55871	-1.96702	**0.04918**
4436552	18	57	852.02	2.44127	1.22104	1.99934	**0.04935**	0.05112	0.13330	0.38351	0.70285	1.65963	0.09699	1.96559	**0.04935**
4379889	55	20	4.70	1.58269	1.20524	1.31318	0.19329	-1.56247	0.73936	-2.11329	**0.04966**	-0.46806	0.63974	-1.96284	**0.04966**
199491	69	6	4.00	2.28134	1.96908	1.15858	0.25045	2.97205	1.28844	2.30670	0.10435	1.96108	**0.04987**	1.96108	**0.04987**
592616	60	15	2.67	2.61590	1.31187	1.99402	**0.04994**	-2.77350	1.30007	-2.13335	0.05423	0.02511	0.97997	1.96050	**0.04994**
167373	4	71	50.25	3.52286	2.35587	1.49535	0.13919	0.42360	0.32433	1.30606	0.19593	1.96012	**0.04998**	1.96012	**0.04998**

[a] Statistics associated to the two part model for association analysis between the trait and OTUs adjusting for age and gender. The binomial analysis that tests for association of detecting a microbe represented by an OTU with the trait, where s.e. represents the standard error of the estimate, *t*-value of a *t* test for the estimate and its associated probability (*P* value). The quantitative analysis tests for association between the trait and the abundance of the OTU for the subjects where that OTU is present, where s.e. represents the standard error of the estimate, *t*-value of a *t* test for the estimate and its associated probability (*P* value). The Meta analysis combine the effect of both binary and quantitative analysis, a meta *P* value was derived using an unweighted Z method. The final association *P* value per OTU-trait pair was assigned from the minimum of *P* values from the binary analysis, quantitative analysis, and meta-analysis.

Table S4b. OTUs associated with Triglycerides at FDR < 0.05 level [a]

	Summary of OTUs reads			Binary model				Quantitative model				Meta analysis		Final association	
OTU	No. Absent	No. Present	mean counts in presents	Estimate	s.e.	*t* value	*P* value	Estimate	s.e.	*t* value	*P* value	Meta z value	*Meta P* value	*z*	*P value*
195123	59	16	3.75	-2.21729	24.85185	-0.08922	0.92915	48.95492	8.02948	6.09690	**0.00004**	2.84996	**0.00437**	4.11935	**0.00004**
2388088	58	17	3.82	95.10042	21.79403	4.36360	**0.00004**	39.05425	31.13143	1.25450	0.23020	3.74494	**0.00018**	4.09631	**0.00004**
178151	71	4	3.25	172.47685	40.31464	4.27827	**0.00006**	-248.98487	0.60620	-410.73177	**0.00155**	0.60777	0.54334	4.02468	**0.00006**
4442508	71	4	6.50	171.59973	40.95988	4.18946	**0.00008**	157.78946	81.41094	1.93818	0.30324	3.52082	**0.00043**	3.94968	**0.00008**
3327894	3	72	136.31	-193.62657	46.67167	-4.14870	**0.00009**	-1.56333	3.09463	-0.50518	0.61505	-3.12399	**0.00178**	-3.91510	**0.00009**
4437746	70	5	10.80	151.71499	36.61591	4.14342	**0.00009**	19.86678	73.27994	0.27111	0.81173	2.93366	**0.00335**	3.91062	**0.00009**
163494	59	16	3.44	19.66282	24.77447	0.79367	0.42999	128.77073	24.77472	5.19767	**0.00017**	3.21483	**0.00131**	3.75726	**0.00017**
177100	71	4	1.25	131.18782	42.91750	3.05674	**0.00314**	506.00000	16.58312	30.51295	**0.02086**	3.72249	**0.00020**	3.72249	**0.00020**
175729	54	21	4.29	-44.07819	22.18797	-1.98658	0.05078	-32.84108	8.43923	-3.89148	**0.00107**	-3.69459	**0.00022**	-3.69459	**0.00022**
174439	61	14	3.29	-54.76893	25.21380	-2.17218	**0.03314**	-27.31647	6.88011	-3.97036	**0.00219**	-3.67198	**0.00024**	-3.67198	**0.00024**
182653	70	5	8.80	142.58930	37.43193	3.80930	**0.00029**	1.45473	93.77599	0.01551	0.98903	2.57188	**0.01011**	3.62344	**0.00029**
318970	67	8	1.63	26.44487	34.24850	0.77215	0.44255	203.03638	23.93529	8.48272	**0.00037**	3.05864	**0.00222**	3.55768	**0.00037**
583134	71	4	2.50	136.03998	42.37249	3.21057	**0.00198**	261.23319	25.75264	10.14394	0.06256	3.50406	**0.00046**	3.50406	**0.00046**
4421273	40	35	9.49	-44.42512	19.87763	-2.23493	**0.02853**	-15.02770	5.07366	-2.96190	**0.00572**	-3.50243	**0.00046**	-3.50243	**0.00046**
3302039	68	7	2.29	102.11586	33.20857	3.07498	**0.00297**	175.24412	64.28930	2.72587	0.05267	3.47051	**0.00052**	3.47051	**0.00052**
187178	51	24	56.92	25.94119	21.60963	1.20045	0.23390	33.32894	8.43313	3.95214	**0.00073**	3.23079	**0.00123**	3.37866	**0.00073**
130763	70	5	9.20	133.13555	37.76472	3.52539	**0.00074**	73.01093	95.72293	0.76273	0.52531	2.83523	**0.00458**	3.37444	**0.00074**
519763	70	5	11.40	129.62156	37.96771	3.41400	**0.00105**	105.28224	75.14789	1.40100	0.29622	3.05477	**0.00225**	3.27552	**0.00105**
4020502	17	58	96.33	-78.91159	23.14056	-3.41010	**0.00107**	0.41016	3.01656	0.13597	0.89234	-2.21798	**0.02656**	-3.27205	**0.00107**
4379646	67	8	1.75	103.46286	30.53319	3.38854	**0.00114**	128.10938	312.41797	0.41006	0.69874	2.57375	**0.01006**	3.25282	**0.00114**
571642	40	35	5.00	-65.66006	19.57723	-3.35390	**0.00127**	-5.44005	7.57639	-0.71803	0.47795	-2.77997	**0.00544**	-3.22188	**0.00127**
170462	68	7	4.00	109.33597	32.83151	3.33021	**0.00137**	24.93031	105.12404	0.23715	0.82419	2.42031	**0.01551**	3.20068	**0.00137**
4444262	65	10	13.70	92.70458	27.91868	3.32052	**0.00141**	-20.86735	25.49646	-0.81844	0.44007	1.71115	0.08705	3.19200	**0.00141**
4378683	52	23	15.17	67.83950	20.54066	3.30269	**0.00149**	0.75949	16.67221	0.04555	0.96412	2.27759	**0.02275**	3.17601	**0.00149**
4469032	70	5	5.80	124.62234	37.97335	3.28184	**0.00159**	-59.90431	113.98038	-0.52557	0.65165	1.91330	0.05571	3.15730	**0.00159**
194089	68	7	2.14	95.52322	33.55399	2.84685	**0.00575**	248.08042	111.32062	2.22852	0.08976	3.15259	**0.00162**	3.15259	**0.00162**
309391	71	4	3.00	137.43994	42.12865	3.26239	**0.00169**	210.19327	157.74733	1.33247	0.40986	2.80294	**0.00506**	3.13982	**0.00169**
1835985	68	7	4.71	60.51589	34.16059	1.77151	0.08071	217.96757	43.58857	5.00057	**0.00749**	3.12608	**0.00177**	3.12608	**0.00177**
151870	70	5	4.80	125.13938	38.57312	3.24421	**0.00179**	-18.51976	99.83160	-0.18551	0.86994	2.09285	**0.03636**	3.12347	**0.00179**
4428929	69	6	1.83	113.13588	34.92739	3.23917	**0.00182**	-67.74654	141.44090	-0.47897	0.66470	1.89894	0.05757	3.11893	**0.00182**
191043	70	5	4.20	122.23330	38.16084	3.20311	**0.00203**	36.30152	169.23000	0.21451	0.85003	2.31613	**0.02055**	3.08643	**0.00203**
326662	70	5	1.40	121.82848	38.09906	3.19768	**0.00206**	-132.36471	222.86241	-0.59393	0.61279	1.82110	0.06859	3.08153	**0.00206**
194053	71	4	1.50	136.71756	43.00021	3.17946	**0.00218**	-381.49057	268.41610	-1.42127	0.39034	1.55993	0.11878	3.06508	**0.00218**
362955	66	9	16.44	-4.71435	31.37827	-0.15024	0.88099	41.96079	8.22838	5.09952	**0.00222**	2.05702	**0.03968**	3.05877	**0.00222**
186352	29	46	8.22	-62.79353	19.85743	-3.16222	**0.00229**	-3.54220	6.64189	-0.53331	0.59656	-2.53062	**0.01139**	-3.04949	**0.00229**
166689	69	6	4.00	110.94030	35.10651	3.16011	**0.00231**	-3.79122	117.39124	-0.03230	0.97626	2.13393	**0.03285**	3.04758	**0.00231**
191361	21	54	17.31	-66.88559	21.18813	-3.15675	**0.00233**	-2.84727	5.68569	-0.50078	0.61868	-2.50476	**0.01225**	-3.04454	**0.00233**
168439	44	31	33.58	33.56790	21.09612	1.59119	0.11595	25.12707	8.50586	2.95409	**0.00629**	3.04354	**0.00234**	3.04354	**0.00234**
183619	70	5	2.20	119.41571	38.42977	3.10738	**0.00270**	25.12318	131.64275	0.19084	0.86627	2.24027	**0.02507**	2.99982	**0.00270**
4343184	63	12	2.75	81.05727	26.17627	3.09659	**0.00279**	-53.77380	48.28355	-1.11371	0.29426	1.37265	0.16986	2.99003	**0.00279**
185802	31	44	91.45	-51.23176	19.71249	-2.59895	**0.01134**	-6.18315	3.56937	-1.73228	0.09074	-2.98658	**0.00282**	-2.98658	**0.00282**
179384	68	7	1.86	74.04184	33.77213	2.19239	**0.03158**	196.52116	67.13172	2.92740	**0.04293**	2.95151	**0.00316**	2.95151	**0.00316**
4331360	22	53	13.06	-0.47368	22.36086	-0.02118	0.98316	-26.35196	8.49753	-3.10113	**0.00317**	-2.10175	**0.03557**	-2.95122	**0.00317**
4391326	69	6	14.17	107.80488	35.36310	3.04851	**0.00322**	-30.98809	61.95396	-0.50018	0.65134	1.76382	0.07776	2.94633	**0.00322**
4468466	18	57	118.93	-41.07753	23.88759	-1.71962	0.08980	-8.62870	3.44402	-2.50542	**0.01528**	-2.91482	**0.00356**	-2.91482	**0.00356**
174571	64	11	2.82	72.60399	27.51598	2.63861	**0.01020**	87.88975	52.13012	1.68597	0.13029	2.88645	**0.00390**	2.88645	**0.00390**
230479	70	5	4.00	114.34613	38.40503	2.97737	**0.00396**	25.79741	144.49669	0.17853	0.87475	2.14895	**0.03164**	2.88145	**0.00396**
300374	43	32	6.13	-41.32386	20.28601	-2.03706	**0.04532**	-14.82788	6.82569	-2.17236	**0.03814**	-2.88143	**0.00396**	-2.88143	**0.00396**
169364	61	14	6.64	29.93819	25.80473	1.16018	0.24981	74.83746	20.62621	3.62827	**0.00397**	2.85067	**0.00436**	2.88064	**0.00397**
199524	61	14	1.29	43.37672	26.09974	1.66196	0.10087	186.45194	65.29559	2.85551	**0.01564**	2.86931	**0.00411**	2.86931	**0.00411**
4423553	37	38	12.08	-47.18898	19.60270	-2.40727	**0.01864**	-10.61398	6.07017	-1.74855	0.08914	-2.86568	**0.00416**	-2.86568	**0.00416**
176269	9	66	145.11	-87.11626	29.47532	-2.95557	**0.00422**	-1.38344	2.83137	-0.48861	0.62681	-2.36720	**0.01792**	-2.86151	**0.00422**
192462	20	55	44.05	-8.98919	22.91413	-0.39230	0.69600	17.92372	5.99021	2.99217	**0.00423**	1.74643	0.08074	2.86055	**0.00423**
4450415	70	5	2.60	100.83604	39.35611	2.56214	**0.01250**	198.67864	76.91674	2.58304	0.12286	2.85722	**0.00427**	2.85722	**0.00427**
168071	30	45	10.31	-58.05155	19.67982	-2.94980	**0.00429**	-1.37593	7.74837	-0.17758	0.85991	-2.14446	**0.03200**	-2.85624	**0.00429**
292041	71	4	1.50	13.80766	45.10377	0.30613	0.76039	-153.36851	1.04496	-146.77020	**0.00434**	-1.80139	0.07164	-2.85252	**0.00434**
2182669	70	5	6.80	7.27226	41.10219	0.17693	0.86006	-16.60887	1.10668	-15.00779	**0.00441**	-1.88862	0.05894	-2.84721	**0.00441**
185864	56	19	4.95	39.36651	23.08040	1.70563	0.09239	56.64785	21.84122	2.59362	**0.01959**	2.84044	**0.00451**	2.84044	**0.00451**
174288	49	26	3.69	-59.12423	20.26925	-2.91694	**0.00471**	8.63900	9.37280	0.92171	0.36625	-1.35950	0.17399	-2.82614	**0.00471**
185034	31	44	11.86	-35.19822	20.25176	-1.73803	0.08648	-15.02325	6.37969	-2.35485	**0.02340**	-2.81508	**0.00488**	-2.81508	**0.00488**
2157225	65	10	2.40	81.87876	28.23526	2.89988	**0.00495**	27.18942	78.15937	0.34787	0.73816	2.22369	**0.02617**	2.81049	**0.00495**
4483337	17	58	50.33	7.76230	24.43743	0.31764	0.75168	14.64031	5.01851	2.91726	**0.00510**	2.20392	**0.02753**	2.80038	**0.00510**
184036	68	7	1.57	38.20940	35.06843	1.08957	0.27954	245.58041	44.22114	5.55346	**0.00515**	2.74298	**0.00609**	2.79780	**0.00515**
181756	70	5	4.20	110.91458	38.52101	2.87933	**0.00524**	-113.72411	189.80861	-0.59915	0.60990	1.61320	0.10670	2.79162	**0.00524**
4453609	9	66	370.65	-84.83721	29.71645	-2.85489	**0.00562**	-0.91090	3.23240	-0.28180	0.77902	-2.15650	**0.03104**	-2.76916	**0.00562**
191110	69	6	1.00	101.05523	35.48670	2.84769	**0.00574**	0.00000	0.00000	0.00000	1.00000	1.95341	0.05077	2.76254	**0.00574**
195756	71	4	1.50	121.69993	42.79332	2.84390	**0.00580**	-89.79246	366.18606	-0.24521	0.84692	1.81443	0.06961	2.75905	**0.00580**
165928	69	6	4.83	2.78407	37.47675	0.07429	0.94099	-59.29387	8.46649	-7.00336	**0.00598**	-1.89148	0.05856	-2.74898	**0.00598**
701221	66	9	2.11	-27.85417	31.08167	-0.89616	0.37315	-58.28232	14.04988	-4.14824	**0.00602**	-2.57183	**0.01012**	-2.74653	**0.00602**
180090	69	6	1.00	99.98104	35.49136	2.81705	**0.00625**	0.00000	0.00000	0.00000	1.00000	1.93346	0.05318	2.73432	**0.00625**
183579	57	18	3.67	50.03141	23.22741	2.15398	**0.03459**	43.66729	23.34783	1.87029	0.08109	2.72770	**0.00638**	2.72770	**0.00638**
197708	68	7	3.29	93.03935	33.23527	2.79942	**0.00657**	77.71388	136.47246	0.56945	0.59951	2.29326	**0.02183**	2.71806	**0.00657**
3692427	69	6	4.83	102.06442	36.76204	2.77635	**0.00700**	9.28890	90.54426	0.10259	0.92476	1.97368	**0.04842**	2.69677	**0.00700**
265871	7	68	161.65	-83.30525	33.52179	-2.48511	**0.01528**	-4.96050	3.54269	-1.40021	0.16621	-2.69427	**0.00705**	-2.69427	**0.00705**
2575651	65	10	7.10	78.63575	28.37572	2.77123	**0.00710**	-48.29897	53.94330	-0.89537	0.40033	1.30886	0.19058	2.69204	**0.00710**
110060	70	5	2.00	107.79014	39.14467	2.75364	**0.00746**	-97.10638	314.88457	-0.30839	0.78694	1.70094	0.08895	2.67577	**0.00746**
213870	64	11	5.00	75.05376	27.27095	2.75215	**0.00749**	-0.24862	45.13530	-0.00551	0.99574	1.88731	0.05912	2.67440	**0.00749**
187210	69	6	1.83	97.78547	35.61370	2.74573	**0.00762**	-62.97015	157.75652	-0.39916	0.71649	1.63010	0.10308	2.66846	**0.00762**
2532173	29	46	9.89	-54.33160	19.83908	-2.73862	**0.00777**	-1.28905	5.39564	-0.23891	0.81231	-2.05013	**0.04035**	-2.66188	**0.00777**
197988	42	33	14.00	-53.17653	19.45338	-2.73354	**0.00788**	-4.97914	6.33993	-0.78536	0.43840	-2.42684	**0.01523**	-2.65718	**0.00788**

Table S4b. OTUs associated with Triglycerides at FDR < 0.05 level [a] (Cont.)

	Summary of OTUs reads			Binary model				Quantitative model				Meta analysis		Final association	
OTU	No. Absent	No. Present	mean counts in presents	Estimate	s.e.	*t* value	*P* value	Estimate	s.e.	*t* value	*P* value	Meta z value	*Meta P* value	*z*	*P value*
749837	61	14	2.71	67.39075	24.78876	2.71860	**0.00821**	61.09484	73.43342	0.83198	0.42312	2.43554	**0.01487**	2.64335	**0.00821**
190639	24	51	24.22	-47.30604	21.03556	-2.24886	**0.02758**	-7.42920	4.76555	-1.55894	0.12558	-2.64101	**0.00827**	-2.64101	**0.00827**
1010113	54	21	14.67	2.02378	22.58537	0.08961	0.92885	13.11867	4.43086	2.96075	**0.00837**	1.92764	0.05390	2.63680	**0.00837**
158950	69	6	5.83	92.56582	36.10961	2.56347	**0.01245**	81.23794	52.42039	1.54974	0.21898	2.63632	**0.00838**	2.63632	**0.00838**
4473788	66	9	2.11	80.38916	29.73540	2.70348	**0.00856**	-224.40974	86.06161	-2.60755	**0.04025**	0.40884	0.68266	2.62934	**0.00856**
132892	60	15	3.40	65.06886	24.21174	2.68749	**0.00894**	43.43479	50.67772	0.85708	0.40819	2.43357	**0.01495**	2.61450	**0.00894**
196982	58	17	2.88	-31.17575	24.04551	-1.29653	0.19893	40.77553	13.46519	3.02822	**0.00903**	0.93780	0.34835	2.61085	**0.00903**
4412540	23	52	179.63	-5.67105	22.03160	-0.25741	0.79760	-13.80607	5.08265	-2.71631	**0.00910**	-2.02580	**0.04279**	-2.60845	**0.00910**
336627	56	19	2.84	14.63388	23.29516	0.62819	0.53186	79.54714	26.82041	2.96592	**0.00910**	2.28628	**0.02224**	2.60813	**0.00910**
4450214	31	44	39.32	-27.13816	20.72865	-1.30921	0.19463	-18.00920	7.26243	-2.47977	**0.01735**	-2.59954	**0.00934**	-2.59954	**0.00934**
193534	68	7	2.43	88.73102	33.25662	2.66807	**0.00942**	26.57816	107.71742	0.24674	0.81726	1.99938	**0.04557**	2.59648	**0.00942**
188966	70	5	2.20	103.46094	38.79245	2.66704	**0.00944**	-246.62377	131.58052	-1.87432	0.20174	0.93260	0.35103	2.59552	**0.00944**
825808	43	32	39.38	-40.55821	19.96602	-2.03136	**0.04591**	-7.34414	4.24532	-1.72994	0.09427	-2.59473	**0.00947**	-2.59473	**0.00947**
4407515	58	17	5.06	61.82362	23.20214	2.66457	**0.00951**	16.04877	27.04859	0.59333	0.56242	2.24327	**0.02488**	2.59322	**0.00951**
782953	15	60	127.50	-64.10696	24.30549	-2.63755	**0.01023**	3.17170	3.00913	1.05403	0.29632	-1.07745	0.28128	-2.56811	**0.01023**
194488	71	4	2.50	-58.87229	45.04644	-1.30692	0.19540	-93.92308	2.88128	-32.59769	**0.01952**	-2.56691	**0.01026**	-2.56691	**0.01026**
180215	70	5	2.00	-16.75253	40.98337	-0.40876	0.68393	33.63396	3.45490	9.73514	**0.01039**	1.52420	0.12746	2.56266	**0.01039**
173863	31	44	5.07	0.12133	21.18528	0.00573	0.99545	-29.39359	10.94683	-2.68512	**0.01042**	-1.80735	0.07071	-2.56169	**0.01042**
179695	71	4	2.50	113.53189	43.22146	2.62675	**0.01053**	51.62255	309.10137	0.16701	0.89465	1.90246	0.05711	2.55806	**0.01053**
147969	35	40	69.65	-40.64780	19.98958	-2.03345	**0.04569**	-6.27839	3.79455	-1.65458	0.10647	-2.55441	**0.01064**	-2.55441	**0.01064**
190301	56	19	3.21	-58.46819	22.37850	-2.61270	**0.01093**	0.98536	9.07411	0.10859	0.91488	-1.72399	0.08471	-2.54498	**0.01093**
183147	70	5	1.60	101.73995	39.01166	2.60794	**0.01107**	247.54682	320.13402	0.77326	0.52025	2.25107	**0.02438**	2.54054	**0.01107**
190100	70	5	1.60	101.91415	39.09090	2.60711	**0.01109**	156.77801	190.05281	0.82492	0.49615	2.27712	**0.02278**	2.53977	**0.01109**
2407149	33	42	5.76	-51.15777	19.62291	-2.60704	**0.01109**	-2.30358	6.35314	-0.36259	0.71887	-2.05038	**0.04033**	-2.53971	**0.01109**
187751	45	30	57.10	26.28048	20.51239	1.28120	0.20423	11.23512	4.55952	2.46410	**0.02039**	2.53755	**0.01116**	2.53755	**0.01116**
194471	63	12	1.92	47.53048	27.19976	1.74746	0.08482	106.06336	50.00205	2.12118	0.06292	2.53367	**0.01129**	2.53367	**0.01129**
199286	40	35	9.26	-31.24569	20.12071	-1.55291	0.12483	-14.49630	6.82252	-2.12477	**0.04142**	-2.52725	**0.01150**	-2.52725	**0.01150**
3747551	68	7	1.57	87.79126	33.86141	2.59266	**0.01153**	-83.50580	122.59605	-0.68115	0.53317	1.34572	0.17839	2.52631	**0.01153**
206513	51	24	5.46	-31.68956	21.44267	-1.47787	0.14380	-18.59210	8.25479	-2.25228	**0.03513**	-2.52340	**0.01162**	-2.52340	**0.01162**
172233	63	12	2.00	48.06567	27.16067	1.76968	0.08101	112.31128	54.64587	2.05526	0.07002	2.51492	**0.01191**	2.51492	**0.01191**
4350477	57	18	5.28	59.39946	23.02421	2.57987	**0.01192**	-17.68972	26.12239	-0.67719	0.50860	1.31052	0.19002	2.51438	**0.01192**
4405104	39	36	18.58	-22.27857	20.13056	-1.10670	0.27211	-23.49909	9.06066	-2.59353	**0.01406**	-2.51311	**0.01197**	-2.51311	**0.01197**
325850	63	12	69.92	68.47034	26.64869	2.56937	**0.01226**	3.15025	20.56974	0.15315	0.88166	1.87627	0.06062	2.50458	**0.01226**
189937	59	16	2.38	45.52820	24.54553	1.85485	0.06771	75.73118	41.03841	1.84537	0.08788	2.49864	**0.01247**	2.49864	**0.01247**
365621	70	5	2.40	100.81869	39.55011	2.54914	**0.01293**	-602.90982	406.31388	-1.48385	0.27611	0.98754	0.32338	2.48569	**0.01293**
3236435	37	38	40.37	-49.74473	19.54899	-2.54462	**0.01308**	6.70704	5.33173	1.25795	0.21674	-0.88121	0.37820	-2.48147	**0.01308**
2565100	67	8	6.25	38.95856	32.90137	1.18410	0.24027	179.87547	53.39242	3.36893	**0.01991**	2.47650	**0.01327**	2.47650	**0.01327**
4326869	71	4	2.75	-51.57389	44.72479	-1.15314	0.25267	-264.64863	7.72278	-34.26855	**0.01857**	-2.47339	**0.01338**	-2.47339	**0.01338**
197499	33	42	8.17	-50.11530	19.77543	-2.53422	**0.01345**	-1.32462	6.47480	-0.20458	0.83896	-1.89149	0.05856	-2.47175	**0.01345**
3507744	68	7	4.57	-20.35545	34.80143	-0.58490	0.56044	59.06433	13.99864	4.21929	**0.01349**	1.33537	0.18175	2.47069	**0.01349**
178242	46	29	7.38	-50.73766	20.07670	-2.52719	**0.01369**	6.94856	6.90439	1.00640	0.32350	-1.04502	0.29601	-2.46518	**0.01369**
196371	69	6	3.33	91.58361	36.28301	2.52415	**0.01380**	51.63088	136.83958	0.37731	0.73105	1.98418	**0.04724**	2.46233	**0.01380**
310380	70	5	4.00	-34.23186	41.37962	-0.82726	0.41082	-241.29679	28.90696	-8.34736	**0.01405**	-2.31820	**0.02044**	-2.45599	**0.01405**
4405423	60	15	4.40	4.75524	25.77278	0.18451	0.85413	-51.18804	17.82959	-2.87096	**0.01406**	-1.60638	0.10819	-2.45561	**0.01406**
302049	58	17	3.94	48.30662	23.55919	2.05044	**0.04396**	46.90615	30.37866	1.54405	0.14488	2.45530	**0.01408**	2.45530	**0.01408**
195081	48	27	6.33	-50.82587	20.28079	-2.50611	**0.01447**	11.31309	7.16308	1.57936	0.12734	-0.65110	0.51498	-2.44545	**0.01447**
184465	58	17	1.94	34.91331	24.11439	1.44782	0.15201	82.67188	37.16405	2.22451	**0.04307**	2.44340	**0.01455**	2.44340	**0.01455**
2700883	18	57	11.93	24.68613	23.86645	1.03434	0.30444	16.66597	6.68503	2.49303	**0.01576**	2.43338	**0.01496**	2.43338	**0.01496**
189334	70	5	3.00	97.46562	39.13854	2.49027	**0.01507**	6.63829	223.16518	0.02975	0.97897	1.73735	0.08233	2.43062	**0.01507**
4449427	31	44	29.41	49.24041	19.79426	2.48761	**0.01518**	0.87771	8.31831	0.10551	0.91648	1.79110	0.07328	2.42813	**0.01518**
183681	43	32	5.41	-49.00989	19.71313	-2.48615	**0.01523**	0.24300	9.87756	0.02460	0.98054	-1.69873	0.08937	-2.42676	**0.01523**
2017729	51	24	3.92	-8.27528	22.25828	-0.37178	0.71115	68.63617	26.04498	2.63529	**0.01547**	1.45018	0.14701	2.42119	**0.01547**
191792	19	56	14.52	-55.41859	22.38435	-2.47577	**0.01565**	1.85411	5.15189	0.35989	0.72036	-1.45597	0.14540	-2.41703	**0.01565**
4457427	25	50	41.32	-51.16154	20.70227	-2.47130	**0.01583**	-1.66139	4.10426	-0.40480	0.68746	-1.99060	**0.04652**	-2.41284	**0.01583**
180421	62	13	3.85	36.64920	26.81960	1.36651	0.17603	109.53120	46.94106	2.33338	**0.04181**	2.39603	**0.01657**	2.39603	**0.01657**
4479397	70	5	1.60	56.79051	40.10896	1.41591	0.16111	62.48330	14.09396	4.43334	**0.04730**	2.39352	**0.01669**	2.39352	**0.01669**
184996	70	5	2.80	-48.81048	40.66515	-1.20030	0.23396	-39.03632	6.78003	-5.75754	**0.02887**	-2.38686	**0.01699**	-2.38686	**0.01699**
322835	38	37	7.84	-47.77160	19.55197	-2.44331	**0.01701**	-4.88765	6.97818	-0.70042	0.48843	-2.17745	**0.02945**	-2.38658	**0.01701**
4301511	33	42	11.90	-47.90802	19.66652	-2.43602	**0.01733**	-4.48482	6.89823	-0.65014	0.51941	-2.13827	**0.03249**	-2.37973	**0.01733**
323135	48	27	24.15	-47.42779	20.40843	-2.32393	**0.02296**	-7.18108	6.46540	-1.11069	0.27770	-2.37564	**0.01752**	-2.37564	**0.01752**
315846	67	8	20.50	-6.10638	33.58215	-0.18183	0.85622	16.45910	4.73879	3.47327	**0.01779**	1.54775	0.12168	2.37003	**0.01779**
349246	69	6	2.00	90.47741	37.33718	2.42325	**0.01790**	-88.28282	79.25003	-1.11398	0.34650	1.00857	0.31318	2.36773	**0.01790**
187038	69	6	1.50	-66.35625	36.56331	-1.81483	0.07371	-19.97471	9.25157	-2.15906	0.11968	-2.36491	**0.01803**	-2.36491	**0.01803**
4370941	45	30	11.83	-31.16896	20.93498	-1.48885	0.14090	-12.51587	6.40912	-1.95282	0.06128	-2.36453	**0.01805**	-2.36453	**0.01805**
307113	63	12	1.67	43.88454	27.20315	1.61322	0.11107	135.41651	69.71354	1.94247	0.08398	2.34863	**0.01884**	2.34863	**0.01884**
4045882	63	12	1.67	43.88454	27.20315	1.61322	0.11107	135.41651	69.71354	1.94247	0.08398	2.34863	**0.01884**	2.34863	**0.01884**
1820513	43	32	67.81	47.53789	19.80276	2.40057	**0.01896**	-9.49668	8.62952	-1.10049	0.28017	0.89553	0.37051	2.34640	**0.01896**
214036	39	36	11.44	46.95873	19.58069	2.39822	**0.01907**	-10.46376	11.18549	-0.93548	0.35634	1.00538	0.31471	2.34419	**0.01907**
4390839	68	7	1.29	-53.88049	34.42758	-1.56504	0.12196	77.17136	20.30473	3.80066	**0.01909**	0.56364	0.57300	2.34371	**0.01909**
208790	68	7	16.43	58.24674	34.22957	1.70165	0.09314	68.61499	32.52459	2.10963	0.10253	2.34178	**0.01919**	2.34178	**0.01919**
189007	67	8	2.00	64.30472	32.04142	2.00692	**0.04851**	194.32692	125.13089	1.55299	0.18113	2.34061	**0.01925**	2.34061	**0.01925**
193915	44	31	3.48	0.68101	20.77383	0.03278	0.97394	40.14230	16.17838	2.48123	**0.01937**	1.67660	0.09362	2.33840	**0.01937**
181059	28	47	150.64	-48.15260	20.19395	-2.38451	**0.01974**	-2.44281	2.82059	-0.86606	0.39115	-2.25483	**0.02414**	-2.33129	**0.01974**
189924	61	14	4.57	-60.08368	25.20948	-2.38338	**0.01979**	-2.45341	5.09056	-0.48195	0.63929	-1.97914	**0.04780**	-2.33022	**0.01979**
1102370	45	30	18.37	-25.48541	20.72501	-1.22969	0.22282	-16.62155	7.67561	-2.16550	**0.03935**	-2.31901	**0.02039**	-2.31901	**0.02039**
4102199	51	24	3.08	20.17826	22.59833	0.89291	0.37488	48.77255	19.44815	2.50782	**0.02043**	2.26679	**0.02340**	2.31836	**0.02043**
179864	64	11	3.64	63.99355	28.01553	2.28422	**0.02531**	66.21111	59.69590	1.10914	0.29960	2.31503	**0.02061**	2.31503	**0.02061**
3235048	48	27	23.07	-48.38194	20.44409	-2.36655	**0.02065**	-2.16861	6.11485	-0.35465	0.72595	-1.88436	0.05952	-2.31437	**0.02065**
150412	70	5	10.20	93.33865	39.45395	2.36576	**0.02069**	-0.98306	45.35544	-0.02167	0.98468	1.62240	0.10472	2.31363	**0.02069**
3138798	31	44	219.93	18.62128	20.47824	0.90932	0.36621	11.09369	4.61090	2.40597	**0.02072**	2.27447	**0.02294**	2.31299	**0.02072**

Table S4b. OTUs associated with Triglycerides at FDR < 0.05 level [a] (Cont.)

	Summary of OTUs reads			Binary model				Quantitative model				Meta analysis		Final association	
OTU	No. Absent	No. Present	mean counts in presents	Estimate	s.e.	*t* value	*P* value	Estimate	s.e.	*t* value	*P* value	Meta z value	*Meta P* value	*z*	*P value*
186133	60	15	12.33	-45.98717	24.77520	-1.85618	0.06752	-8.12557	5.27906	-1.53921	0.14970	-2.31139	**0.02081**	-2.31139	**0.02081**
198754	70	5	2.80	-56.93224	40.10147	-1.41970	0.16001	-21.44762	5.64250	-3.80108	0.06277	-2.30933	**0.02093**	-2.30933	**0.02093**
4079463	59	16	4.19	-22.29402	24.91801	-0.89470	0.37393	-24.19937	9.22253	-2.62394	**0.02103**	-2.26035	**0.02380**	-2.30748	**0.02103**
316732	14	61	101.10	7.89002	26.03172	0.30309	0.76269	-11.71085	4.93732	-2.37190	**0.02104**	-1.41802	0.15618	-2.30733	**0.02104**
3997242	71	4	1.00	103.78485	44.00119	2.35868	**0.02106**	0.00000	0.00000	0.00000	1.00000	1.63127	0.10283	2.30696	**0.02106**
187883	64	11	2.45	-39.76123	28.54934	-1.39272	0.16799	-28.67722	13.13143	-2.18386	0.06050	-2.30223	**0.02132**	-2.30223	**0.02132**
198930	67	8	2.88	-41.32565	32.50083	-1.27153	0.20763	-54.06759	20.58156	-2.62699	**0.04670**	-2.29745	**0.02159**	-2.29745	**0.02159**
4458576	23	52	72.92	-0.53224	22.12432	-0.02406	0.98087	14.75022	6.21511	2.37328	**0.02160**	1.60754	0.10794	2.29738	**0.02160**
3903651	21	54	29.11	29.56909	22.39833	1.32015	0.19097	12.75872	6.44541	1.97950	0.05317	2.29192	**0.02191**	2.29192	**0.02191**
196664	21	54	29.11	29.56909	22.39833	1.32015	0.19097	12.75872	6.44541	1.97950	0.05317	2.29192	**0.02191**	2.29192	**0.02191**
4447950	14	61	232.70	17.30970	26.17808	0.66123	0.51058	8.25941	3.51021	2.35297	**0.02204**	2.08432	**0.03713**	2.28973	**0.02204**
2403301	57	18	52.33	-38.44138	23.67794	-1.62351	0.10885	-8.67227	4.98683	-1.73903	0.10250	-2.28835	**0.02212**	-2.28835	**0.02212**
192244	58	17	5.65	-54.76460	23.49365	-2.33104	**0.02256**	0.90976	8.61323	0.10562	0.91738	-1.53948	0.12369	-2.28088	**0.02256**
179267	45	30	6.03	-40.35553	20.16209	-2.00156	**0.04910**	-8.31587	6.45589	-1.28811	0.20864	-2.28044	**0.02258**	-2.28044	**0.02258**
198587	23	52	10.27	-37.36723	21.56507	-1.73277	0.08742	-10.25507	6.66537	-1.53856	0.13035	-2.27824	**0.02271**	-2.27824	**0.02271**
189997	71	4	1.50	1.15733	45.13493	0.02564	0.97961	166.77451	6.09576	27.35910	**0.02326**	1.62261	0.10467	2.26916	**0.02326**
4463532	50	25	4.52	21.41429	21.45106	0.99829	0.32148	38.86307	16.39041	2.37109	**0.02691**	2.26569	**0.02347**	2.26569	**0.02347**
198956	46	29	2.59	-33.35409	20.74317	-1.60796	0.11222	-17.80628	10.66404	-1.66975	0.10697	-2.26293	**0.02364**	-2.26293	**0.02364**
180352	70	5	4.00	-68.84537	39.89800	-1.72553	0.08872	22.36105	3.50075	6.38750	**0.02364**	0.39646	0.69176	2.26287	**0.02364**
232222	70	5	6.60	-35.97630	40.59184	-0.88629	0.37841	21.25173	3.33615	6.37014	**0.02377**	0.97582	0.32915	2.26085	**0.02377**
191483	60	15	2.33	20.45718	25.24011	0.81050	0.42032	59.95420	23.17769	2.58672	**0.02380**	2.16812	**0.03015**	2.26032	**0.02380**
4329132	69	6	2.83	63.65980	36.87160	1.72653	0.08854	202.90912	100.63059	2.01638	0.13714	2.25545	**0.02411**	2.25545	**0.02411**
158855	67	8	11.63	-39.80098	32.51657	-1.22402	0.22493	19.67791	6.15779	3.19561	**0.02411**	0.73667	0.46132	2.25532	**0.02411**
183686	51	24	11.63	44.78100	21.09007	2.12332	**0.03716**	16.24957	14.36342	1.13132	0.27068	2.25246	**0.02429**	2.25246	**0.02429**
840376	64	11	17.09	0.58765	28.66581	0.02050	0.98370	32.59523	11.80323	2.76155	**0.02462**	1.60358	0.10881	2.24738	**0.02462**
174443	52	23	5.65	-39.41158	21.55842	-1.82813	0.07167	-16.19527	11.34105	-1.42802	0.16872	-2.24686	**0.02465**	-2.24686	**0.02465**
4469007	13	62	62.66	-4.60304	26.81843	-0.17164	0.86420	-11.39746	4.96020	-2.29778	**0.02514**	-1.70435	0.08832	-2.23929	**0.02514**
35260	69	6	14.50	-69.94902	36.46915	-1.91803	0.05907	-10.08927	6.17893	-1.63285	0.20101	-2.23894	**0.02516**	-2.23894	**0.02516**
552235	68	7	4.57	76.96918	33.66694	2.28619	**0.02519**	4.23333	82.63947	0.05123	0.96160	1.61692	0.10590	2.23852	**0.02519**
4398588	63	12	4.00	-10.40146	27.71216	-0.37534	0.70851	-32.59845	12.17097	-2.67838	**0.02527**	-1.84630	0.06485	-2.23720	**0.02527**
175148	50	25	5.04	-47.39022	20.87941	-2.26971	**0.02622**	-5.99276	7.73416	-0.77484	0.44668	-2.10993	**0.03486**	-2.22293	**0.02622**
180307	70	5	1.80	89.15115	39.32119	2.26725	**0.02638**	607.14019	587.78010	1.03294	0.41018	2.15256	**0.03135**	2.22060	**0.02638**
4299126	56	19	41.21	37.91930	22.98491	1.64975	0.10335	23.75111	14.91958	1.59194	0.13096	2.21972	**0.02644**	2.21972	**0.02644**
186732	18	57	11.37	1.13647	23.83635	0.04768	0.96210	17.89702	7.84218	2.28215	**0.02645**	1.60309	0.10891	2.21960	**0.02645**
2497335	29	46	20.37	-13.31593	20.76661	-0.64122	0.52342	16.20784	7.06461	2.29423	**0.02672**	1.11542	0.26467	2.21554	**0.02672**
208539	67	8	3.38	71.79815	31.74409	2.26178	**0.02673**	-64.47749	60.90414	-1.05867	0.33819	0.88931	0.37384	2.21542	**0.02673**
4469576	29	46	37.48	17.66814	20.76826	0.85073	0.39774	14.66200	6.39131	2.29405	**0.02673**	2.16448	**0.03043**	2.21537	**0.02673**
197890	60	15	4.20	43.02557	24.95242	1.72430	0.08894	47.54074	31.16646	1.52538	0.15308	2.21306	**0.02689**	2.21306	**0.02689**
188047	12	63	20.89	-60.93498	27.00489	-2.25644	**0.02708**	-4.19129	4.87253	-0.86019	0.39311	-2.16683	**0.03025**	-2.21037	**0.02708**
176062	41	34	33.12	-36.87512	20.92547	-1.76221	0.08228	-8.99778	6.34090	-1.41901	0.16587	-2.20844	**0.02721**	-2.20844	**0.02721**
194696	69	6	2.50	82.79768	36.84148	2.24740	**0.02768**	-196.58317	112.94388	-1.74054	0.18014	0.60915	0.54242	2.20181	**0.02768**
263518	41	34	22.88	-28.78536	20.23656	-1.42244	0.15922	-9.69650	5.54645	-1.74824	0.09032	-2.19303	**0.02831**	-2.19303	**0.02831**
191180	68	7	2.14	75.36356	33.72509	2.23464	**0.02854**	-219.62505	120.35642	-1.82479	0.14208	0.51028	0.60986	2.18972	**0.02854**
179826	60	15	2.20	14.66326	25.72088	0.57009	0.57039	100.85475	40.56138	2.48647	**0.02862**	1.94893	0.05130	2.18873	**0.02862**
180257	69	6	2.50	77.14763	36.31339	2.12450	**0.03706**	129.26654	107.05085	1.20752	0.31375	2.18670	**0.02876**	2.18670	**0.02876**
291090	65	10	19.70	64.37866	28.85791	2.23088	**0.02880**	-0.61572	28.62875	-0.02151	0.98344	1.53117	0.12573	2.18615	**0.02880**
180462	68	7	2.29	75.31237	33.75962	2.23084	**0.02881**	-232.69295	106.65093	-2.18182	0.09455	0.36363	0.71613	2.18611	**0.02881**
190980	71	4	2.00	97.60779	43.75834	2.23061	**0.02882**	261.58737	367.86899	0.71109	0.60649	1.90989	0.05615	2.18589	**0.02882**
186468	62	13	3.46	44.48865	26.27467	1.69321	0.09474	53.20290	34.67023	1.53454	0.15591	2.18489	**0.02890**	2.18489	**0.02890**
175535	13	62	10.56	-59.11020	26.52919	-2.22812	**0.02900**	-5.67483	6.21380	-0.91326	0.36482	-2.18478	**0.02890**	-2.18478	**0.02890**
177040	58	17	4.71	11.46621	24.26971	0.47245	0.63803	62.17285	25.60002	2.42862	**0.02922**	1.87451	0.06086	2.18051	**0.02922**
311947	52	23	38.52	-37.15756	21.55272	-1.72403	0.08899	-9.96520	6.95519	-1.43277	0.16737	-2.17890	**0.02934**	-2.17890	**0.02934**
183030	65	10	4.90	-64.18760	28.87632	-2.22285	**0.02937**	1.84904	8.02197	0.23050	0.82430	-1.38346	0.16652	-2.17853	**0.02937**
562244	67	8	18.25	-3.84209	32.90030	-0.11678	0.90736	22.59323	7.48547	3.01828	**0.02947**	1.45714	0.14508	2.17708	**0.02947**
195436	37	38	4.76	-43.80452	19.74541	-2.21847	**0.02968**	1.87247	7.96292	0.23515	0.81546	-1.37249	0.16991	-2.17438	**0.02968**
192127	38	37	4.57	-43.53959	19.62766	-2.21828	**0.02969**	0.04467	5.16672	0.00865	0.99315	-1.53132	0.12569	-2.17420	**0.02969**
189450	66	9	2.44	58.59327	30.73744	1.90625	0.06061	128.24232	96.21839	1.33283	0.23097	2.17379	**0.02972**	2.17379	**0.02972**
850841	68	7	17.14	-30.77180	34.89092	-0.88194	0.38074	7.79352	2.36625	3.29362	**0.03011**	0.91363	0.36091	2.16860	**0.03011**
3756485	44	31	5.19	-39.17592	20.29770	-1.93007	0.05754	-10.01165	8.40536	-1.19110	0.24361	-2.16743	**0.03020**	-2.16743	**0.03020**
211907	42	33	3.91	-44.44179	20.16538	-2.20387	**0.03073**	4.32890	6.42731	0.67352	0.50577	-1.05719	0.29042	-2.16052	**0.03073**
344525	35	40	4.93	-4.66459	20.32584	-0.22949	0.81914	25.76227	11.47582	2.24492	**0.03084**	1.36508	0.17223	2.15917	**0.03084**
358781	21	54	282.70	-47.44914	22.04635	-2.15224	**0.03473**	-2.02599	2.14130	-0.94615	0.34854	-2.15591	**0.03109**	-2.15591	**0.03109**
175520	69	6	2.33	81.00575	36.84814	2.19837	**0.03114**	-130.52129	125.14408	-1.04297	0.37361	0.89490	0.37084	2.15531	**0.03114**
849346	70	5	3.60	-66.05151	39.96355	-1.65279	0.10273	-38.54768	17.41775	-2.21313	0.15735	-2.15371	**0.03126**	-2.15371	**0.03126**
300662	71	4	4.00	-29.29696	45.16650	-0.64864	0.51863	-35.59448	1.77158	-20.09189	**0.03166**	-1.97576	**0.04818**	-2.14869	**0.03166**
350627	69	6	7.50	12.16603	37.35482	0.32569	0.74560	34.01557	8.92820	3.80990	**0.03179**	1.74760	0.08053	2.14704	**0.03179**
366794	42	33	5.21	-40.39501	19.89066	-2.03085	**0.04596**	-6.26896	5.91963	-1.05901	0.29805	-2.14703	**0.03179**	-2.14703	**0.03179**
329798	65	10	5.80	63.12643	28.89063	2.18501	**0.03214**	-12.35026	41.35487	-0.29864	0.77388	1.31192	0.18955	2.14263	**0.03214**
505670	65	10	4.00	63.21708	28.94208	2.18426	**0.03220**	-49.29793	61.30630	-0.80413	0.44777	0.97777	0.32819	2.14191	**0.03220**
4341886	63	12	1.92	40.45896	27.62267	1.46470	0.14736	123.71837	71.30404	1.73508	0.11675	2.13369	**0.03287**	2.13369	**0.03287**
185597	55	20	4.15	-22.77759	23.35955	-0.97509	0.33278	-28.81691	13.00726	-2.21545	**0.04067**	-2.13223	**0.03299**	-2.13223	**0.03299**
330414	69	6	2.83	62.52221	36.67955	1.70455	0.09259	170.95660	99.50689	1.71804	0.18429	2.12808	**0.03333**	2.12808	**0.03333**
4449054	21	54	13.20	-12.26756	22.77832	-0.53856	0.59185	-12.97377	5.94102	-2.18376	**0.03360**	-1.88157	0.05989	-2.12478	**0.03360**
194670	26	49	7.55	-39.08946	21.07954	-1.85438	0.06778	-7.67923	6.45195	-1.19022	0.24007	-2.12223	**0.03382**	-2.12223	**0.03382**
4446902	41	34	7.62	41.17358	19.82570	2.07678	**0.04139**	12.16886	12.46695	0.97609	0.33658	2.12170	**0.03386**	2.12170	**0.03386**
199421	54	21	2.95	-21.46166	22.44418	-0.95622	0.34216	-26.15673	11.83612	-2.20991	**0.04030**	-2.12169	**0.03386**	-2.12169	**0.03386**
182506	71	4	4.50	94.41359	43.76729	2.15717	**0.03433**	-32.00062	46.81307	-0.68358	0.61827	1.14400	0.25262	2.11617	**0.03433**
2203165	69	6	2.67	80.44687	37.30435	2.15650	**0.03439**	-72.55753	21.79962	-3.32839	**0.04477**	0.07690	0.93871	2.11553	**0.03439**
2724175	42	33	50.27	31.70736	20.13931	1.57440	0.11978	11.24396	7.63519	1.47265	0.15126	2.11481	**0.03445**	2.11481	**0.03445**
191738	66	9	5.78	-52.08477	30.68670	-1.69731	0.09396	19.17883	7.04598	2.72195	**0.03455**	0.31019	0.75642	2.11356	**0.03455**

Table S4b. OTUs associated with Triglycerides at FDR < 0.05 level [a] (Cont.)

	Summary of OTUs reads			Binary model				Quantitative model				Meta analysis		Final association	
OTU	No. Absent	No. Present	mean counts in presents	Estimate	s.e.	*t* value	*P* value	Estimate	s.e.	*t* value	*P* value	Meta z value	*Meta P* value	*z*	*P value*
180341	70	5	1.60	-40.02909	40.49389	-0.98852	0.32621	71.01187	13.55847	5.23745	**0.03458**	0.80010	0.42365	2.11329	**0.03458**
210095	69	6	2.83	58.72751	36.73604	1.59864	0.11428	340.34626	183.16459	1.85814	0.16012	2.10993	**0.03486**	2.10993	**0.03486**
197458	65	10	2.40	-38.12312	29.49843	-1.29238	0.20036	25.08899	9.66463	2.59596	**0.03563**	0.58021	0.56177	2.10108	**0.03563**
4427290	43	32	17.88	-25.33008	20.43265	-1.23969	0.21912	12.03329	5.46772	2.20079	**0.03587**	0.61488	0.53863	2.09845	**0.03587**
216550	67	8	34.75	43.04540	32.87647	1.30931	0.19459	24.77391	12.09462	2.04834	0.09584	2.09477	**0.03619**	2.09477	**0.03619**
184394	57	18	4.67	14.24159	24.28375	0.58647	0.55940	68.76863	29.89382	2.30043	**0.03619**	1.89397	0.05823	2.09474	**0.03619**
4439487	62	13	4.31	-51.23971	26.10853	-1.96257	0.05356	-9.66298	8.90679	-1.08490	0.30343	-2.09267	**0.03638**	-2.09267	**0.03638**
4479443	65	10	2.90	59.26078	29.01317	2.04255	**0.04476**	50.29553	49.41583	1.01780	0.34265	2.09010	**0.03661**	2.09010	**0.03661**
176318	46	29	31.45	-15.63404	20.78516	-0.75217	0.45440	-14.03128	6.38056	-2.19907	**0.03697**	-2.00410	**0.04506**	-2.08612	**0.03697**
186090	64	11	4.09	55.60762	28.47208	1.95306	0.05470	51.19910	46.58712	1.09900	0.30374	2.08575	**0.03700**	2.08575	**0.03700**
215231	42	33	3.91	-41.51987	19.83661	-2.09309	**0.03986**	-7.80261	8.65428	-0.90159	0.37446	-2.08123	**0.03741**	-2.08123	**0.03741**
185584	45	30	5.60	-40.45630	20.39153	-1.98398	0.05107	-8.51460	8.45440	-1.00712	0.32282	-2.07859	**0.03766**	-2.07859	**0.03766**
184525	65	10	1.70	-45.66193	29.43911	-1.55106	0.12527	-33.20005	21.10459	-1.57312	0.15969	-2.07828	**0.03768**	-2.07828	**0.03768**
4381749	55	20	4.10	-44.80400	22.33716	-2.00581	**0.04864**	-9.94617	10.03171	-0.99147	0.33536	-2.07547	**0.03794**	-2.07547	**0.03794**
191153	11	64	130.27	-58.75910	27.86037	-2.10906	**0.03842**	-2.52817	4.06322	-0.62221	0.53612	-1.90145	0.05724	-2.07036	**0.03842**
3474081	70	5	2.00	-28.65714	40.51467	-0.70733	0.48165	48.56801	9.80857	4.95159	**0.03845**	0.96618	0.33396	2.07004	**0.03845**
212686	61	14	3.07	45.41081	25.50414	1.78053	0.07921	51.28357	41.40454	1.23860	0.24127	2.06978	**0.03847**	2.06978	**0.03847**
176297	68	7	2.00	71.31843	33.83512	2.10782	**0.03853**	-176.10191	132.65282	-1.32754	0.25503	0.65829	0.51035	2.06919	**0.03853**
215468	68	7	3.57	-53.75607	34.38502	-1.56336	0.12235	-18.95715	11.25971	-1.68363	0.16754	-2.06837	**0.03861**	-2.06837	**0.03861**
4379889	55	20	4.70	16.60081	23.01108	0.72143	0.47298	53.69522	23.99379	2.23788	**0.03891**	1.96775	**0.04910**	2.06518	**0.03891**
195937	27	48	9.63	-43.32982	20.63266	-2.10006	**0.03923**	-4.16877	6.36709	-0.65474	0.51597	-1.91722	0.05521	-2.06179	**0.03923**
293883	58	17	13.82	-21.40866	24.10232	-0.88824	0.37737	21.43180	9.43769	2.27087	**0.03947**	0.83193	0.40545	2.05928	**0.03947**
4439469	10	65	24.65	13.20769	29.79212	0.44333	0.65886	13.05191	6.21000	2.10176	**0.03964**	1.76701	0.07723	2.05744	**0.03964**
188449	67	8	3.50	-66.96702	31.96559	-2.09497	**0.03969**	3.49068	9.46243	0.36890	0.72731	-1.20790	0.22708	-2.05694	**0.03969**
193709	21	54	7.06	-46.00061	21.97118	-2.09368	**0.03981**	7.83309	8.27586	0.94650	0.34836	-0.79049	0.42924	-2.05571	**0.03981**
3424669	69	6	2.33	75.98308	36.31415	2.09238	**0.03993**	-2.60735	184.99395	-0.01409	0.98964	1.44355	0.14887	2.05447	**0.03993**
361507	66	9	143.67	24.05141	31.08120	0.77383	0.44157	14.23496	5.45257	2.61069	**0.04008**	1.99576	**0.04596**	2.05289	**0.04008**
177828	63	12	3.08	-56.11179	26.86050	-2.08901	**0.04024**	-6.32096	9.09121	-0.69528	0.50443	-1.92247	0.05455	-2.05125	**0.04024**
2066056	59	16	7.56	-50.49231	24.17159	-2.08891	**0.04025**	4.21614	6.71089	0.62825	0.54072	-1.01783	0.30876	-2.05116	**0.04025**
184770	50	25	4.28	-44.20894	21.17769	-2.08752	**0.04038**	-4.96994	8.96057	-0.55465	0.58473	-1.83588	0.06638	-2.04984	**0.04038**
4324985	67	8	4.63	-66.51257	31.90392	-2.08478	**0.04064**	12.03707	10.76482	1.11819	0.31431	-0.73610	0.46167	-2.04722	**0.04064**
589329	67	8	1.63	-30.08565	32.66293	-0.92109	0.36008	-38.74674	14.86474	-2.60662	**0.04786**	-2.04622	**0.04074**	-2.04622	**0.04074**
953855	67	8	1.63	66.31854	32.05907	2.06864	**0.04217**	-65.16558	129.69108	-0.50247	0.63668	1.10272	0.27015	2.03182	**0.04217**
173876	45	30	23.07	-41.61592	20.12937	-2.06742	**0.04229**	4.14216	5.94896	0.69628	0.49220	-0.95025	0.34199	-2.03066	**0.04229**
193528	10	65	122.38	2.38509	29.83729	0.07994	0.93651	-11.32880	5.48366	-2.06592	**0.04302**	-1.37451	0.16928	-2.02350	**0.04302**
4332078	59	16	3.81	9.33070	24.91611	0.37448	0.70914	86.14523	38.42803	2.24173	**0.04306**	1.69432	0.09020	2.02313	**0.04306**
187868	68	7	1.71	-25.56174	34.75370	-0.73551	0.46442	44.95000	15.37254	2.92405	**0.04307**	0.91319	0.36114	2.02304	**0.04307**
177660	66	9	3.44	-63.03137	30.61994	-2.05851	**0.04316**	-8.00742	24.08623	-0.33245	0.75086	-1.65439	0.09805	-2.02215	**0.04316**
312770	67	8	4.88	-47.43889	32.37564	-1.46527	0.14720	-13.56219	8.19078	-1.65579	0.15867	-2.02166	**0.04321**	-2.02166	**0.04321**
198209	69	6	2.33	-26.88959	37.25051	-0.72186	0.47272	53.30309	15.79519	3.37464	**0.04326**	0.92144	0.35682	2.02117	**0.04326**
186866	48	27	10.22	41.31081	20.72631	1.99316	0.05003	15.79267	17.24843	0.91560	0.36898	2.02094	**0.04329**	2.02094	**0.04329**
199344	64	11	3.27	-36.48136	28.34375	-1.28710	0.20218	-36.13552	20.33738	-1.77680	0.11350	-2.02092	**0.04329**	-2.02092	**0.04329**
234443	67	8	6.88	-13.02119	33.15773	-0.39270	0.69570	-25.30199	9.40661	-2.68981	**0.04331**	-1.70544	0.08811	-2.02072	**0.04331**
3430935	69	6	17.33	-36.60956	37.24305	-0.98299	0.32890	-15.43985	5.25544	-2.93788	0.06061	-2.01711	**0.04368**	-2.01711	**0.04368**
199081	64	11	5.36	57.23552	27.91238	2.05054	**0.04395**	13.49510	44.39096	0.30401	0.76888	1.63227	0.10262	2.01454	**0.04395**
186997	40	35	4.14	-7.72703	20.30765	-0.38050	0.70470	30.51314	14.55102	2.09698	**0.04399**	1.15629	0.24756	2.01423	**0.04399**
553611	45	30	9.03	-41.39126	20.40109	-2.02888	**0.04617**	10.85141	5.13887	2.11163	**0.04411**	0.01355	0.98919	2.01300	**0.04411**
194672	36	39	8.36	-29.34645	20.01261	-1.46640	0.14689	-11.54542	8.12299	-1.42133	0.16383	-2.01024	**0.04441**	-2.01024	**0.04441**
4094866	57	18	5.89	44.46619	23.16957	1.91916	0.05893	27.48103	27.96941	0.98254	0.34142	2.00826	**0.04462**	2.00826	**0.04462**
4340358	62	13	4.85	-29.73159	26.60440	-1.11754	0.26748	-24.50329	12.75516	-1.92105	0.08366	-2.00727	**0.04472**	-2.00727	**0.04472**
185164	40	35	9.03	-31.55273	19.99885	-1.57773	0.11901	-9.25878	7.09041	-1.30582	0.20092	-2.00667	**0.04479**	-2.00667	**0.04479**
182431	52	23	7.65	-44.05041	21.57865	-2.04139	**0.04488**	-4.04268	7.58839	-0.53275	0.60008	-1.78904	0.07361	-2.00580	**0.04488**
147702	30	45	27.76	-10.87441	20.68496	-0.52572	0.60070	-16.19948	7.83595	-2.06733	**0.04490**	-1.78825	0.07374	-2.00557	**0.04490**
288651	26	49	32.88	-42.34579	20.76412	-2.03937	**0.04508**	9.72487	6.09121	1.59654	0.11722	-0.30922	0.75715	-2.00387	**0.04508**
539647	60	15	19.27	-50.23989	24.66023	-2.03728	**0.04530**	10.45396	5.96488	1.75258	0.10516	-0.26979	0.78732	-2.00188	**0.04530**
4480861	48	27	61.48	-41.98303	20.62267	-2.03577	**0.04545**	-0.50362	4.92345	-0.10229	0.91938	-1.48609	0.13725	-2.00043	**0.04545**
194104	70	5	3.20	81.33446	39.97806	2.03448	**0.04559**	123.22147	157.51848	0.78227	0.51597	1.87296	0.06107	1.99919	**0.04559**
4431545	44	31	4.13	22.37017	20.62569	1.08458	0.28173	32.33352	17.83162	1.81327	0.08052	1.99694	**0.04583**	1.99694	**0.04583**
370086	45	30	20.33	-41.86603	20.60485	-2.03185	**0.04586**	-1.28577	5.93926	-0.21649	0.83024	-1.56347	0.11794	-1.99669	**0.04586**
1860112	60	15	8.60	38.23811	25.05052	1.52644	0.13128	30.67974	22.01897	1.39333	0.18879	1.99633	**0.04590**	1.99633	**0.04590**
185949	70	5	3.60	-22.74211	40.57511	-0.56049	0.57688	38.85712	8.63679	4.49902	**0.04602**	1.01630	0.30949	1.99521	**0.04602**
306299	50	25	7.36	25.59270	21.43467	1.19399	0.23640	31.20253	18.25723	1.70905	0.10151	1.99517	**0.04602**	1.99517	**0.04602**
540055	49	26	15.96	-42.08913	20.73391	-2.02997	**0.04606**	0.77178	6.34430	0.12165	0.90423	-1.32552	0.18500	-1.99488	**0.04606**
1600780	69	6	3.67	62.57080	36.74218	1.70297	0.09289	91.30609	64.94745	1.40585	0.25444	1.99403	**0.04615**	1.99403	**0.04615**
190649	41	34	4.21	-40.34723	19.94520	-2.02290	**0.04680**	-6.19852	8.35478	-0.74191	0.46372	-1.92394	0.05436	-1.98813	**0.04680**
1726408	70	5	40.80	-38.11288	40.46609	-0.94185	0.34942	17.60601	3.99602	4.40589	**0.04785**	0.73751	0.46081	1.97872	**0.04785**
4300690	42	33	100.88	-40.19395	19.96728	-2.01299	**0.04786**	-1.69163	3.61119	-0.46844	0.64286	-1.72701	0.08417	-1.97865	**0.04786**
2506486	41	34	4.32	32.92910	20.02023	1.64479	0.10437	20.61654	17.32413	1.19005	0.24306	1.97381	**0.04840**	1.97381	**0.04840**
509621	68	7	255.00	68.13465	34.01385	2.00314	**0.04893**	-26.03839	26.53066	-0.98145	0.38194	0.77421	0.43880	1.96923	**0.04893**
190772	45	30	6.20	-26.28120	20.81020	-1.26290	0.21070	-15.50280	9.81137	-1.58008	0.12573	-1.96773	**0.04910**	-1.96773	**0.04910**
3195723	52	23	6.52	36.73227	21.68265	1.69409	0.09457	31.38633	27.62184	1.13629	0.26927	1.96329	**0.04961**	1.96329	**0.04961**
189708	67	8	2.25	-63.97242	32.04581	-1.99628	**0.04969**	9.52509	13.84101	0.68818	0.52196	-0.93503	0.34977	-1.96266	**0.04969**

[a] Statistics associated to the two part model for association analysis between the trait and OTUs adjusting for age and gender. The binomial analysis that tests for association of detecting a microbe represented by an OTU with the trait, where s.e. represents the standard error of the estimate, *t* -value of a *t* test for the estimate and its associated probability (*P* value). The quantitative analysis tests for association between the trait and the abundance of the OTU for the subjects where that OTU is present, where s.e. represents the standard error of the estimate, *t* -value of a *t* test for the estimate and its associated probability (*P* value). The Meta analysis combine the effect of both binary and quantitative analysis, a meta *P* value was derived using an unweighted Z method. The final association *P* value per OTU-trait pair was assigned from the minimum of *P* values from the binary analysis, quantitative analysis, and meta-analysis.

Table S4c. OTUs associated with High-density lipoprotein (HDL) at FDR < 0.05 level [a]

	Summary of OTUs reads			Binary model				Quantitative model				Meta analysis		Final association	
OTU	No. Absent	No. Present	mean counts in presents	Estimate	s.e.	*t* value	*P* value	Estimate	s.e.	*t* value	*P* value	Meta z value	*Meta P* value	*z*	*P value*
299302	66	9	3.33	17.62727	3.94662	4.46642	**0.00003**	1.96800	10.36870	0.18980	0.85572	3.08572	**0.00203**	4.18204	**0.00003**
345301	65	10	4.90	13.79417	3.94346	3.49798	**0.00081**	11.54877	3.40796	3.38876	**0.01162**	4.15331	**0.00003**	4.15331	**0.00003**
4324985	67	8	4.63	17.97815	4.18987	4.29086	**0.00005**	10.11734	8.87482	1.14001	0.30593	3.57730	**0.00035**	4.03528	**0.00005**
35260	69	6	14.50	19.50821	4.82356	4.04436	**0.00013**	9.70897	3.60326	2.69450	0.07413	3.96825	**0.00007**	3.96825	**0.00007**
177828	63	12	3.08	14.68128	3.55464	4.13017	**0.00010**	-3.83281	6.09315	-0.62904	0.54496	2.32922	**0.01985**	3.89935	**0.00010**
3826120	71	4	1.75	23.67715	5.82352	4.06578	**0.00012**	-7.51531	51.89484	-0.14482	0.90844	2.63713	**0.00836**	3.84446	**0.00012**
749837	61	14	2.71	2.37180	3.70953	0.63938	0.52460	-30.34829	5.25162	-5.77884	**0.00012**	-2.26537	**0.02349**	-3.83998	**0.00012**
189459	70	5	2.00	21.30268	5.26876	4.04321	**0.00013**	-10.82277	20.66139	-0.52382	0.65267	2.38656	**0.01701**	3.82516	**0.00013**
190980	71	4	2.00	22.50777	5.89703	3.81680	**0.00028**	-9.85350	45.46907	-0.21671	0.86414	2.44577	**0.01445**	3.62995	**0.00028**
218710	67	8	3.75	16.28590	4.31329	3.77575	**0.00033**	-0.92213	9.18546	-0.10039	0.92394	2.47401	**0.01336**	3.59425	**0.00033**
180352	70	5	4.00	19.73198	5.33380	3.69942	**0.00042**	-17.21734	16.48598	-1.04436	0.40595	1.90676	0.05655	3.52760	**0.00042**
925131	69	6	76.17	18.09452	4.89841	3.69396	**0.00043**	-25.20579	12.92924	-1.94952	0.14634	1.46389	0.14322	3.52282	**0.00043**
199335	66	9	2.78	14.88843	4.10316	3.62853	**0.00053**	18.45221	10.92839	1.68847	0.14229	3.48798	**0.00049**	3.48798	**0.00049**
589989	71	4	4.75	21.06672	6.18516	3.40601	**0.00108**	101.10681	16.04890	6.29992	0.10022	3.47346	**0.00051**	3.47346	**0.00051**
1599042	63	12	21.33	13.24711	3.64254	3.63677	**0.00052**	-3.13136	4.42606	-0.70748	0.49718	1.97546	**0.04822**	3.47266	**0.00052**
196724	70	5	1.60	15.60220	5.55728	2.80752	**0.00642**	30.49865	5.37812	5.67087	**0.02972**	3.46439	**0.00053**	3.46439	**0.00053**
186913	70	5	2.00	20.04026	5.52797	3.62525	**0.00054**	-13.29087	20.14783	-0.65967	0.57727	2.05425	**0.03995**	3.46253	**0.00054**
174443	52	23	5.65	8.23427	2.99858	2.74606	**0.00761**	7.66099	3.24795	2.35872	**0.02862**	3.43469	**0.00059**	3.43469	**0.00059**
174792	59	16	4.13	3.73691	3.57408	1.04556	0.29926	17.27877	3.87030	4.46445	**0.00064**	3.14886	**0.00164**	3.41514	**0.00064**
337161	62	13	9.46	12.35754	3.54168	3.48917	**0.00083**	0.01305	4.65352	0.00280	0.99782	2.36533	**0.01801**	3.34235	**0.00083**
323903	59	16	5.06	6.69106	3.44924	1.93986	0.05631	8.19809	2.47053	3.31835	**0.00555**	3.31063	**0.00093**	3.31063	**0.00093**
1044419	70	5	155.80	18.54577	5.38472	3.44415	**0.00096**	2.01872	10.11859	0.19951	0.86031	2.45956	**0.01391**	3.30236	**0.00096**
177567	67	8	2.38	12.89635	4.45258	2.89637	**0.00500**	24.42468	10.34620	2.36074	0.06469	3.29134	**0.00100**	3.29134	**0.00100**
187196	71	4	2.25	20.47051	5.98244	3.42177	**0.00103**	-31.69466	23.96901	-1.32232	0.41220	1.74119	0.08165	3.28244	**0.00103**
198422	45	30	6.37	-0.43555	2.96303	-0.14699	0.88355	5.73123	1.57951	3.62848	**0.00117**	2.19139	**0.02842**	3.24557	**0.00117**
186030	67	8	2.25	14.73935	4.37715	3.36734	**0.00122**	2.91703	14.12332	0.20654	0.84452	2.42538	**0.01529**	3.23389	**0.00122**
370357	69	6	2.33	17.25459	5.13028	3.36328	**0.00124**	2.17277	14.67361	0.14807	0.89168	2.38044	**0.01729**	3.23026	**0.00124**
3134492	13	62	139.89	3.53904	3.80994	0.92890	0.35605	-2.20122	0.65553	-3.35791	**0.00138**	-1.60931	0.10755	-3.19883	**0.00138**
310633	71	4	10.00	20.08285	6.03538	3.32752	**0.00138**	6.67886	13.00793	0.51345	0.69802	2.53587	**0.01122**	3.19827	**0.00138**
188316	68	7	2.29	15.46694	4.65931	3.31958	**0.00142**	23.96252	17.82645	1.34421	0.25005	3.06981	**0.00214**	3.19115	**0.00142**
291054	70	5	14.20	18.10862	5.45606	3.31899	**0.00142**	7.52789	8.81516	0.85397	0.48308	2.75204	**0.00592**	3.19063	**0.00142**
362955	66	9	16.44	8.42441	4.37383	1.92609	0.05804	-15.97840	2.96730	-5.38483	**0.00169**	-0.88026	0.37872	-3.14027	**0.00169**
4439360	7	68	95.34	4.66983	4.95560	0.94233	0.34917	-2.67298	0.81664	-3.27312	**0.00171**	-1.55632	0.11963	-3.13717	**0.00171**
179499	68	7	2.86	15.14678	4.68682	3.23178	**0.00186**	-11.49899	10.45738	-1.09961	0.33324	1.51650	0.12939	3.11227	**0.00186**
185597	55	20	4.15	3.55513	3.33415	1.06628	0.28986	9.93404	2.70091	3.67803	**0.00186**	2.94821	**0.00320**	3.11098	**0.00186**
181560	60	15	6.00	10.95721	3.39655	3.22598	**0.00189**	3.08662	4.32610	0.71349	0.48919	2.68605	**0.00723**	3.10705	**0.00189**
352747	67	8	2.88	14.01221	4.39544	3.18789	**0.00212**	-3.71022	9.21262	-0.40273	0.70379	1.90387	0.05693	3.07270	**0.00212**
299755	71	4	5.00	19.28252	6.07152	3.17590	**0.00220**	-9.61323	91.28508	-0.10531	0.93320	2.10579	**0.03522**	3.06186	**0.00220**
300662	71	4	4.00	-0.78656	6.47309	-0.12151	0.90362	3.95724	0.01401	282.37870	**0.00225**	2.07423	**0.03806**	3.05448	**0.00225**
4439487	62	13	4.31	11.32469	3.58970	3.15477	**0.00234**	6.50974	4.84996	1.34222	0.20920	3.03952	**0.00237**	3.04276	**0.00234**
193969	61	14	21.43	11.11664	3.52854	3.15049	**0.00237**	-0.55228	3.61776	-0.15266	0.88143	2.04335	**0.04102**	3.03888	**0.00237**
183822	68	7	2.00	14.76980	4.68894	3.14992	**0.00238**	-16.23800	15.42116	-1.05297	0.35176	1.49000	0.13622	3.03837	**0.00238**
319197	70	5	1.40	13.49346	5.60215	2.40862	**0.01857**	73.77130	18.10403	4.07486	0.05528	3.01981	**0.00253**	3.01981	**0.00253**
186851	60	15	7.20	10.65624	3.41245	3.12275	**0.00258**	-4.34971	4.61320	-0.94288	0.36434	1.48961	0.13633	3.01376	**0.00258**
4405146	49	26	51.81	8.81930	2.86746	3.07564	**0.00297**	-1.68362	1.32528	-1.27039	0.21665	1.22719	0.21975	2.97101	**0.00297**
4391262	49	26	15.23	-0.55207	3.10316	-0.17790	0.85930	2.47946	0.74916	3.30967	**0.00306**	1.96898	**0.04895**	2.96183	**0.00306**
184534	66	9	14.67	12.86452	4.21851	3.04954	**0.00321**	-9.39345	3.90109	-2.40790	0.05272	0.71424	0.47508	2.94727	**0.00321**
3785400	69	6	56.67	15.33582	5.02956	3.04914	**0.00321**	-10.07777	2.97832	-3.38371	**0.04297**	0.65261	0.51401	2.94690	**0.00321**
215468	68	7	3.57	14.22853	4.70705	3.02281	**0.00347**	3.04877	9.88238	0.30851	0.77310	2.27070	**0.02317**	2.92292	**0.00347**
337285	64	11	4.00	11.61347	3.86571	3.00423	**0.00366**	4.31380	6.99879	0.61636	0.55478	2.47246	**0.01342**	2.90597	**0.00366**
1102370	45	30	18.37	8.09690	2.83664	2.85440	**0.00563**	2.23568	1.63243	1.36954	0.18211	2.90125	**0.00372**	2.90125	**0.00372**
177503	62	13	2.85	10.31740	3.64967	2.82694	**0.00608**	8.29932	5.70825	1.45392	0.17662	2.89536	**0.00379**	2.89536	**0.00379**
175729	54	21	4.29	7.16654	3.14529	2.27849	**0.02567**	6.09643	3.07317	1.98376	0.06275	2.89363	**0.00381**	2.89363	**0.00381**
181826	60	15	3.47	6.92039	3.53113	1.95982	0.05389	10.50687	4.30979	2.43791	**0.03128**	2.88589	**0.00390**	2.88589	**0.00390**
177660	66	9	3.44	12.66617	4.24833	2.98145	**0.00391**	3.34835	17.35082	0.19298	0.85334	2.17084	**0.02994**	2.88517	**0.00391**
229348	64	11	13.64	11.46800	3.86893	2.96413	**0.00411**	-4.75054	3.29744	-1.44067	0.18764	1.09726	0.27253	2.86934	**0.00411**
105287	64	11	3.09	3.17775	4.15862	0.76413	0.44728	9.94492	2.50469	3.97053	**0.00412**	2.56615	**0.01028**	2.86914	**0.00412**
4111715	50	25	5.60	-1.73332	3.14098	-0.55184	0.58277	2.95515	0.92315	3.20115	**0.00412**	1.64007	0.10099	2.86876	**0.00412**
701221	66	9	2.11	10.67676	4.28582	2.49118	**0.01504**	21.00520	10.97443	1.91401	0.10413	2.86848	**0.00412**	2.86848	**0.00412**
4295707	49	26	67.85	8.52019	2.87618	2.96233	**0.00413**	0.40298	1.40831	0.28614	0.77733	2.22774	**0.02590**	2.86770	**0.00413**
2309802	69	6	4.50	11.57522	5.18436	2.23272	**0.02868**	17.16839	5.92114	2.89951	0.06253	2.86409	**0.00418**	2.86409	**0.00418**
293883	58	17	13.82	9.67151	3.27045	2.95724	**0.00420**	1.31984	2.69372	0.48997	0.63174	2.36337	**0.01811**	2.86304	**0.00420**
183681	43	32	5.41	8.17531	2.77312	2.94806	**0.00431**	0.95617	2.43591	0.39253	0.69753	2.29336	**0.02183**	2.85464	**0.00431**
4381054	55	20	3.00	7.26727	3.16353	2.29720	**0.02452**	6.97545	3.69598	1.88731	0.07631	2.84357	**0.00446**	2.84357	**0.00446**
180982	38	37	16.59	-8.01179	2.74122	-2.92271	**0.00463**	0.58902	0.69122	0.85215	0.40009	-1.40712	0.15939	-2.83143	**0.00463**
186077	62	13	4.69	10.01256	3.64270	2.74867	**0.00756**	5.39289	3.77800	1.42744	0.18392	2.82839	**0.00468**	2.82839	**0.00468**
185164	40	35	9.03	1.06025	2.90442	0.36505	0.71615	4.83973	1.60845	3.00894	**0.00508**	2.23849	**0.02519**	2.80208	**0.00508**
2123717	36	39	6.79	-6.53374	2.79690	-2.33606	**0.02228**	-1.51686	0.88321	-1.71743	0.09449	-2.79858	**0.00513**	-2.79858	**0.00513**
798581	39	36	7.00	7.89833	2.74777	2.87446	**0.00532**	-0.75431	1.70048	-0.44358	0.66024	1.65998	0.09692	2.78715	**0.00532**
195756	71	4	1.50	-9.57561	6.35063	-1.50782	0.13598	-3.12732	0.07072	-44.22135	**0.01439**	-2.78475	**0.00536**	-2.78475	**0.00536**
189235	68	7	8.29	13.56502	4.73816	2.86293	**0.00549**	8.14933	9.04302	0.90117	0.41845	2.53545	**0.01123**	2.77655	**0.00549**
849346	70	5	3.60	12.63987	5.62478	2.24718	**0.02769**	47.49714	14.93665	3.17991	0.08629	2.76967	**0.00561**	2.76967	**0.00561**
4356062	71	4	1.25	1.02975	6.49349	0.15858	0.87444	-14.95717	0.13392	-111.68710	**0.00570**	-1.84311	0.06531	-2.76456	**0.00570**
4439489	70	5	3.40	15.82230	5.56541	2.84297	**0.00581**	9.64684	22.54162	0.42796	0.71036	2.21294	**0.02690**	2.75819	**0.00581**
199344	64	11	3.27	9.18620	3.95143	2.32478	**0.02291**	12.49360	6.90701	1.80883	0.10808	2.74487	**0.00605**	2.74487	**0.00605**
197343	69	6	2.17	1.15183	5.45921	0.21099	0.83349	30.20840	4.40137	6.86341	**0.00633**	2.07906	**0.03761**	2.73002	**0.00633**
4333897	63	12	2.67	0.47799	3.95454	0.12087	0.90413	5.71353	1.62809	3.50934	**0.00663**	2.00504	**0.04496**	2.71510	**0.00663**

Table S4c. OTUs associated with High-density lipoprotein (HDL) at FDR < 0.05 level [a] (Cont.)

	Summary of OTUs reads			Binary model				Quantitative model				Meta analysis		Final association	
OTU	No. Absent	No. Present	mean counts in presents	Estimate	s.e.	*t* value	*P* value	Estimate	s.e.	*t* value	*P* value	Meta z value	*Meta P* value	*z*	*P value*
172878	71	4	3.25	15.75979	6.20616	2.53938	**0.01327**	36.93982	10.66263	3.46442	0.17890	2.70166	**0.00690**	2.70166	**0.00690**
4323555	70	5	2.60	-3.10597	5.79985	-0.53553	0.59394	-10.01477	0.85345	-11.73452	**0.00718**	-2.27782	**0.02274**	-2.68819	**0.00718**
158855	67	8	11.63	7.73045	4.60594	1.67837	0.09761	-5.03253	1.15402	-4.36088	**0.00728**	-0.72621	0.46771	-2.68356	**0.00728**
191251	24	51	13.16	-0.89823	3.14401	-0.28570	0.77593	-2.38373	0.85158	-2.79920	**0.00736**	-2.09649	**0.03604**	-2.68026	**0.00736**
312586	69	6	3.00	13.96949	5.08851	2.74530	**0.00763**	10.51387	19.84381	0.52983	0.63294	2.22432	**0.02613**	2.66806	**0.00763**
253471	71	4	7.25	15.59584	6.19092	2.51915	**0.01398**	15.64514	4.80297	3.25739	0.18962	2.66533	**0.00769**	2.66533	**0.00769**
300374	43	32	6.13	6.03575	2.89543	2.08457	**0.04066**	3.52385	1.97762	1.78187	0.08525	2.66441	**0.00771**	2.66441	**0.00771**
191483	60	15	2.33	-5.00453	3.57526	-1.39977	0.16588	-6.44760	2.34774	-2.74630	**0.01772**	-2.65654	**0.00789**	-2.65654	**0.00789**
180312	51	24	4.92	3.71362	3.08292	1.20458	0.23231	6.91502	2.47323	2.79594	**0.01083**	2.64645	**0.00813**	2.64645	**0.00813**
113919	61	14	93.21	9.62924	3.54437	2.71677	**0.00825**	1.04774	1.86362	0.56220	0.58525	2.25382	**0.02421**	2.64165	**0.00825**
180136	45	30	7.27	3.01905	3.05016	0.98980	0.32558	7.16313	2.51740	2.84545	**0.00836**	2.55981	**0.01047**	2.63707	**0.00836**
168439	44	31	33.58	-7.60837	2.93351	-2.59361	**0.01150**	-0.99332	0.80851	-1.22858	0.22946	-2.63677	**0.00837**	-2.63677	**0.00837**
178015	65	10	2.70	11.02965	4.06985	2.71009	**0.00840**	-14.64466	12.28010	-1.19255	0.27190	1.08665	0.27719	2.63546	**0.00840**
4421273	40	35	9.49	5.87661	2.85487	2.05845	**0.04317**	2.75777	1.56814	1.75862	0.08820	2.63542	**0.00840**	2.63542	**0.00840**
2298935	20	55	31.00	0.23284	3.27730	0.07105	0.94356	-2.46648	0.90111	-2.73715	**0.00846**	-1.81178	0.07002	-2.63304	**0.00846**
4481861	69	6	5.17	13.78793	5.09539	2.70596	**0.00850**	-11.94548	5.46068	-2.18755	0.11652	0.75101	0.45264	2.63163	**0.00850**
3138798	31	44	219.93	-5.55266	2.86972	-1.93491	0.05693	-0.88308	0.47648	-1.85336	0.07104	-2.62273	**0.00872**	-2.62273	**0.00872**
176062	41	34	33.12	7.84513	2.91106	2.69494	**0.00876**	1.75235	1.59044	1.10180	0.27903	2.61906	**0.00882**	2.62141	**0.00876**
851733	59	16	70.75	-4.24930	3.50354	-1.21286	0.22915	-1.34752	0.46378	-2.90549	**0.01228**	-2.62097	**0.00877**	-2.62097	**0.00877**
368261	70	5	2.20	-5.40532	5.82918	-0.92729	0.35688	2.61971	0.24699	10.60668	**0.00877**	1.20172	0.22947	2.62082	**0.00877**
2250985	34	41	10.93	-5.34829	2.84594	-1.87927	0.06425	-1.85415	0.97858	-1.89473	0.06576	-2.60957	**0.00907**	-2.60957	**0.00907**
3903651	21	54	29.11	-8.28147	3.08903	-2.68093	**0.00910**	-0.58679	0.67143	-0.87394	0.38625	-2.45710	**0.01401**	-2.60841	**0.00910**
196664	21	54	29.11	-8.28147	3.08903	-2.68093	**0.00910**	-0.58679	0.67143	-0.87394	0.38625	-2.45710	**0.01401**	-2.60841	**0.00910**
111135	70	5	11.00	14.83936	5.54200	2.67762	**0.00918**	-0.27015	10.27993	-0.02628	0.98142	1.82579	0.06788	2.60534	**0.00918**
4468234	1	74	18.84	0.00000	0.00000	0.00000	1.00000	-2.35815	0.88092	-2.67692	**0.00922**	-1.84111	0.06560	-2.60373	**0.00922**
183698	66	9	4.00	11.54758	4.32898	2.66751	**0.00943**	-2.97801	10.77117	-0.27648	0.79146	1.64864	0.09922	2.59595	**0.00943**
305141	61	14	9.14	8.91168	3.56825	2.49749	**0.01479**	-8.67442	2.76678	-3.13521	**0.00949**	-0.11068	0.91187	-2.59391	**0.00949**
157470	64	11	2.73	5.43436	4.04985	1.34187	0.18386	15.76314	5.41324	2.91196	**0.01953**	2.59100	**0.00957**	2.59100	**0.00957**
360508	42	33	9.12	6.31233	2.82518	2.23431	**0.02857**	4.51120	2.97952	1.51407	0.14047	2.59043	**0.00959**	2.59043	**0.00959**
346639	66	9	4.67	11.36127	4.28058	2.65415	**0.00978**	0.81080	6.46756	0.12536	0.90433	1.91183	0.05590	2.58354	**0.00978**
177062	65	10	3.70	9.91395	4.10379	2.41580	**0.01824**	11.08487	7.79551	1.42196	0.19803	2.57947	**0.00990**	2.57947	**0.00990**
2506486	41	34	4.32	-7.37126	2.78195	-2.64967	**0.00990**	-0.68254	1.23900	-0.55088	0.58567	-2.20936	**0.02715**	-2.57938	**0.00990**
191660	69	6	1.67	11.08908	5.18745	2.13768	**0.03594**	30.49819	14.30504	2.13199	0.12278	2.57445	**0.01004**	2.57445	**0.01004**
1522739	71	4	2.25	3.72494	6.43723	0.57866	0.56463	-33.30790	0.53109	-62.71624	**0.01015**	-1.41046	0.15840	-2.57068	**0.01015**
3709990	63	12	3.08	-6.54969	3.96537	-1.65172	0.10295	-5.71672	2.46507	-2.31909	**0.04555**	-2.56697	**0.01026**	-2.56697	**0.01026**
180473	54	21	4.05	8.12393	3.08269	2.63534	**0.01029**	-0.97962	2.94750	-0.33236	0.74346	1.58305	0.11341	2.56605	**0.01029**
4483337	17	58	50.33	1.86103	3.48801	0.53355	0.59529	-1.81917	0.68811	-2.64373	**0.01066**	-1.43003	0.15271	-2.55355	**0.01066**
4444262	65	10	13.70	-3.56985	4.26389	-0.83723	0.40523	4.24689	1.23243	3.44594	**0.01075**	1.21509	0.22433	2.55071	**0.01075**
306299	50	25	7.36	-7.73511	2.95613	-2.61664	**0.01081**	1.81627	1.45256	1.25039	0.22429	-0.94289	0.34573	-2.54864	**0.01081**
4319785	66	9	131.67	11.20635	4.28371	2.61604	**0.01083**	2.86017	2.71133	1.05489	0.33209	2.48761	**0.01286**	2.54809	**0.01083**
308322	69	6	4.00	10.39503	5.20966	1.99534	**0.04979**	47.91467	20.42151	2.34628	0.10064	2.54805	**0.01083**	2.54805	**0.01083**
189960	44	31	4.84	-2.73631	2.94160	-0.93021	0.35537	-3.20956	1.17637	-2.72836	**0.01087**	-2.45442	**0.01411**	-2.54685	**0.01087**
182188	56	19	3.68	7.47559	3.21793	2.32311	**0.02300**	5.54774	3.99784	1.38768	0.18426	2.54640	**0.01088**	2.54640	**0.01088**
4480861	48	27	61.48	7.56556	2.89679	2.61170	**0.01096**	1.05257	1.54711	0.68035	0.50279	2.27275	**0.02304**	2.54405	**0.01096**
3746307	70	5	3.20	14.47322	5.57101	2.59795	**0.01137**	-17.13554	7.44351	-2.30208	0.14794	0.76677	0.44322	2.53124	**0.01137**
4377144	70	5	7.40	12.07259	5.63672	2.14178	**0.03560**	19.11823	8.06383	2.37086	0.14118	2.52644	**0.01152**	2.52644	**0.01152**
538322	45	30	6.60	1.97788	2.95556	0.66921	0.50550	5.09757	1.88707	2.70132	**0.01178**	2.25169	**0.02434**	2.51852	**0.01178**
4483037	54	21	2.67	-8.08739	3.13400	-2.58054	**0.01190**	0.70890	1.39505	0.50815	0.61752	-1.42527	0.15408	-2.51500	**0.01190**
192818	71	4	1.25	1.97686	6.48379	0.30489	0.76133	-13.64865	0.25640	-53.23167	**0.01196**	-1.56245	0.11818	-2.51338	**0.01196**
194371	59	16	3.44	8.73377	3.38712	2.57853	**0.01197**	2.15402	4.51317	0.47727	0.64109	2.10668	**0.03515**	2.51313	**0.01197**
107044	30	45	60.24	-2.35746	2.94709	-0.79993	0.42638	-1.81929	0.69381	-2.62217	**0.01212**	-2.33631	**0.01948**	-2.50865	**0.01212**
1607319	64	11	1.27	-6.56641	4.13925	-1.58638	0.11704	-9.45141	4.05103	-2.33309	**0.04793**	-2.50693	**0.01218**	-2.50693	**0.01218**
187267	53	22	8.50	6.39578	3.14383	2.03439	**0.04560**	4.59906	2.84294	1.61771	0.12221	2.50647	**0.01219**	2.50647	**0.01219**
1000547	51	24	2.75	-6.82274	3.00151	-2.27310	**0.02600**	-2.76388	2.03122	-1.36070	0.18803	-2.50496	**0.01225**	-2.50496	**0.01225**
2840201	65	10	3.90	10.49178	4.08379	2.56913	**0.01227**	-13.83767	7.73605	-1.78873	0.11680	0.66185	0.50807	2.50436	**0.01227**
199182	63	12	4.08	9.90358	3.85704	2.56766	**0.01231**	8.08833	8.86250	0.91265	0.38522	2.38387	**0.01713**	2.50299	**0.01231**
184940	68	7	6.14	12.24342	4.77070	2.56638	**0.01236**	3.61748	6.24903	0.57889	0.59370	2.14626	**0.03185**	2.50179	**0.01236**
188317	68	7	1.57	8.56320	4.94126	1.73300	0.08738	28.30567	11.39574	2.48388	0.06793	2.49954	**0.01244**	2.49954	**0.01244**
187917	40	35	10.09	2.18348	2.90103	0.75265	0.45411	3.79141	1.43198	2.64766	**0.01247**	2.29598	**0.02168**	2.49842	**0.01247**
182167	41	34	9.29	0.47862	2.96528	0.16141	0.87223	4.45152	1.68211	2.64639	**0.01267**	1.87654	0.06058	2.49300	**0.01267**
4333652	67	8	2.13	11.47846	4.49672	2.55263	**0.01281**	8.20165	15.44828	0.53091	0.61821	2.11237	**0.03465**	2.48895	**0.01281**
188764	66	9	1.67	2.69748	4.47432	0.60288	0.54848	28.74267	8.22038	3.49651	**0.01288**	2.18290	**0.02904**	2.48705	**0.01288**
182163	69	6	9.00	13.20879	5.18225	2.54885	**0.01294**	8.48868	7.89073	1.07578	0.36084	2.40359	**0.01623**	2.48542	**0.01294**
3154070	2	73	33.99	-15.99891	8.79779	-1.81851	0.07314	-1.30796	0.74992	-1.74415	0.08552	-2.48295	**0.01303**	-2.48295	**0.01303**
3014082	67	8	3.88	11.67212	4.59329	2.54113	**0.01320**	-3.78206	8.61745	-0.43888	0.67906	1.45979	0.14435	2.47820	**0.01320**
4467447	22	53	49.87	-3.73379	3.15895	-1.18197	0.24111	-1.74478	0.72525	-2.40577	**0.01988**	-2.47549	**0.01331**	-2.47549	**0.01331**
4446902	41	34	7.62	-4.96513	2.85771	-1.73745	0.08658	-2.03441	1.10325	-1.84402	0.07476	-2.47180	**0.01344**	-2.47180	**0.01344**
2920309	51	24	3.21	-3.97219	3.14119	-1.26455	0.21011	-3.15749	1.30947	-2.41128	**0.02514**	-2.46961	**0.01353**	-2.46961	**0.01353**
1566691	69	6	2.33	13.02357	5.16782	2.52013	**0.01395**	-10.67000	18.28895	-0.58341	0.60058	1.36825	0.17123	2.45857	**0.01395**
199279	55	20	1.95	3.50213	3.31541	1.05632	0.29435	9.81330	3.63561	2.69922	**0.01520**	2.45805	**0.01397**	2.45805	**0.01397**
3426658	41	34	2.44	-7.03669	2.79386	-2.51863	**0.01400**	-0.48422	1.35410	-0.35760	0.72307	-1.98805	**0.04681**	-2.45716	**0.01400**
171559	61	14	1.57	-8.08074	3.60556	-2.24119	**0.02810**	-5.16548	3.81768	-1.35304	0.20320	-2.45254	**0.01419**	-2.45254	**0.01419**
3472078	28	47	14.98	-7.21262	2.87659	-2.50735	**0.01442**	-0.66876	0.71172	-0.93964	0.35253	-2.38741	**0.01697**	-2.44662	**0.01442**
266210	69	6	12.50	1.79127	5.42939	0.32992	0.74242	-11.48507	2.24751	-5.11012	**0.01450**	-1.49625	0.13459	-2.44467	**0.01450**
340474	46	29	3.79	-5.12739	3.01990	-1.69787	0.09385	-2.05167	1.10604	-1.85496	0.07497	-2.44379	**0.01453**	-2.44379	**0.01453**
4349261	52	23	26.26	6.53165	3.06303	2.13242	**0.03638**	2.65885	1.88564	1.41005	0.17389	2.44124	**0.01464**	2.44124	**0.01464**
193852	67	8	2.00	11.27475	4.50803	2.50104	**0.01466**	2.86763	13.07623	0.21930	0.83509	1.87304	0.06106	2.44070	**0.01466**

Table S4c. OTUs associated with High-density lipoprotein (HDL) at FDR < 0.05 level [a] (Cont.)

	Summary of OTUs reads			Binary model				Quantitative model				Meta analysis		Final association	
OTU	No. Absent	No. Present	mean counts in presents	Estimate	s.e.	*t* value	*P* value	Estimate	s.e.	*t* value	*P* value	Meta z value	*Meta P* value	*z*	*P value*
184770	50	25	4.28	7.45445	2.99046	2.49274	**0.01498**	-0.04027	2.62548	-0.01534	0.98790	1.70962	0.08734	2.43293	**0.01498**
188333	69	6	3.50	10.82773	5.18823	2.08698	**0.04043**	21.87608	11.93717	1.83260	0.16424	2.43263	**0.01499**	2.43263	**0.01499**
185802	31	44	91.45	5.89708	2.86324	2.05958	**0.04305**	1.29567	0.90151	1.43722	0.15824	2.42833	**0.01517**	2.42833	**0.01517**
172777	28	47	7.26	-6.33421	2.90813	-2.17810	**0.03267**	-1.09183	0.82947	-1.31629	0.19489	-2.42701	**0.01522**	-2.42701	**0.01522**
4480359	28	47	8.09	4.22748	2.95591	1.43018	0.15699	2.83832	1.36692	2.07643	**0.04373**	2.42676	**0.01523**	2.42676	**0.01523**
3265161	34	41	8.24	-4.41657	2.88149	-1.53274	0.12972	-2.37416	1.20472	-1.97071	0.05607	-2.42231	**0.01542**	-2.42231	**0.01542**
306315	64	11	3.18	9.82768	3.97811	2.47044	**0.01586**	0.15485	9.05292	0.01711	0.98677	1.71729	0.08593	2.41203	**0.01586**
4405423	60	15	4.40	-2.36862	3.67357	-0.64477	0.52112	5.79639	2.07330	2.79573	**0.01617**	1.24691	0.21243	2.40501	**0.01617**
845444	61	14	2.64	8.79374	3.57255	2.46147	**0.01623**	-4.70108	4.83064	-0.97318	0.35138	1.04065	0.29804	2.40362	**0.01623**
4202174	40	35	9.69	6.83659	2.79141	2.44915	**0.01675**	-1.45942	1.50997	-0.96652	0.34103	1.01818	0.30859	2.39206	**0.01675**
235212	69	6	44.00	12.63518	5.16029	2.44854	**0.01678**	-2.94776	10.72238	-0.27492	0.80123	1.51301	0.13028	2.39148	**0.01678**
339494	61	14	100.57	8.75624	3.57678	2.44808	**0.01680**	0.09176	2.73103	0.03360	0.97380	1.71395	0.08654	2.39105	**0.01680**
189820	68	7	2.71	2.71259	4.97842	0.54487	0.58753	9.01001	2.28522	3.94273	**0.01692**	2.07242	**0.03823**	2.38842	**0.01692**
179018	54	21	9.76	7.57805	3.11099	2.43590	**0.01733**	-0.83004	2.30205	-0.36057	0.72262	1.43165	0.15225	2.37961	**0.01733**
553611	45	30	9.03	3.86565	2.96297	1.30466	0.19617	-4.16320	1.64272	-2.53433	**0.01738**	-0.76800	0.44249	-2.37867	**0.01738**
4468466	18	57	118.93	1.43663	3.47915	0.41293	0.68089	1.57718	0.64471	2.44635	**0.01772**	1.96767	**0.04911**	2.37145	**0.01772**
198696	71	4	2.75	14.52341	6.21993	2.33498	**0.02234**	34.90798	16.77692	2.08071	0.28521	2.37113	**0.01773**	2.37113	**0.01773**
4405482	59	16	1.56	-6.74376	3.45311	-1.95295	0.05471	-3.80534	2.50841	-1.51703	0.15320	-2.36845	**0.01786**	-2.36845	**0.01786**
2388088	58	17	3.82	-8.13916	3.36848	-2.41627	**0.01822**	-0.62350	2.20203	-0.28315	0.78121	-1.86600	0.06204	-2.36117	**0.01822**
3232988	68	7	11.00	11.38190	4.79824	2.37210	**0.02036**	7.36985	6.33520	1.16332	0.30937	2.35902	**0.01832**	2.35902	**0.01832**
363646	58	17	13.29	-4.78305	3.42089	-1.39819	0.16635	-2.56578	1.21143	-2.11798	0.05255	-2.34944	**0.01880**	-2.34944	**0.01880**
198210	52	23	5.78	3.58029	3.16285	1.13198	0.26140	5.11932	2.16742	2.36194	**0.02843**	2.34361	**0.01910**	2.34361	**0.01910**
43950	67	8	2.38	-3.49443	4.72817	-0.73907	0.46227	12.28831	3.60741	3.40641	**0.01912**	1.13711	0.25549	2.34323	**0.01912**
177697	59	16	2.00	8.19570	3.42562	2.39247	**0.01935**	-5.00641	5.86493	-0.85362	0.40877	1.06966	0.28477	2.33878	**0.01935**
189083	40	35	32.46	6.71275	2.80792	2.39065	**0.01944**	-0.89712	1.31529	-0.68207	0.50010	1.17573	0.23970	2.33707	**0.01944**
4370025	59	16	7.63	-6.99729	3.53484	-1.97952	0.05158	3.03951	1.14141	2.66295	**0.01952**	0.27489	0.78340	2.33536	**0.01952**
4444277	64	11	102.55	9.39930	3.94447	2.38291	**0.01982**	-3.59030	1.99628	-1.79849	0.10981	0.51669	0.60537	2.32978	**0.01982**
177518	62	13	4.23	6.72113	3.74592	1.79425	0.07697	-11.83318	4.27767	-2.76627	**0.01991**	-0.39557	0.69242	-2.32796	**0.01991**
189937	59	16	2.38	-3.70750	3.56405	-1.04025	0.30171	-7.70806	3.01890	-2.55327	**0.02404**	-2.32583	**0.02003**	-2.32583	**0.02003**
178759	64	11	12.27	9.38274	3.94826	2.37643	**0.02014**	0.66146	4.60866	0.14353	0.88942	1.74140	0.08161	2.32368	**0.02014**
195214	66	9	3.44	9.25278	4.37167	2.11653	**0.03776**	9.80179	7.29770	1.34313	0.22780	2.32180	**0.02024**	2.32180	**0.02024**
2403301	57	18	52.33	7.85916	3.31846	2.36832	**0.02056**	1.24943	1.81053	0.69009	0.50068	2.11387	**0.03453**	2.31604	**0.02056**
1602805	48	27	5.70	-4.35234	2.97749	-1.46175	0.14816	-2.60140	1.36018	-1.91254	0.06781	-2.31387	**0.02068**	-2.31387	**0.02068**
175184	64	11	2.55	4.18140	4.12754	1.01305	0.31443	9.92349	3.55798	2.78908	**0.02359**	2.31201	**0.02078**	2.31201	**0.02078**
191043	70	5	4.20	-6.37491	5.78074	-1.10278	0.27379	13.11592	1.92312	6.82014	**0.02083**	0.86033	0.38961	2.31106	**0.02083**
182797	69	6	2.83	12.39385	5.24864	2.36135	**0.02092**	-23.37186	31.27050	-0.74741	0.50907	1.16615	0.24356	2.30947	**0.02092**
190676	61	14	24.79	8.58277	3.64596	2.35405	**0.02130**	-4.57878	2.91270	-1.57201	0.14425	0.59570	0.55138	2.30259	**0.02130**
213394	59	16	41.63	-6.66359	3.48260	-1.91340	0.05967	-0.88872	0.61335	-1.44895	0.17104	-2.29956	**0.02147**	-2.29956	**0.02147**
579541	63	12	7.67	8.97303	3.82887	2.34352	**0.02187**	-5.93500	5.81260	-1.02106	0.33389	0.93787	0.34831	2.29266	**0.02187**
3754778	68	7	18.86	11.27637	4.82182	2.33861	**0.02214**	1.56475	6.82810	0.22916	0.82998	1.76972	0.07677	2.28803	**0.02214**
195015	63	12	3.33	-3.88252	3.92690	-0.98870	0.32612	4.20029	1.52415	2.75582	**0.02226**	0.92198	0.35654	2.28584	**0.02226**
586453	64	11	7.36	-8.07804	3.99195	-2.02358	**0.04673**	-1.87272	1.39493	-1.34251	0.21628	-2.28061	**0.02257**	-2.28061	**0.02257**
864465	51	24	3.08	-1.79239	3.17444	-0.56463	0.57408	-5.91891	2.40542	-2.46066	**0.02262**	-2.00954	**0.04448**	-2.27986	**0.02262**
59563	66	9	17.89	5.66046	4.41243	1.28284	0.20366	3.61265	1.48528	2.43231	0.05100	2.27876	**0.02268**	2.27876	**0.02268**
157162	70	5	3.00	13.04286	5.60318	2.32776	**0.02274**	21.12212	20.42321	1.03422	0.40970	2.19359	**0.02826**	2.27779	**0.02274**
175612	70	5	65.20	-2.97478	6.02417	-0.49381	0.62295	-4.30214	0.66853	-6.43522	**0.02331**	-1.95165	0.05098	-2.26837	**0.02331**
2203165	69	6	2.67	-9.31685	5.39010	-1.72851	0.08818	8.65283	2.01919	4.28529	**0.02335**	0.39779	0.69079	2.26762	**0.02335**
188735	30	45	7.40	-6.71530	2.89885	-2.31654	**0.02338**	0.21021	1.08517	0.19371	0.84733	-1.46702	0.14237	-2.26720	**0.02338**
173965	60	15	2.40	-1.34340	3.62099	-0.37100	0.71172	6.70568	2.59112	2.58794	**0.02375**	1.33759	0.18103	2.26119	**0.02375**
4318284	69	6	28.83	-10.39242	5.20328	-1.99728	**0.04957**	-1.39834	0.89926	-1.55499	0.21779	-2.25994	**0.02382**	-2.25994	**0.02382**
177520	44	31	6.06	3.11699	2.93072	1.06356	0.29108	4.85956	2.15941	2.25041	**0.03246**	2.25878	**0.02390**	2.25878	**0.02390**
184990	66	9	4.89	9.98163	4.32606	2.30732	**0.02392**	-2.51763	6.97759	-0.36082	0.73060	1.35352	0.17589	2.25849	**0.02392**
193161	71	4	1.25	14.31682	6.22947	2.29824	**0.02445**	-111.51370	119.01938	-0.93694	0.52072	1.13680	0.25562	2.24991	**0.02445**
4302904	51	24	5.67	-3.75655	3.11089	-1.20755	0.23117	-3.22354	1.53170	-2.10454	**0.04755**	-2.24769	**0.02460**	-2.24769	**0.02460**
4414420	71	4	1.75	-5.06823	6.44214	-0.78673	0.43402	-5.17015	0.20021	-25.82325	**0.02464**	-2.14205	**0.03219**	-2.24699	**0.02464**
366147	62	13	6.31	-5.06882	3.80567	-1.33191	0.18709	-3.45260	1.65350	-2.08806	0.06334	-2.24582	**0.02472**	-2.24582	**0.02472**
184426	46	29	5.07	3.85419	2.95914	1.30247	0.19691	3.85344	1.96402	1.96202	0.06055	2.23955	**0.02512**	2.23955	**0.02512**
843569	69	6	10.83	11.89433	5.20935	2.28326	**0.02537**	-9.84103	4.74599	-2.07355	0.12980	0.50973	0.61024	2.23575	**0.02537**
194660	47	28	5.07	5.08739	2.93628	1.73260	0.08745	4.02719	2.69654	1.49347	0.14783	2.23181	**0.02563**	2.23181	**0.02563**
4332078	59	16	3.81	7.84483	3.44234	2.27893	**0.02564**	-6.71919	5.40126	-1.24400	0.23546	0.73910	0.45985	2.23165	**0.02564**
577294	65	10	2.10	-9.41964	4.14054	-2.27498	**0.02589**	0.95464	2.75436	0.34659	0.73908	-1.33986	0.18029	-2.22791	**0.02589**
186876	70	5	2.40	-1.56945	5.84146	-0.26867	0.78895	11.11952	1.82586	6.09001	**0.02592**	1.38575	0.16582	2.22742	**0.02592**
336281	67	8	50.00	10.35414	4.55428	2.27350	**0.02598**	-0.19698	3.63849	-0.05414	0.95892	1.53796	0.12406	2.22651	**0.02598**
182431	52	23	7.65	4.23692	3.13233	1.35264	0.18040	4.55016	2.38077	1.91122	0.07042	2.22650	**0.02598**	2.22650	**0.02598**
4332428	71	4	2.00	14.18813	6.25496	2.26830	**0.02631**	6.03493	125.71564	0.04800	0.96946	1.59797	0.11005	2.22159	**0.02631**
304973	62	13	3.31	-1.05887	3.97503	-0.26638	0.79071	-6.72079	2.58204	-2.60290	**0.02636**	-1.75805	0.07874	-2.22087	**0.02636**
4423553	37	38	12.08	5.50381	2.83890	1.93871	0.05646	2.05349	1.63508	1.25589	0.21747	2.22086	**0.02636**	2.22086	**0.02636**
181140	59	16	3.06	3.30982	3.53039	0.93752	0.35162	11.02287	4.45123	2.47636	**0.02780**	2.21434	**0.02681**	2.21434	**0.02681**
849440	70	5	1.80	5.15182	5.81277	0.88629	0.37841	15.16582	2.54595	5.95684	**0.02704**	2.18617	**0.02880**	2.21089	**0.02704**
153075	67	8	10.50	10.25088	4.54887	2.25350	**0.02727**	-0.17991	6.21917	-0.02893	0.97804	1.54153	0.12319	2.20758	**0.02727**
319411	71	4	2.25	11.01250	6.35162	1.73381	0.08723	20.98350	5.35173	3.92088	0.15898	2.20526	**0.02744**	2.20526	**0.02744**
34789	63	12	3.83	7.68997	3.86934	1.98741	0.05068	8.67432	6.99649	1.23981	0.24640	2.20145	**0.02770**	2.20145	**0.02770**
4450214	31	44	39.32	4.68731	2.94613	1.59101	0.11599	1.71498	1.09391	1.56776	0.12462	2.19732	**0.02800**	2.19732	**0.02800**
4381749	55	20	4.10	2.74327	3.26430	0.84039	0.40347	6.94677	2.89176	2.40226	**0.02800**	2.14448	**0.03199**	2.19732	**0.02800**
183454	67	8	15.75	10.24523	4.57518	2.23931	**0.02823**	-6.00090	5.39044	-1.11325	0.31624	0.84281	0.39933	2.19414	**0.02823**
181754	49	26	4.00	3.33480	3.05852	1.09033	0.27920	4.15113	1.94904	2.12983	**0.04411**	2.18863	**0.02862**	2.18863	**0.02862**
4470870	51	24	78.92	0.21710	3.13909	0.06916	0.94505	1.96145	0.83665	2.34442	**0.02897**	1.59300	0.11116	2.18393	**0.02897**

Table S4c. OTUs associated with High-density lipoprotein (HDL) at FDR < 0.05 [a] level (Cont.)

	Summary of OTUs reads			Binary model				Quantitative model				Meta analysis		Final association	
OTU	No. Absent	No. Present	mean counts in presents	Estimate	s.e.	*t* value	*P* value	Estimate	s.e.	*t* value	*P* value	Meta z value	*Meta P* value	*z*	*P value*
3756485	44	31	5.19	3.50648	2.94608	1.19022	0.23787	4.55065	2.29809	1.98019	0.05759	2.17730	**0.02946**	2.17730	**0.02946**
807548	70	5	5.60	10.68571	5.67208	1.88392	0.06361	21.58441	12.29857	1.75503	0.22134	2.17636	**0.02953**	2.17636	**0.02953**
184209	50	25	2.80	-6.61241	2.97864	-2.21994	**0.02957**	-1.79854	2.07964	-0.86483	0.39646	-2.13810	**0.03251**	-2.17578	**0.02957**
307984	65	10	3.30	-2.93791	4.34048	-0.67686	0.50066	5.97263	2.19926	2.71574	**0.02995**	1.05876	0.28971	2.17076	**0.02995**
4300127	65	10	324.60	7.80503	4.16574	1.87362	0.06504	2.54212	1.90217	1.33643	0.22321	2.16587	**0.03032**	2.16587	**0.03032**
157631	71	4	11.25	13.79636	6.26213	2.20314	**0.03079**	-16.73865	4.72058	-3.54589	0.17499	0.56816	0.56992	2.15984	**0.03079**
4365130	16	59	116.85	-7.64795	3.47312	-2.20204	**0.03087**	-0.35028	0.56412	-0.62094	0.53716	-1.96286	**0.04966**	-2.15879	**0.03087**
4212012	64	11	1.18	8.85015	4.02317	2.19980	**0.03103**	-6.41594	17.53517	-0.36589	0.72393	1.27523	0.20223	2.15666	**0.03103**
288521	63	12	8.83	-8.42247	3.83119	-2.19840	**0.03114**	1.67747	1.99684	0.84006	0.42263	-0.95704	0.33855	-2.15533	**0.03114**
192437	26	49	16.29	6.47436	2.94865	2.19570	**0.03134**	0.27846	1.31687	0.21146	0.83346	1.67092	0.09474	2.15277	**0.03134**
299441	69	6	9.83	8.95229	5.28125	1.69511	0.09438	5.40630	3.01901	1.79075	0.17126	2.15025	**0.03154**	2.15025	**0.03154**
362765	46	29	4.90	-4.11079	2.95822	-1.38962	0.16893	-2.09731	1.21699	-1.72335	0.09670	-2.14729	**0.03177**	-2.14729	**0.03177**
182735	59	16	4.38	-4.92118	3.61829	-1.36009	0.17805	-3.34295	1.83720	-1.81959	0.09192	-2.14406	**0.03203**	-2.14406	**0.03203**
2018038	61	14	3.21	7.87091	3.60415	2.18384	**0.03223**	-1.37558	5.22348	-0.26335	0.79715	1.33253	0.18269	2.14151	**0.03223**
840376	64	11	17.09	-2.23961	4.08825	-0.54782	0.58551	-4.97413	1.93267	-2.57370	**0.03294**	-1.89377	0.05826	-2.13285	**0.03294**
176753	68	7	3.14	0.59852	4.99631	0.11979	0.90498	10.31550	3.22550	3.19811	**0.03296**	1.59236	0.11130	2.13257	**0.03296**
175485	11	64	27.86	0.24025	4.12938	0.05818	0.95377	-1.87797	0.86189	-2.17891	**0.03321**	-1.46480	0.14297	-2.12952	**0.03321**
199286	40	35	9.26	1.84293	2.91521	0.63218	0.52927	3.36741	1.51428	2.22377	**0.03335**	1.94950	0.05124	2.12790	**0.03335**
19080	62	13	64.00	-4.49425	3.87853	-1.15875	0.25039	0.88311	0.35854	2.46310	**0.03350**	0.69060	0.48982	2.12606	**0.03350**
312882	57	18	4.50	-3.51069	3.41423	-1.02825	0.30727	3.88645	1.66078	2.34014	**0.03351**	0.78129	0.43463	2.12587	**0.03351**
1820776	64	11	40.09	4.86953	4.06504	1.19790	0.23488	6.64395	3.16643	2.09825	0.06913	2.12519	**0.03357**	2.12519	**0.03357**
3211875	25	50	7.50	-2.71437	3.06223	-0.88640	0.37835	-2.46462	1.12838	-2.18422	**0.03397**	-2.12229	**0.03381**	-2.12229	**0.03381**
186133	60	15	12.33	1.90868	3.61746	0.52763	0.59938	5.41269	2.26614	2.38850	**0.03423**	1.86864	0.06167	2.11737	**0.03423**
3235048	48	27	23.07	4.08237	2.99485	1.36313	0.17709	3.05976	1.79149	1.70794	0.10055	2.11563	**0.03438**	2.11563	**0.03438**
341730	67	8	2.50	-5.83037	4.64928	-1.25404	0.21388	-5.95694	2.72343	-2.18730	0.08037	-2.11531	**0.03440**	-2.11531	**0.03440**
184464	45	30	150.53	6.18295	2.87525	2.15041	**0.03488**	-0.34095	0.96056	-0.35494	0.72539	1.24342	0.21371	2.10973	**0.03488**
182874	55	20	43.10	3.28269	3.25793	1.00760	0.31702	-3.67520	1.60323	-2.29237	**0.03491**	-0.78400	0.43304	-2.10935	**0.03491**
183970	69	6	4.67	11.17740	5.20973	2.14548	**0.03529**	2.74858	10.32287	0.26626	0.80729	1.66097	0.09672	2.10505	**0.03529**
3186216	63	12	1.67	8.26326	3.85407	2.14404	**0.03541**	-9.06866	10.26657	-0.88332	0.40004	0.89246	0.37215	2.10367	**0.03541**
1943669	69	6	1.33	0.78150	5.35306	0.14599	0.88434	16.33430	4.50046	3.62947	**0.03601**	1.58556	0.11284	2.09685	**0.03601**
180216	56	19	47.32	-4.91385	3.34063	-1.47094	0.14567	2.00308	0.87643	2.28551	**0.03626**	0.45181	0.65141	2.09397	**0.03626**
363321	67	8	5.63	-1.28274	4.69365	-0.27329	0.78541	7.75375	2.73122	2.83893	**0.03629**	1.28790	0.19778	2.09364	**0.03629**
306704	70	5	8.60	2.81252	5.83498	0.48201	0.63126	-6.91384	1.35528	-5.10141	**0.03634**	-1.14064	0.25402	-2.09306	**0.03634**
4458576	23	52	72.92	-0.85770	3.16027	-0.27140	0.78686	-1.88586	0.87762	-2.14882	**0.03662**	-1.66906	0.09510	-2.09002	**0.03662**
4256470	6	69	44.28	-9.04712	5.24848	-1.72376	0.08904	-0.84505	0.67028	-1.26073	0.21184	-2.08524	**0.03705**	-2.08524	**0.03705**
193654	59	16	4.25	-1.42912	3.56035	-0.40140	0.68932	-5.63856	2.43016	-2.32024	**0.03723**	-1.75574	0.07913	-2.08321	**0.03723**
4060501	46	29	13.69	-6.15238	2.90154	-2.12039	**0.03742**	0.56037	1.20861	0.46365	0.64676	-1.14755	0.25115	-2.08116	**0.03742**
358798	51	24	5.58	-0.17732	3.20076	-0.05540	0.95597	4.98157	2.24645	2.21753	**0.03775**	1.43001	0.15271	2.07755	**0.03775**
2438203	43	32	7.66	-3.35223	2.90786	-1.15282	0.25280	-2.90890	1.56535	-1.85831	0.07330	-2.07502	**0.03799**	-2.07502	**0.03799**
858999	70	5	2.20	11.14017	5.66006	1.96821	0.05290	12.59902	9.55762	1.31822	0.31816	2.07467	**0.03802**	2.07467	**0.03802**
3856408	60	15	2.47	-0.64267	3.66249	-0.17547	0.86120	-5.19396	2.22862	-2.33057	**0.03803**	-1.59054	0.11171	-2.07452	**0.03803**
356745	35	40	7.15	3.71726	2.88326	1.28926	0.20144	3.06995	1.80846	1.69755	0.09799	2.07335	**0.03814**	2.07335	**0.03814**
301253	40	35	35.94	5.95000	2.81920	2.11053	**0.03829**	-0.77370	1.47576	-0.52427	0.60370	1.09791	0.27224	2.07177	**0.03829**
176104	38	37	14.35	1.22775	2.92650	0.41953	0.67608	3.30555	1.53556	2.15267	**0.03853**	1.75858	0.07865	2.06918	**0.03853**
174885	68	7	2.86	-7.13107	4.91203	-1.45176	0.15091	-3.83259	2.06005	-1.86044	0.13633	-2.06893	**0.03855**	-2.06893	**0.03855**
4329572	69	6	3.00	-10.91623	5.18565	-2.10508	**0.03877**	1.09123	2.89389	0.37708	0.73121	-1.21839	0.22308	-2.06658	**0.03877**
1646171	59	16	7.25	-7.22280	3.43481	-2.10282	**0.03898**	-1.05312	1.47800	-0.71253	0.48873	-1.94932	0.05126	-2.06442	**0.03898**
2953981	67	8	3.50	-9.55658	4.55855	-2.09641	**0.03956**	-1.22582	2.44128	-0.50212	0.63691	-1.78922	0.07358	-2.05831	**0.03956**
4438999	61	14	3.93	-2.03534	3.71226	-0.54828	0.58520	-4.66480	1.99923	-2.33330	**0.03964**	-1.84082	0.06565	-2.05750	**0.03964**
180107	61	14	3.93	-2.03534	3.71226	-0.54828	0.58520	-4.66480	1.99923	-2.33330	**0.03964**	-1.84082	0.06565	-2.05750	**0.03964**
555547	59	16	3.56	7.18998	3.43505	2.09312	**0.03986**	-2.14329	4.48764	-0.47760	0.64087	1.12337	0.26128	2.05518	**0.03986**
332732	47	28	16.50	-3.84262	2.96741	-1.29494	0.19948	1.69724	0.78310	2.16733	**0.03994**	0.54543	0.58546	2.05439	**0.03994**
206513	51	24	5.46	4.89791	3.05656	1.60242	0.11344	3.86221	2.83274	1.36342	0.18719	2.05192	**0.04018**	2.05192	**0.04018**
365717	62	13	3.69	-7.81546	3.74338	-2.08781	**0.04035**	3.28889	3.13608	1.04873	0.31899	-0.74499	0.45628	-2.05011	**0.04035**
4428313	48	27	160.15	0.37137	3.01981	0.12298	0.90247	-1.35312	0.62484	-2.16555	**0.04051**	-1.36190	0.17323	-2.04856	**0.04051**
355187	69	6	3.50	10.77307	5.19229	2.07482	**0.04158**	-4.19572	16.95523	-0.24746	0.82052	1.28046	0.20038	2.03772	**0.04158**
3186388	60	15	1.80	-7.29929	3.52238	-2.07226	**0.04182**	-0.91401	3.36567	-0.27157	0.79057	-1.62695	0.10375	-2.03528	**0.04182**
4379646	67	8	1.75	-9.45478	4.56467	-2.07130	**0.04192**	7.40625	15.61294	0.47437	0.65523	-1.12278	0.26153	-2.03436	**0.04192**
186624	68	7	1.14	-0.68673	4.98347	-0.13780	0.89078	34.09677	11.55594	2.95059	**0.04194**	1.34121	0.17985	2.03407	**0.04194**
725212	65	10	5.30	8.61283	4.16050	2.07014	**0.04203**	0.45977	3.07565	0.14949	0.88538	1.53966	0.12364	2.03326	**0.04203**
175642	71	4	3.25	6.76047	6.42130	1.05282	0.29594	68.61899	7.28972	9.41312	0.06738	2.03245	**0.04211**	2.03245	**0.04211**
4336939	61	14	9.07	7.52354	3.63754	2.06830	**0.04220**	3.54790	4.51954	0.78501	0.44902	1.97180	**0.04863**	2.03150	**0.04220**
192347	61	14	1.57	6.76390	3.71209	1.82213	0.07259	9.54382	8.43351	1.13165	0.28185	2.03052	**0.04230**	2.03052	**0.04230**
174831	43	32	34.69	5.89301	2.85264	2.06581	**0.04245**	-1.12526	1.26725	-0.88796	0.38187	0.81647	0.41423	2.02912	**0.04245**
308498	63	12	12.08	6.06188	3.88825	1.55902	0.12338	4.91918	3.41830	1.43907	0.18399	2.02893	**0.04247**	2.02893	**0.04247**
4473509	24	51	20.41	6.23480	3.02107	2.06377	**0.04264**	0.13949	1.14814	0.12150	0.90381	1.51889	0.12879	2.02718	**0.04264**
1945397	66	9	9.00	8.94714	4.33622	2.06335	**0.04269**	0.77865	9.37038	0.08310	0.93648	1.48950	0.13636	2.02677	**0.04269**
4453501	27	48	37.50	-6.24747	3.02889	-2.06263	**0.04276**	0.53813	0.61427	0.87604	0.38566	-0.81923	0.41265	-2.02608	**0.04276**
571642	40	35	5.00	4.43445	2.96275	1.49674	0.13883	3.04217	2.16463	1.40540	0.16954	2.01797	**0.04359**	2.01797	**0.04359**
192244	58	17	5.65	2.98394	3.46416	0.86138	0.39189	6.29280	2.87626	2.18785	**0.04614**	2.01548	**0.04385**	2.01548	**0.04385**
4338990	54	21	11.48	3.12418	3.21045	0.97313	0.33375	-4.52447	2.08919	-2.16566	**0.04401**	-0.74062	0.45893	-2.01398	**0.04401**
4469167	64	11	1.64	8.20917	4.00959	2.04738	**0.04427**	-2.31965	11.48733	-0.20193	0.84501	1.28413	0.19910	2.01153	**0.04427**
1136387	67	8	15.25	-4.62636	4.67216	-0.99020	0.32539	-2.71062	1.13746	-2.38304	0.06293	-2.01040	**0.04439**	-2.01040	**0.04439**
4480176	69	6	7.67	7.87826	5.26231	1.49711	0.13874	10.32855	5.82403	1.77344	0.17426	2.00758	**0.04469**	2.00758	**0.04469**
216111	47	28	4.11	-6.06107	2.96640	-2.04324	**0.04469**	0.78375	1.77941	0.44046	0.66339	-1.11181	0.26622	-2.00757	**0.04469**
589329	67	8	1.63	4.77580	4.66156	1.02451	0.30903	8.16097	3.53207	2.31053	0.06886	2.00578	**0.04488**	2.00578	**0.04488**
352529	59	16	5.94	-4.12452	3.60804	-1.14315	0.25676	-3.43329	1.87119	-1.83482	0.08951	-2.00256	**0.04522**	-2.00256	**0.04522**

Table S4c. OTUs associated with High-density lipoprotein (HDL) at FDR < 0.05 level [a] (Cont.)

	Summary of OTUs reads			Binary model				Quantitative model				Meta analysis		Final association	
OTU	No. Absent	No. Present	mean counts in presents	Estimate	s.e.	*t* value	*P* value	Estimate	s.e.	*t* value	*P* value	Meta z value	*Meta P* value	*z*	*P value*
327209	67	8	3.00	8.80193	4.59939	1.91372	0.05963	8.58688	8.19939	1.04726	0.34294	2.00244	**0.04524**	2.00244	**0.04524**
2058521	60	15	3.13	-4.81946	3.57931	-1.34648	0.18237	5.28382	2.36585	2.23337	**0.04534**	0.47238	0.63666	2.00153	**0.04534**
180523	65	10	1.20	8.44456	4.14709	2.03626	**0.04540**	0.07714	22.53905	0.00342	0.99736	1.41719	0.15643	2.00090	**0.04540**
4413347	71	4	2.00	2.15577	6.51441	0.33092	0.74166	25.95507	1.85530	13.98967	**0.04543**	1.64778	0.09940	2.00066	**0.04543**
3141094	37	38	3.21	-3.65062	2.86679	-1.27342	0.20697	-2.61438	1.62843	-1.60546	0.11738	-1.99958	**0.04555**	-1.99958	**0.04555**
4427290	43	32	17.88	-5.83634	2.86984	-2.03369	**0.04567**	-0.53660	0.86273	-0.62198	0.53881	-1.84770	0.06465	-1.99844	**0.04567**
576400	69	6	2.67	10.62105	5.22295	2.03354	**0.04568**	-10.45265	31.45136	-0.33234	0.76150	1.19839	0.23077	1.99829	**0.04568**
196597	70	5	2.40	11.53299	5.67213	2.03327	**0.04571**	-37.42343	51.70579	-0.72378	0.54441	0.98421	0.32501	1.99804	**0.04571**
553080	28	47	17.00	1.81997	3.00434	0.60578	0.54656	2.66477	1.29666	2.05510	**0.04584**	1.83835	0.06601	1.99691	**0.04584**
111771	68	7	2.29	1.41162	4.98264	0.28331	0.77775	6.60811	2.30920	2.86164	**0.04585**	1.61149	0.10707	1.99674	**0.04585**
287790	54	21	33.05	3.12276	3.21565	0.97111	0.33474	3.20585	1.61853	1.98071	0.06311	1.99615	**0.04592**	1.99615	**0.04592**
192983	44	31	4.39	4.66682	2.96916	1.57176	0.12039	3.14849	2.43449	1.29329	0.20648	1.99153	**0.04642**	1.99153	**0.04642**
308386	51	24	81.96	3.55190	3.10337	1.14453	0.25619	2.44311	1.38897	1.75894	0.09315	1.99011	**0.04658**	1.99011	**0.04658**
310380	70	5	4.00	11.69708	5.77964	2.02384	**0.04670**	51.85262	71.09571	0.72934	0.54165	1.83802	0.06606	1.98903	**0.04670**
215097	70	5	17.80	2.22721	5.87566	0.37906	0.70576	-4.73434	1.06205	-4.45772	**0.04682**	-1.13872	0.25482	-1.98794	**0.04682**
265871	7	68	161.65	9.81761	4.85603	2.02174	**0.04692**	-0.50177	0.70003	-0.71678	0.47608	0.90113	0.36752	1.98701	**0.04692**
183030	65	10	4.90	8.38265	4.15012	2.01986	**0.04712**	-1.63515	2.42654	-0.67386	0.52202	0.95105	0.34158	1.98522	**0.04712**
656881	54	21	8.67	-0.97245	3.29060	-0.29552	0.76844	2.38835	1.12297	2.12681	**0.04752**	1.19304	0.23285	1.98162	**0.04752**
1061772	62	13	2.46	-1.60995	3.82460	-0.42095	0.67505	8.09224	3.58647	2.25632	**0.04767**	1.10387	0.26965	1.98034	**0.04767**
3887769	55	20	1.95	-1.89495	3.30108	-0.57404	0.56773	-4.32207	2.02493	-2.13443	**0.04767**	-1.80435	0.07118	-1.98034	**0.04767**
197890	60	15	4.20	-7.12737	3.54066	-2.01301	**0.04785**	-1.24207	2.20561	-0.56314	0.58371	-1.78660	0.07400	-1.97866	**0.04785**
2831841	54	21	3.67	-6.32698	3.14688	-2.01056	**0.04812**	0.61574	1.12027	0.54963	0.58933	-1.01577	0.30974	-1.97632	**0.04812**
209845	63	12	5.67	-2.28564	4.02686	-0.56760	0.57207	-4.14822	1.82028	-2.27889	**0.04865**	-1.79367	0.07287	-1.97164	**0.04865**
302333	63	12	2.50	-1.09751	3.96222	-0.27699	0.78258	7.15892	3.14145	2.27886	**0.04865**	1.19901	0.23052	1.97162	**0.04865**
110192	60	15	20.60	7.08739	3.53528	2.00476	**0.04875**	-1.42531	2.67173	-0.53348	0.60344	1.02624	0.30478	1.97078	**0.04875**
180421	62	13	3.85	-7.57263	3.77831	-2.00423	**0.04881**	-0.67186	3.63115	-0.18503	0.85691	-1.52069	0.12834	-1.97027	**0.04881**

[a] Statistics associated to the two part model for association analysis between the trait and OTUs adjusting for age and gender. The binomial analysis that tests for association of detecting a microbe represented by an OTU with the trait, where s.e. represents the standard error of the estimate, *t* -value of a *t* test for the estimate and its associated probability (*P* value). The quantitative analysis tests for association between the trait and the abundance of the OTU for the subjects where that OTU is present, where s.e. represents the standard error of the estimate, *t* -value of a *t* test for the estimate and its associated probability (*P* value). The Meta analysis combine the effect of both binary and quantitative analysis, a meta *P* value was derived using an unweighted Z method. The final association *P* value per OTU-trait pair was assigned from the minimum of *P* values from the binary analysis, quantitative analysis, and meta-analysis.

Table S4d. OTUs associated with Low-density lipoprotein (LDL) at FDR < 0.05 level [a]

	Summary of OTUs reads			Binary model				Quantitative model				Meta analysis		Final association	
OTU	No. Absent	No. Present	mean counts in presents	Estimate	s.e.	*t* value	*P* value	Estimate	s.e.	*t* value	*P* value	Meta z value	*Meta P* value	*z*	*P value*
4342104	59	16	5.38	-0.88834	5.95581	-0.14916	0.88185	-10.98124	1.92748	-5.69721	**0.00007**	-2.90906	**0.00363**	-3.96541	**0.00007**
178064	41	34	11.62	0.80299	4.75263	0.16896	0.86630	-8.93219	2.02240	-4.41663	**0.00011**	-2.61038	**0.00904**	-3.85998	**0.00011**
740158	66	9	3.44	26.12882	6.60018	3.95880	**0.00017**	-2.83192	5.21451	-0.54308	0.60664	2.28951	**0.02205**	3.75273	**0.00017**
187946	70	5	1.40	21.37459	9.20398	2.32232	**0.02305**	14.78806	0.79246	18.66102	**0.00286**	3.71594	**0.00020**	3.71594	**0.00020**
324015	70	5	2.40	-33.43605	8.70734	-3.83999	**0.00026**	-32.34373	25.44928	-1.27091	0.33158	-3.26755	**0.00108**	-3.65008	**0.00026**
187517	61	14	14.14	21.06695	5.54269	3.80086	**0.00030**	3.09742	4.55291	0.68031	0.51037	3.02243	**0.00251**	3.61610	**0.00030**
311947	52	23	38.52	17.28304	4.71044	3.66909	**0.00046**	-1.26185	2.79722	-0.45111	0.65676	2.16137	**0.03067**	3.50103	**0.00046**
194374	70	5	2.00	-21.57047	9.17216	-2.35173	**0.02143**	-39.12257	4.58833	-8.52654	**0.01348**	-3.37382	**0.00074**	-3.37382	**0.00074**
851733	59	16	70.75	13.90437	5.54136	2.50920	**0.01435**	3.86261	1.49837	2.57787	**0.02295**	3.33940	**0.00084**	3.33940	**0.00084**
4484075	38	37	4.03	15.18892	4.39600	3.45517	**0.00093**	-4.38594	2.97841	-1.47258	0.15006	1.32430	0.18540	3.31216	**0.00093**
315982	71	4	2.25	33.30952	9.80708	3.39648	**0.00111**	-13.56585	11.28947	-1.20164	0.44186	1.76129	0.07819	3.25990	**0.00111**
4416614	30	45	21.09	5.63321	4.78478	1.17732	0.24295	-6.17669	1.77187	-3.48597	**0.00116**	-1.47104	0.14128	-3.24802	**0.00116**
4388068	69	6	18.83	24.17466	8.25996	2.92673	**0.00458**	6.07410	2.35206	2.58246	0.08161	3.23611	**0.00121**	3.23611	**0.00121**
3946926	69	6	3.17	27.29196	8.19991	3.32832	**0.00138**	10.96050	10.29749	1.06439	0.36523	2.90228	**0.00370**	3.19899	**0.00138**
329798	65	10	5.80	21.11079	6.50161	3.24701	**0.00177**	-0.38698	4.84459	-0.07988	0.93857	2.15591	**0.03109**	3.12599	**0.00177**
275237	42	33	229.64	10.86722	4.59273	2.36618	**0.02067**	2.38419	1.12840	2.11289	**0.04304**	3.06696	**0.00216**	3.06696	**0.00216**
4398588	63	12	4.00	19.20976	6.06397	3.16785	**0.00225**	-3.45409	5.47678	-0.63068	0.54393	1.73079	0.08349	3.05459	**0.00225**
522582	61	14	27.86	17.89411	5.69572	3.14168	**0.00244**	-0.76225	3.16630	-0.24074	0.81419	1.97698	**0.04804**	3.03091	**0.00244**
303320	70	5	2.40	26.02100	9.16116	2.84036	**0.00586**	7.45560	3.14542	2.37031	0.14124	2.98893	**0.00280**	2.98893	**0.00280**
190453	60	15	17.07	11.63798	5.88280	1.97831	0.05172	5.53893	2.13016	2.60024	**0.02322**	2.98068	**0.00288**	2.98068	**0.00288**
196957	22	53	9.45	-9.00398	5.09603	-1.76686	0.08149	-4.76128	1.87110	-2.54464	**0.01407**	-2.96808	**0.00300**	-2.96808	**0.00300**
188262	71	4	1.00	30.99613	10.29887	3.00966	**0.00360**	0.00000	0.00000	0.00000	1.00000	2.05834	**0.03956**	2.91093	**0.00360**
290284	69	6	3.50	13.77370	8.60691	1.60031	0.11391	38.07767	7.02055	5.42374	**0.01230**	2.88807	**0.00388**	2.88807	**0.00388**
3907189	67	8	1.88	21.43065	7.26824	2.94853	**0.00430**	-16.40488	10.31099	-1.59101	0.17248	1.05416	0.29181	2.85508	**0.00430**
180421	62	13	3.85	-17.56717	5.99120	-2.93216	**0.00451**	1.18999	6.79918	0.17502	0.86456	-1.88763	0.05908	-2.84009	**0.00451**
3325758	28	47	17.45	4.14986	4.86838	0.85241	0.39681	-4.58239	1.53114	-2.99279	**0.00452**	-1.40858	0.15896	-2.83935	**0.00452**
157424	48	27	265.70	-7.77339	4.86883	-1.59656	0.11474	3.18072	1.01550	3.13216	**0.00452**	0.89228	0.37224	2.83911	**0.00452**
182647	63	12	29.67	-17.84602	6.11716	-2.91737	**0.00471**	0.03623	2.51661	0.01439	0.98883	-1.98876	**0.04673**	-2.82653	**0.00471**
166689	69	6	4.00	23.98735	8.27182	2.89989	**0.00495**	-5.87308	11.00322	-0.53376	0.63052	1.64721	0.09951	2.81050	**0.00495**
4309301	13	62	21.26	17.11552	5.92146	2.89042	**0.00508**	-2.11228	1.39001	-1.51961	0.13395	0.92143	0.35682	2.80181	**0.00508**
4483570	71	4	5.25	-28.91487	10.00572	-2.88984	**0.00509**	-4.38204	13.78066	-0.31798	0.80400	-2.15628	**0.03106**	-2.80127	**0.00509**
198754	70	5	2.80	-25.07785	9.01543	-2.78166	**0.00690**	-15.66293	8.53552	-1.83503	0.20793	-2.80081	**0.00510**	-2.80081	**0.00510**
192462	20	55	44.05	14.55947	5.07011	2.87163	**0.00536**	-0.22045	1.35137	-0.16313	0.87105	1.85419	0.06371	2.78455	**0.00536**
232900	67	8	5.38	-20.84384	7.26226	-2.87016	**0.00538**	5.22908	2.95984	1.76668	0.13753	-0.91794	0.35865	-2.78320	**0.00538**
184248	62	13	4.38	-3.51732	6.24363	-0.56334	0.57495	10.89764	3.08131	3.53670	**0.00539**	1.57133	0.11611	2.78297	**0.00539**
41229	70	5	19.20	-16.47291	9.29383	-1.77246	0.08055	-6.43039	1.11462	-5.76914	**0.02876**	-2.78199	**0.00540**	-2.78199	**0.00540**
4288931	60	15	21.40	16.26011	5.67422	2.86561	**0.00545**	-0.67602	3.49311	-0.19353	0.84978	1.83114	0.06708	2.77902	**0.00545**
3141342	60	15	4.33	2.64516	5.98503	0.44196	0.65984	-16.05184	4.77594	-3.36098	**0.00566**	-1.64508	0.09995	-2.76663	**0.00566**
1599042	63	12	21.33	17.40825	6.13678	2.83671	**0.00592**	4.26915	4.76930	0.89513	0.39403	2.54895	**0.01080**	2.75243	**0.00592**
4296763	70	5	102.80	-5.82722	9.48350	-0.61446	0.54085	-3.40194	0.26343	-12.91382	**0.00594**	-2.37760	**0.01743**	-2.75091	**0.00594**
1679707	62	13	3.23	-16.79015	5.94245	-2.82546	**0.00611**	7.62029	4.87507	1.56311	0.14909	-0.91875	0.35822	-2.74207	**0.00611**
186913	70	5	2.00	26.30931	9.31248	2.82517	**0.00611**	1.66314	15.78070	0.10539	0.92568	2.00470	**0.04500**	2.74180	**0.00611**
730906	53	22	2.91	-14.18083	5.04214	-2.81246	**0.00633**	-4.25170	4.17520	-1.01832	0.32132	-2.63174	**0.00849**	-2.73009	**0.00633**
750071	55	20	247.60	11.58759	5.19352	2.23116	**0.02879**	3.06767	1.74494	1.75804	0.09673	2.72047	**0.00652**	2.72047	**0.00652**
302333	63	12	2.50	-2.15856	6.46778	-0.33374	0.73955	-17.75142	5.07464	-3.49807	**0.00674**	-2.15079	**0.03149**	-2.70923	**0.00674**
180552	60	15	4.27	-8.88813	5.83295	-1.52378	0.13194	-12.93252	4.87103	-2.65499	**0.02098**	-2.69742	**0.00699**	-2.69742	**0.00699**
2331530	71	4	1.50	9.36121	10.48980	0.89241	0.37515	-37.54324	0.41251	-91.01093	**0.00699**	-1.28002	0.20054	-2.69710	**0.00699**
825808	43	32	39.38	-12.63515	4.55329	-2.77495	**0.00703**	-1.24808	1.63706	-0.76239	0.45198	-2.43781	**0.01478**	-2.69547	**0.00703**
191792	19	56	14.52	10.20674	5.30641	1.92347	0.05837	3.19653	1.63250	1.95806	0.05550	2.69255	**0.00709**	2.69255	**0.00709**
161007	68	7	1.43	-21.40135	7.75189	-2.76079	**0.00731**	-15.68725	15.65006	-1.00238	0.37288	-2.52683	**0.01151**	-2.68239	**0.00731**
4425368	68	7	19.71	-10.65979	8.06752	-1.32132	0.19058	-6.59063	1.54941	-4.25363	**0.01312**	-2.67949	**0.00737**	-2.67949	**0.00737**
564320	66	9	5.78	13.24153	7.11481	1.86112	0.06681	18.85883	7.77264	2.42631	0.05142	2.67351	**0.00751**	2.67351	**0.00751**
315223	62	13	6.62	-1.40114	6.25449	-0.22402	0.82338	-10.39660	3.11633	-3.33617	**0.00754**	-2.04725	**0.04063**	-2.67204	**0.00754**
347243	71	4	2.25	27.77277	10.15788	2.73411	**0.00787**	-12.63501	20.75306	-0.60883	0.65184	1.56023	0.11870	2.65771	**0.00787**
184174	31	44	9.09	-9.62928	4.68442	-2.05560	**0.04345**	-3.47876	1.97568	-1.76079	0.08573	-2.64297	**0.00822**	-2.64297	**0.00822**
4383922	69	6	2.00	-22.64861	8.34203	-2.71500	**0.00829**	-7.50364	9.39308	-0.79885	0.48277	-2.36305	**0.01813**	-2.64001	**0.00829**
4454531	48	27	12.30	-12.68433	4.72714	-2.68330	**0.00904**	1.69457	2.74605	0.61709	0.54299	-1.41585	0.15682	-2.61061	**0.00904**
289734	19	56	17.04	-7.82875	5.36297	-1.45978	0.14870	-3.73840	1.62514	-2.30035	**0.02539**	-2.60180	**0.00927**	-2.60180	**0.00927**
192912	68	7	4.00	-18.95798	7.82235	-2.42356	**0.01788**	-8.24358	5.24131	-1.57281	0.19087	-2.59935	**0.00934**	-2.59935	**0.00934**
194320	44	31	3.90	12.14700	4.58861	2.64721	**0.00996**	-1.18671	3.41837	-0.34716	0.73107	1.57924	0.11428	2.57709	**0.00996**
185861	66	9	2.33	18.45330	6.97165	2.64691	**0.00997**	13.86493	18.01223	0.76975	0.47066	2.33219	**0.01969**	2.57681	**0.00997**
197540	69	6	4.33	19.67821	8.46956	2.32340	**0.02299**	21.18513	11.80659	1.79435	0.17064	2.57657	**0.00998**	2.57657	**0.00998**
177758	64	11	4.82	-8.96680	6.60513	-1.35755	0.17885	-7.31402	2.56764	-2.84854	**0.02153**	-2.57593	**0.01000**	-2.57593	**0.01000**
114400	71	4	3.25	26.66706	10.08524	2.64417	**0.01005**	-16.62069	7.57721	-2.19351	0.27231	1.04404	0.29646	2.57426	**0.01005**
4459708	71	4	2.00	-20.99983	10.42537	-2.01430	**0.04771**	28.81613	0.46022	62.61387	**0.01017**	0.41734	0.67643	2.57011	**0.01017**
320786	67	8	3.38	-16.15355	7.43030	-2.17401	**0.03299**	-5.80914	3.25490	-1.78474	0.13437	-2.56627	**0.01028**	-2.56627	**0.01028**
194670	26	49	7.55	-4.07028	5.01197	-0.81211	0.41940	-5.08079	1.89914	-2.67532	**0.01031**	-2.38496	**0.01708**	-2.56539	**0.01031**
4333897	63	12	2.67	-16.20971	6.16842	-2.62786	**0.01049**	4.87649	6.01408	0.81084	0.43837	-1.26158	0.20710	-2.55909	**0.01049**
389371	60	15	4.07	-14.91224	5.67564	-2.62741	**0.01051**	9.81908	3.50484	2.80158	**0.01600**	-0.10585	0.91570	-2.55868	**0.01051**
187883	64	11	2.45	-13.47873	6.56141	-2.05424	**0.04358**	-10.57509	5.91339	-1.78833	0.11152	-2.55227	**0.01070**	-2.55227	**0.01070**
997267	70	5	3.00	24.18485	9.27333	2.60800	**0.01107**	0.13760	5.63260	0.02443	0.98273	1.81179	0.07002	2.54060	**0.01107**
293883	58	17	13.82	14.08985	5.40557	2.60655	**0.01111**	-2.47945	3.26659	-0.75903	0.46042	1.27357	0.20282	2.53925	**0.01111**
158649	71	4	2.25	-26.30537	10.11268	-2.60123	**0.01127**	11.03237	0.80464	13.71088	**0.04635**	-0.38332	0.70148	-2.53429	**0.01127**
4294457	64	11	4.64	13.49883	6.64306	2.03202	**0.04584**	8.82598	5.00543	1.76328	0.11587	2.52381	**0.01161**	2.52381	**0.01161**
197698	55	20	8.15	13.26671	5.13087	2.58566	**0.01174**	-5.06352	2.72049	-1.86125	0.08009	0.54421	0.58630	2.51978	**0.01174**
3014082	67	8	3.88	-19.31087	7.49072	-2.57797	**0.01198**	-0.92865	5.57384	-0.16661	0.87421	-1.88863	0.05894	-2.51261	**0.01198**
849535	65	10	315.80	17.17246	6.66786	2.57541	**0.01207**	-1.42239	2.63755	-0.53929	0.60641	1.41069	0.15834	2.51022	**0.01207**

Table S4d. OTUs associated with Low-density lipoprotein (LDL) at FDR < 0.05 level [a] (Cont.)

	Summary of OTUs reads			Binary model				Quantitative model				Meta analysis		Final association	
OTU	No. Absent	No. Present	mean counts in presents	Estimate	s.e.	*t* value	*P* value	Estimate	s.e.	*t* value	*P* value	Meta z value	*Meta P* value	*z*	*P value*
177222	43	32	5.00	-11.78192	4.58412	-2.57016	**0.01223**	0.31382	2.61503	0.12001	0.90531	-1.68741	0.09152	-2.50532	**0.01223**
175922	69	6	2.67	21.46556	8.36072	2.56743	**0.01232**	8.09196	6.61350	1.22355	0.30846	2.48989	**0.01278**	2.50277	**0.01232**
511378	69	6	4.67	-21.44740	8.36077	-2.56524	**0.01239**	3.27548	7.31088	0.44803	0.68451	-1.48098	0.13861	-2.50073	**0.01239**
4307484	39	36	3.86	3.00200	4.72340	0.63556	0.52708	-8.35818	3.17152	-2.63539	**0.01270**	-1.31485	0.18856	-2.49195	**0.01270**
186687	60	15	2.60	-14.51348	5.68532	-2.55280	**0.01281**	-1.54854	5.88471	-0.26315	0.79690	-1.94205	0.05213	-2.48911	**0.01281**
178478	34	41	4.98	1.27133	4.80131	0.26479	0.79193	-5.56927	2.15322	-2.58649	**0.01365**	-1.55741	0.11937	-2.46631	**0.01365**
760544	63	12	4.67	-8.76776	6.53087	-1.34251	0.18365	-9.28375	3.63697	-2.55261	**0.03106**	-2.46489	**0.01371**	-2.46489	**0.01371**
360636	32	43	29.30	1.27715	4.79847	0.26616	0.79088	-3.37064	1.31305	-2.56703	**0.01410**	-1.54817	0.12158	-2.45462	**0.01410**
197499	33	42	8.17	11.60486	4.61760	2.51318	**0.01420**	-0.34621	2.85142	-0.12142	0.90399	1.64858	0.09923	2.45207	**0.01420**
193968	17	58	13.78	7.28338	5.58655	1.30374	0.19648	-3.71631	1.47181	-2.52500	**0.01448**	-0.81558	0.41474	-2.44505	**0.01448**
300235	60	15	1.60	-14.21473	5.67404	-2.50522	**0.01450**	4.46741	7.88620	0.56648	0.58151	-1.33886	0.18062	-2.44462	**0.01450**
656881	54	21	8.67	-12.87293	5.15750	-2.49596	**0.01485**	0.11916	2.16502	0.05504	0.95671	-1.68410	0.09216	-2.43595	**0.01485**
198909	49	26	7.35	0.79076	5.06270	0.15619	0.87632	-7.34490	2.79113	-2.63151	**0.01492**	-1.61132	0.10711	-2.43439	**0.01492**
294791	69	6	2.67	1.62642	8.73296	0.18624	0.85278	-21.00166	4.16585	-5.04139	**0.01505**	-1.58791	0.11231	-2.43122	**0.01505**
4435309	70	5	7.80	22.83528	9.17929	2.48770	**0.01517**	4.16809	8.92702	0.46691	0.68649	2.00240	**0.04524**	2.42821	**0.01517**
782953	15	60	127.50	-9.95535	5.82218	-1.70990	0.09159	-1.73077	0.97519	-1.77480	0.08127	-2.42568	**0.01528**	-2.42568	**0.01528**
3826120	71	4	1.75	-18.05304	10.32667	-1.74820	0.08469	-8.13602	1.14236	-7.12211	0.08881	-2.42242	**0.01542**	-2.42242	**0.01542**
291644	63	12	3.08	-5.17380	6.47949	-0.79849	0.42721	19.54576	6.56809	2.97587	**0.01555**	1.14923	0.25046	2.41923	**0.01555**
19611	55	20	3.95	12.76410	5.15883	2.47422	**0.01571**	1.20255	4.00213	0.30048	0.76746	1.91717	0.05522	2.41558	**0.01571**
4418787	65	10	7.20	-13.66774	6.78117	-2.01554	**0.04758**	-6.32340	3.92928	-1.60930	0.15159	-2.41480	**0.01574**	-2.41480	**0.01574**
332588	58	17	2.88	-13.42256	5.42941	-2.47220	**0.01579**	10.09564	5.91530	1.70670	0.10995	-0.57648	0.56429	-2.41368	**0.01579**
2046330	67	8	5.88	18.17297	7.36124	2.46874	**0.01593**	-2.27283	12.74650	-0.17831	0.86548	1.58465	0.11305	2.41044	**0.01593**
177062	65	10	3.70	6.87323	6.91951	0.99331	0.32388	-19.62657	6.21263	-3.15914	**0.01595**	-1.00664	0.31411	-2.41012	**0.01595**
1105984	40	35	97.49	-9.09981	4.65098	-1.95653	0.05428	-1.55809	1.03099	-1.51126	0.14053	-2.40302	**0.01626**	-2.40302	**0.01626**
4429981	61	14	38.07	14.32305	5.83648	2.45406	**0.01655**	-6.29013	3.48664	-1.80407	0.09864	0.52694	0.59824	2.39666	**0.01655**
4469032	70	5	5.80	18.38960	9.25001	1.98806	0.05061	13.27955	5.85506	2.26805	0.15144	2.39656	**0.01655**	2.39656	**0.01655**
537219	63	12	1.92	10.49846	6.33782	1.65648	0.10198	16.85988	8.54719	1.97256	0.08001	2.39424	**0.01665**	2.39424	**0.01665**
208739	32	43	3.77	1.64480	4.81147	0.34185	0.73346	-5.40268	2.17100	-2.48856	**0.01709**	-1.44552	0.14831	-2.38480	**0.01709**
189235	68	7	8.29	19.11760	7.84773	2.43607	**0.01732**	-6.71625	7.03295	-0.95497	0.39367	1.07961	0.28032	2.37977	**0.01732**
184342	68	7	1.86	-19.09065	7.86798	-2.42637	**0.01776**	5.55774	9.70181	0.57286	0.59741	-1.30287	0.19262	-2.37067	**0.01776**
4311621	67	8	2.50	17.91572	7.40311	2.42003	**0.01804**	0.96770	9.36737	0.10331	0.92174	1.74157	0.08158	2.36470	**0.01804**
1110312	66	9	8.44	0.42777	7.31895	0.05845	0.95355	-12.45536	3.86404	-3.22340	**0.01806**	-1.63069	0.10296	-2.36439	**0.01806**
171559	61	14	1.57	-14.12991	5.85675	-2.41258	**0.01839**	10.99410	8.69877	1.26387	0.23240	-0.82273	0.41066	-2.35770	**0.01839**
180341	70	5	1.60	-21.99111	9.15306	-2.40260	**0.01886**	-3.00282	8.34702	-0.35975	0.75347	-1.88258	0.05976	-2.34831	**0.01886**
186732	18	57	11.37	1.10321	5.56063	0.19840	0.84329	-4.23834	1.75178	-2.41945	**0.01894**	-1.51957	0.12862	-2.34668	**0.01894**
230479	70	5	4.00	17.46260	9.27159	1.88345	0.06368	12.67720	5.39774	2.34861	0.14332	2.34616	**0.01897**	2.34616	**0.01897**
180121	71	4	3.25	24.43042	10.20386	2.39423	**0.01926**	-7.59061	44.31301	-0.17130	0.89200	1.55893	0.11901	2.34044	**0.01926**
3195500	46	29	5.17	-11.18393	4.67708	-2.39122	**0.01941**	5.33190	3.06658	1.73871	0.09392	-0.46847	0.63945	-2.33761	**0.01941**
179744	56	19	16.32	12.51322	5.23732	2.38924	**0.01950**	0.14947	2.90370	0.05148	0.95958	1.68745	0.09152	2.33574	**0.01950**
4419459	55	20	16.10	12.29083	5.16602	2.37917	**0.02000**	1.05787	2.88320	0.36691	0.71821	1.90007	0.05742	2.32626	**0.02000**
337161	62	13	9.46	9.33447	6.15498	1.51657	0.13375	8.03199	4.01682	1.99959	0.07344	2.32607	**0.02001**	2.32607	**0.02001**
187388	62	13	2.62	-1.22387	6.25571	-0.19564	0.84544	9.18356	3.32662	2.76062	**0.02011**	1.50570	0.13214	2.32432	**0.02011**
1602805	48	27	5.70	-10.27774	4.78211	-2.14920	**0.03498**	-4.21752	3.50291	-1.20400	0.24033	-2.32126	**0.02027**	-2.32126	**0.02027**
196061	68	7	5.57	-19.34828	8.16781	-2.36885	**0.02053**	-3.14524	7.53506	-0.41741	0.69781	-1.91260	0.05580	-2.31654	**0.02053**
193053	18	57	11.40	11.32337	5.37773	2.10560	**0.03873**	2.26632	1.86034	1.21823	0.22843	2.31329	**0.02071**	2.31329	**0.02071**
2280825	68	7	3.14	18.63227	7.88003	2.36449	**0.02075**	15.36177	15.38578	0.99844	0.37457	2.26301	**0.02364**	2.31243	**0.02075**
301578	61	14	14.29	-12.39692	6.07513	-2.04060	**0.04496**	-3.63350	2.73609	-1.32799	0.21108	-2.30209	**0.02133**	-2.30209	**0.02133**
2423305	57	18	7.61	12.64728	5.37668	2.35225	**0.02140**	2.61544	2.65562	0.98487	0.34031	2.30124	**0.02138**	2.30124	**0.02138**
158855	67	8	11.63	4.08597	7.65094	0.53405	0.59495	8.79437	2.66272	3.30277	**0.02141**	2.00277	**0.04520**	2.30068	**0.02141**
4377149	49	26	11.12	11.23167	4.79301	2.34334	**0.02188**	-1.45942	2.29166	-0.63684	0.53052	1.17753	0.23898	2.29249	**0.02188**
174831	43	32	34.69	10.82683	4.62079	2.34307	**0.02189**	-0.32078	1.78948	-0.17926	0.85898	1.49522	0.13486	2.29224	**0.02189**
187126	57	18	3.22	-12.51394	5.34099	-2.34300	**0.02190**	4.39269	3.42318	1.28322	0.21889	-0.75143	0.45240	-2.29217	**0.02190**
198190	69	6	1.50	-11.92948	8.60899	-1.38570	0.17012	38.48179	8.76711	4.38933	**0.02190**	0.65067	0.51526	2.29201	**0.02190**
190991	63	12	1.92	-14.59073	6.23319	-2.34081	**0.02202**	5.66536	6.46491	0.87632	0.40364	-1.02881	0.30357	-2.29011	**0.02202**
4254313	71	4	1031.50	23.95371	10.24013	2.33920	**0.02210**	0.33957	1.04991	0.32343	0.80086	1.79663	0.07239	2.28858	**0.02210**
4354103	66	9	34.22	16.39973	7.02284	2.33520	**0.02232**	-0.38447	4.18372	-0.09190	0.92977	1.55329	0.12035	2.28481	**0.02232**
183579	57	18	3.67	-12.58890	5.39147	-2.33497	**0.02234**	-0.66967	3.11961	-0.21467	0.83292	-1.76462	0.07763	-2.28459	**0.02234**
197760	70	5	2.00	2.20635	9.55392	0.23094	0.81802	-48.64536	7.50353	-6.48299	**0.02298**	-1.44514	0.14842	-2.27383	**0.02298**
688701	64	11	3.27	-3.34517	6.67787	-0.50093	0.61795	16.57325	5.91239	2.80314	**0.02309**	1.25387	0.20989	2.27201	**0.02309**
544996	65	10	5.00	5.82627	7.04130	0.82744	0.41072	-6.94180	2.39636	-2.89681	**0.02309**	-1.02481	0.30545	-2.27193	**0.02309**
1508541	40	35	4.03	-10.61321	4.57547	-2.31959	**0.02320**	-0.42923	3.12458	-0.13737	0.89160	-1.70155	0.08884	-2.27008	**0.02320**
4366834	61	14	113.14	9.63545	5.99526	1.60718	0.11239	4.13712	2.34583	1.76361	0.10552	2.26713	**0.02338**	2.26713	**0.02338**
199430	38	37	3.59	-10.60373	4.58387	-2.31327	**0.02357**	0.10751	2.40041	0.04479	0.96454	-1.56953	0.11652	-2.26411	**0.02357**
113827	68	7	2.71	18.46711	7.99844	2.30884	**0.02383**	-17.26034	9.68532	-1.78211	0.14932	0.57839	0.56300	2.25992	**0.02383**
307113	63	12	1.67	-14.38819	6.23492	-2.30768	**0.02389**	-3.72089	6.65580	-0.55904	0.58977	-1.97848	**0.04787**	-2.25883	**0.02389**
4045882	63	12	1.67	-14.38819	6.23492	-2.30768	**0.02389**	-3.72089	6.65580	-0.55904	0.58977	-1.97848	**0.04787**	-2.25883	**0.02389**
311718	71	4	3.25	23.49230	10.20105	2.30293	**0.02417**	-17.34392	3.53177	-4.91083	0.12789	0.51750	0.60481	2.25434	**0.02417**
192046	52	23	4.78	-1.68772	5.12868	-0.32908	0.74305	-9.01784	3.71423	-2.42791	**0.02475**	-1.81948	0.06884	-2.24532	**0.02475**
562410	69	6	1.50	-11.49070	8.65233	-1.32805	0.18836	-18.92804	6.57153	-2.88031	0.06351	-2.24226	**0.02494**	-2.24226	**0.02494**
3887769	55	20	1.95	-11.94135	5.21566	-2.28952	**0.02498**	9.33788	5.86436	1.59231	0.12974	-0.51373	0.60744	-2.24166	**0.02498**
186122	70	5	3.40	-11.73568	9.40720	-1.24752	0.21625	11.03317	1.78173	6.19238	**0.02510**	0.70943	0.47806	2.23985	**0.02510**
3709990	63	12	3.08	-7.09836	6.54274	-1.08492	0.28158	-12.41207	5.06068	-2.45265	**0.03660**	-2.23942	**0.02513**	-2.23942	**0.02513**
298451	70	5	26.80	18.49607	9.23481	2.00286	**0.04896**	18.12237	10.68748	1.69566	0.23204	2.23735	**0.02526**	2.23735	**0.02526**
72820	26	49	33.04	-7.75038	4.98050	-1.55615	0.12406	-2.18259	1.31743	-1.65670	0.10439	-2.23581	**0.02536**	-2.23581	**0.02536**
176077	66	9	2.67	16.04993	7.03326	2.28200	**0.02545**	-1.78713	9.95912	-0.17945	0.86349	1.45850	0.14470	2.23456	**0.02545**
268730	62	13	120.54	9.29059	6.15902	1.50845	0.13581	3.94175	2.14170	1.84048	0.09552	2.23340	**0.02552**	2.23340	**0.02552**
301253	40	35	35.94	10.44357	4.58086	2.27983	**0.02558**	0.10031	1.90424	0.05268	0.95832	1.61557	0.10619	2.23250	**0.02558**

Table S4d. OTUs associated with Low-density lipoprotein (LDL) at FDR < 0.05 level [a] (Cont.)

	Summary of OTUs reads			Binary model				Quantitative model				Meta analysis		Final association	
OTU	No. Absent	No. Present	mean counts in presents	Estimate	s.e.	*t* value	*P* value	Estimate	s.e.	*t* value	*P* value	Meta z value	*Meta P* value	*z*	*P value*
232222	70	5	6.60	20.89464	9.19952	2.27127	**0.02612**	7.18856	5.99562	1.19897	0.35333	2.22920	**0.02580**	2.22920	**0.02580**
1566691	69	6	2.33	-19.33250	8.50209	-2.27385	**0.02596**	-3.41588	12.62621	-0.27054	0.80429	-1.74984	0.08015	-2.22684	**0.02596**
663500	55	20	69.10	11.36734	5.18094	2.19407	**0.03146**	2.02326	1.97203	1.02598	0.31928	2.22538	**0.02606**	2.22538	**0.02606**
515299	70	5	14.60	-1.68632	9.48650	-0.17776	0.85941	-14.28656	2.35783	-6.05921	**0.02617**	-1.69759	0.08958	-2.22364	**0.02617**
182289	34	41	10.37	-8.72602	4.64148	-1.88001	0.06415	-2.34816	1.79101	-1.31109	0.19770	-2.21979	**0.02643**	-2.21979	**0.02643**
3465233	56	19	7.47	-0.38312	5.45073	-0.07029	0.94416	-8.06888	3.30135	-2.44411	**0.02648**	-1.61864	0.10552	-2.21906	**0.02648**
2341726	69	6	2.00	-8.85261	8.76251	-1.01028	0.31574	-9.69267	2.60847	-3.71584	**0.03390**	-2.20932	**0.02715**	-2.20932	**0.02715**
288651	26	49	32.88	0.12993	4.98313	0.02607	0.97927	-2.87694	1.26169	-2.28023	**0.02728**	-1.54259	0.12293	-2.20754	**0.02728**
4338990	54	21	11.48	8.27520	5.18534	1.59589	0.11489	3.85943	2.39106	1.61411	0.12390	2.20277	**0.02761**	2.20277	**0.02761**
197426	70	5	4.80	-14.65043	9.38497	-1.56105	0.12290	-13.52442	5.08152	-2.66149	0.11692	-2.19949	**0.02784**	-2.19949	**0.02784**
181918	69	6	2.50	0.59898	8.73785	0.06855	0.94554	20.29125	5.07391	3.99914	**0.02802**	1.60178	0.10920	2.19695	**0.02802**
4358921	59	16	1.69	-12.52384	5.59247	-2.23941	**0.02822**	-5.51719	6.44680	-0.85580	0.40760	-2.13712	**0.03259**	-2.19423	**0.02822**
180825	71	4	2.25	-20.13912	10.26693	-1.96155	0.05368	-22.44619	8.93889	-2.51107	0.24127	-2.19289	**0.02832**	-2.19289	**0.02832**
4020046	64	11	7.64	-14.54489	6.50600	-2.23561	**0.02848**	7.58041	4.36869	1.73517	0.12093	-0.45237	0.65100	-2.19063	**0.02848**
509709	68	7	1.71	8.88624	8.08137	1.09960	0.27517	-39.01595	11.63859	-3.35229	**0.02851**	-0.77710	0.43710	-2.19022	**0.02851**
229088	71	4	7.25	19.52727	10.27896	1.89973	0.06147	12.75541	4.63564	2.75160	0.22192	2.18604	**0.02881**	2.18604	**0.02881**
4425571	30	45	21.82	-9.56951	4.74944	-2.01487	**0.04765**	-1.66457	1.48188	-1.12328	0.26770	-2.18413	**0.02895**	-2.18413	**0.02895**
310247	70	5	2.00	-20.61426	9.26096	-2.22593	**0.02915**	35.96538	37.50569	0.95893	0.43878	-0.99505	0.31971	-2.18146	**0.02915**
4472130	40	35	5.14	10.21205	4.59527	2.22230	**0.02941**	1.62555	3.72024	0.43695	0.66508	1.84620	0.06486	2.17801	**0.02941**
518820	70	5	10.40	20.63366	9.31245	2.21571	**0.02987**	-24.44225	125.55012	-0.19468	0.86363	1.41421	0.15730	2.17176	**0.02987**
312311	68	7	2.57	-7.30610	8.10506	-0.90142	0.37037	-34.84946	10.57684	-3.29488	**0.03008**	-2.16718	**0.03022**	-2.16907	**0.03008**
185864	56	19	4.95	1.73437	5.48959	0.31594	0.75296	-8.71835	3.69074	-2.36223	**0.03117**	-1.30117	0.19320	-2.15487	**0.03117**
4379449	68	7	31.43	-0.87960	8.20209	-0.10724	0.91490	-5.75344	1.76883	-3.25267	**0.03130**	-1.59816	0.11001	-2.15328	**0.03130**
581201	48	27	4.33	10.64822	4.85181	2.19469	**0.03141**	3.34470	4.66840	0.71646	0.48062	2.02028	**0.04335**	2.15181	**0.03141**
4483045	67	8	1.50	-15.04771	7.49127	-2.00870	**0.04832**	-19.46028	16.31954	-1.19245	0.28658	-2.14974	**0.03158**	-2.14974	**0.03158**
199293	8	67	15.46	-11.36520	7.57708	-1.49995	0.13800	-2.18053	1.38343	-1.57618	0.11992	-2.14848	**0.03168**	-2.14848	**0.03168**
4433737	62	13	7.54	-2.12816	6.28482	-0.33862	0.73588	8.33726	3.34656	2.49130	**0.03192**	1.27853	0.20106	2.14542	**0.03192**
195436	37	38	4.76	-10.07244	4.61203	-2.18395	**0.03222**	3.99175	2.31523	1.72413	0.09351	-0.32841	0.74260	-2.14162	**0.03222**
591439	69	6	2.67	15.08804	8.59941	1.75454	0.08359	21.18671	12.71306	1.66653	0.19420	2.14145	**0.03224**	2.14145	**0.03224**
184238	31	44	10.70	10.21115	4.69852	2.17327	**0.03305**	-2.18242	2.04100	-1.06929	0.29120	0.76082	0.44676	2.13147	**0.03305**
168439	44	31	33.58	-0.15940	5.00842	-0.03183	0.97470	-3.48171	1.55291	-2.24205	**0.03306**	-1.52950	0.12614	-2.13132	**0.03306**
4438999	61	14	3.93	4.97028	6.04554	0.82214	0.41371	-9.33750	3.83606	-2.43414	**0.03317**	-0.92822	0.35330	-2.13008	**0.03317**
180107	61	14	3.93	4.97028	6.04554	0.82214	0.41371	-9.33750	3.83606	-2.43414	**0.03317**	-0.92822	0.35330	-2.13008	**0.03317**
191148	71	4	2.50	22.15507	10.20323	2.17138	**0.03320**	4.27399	27.50356	0.15540	0.90186	1.59310	0.11114	2.12967	**0.03320**
367813	65	10	18.50	11.84985	6.82684	1.73577	0.08688	7.25809	5.05213	1.43664	0.19397	2.12910	**0.03325**	2.12910	**0.03325**
299407	55	20	11.20	6.94225	5.28890	1.31261	0.19348	5.07175	2.79515	1.81448	0.08729	2.12854	**0.03329**	2.12854	**0.03329**
158423	60	15	79.60	0.38358	5.92762	0.06471	0.94858	4.82657	2.01114	2.39992	**0.03352**	1.54873	0.12145	2.12575	**0.03352**
4443846	46	29	6.10	-8.72608	4.82393	-1.80891	0.07464	-3.18156	2.53804	-1.25355	0.22116	-2.12565	**0.03353**	-2.12565	**0.03353**
113654	67	8	2.88	-9.90391	7.57691	-1.30712	0.19533	-34.62786	16.31350	-2.12265	0.08721	-2.12504	**0.03358**	-2.12504	**0.03358**
4315788	68	7	10.43	10.71832	8.06020	1.32978	0.18779	14.39060	6.58315	2.18597	0.09411	2.11512	**0.03442**	2.11512	**0.03442**
183604	65	10	3.50	-14.57335	6.76876	-2.15303	**0.03467**	1.47711	3.14414	0.46980	0.65278	-1.17544	0.23982	-2.11223	**0.03467**
175844	49	26	104.65	-10.37505	4.83180	-2.14724	**0.03514**	0.87230	1.48705	0.58660	0.56319	-1.08089	0.27975	-2.10672	**0.03514**
180462	68	7	2.29	-14.92898	7.95301	-1.87715	0.06455	-16.28100	12.36019	-1.31721	0.25815	-2.10656	**0.03516**	-2.10656	**0.03516**
187179	61	14	9.86	-12.80080	5.96275	-2.14679	**0.03518**	-0.82618	3.10108	-0.26642	0.79485	-1.67324	0.09428	-2.10629	**0.03518**
192741	49	26	10.42	10.37239	4.83213	2.14654	**0.03520**	-2.51507	2.28985	-1.09836	0.28341	0.73071	0.46496	2.10606	**0.03520**
3507744	68	7	4.57	-16.90750	7.89232	-2.14227	**0.03555**	5.87076	3.98831	1.47199	0.21500	-0.60957	0.54215	-2.10199	**0.03555**
535955	64	11	2.36	8.92343	6.61762	1.34843	0.18175	13.25453	7.19462	1.84228	0.10268	2.09825	**0.03588**	2.09825	**0.03588**
209760	65	10	2.20	-3.62594	6.96750	-0.52041	0.60438	8.34548	3.22090	2.59104	**0.03589**	1.11726	0.26388	2.09817	**0.03589**
192438	52	23	12.48	-7.77997	5.10533	-1.52389	0.13192	-3.28416	2.16076	-1.51991	0.14419	-2.09796	**0.03591**	-2.09796	**0.03591**
189271	68	7	1.29	1.91240	8.13619	0.23505	0.81484	23.58218	7.59865	3.10347	**0.03610**	1.64755	0.09944	2.09581	**0.03610**
319275	44	31	5.61	-5.46129	4.80729	-1.13604	0.25971	-5.30709	2.77666	-1.91132	0.06625	-2.09574	**0.03611**	-2.09574	**0.03611**
195252	67	8	4.00	15.85947	7.43458	2.13320	**0.03632**	-2.74168	7.13861	-0.38406	0.71672	1.22366	0.22108	2.09336	**0.03632**
187386	53	22	3.86	-2.14958	5.19411	-0.41385	0.68021	-6.75550	3.00246	-2.24999	**0.03649**	-1.77028	0.07668	-2.09138	**0.03649**
365385	56	19	7.63	-5.55062	5.40387	-1.02716	0.30779	-6.81965	3.27177	-2.08439	0.05351	-2.08643	**0.03694**	-2.08643	**0.03694**
4381555	62	13	3.92	-9.91791	6.30598	-1.57278	0.12015	-6.57244	4.36275	-1.50649	0.16286	-2.08571	**0.03700**	-2.08571	**0.03700**
197179	69	6	3.83	18.01847	8.47897	2.12508	**0.03701**	-18.11228	6.58765	-2.74943	0.07078	0.19708	0.84376	2.08562	**0.03701**
1986406	71	4	1.75	21.71645	10.22529	2.12380	**0.03712**	-20.90929	24.96693	-0.83748	0.55616	1.05773	0.29018	2.08441	**0.03712**
183454	67	8	15.75	14.75480	7.52768	1.96007	0.05386	5.33735	4.70240	1.13503	0.30783	2.08439	**0.03712**	2.08439	**0.03712**
539647	60	15	19.27	-0.16128	5.91782	-0.02725	0.97833	5.84996	2.50299	2.33719	**0.03758**	1.45118	0.14673	2.07944	**0.03758**
48084	35	40	36.20	9.74876	4.60219	2.11829	**0.03760**	-0.86021	1.41023	-0.60998	0.54560	1.04284	0.29702	2.07916	**0.03760**
182483	60	15	53.40	12.27166	5.79618	2.11720	**0.03770**	-1.60375	2.43407	-0.65888	0.52242	1.01717	0.30907	2.07812	**0.03770**
199761	25	50	9.80	-10.30836	4.88136	-2.11178	**0.03818**	-0.72255	1.93611	-0.37320	0.71068	-1.72810	0.08397	-2.07296	**0.03818**
182577	43	32	3.63	-6.77656	4.71760	-1.43644	0.15521	-5.00074	3.22103	-1.55253	0.13138	-2.07186	**0.03828**	-2.07186	**0.03828**
362765	46	29	4.90	-9.61136	4.76152	-2.01855	**0.04726**	-2.26776	2.35572	-0.96266	0.34459	-2.07119	**0.03834**	-2.07119	**0.03834**
197696	68	7	3.71	5.27779	8.16177	0.64665	0.51992	11.32303	3.73058	3.03519	**0.03858**	1.91773	0.05515	2.06860	**0.03858**
297385	68	7	2.57	-14.52409	8.04696	-1.80492	0.07527	-15.44160	11.54314	-1.33773	0.25198	-2.06785	**0.03865**	-2.06785	**0.03865**
4465124	40	35	31.57	6.66319	4.69373	1.41959	0.16004	2.98890	1.92300	1.55429	0.12995	2.06421	**0.03900**	2.06421	**0.03900**
158310	68	7	25.43	-7.81188	8.14482	-0.95912	0.34071	10.44594	3.45736	3.02137	**0.03911**	0.78507	0.43242	2.06302	**0.03911**
348304	53	22	3.86	-11.33853	5.40726	-2.09691	**0.03951**	6.18374	3.84062	1.61009	0.12387	-0.36773	0.71308	-2.05879	**0.03951**
186352	29	46	8.22	0.02397	4.94492	0.00485	0.99615	-4.98215	2.34806	-2.12182	**0.03965**	-1.45134	0.14669	-2.05733	**0.03965**
19080	62	13	64.00	7.65886	6.32742	1.21042	0.23008	3.25862	1.72014	1.89440	0.08743	2.05718	**0.03967**	2.05718	**0.03967**
617098	64	11	4.18	7.30232	6.64044	1.09967	0.27514	10.58742	5.04728	2.09765	0.06919	2.05661	**0.03972**	2.05661	**0.03972**
195081	48	27	6.33	-0.20218	4.93443	-0.04097	0.96743	-6.90726	3.18160	-2.17100	**0.04005**	-1.48076	0.13867	-2.05328	**0.04005**
192231	70	5	1.00	19.26371	9.21656	2.09012	**0.04014**	0.00000	0.00000	0.00000	1.00000	1.45120	0.14672	2.05231	**0.04014**
193191	40	35	34.80	-4.24936	4.72293	-0.89973	0.37126	-3.10459	1.48659	-2.08840	**0.04480**	-2.05103	**0.04026**	-2.05103	**0.04026**
189356	67	8	2.50	7.81426	7.74838	1.00850	0.31659	-11.10934	4.05008	-2.74299	**0.04065**	-0.73937	0.45968	-2.04711	**0.04065**
196724	70	5	1.60	-0.75162	9.55705	-0.07865	0.93753	-5.47871	1.14132	-4.80031	**0.04076**	-1.50212	0.13307	-2.04594	**0.04076**

Table S4d. OTUs associated with Low-density lipoprotein (LDL) at FDR < 0.05 level [a] (Cont.)

	Summary of OTUs reads			Binary model				Quantitative model				Meta analysis		Final association	
OTU	No. Absent	No. Present	mean counts in presents	Estimate	s.e.	*t* value	*P* value	Estimate	s.e.	*t* value	*P* value	Meta z value	*Meta P* value	*z*	*P value*
4462107	69	6	2.67	-17.63967	8.47461	-2.08147	**0.04095**	17.23641	10.41733	1.65459	0.19658	-0.53225	0.59455	-2.04407	**0.04095**
4434579	62	13	83.77	-9.46807	6.16375	-1.53609	0.12890	4.41660	1.88322	2.34525	**0.04097**	0.37149	0.71027	2.04382	**0.04097**
941096	61	14	3.00	-3.40332	6.09694	-0.55820	0.57844	10.13533	4.38219	2.31285	**0.04109**	1.05142	0.29307	2.04260	**0.04109**
1046997	70	5	2.20	-19.13633	9.21668	-2.07627	**0.04144**	41.96582	16.81099	2.49633	0.12992	-0.37102	0.71063	-2.03911	**0.04144**
3138798	31	44	219.93	-9.67748	4.66854	-2.07291	**0.04176**	-0.69154	0.90844	-0.76123	0.45088	-1.97273	**0.04853**	-2.03590	**0.04176**
588471	64	11	315.64	5.73387	6.66689	0.86005	0.39261	2.83990	1.18346	2.39965	**0.04320**	2.03410	**0.04194**	2.03410	**0.04194**
113542	45	30	17.70	9.72484	4.69996	2.06913	**0.04212**	-1.62757	2.09181	-0.77807	0.44329	0.89495	0.37081	2.03229	**0.04212**
180216	56	19	47.32	-7.48430	5.46507	-1.36948	0.17511	-2.90041	1.81801	-1.59537	0.13019	-2.02893	**0.04247**	-2.02893	**0.04247**
359445	67	8	4.13	15.43281	7.48200	2.06266	**0.04275**	2.11211	5.64371	0.37424	0.72357	1.68278	0.09242	2.02611	**0.04275**
163494	59	16	3.44	-11.63567	5.64195	-2.06235	**0.04278**	4.36595	6.43363	0.67861	0.50928	-0.96581	0.33414	-2.02582	**0.04278**
4458227	66	9	16.11	9.60854	7.19460	1.33552	0.18591	-9.83031	3.84348	-2.55766	**0.04304**	-0.49534	0.62036	-2.02328	**0.04304**
174862	70	5	2.80	-14.51379	9.40316	-1.54350	0.12709	-96.79624	48.16774	-2.00957	0.18221	-2.02208	**0.04317**	-2.02208	**0.04317**
3318103	59	16	2.38	-0.27603	5.78411	-0.04772	0.96207	13.99893	6.25365	2.23852	**0.04332**	1.39519	0.16296	2.02066	**0.04332**
211720	60	15	46.73	3.75318	5.92501	0.63345	0.52845	4.25210	1.88265	2.25857	**0.04332**	1.87451	0.06086	2.02058	**0.04332**
4451906	44	31	4.68	5.46640	4.83881	1.12970	0.26235	5.69919	3.16867	1.79861	0.08287	2.01887	**0.04350**	2.01887	**0.04350**
182911	39	36	7.17	-7.17050	4.66477	-1.53716	0.12864	-2.93806	2.16330	-1.35814	0.18363	-2.01465	**0.04394**	-2.01465	**0.04394**
197970	27	48	8.15	-9.90085	4.83607	-2.04729	**0.04428**	0.71590	1.91403	0.37403	0.71014	-1.15949	0.24625	-2.01144	**0.04428**
793109	68	7	13.57	13.97993	8.17689	1.70969	0.09163	6.13411	4.56736	1.34303	0.25040	2.00552	**0.04491**	2.00552	**0.04491**
188851	43	32	293.06	6.86774	4.79102	1.43346	0.15605	1.65990	1.14291	1.45235	0.15714	2.00340	**0.04513**	2.00340	**0.04513**
197539	69	6	4.50	12.68150	8.59460	1.47552	0.14443	12.39950	6.89900	1.79729	0.17014	2.00198	**0.04529**	2.00198	**0.04529**
350832	71	4	7.25	14.44295	10.39355	1.38961	0.16893	32.94819	7.67109	4.29511	0.14563	2.00168	**0.04532**	2.00168	**0.04532**
4357713	55	20	2.90	8.45181	5.25865	1.60722	0.11238	5.49913	4.26651	1.28891	0.21469	1.99993	**0.04551**	1.99993	**0.04551**
194735	71	4	2.25	20.85830	10.24940	2.03507	**0.04553**	30.14229	93.03498	0.32399	0.80054	1.59270	0.11123	1.99976	**0.04553**
4395096	48	27	4.19	-3.56369	4.91390	-0.72523	0.47066	6.01772	2.85405	2.10849	**0.04561**	0.90336	0.36633	1.99894	**0.04561**
36647	71	4	1.00	-20.82529	10.24151	-2.03342	**0.04570**	0.00000	0.00000	0.00000	1.00000	-1.41293	0.15768	-1.99818	**0.04570**
174571	64	11	2.82	-13.26388	6.53973	-2.02820	**0.04624**	-0.40650	7.51616	-0.05408	0.95819	-1.44647	0.14805	-1.99319	**0.04624**
4449055	60	15	2.53	-8.73611	5.83936	-1.49607	0.13900	-7.90382	5.57877	-1.41677	0.18198	-1.98992	**0.04660**	-1.98992	**0.04660**
841907	52	23	37.48	2.36605	5.14556	0.45982	0.64703	-4.66353	2.20072	-2.11909	**0.04679**	-1.08207	0.27922	-1.98817	**0.04679**
4436046	62	13	15.46	12.31058	6.08737	2.02232	**0.04686**	1.91411	3.65093	0.52428	0.61151	1.76458	0.07763	1.98757	**0.04686**
303326	71	4	2.25	20.79149	10.28947	2.02066	**0.04704**	-33.59815	22.26129	-1.50926	0.37253	0.77374	0.43908	1.98598	**0.04704**
2438203	43	32	7.66	-6.19707	4.73546	-1.30865	0.19481	-3.68890	2.37273	-1.55471	0.13086	-1.98498	**0.04715**	-1.98498	**0.04715**
4397092	57	18	5.94	9.43213	5.61595	1.67953	0.09738	5.07835	4.24984	1.19495	0.25066	1.98444	**0.04721**	1.98444	**0.04721**
182269	71	4	3.25	-9.42730	10.60116	-0.88927	0.37682	-15.60806	1.34625	-11.59377	0.05477	-1.98303	**0.04736**	-1.98303	**0.04736**
214036	39	36	11.44	-9.30230	4.61972	-2.01361	**0.04779**	0.65922	2.23043	0.29556	0.76942	-1.19226	0.23316	-1.97924	**0.04779**
157162	70	5	3.00	0.10857	9.48663	0.01144	0.99090	-5.79899	1.31705	-4.40302	**0.04791**	-1.39074	0.16431	-1.97820	**0.04791**
3544699	68	7	4.43	-15.92721	7.91541	-2.01218	**0.04794**	13.71178	11.27838	1.21576	0.29092	-0.65179	0.51454	-1.97787	**0.04794**
179267	45	30	6.03	-9.45764	4.70356	-2.01074	**0.04810**	-2.07545	2.67559	-0.77570	0.44467	-1.93806	0.05262	-1.97650	**0.04810**
191153	11	64	130.27	4.84967	6.67443	0.72660	0.46982	2.46685	1.22384	2.01566	**0.04825**	1.90774	0.05642	1.97519	**0.04825**
368236	67	8	2.13	-6.36010	7.63890	-0.83259	0.40783	-23.84230	9.24825	-2.57804	**0.04955**	-1.97392	**0.04839**	-1.97392	**0.04839**
43950	67	8	2.38	7.86195	7.69359	1.02188	0.31026	16.16711	7.23981	2.23309	0.07587	1.97272	**0.04853**	1.97272	**0.04853**
4379646	67	8	1.75	-14.97473	7.46609	-2.00570	**0.04865**	39.62625	27.01240	1.46696	0.20230	-0.49261	0.62229	-1.97167	**0.04865**
4111715	50	25	5.60	-7.61745	5.06027	-1.50534	0.13661	-3.26704	2.43879	-1.33961	0.19405	-1.97087	**0.04874**	-1.97087	**0.04874**
193672	43	32	6.75	9.31388	4.66087	1.99832	**0.04946**	-2.13200	2.68440	-0.79422	0.43352	0.83539	0.40350	1.96461	**0.04946**
185222	71	4	1.25	6.00101	10.50966	0.57100	0.56978	-25.09735	1.96561	-12.76820	**0.04976**	-0.98546	0.32440	-1.96204	**0.04976**
157327	31	44	5.41	-9.35015	4.68836	-1.99433	**0.04990**	1.08507	2.47003	0.43930	0.66275	-1.07811	0.28098	-1.96079	**0.04990**

[a] Statistics associated to the two part model for association analysis between the trait and OTUs adjusting for age and gender. The binomial analysis that tests for association of detecting a microbe represented by an OTU with the trait, where s.e. represents the standard error of the estimate, *t* -value of a *t* test for the estimate and its associated probability (*P* value). The quantitative analysis tests for association between the trait and the abundance of the OTU for the subjects where that OTU is present, where s.e. represents the standard error of the estimate, *t* -value of a *t* test for the estimate and its associated probability (*P* value). The Meta analysis combine the effect of both binary and quantitative analysis, a meta *P* value was derived using an unweighted Z method. The final association *P* value per OTU-trait pair was assigned from the minimum of *P* values from the binary analysis, quantitative analysis, and meta-analysis.

Table S4e. OTUs associated with Total cholesterol (TC) at FDR < 0.05 level [a]

	Summary of OTUs reads			Binary model				Quantitative model				Meta analysis		Final association	
OTU	No. Absent	No. Present	mean counts in presents	Estimate	s.e.	*t* value	*P* value	Estimate	s.e.	*t* value	*P* value	Meta z value	*Meta P* value	*z*	*P value*
207340	71	4	2.00	-1.97281	12.83521	-0.15370	0.87827	16.00000	0.00000	743.20468	**0.00000**	5.77705	**0.00000**	8.32315	**0.00000**
324015	70	5	2.40	-48.08228	10.17661	-4.72478	**0.00001**	-9.58050	30.00679	-0.31928	0.77978	-3.30522	**0.00095**	-4.39467	**0.00001**
389371	60	15	4.07	-28.67954	6.40380	-4.47852	**0.00003**	9.47758	4.06824	2.32965	**0.03809**	-1.49783	0.13418	-4.19209	**0.00003**
4342104	59	16	5.38	-9.60323	7.17084	-1.33921	0.18472	-15.22414	2.62694	-5.79538	**0.00006**	-3.76929	**0.00016**	-4.00420	**0.00006**
198754	70	5	2.80	-41.14252	10.49688	-3.91950	**0.00020**	-19.94406	7.10899	-2.80547	0.10704	-3.76924	**0.00016**	-3.76924	**0.00016**
3115852	65	10	2.20	25.95953	7.92211	3.27685	**0.00162**	16.36467	6.66430	2.45557	**0.04375**	3.65525	**0.00026**	3.65525	**0.00026**
361507	66	9	143.67	30.22423	8.12948	3.71785	**0.00039**	1.24020	1.94140	0.63882	0.54654	2.93215	**0.00337**	3.54373	**0.00039**
184248	62	13	4.38	-9.54302	7.54261	-1.26522	0.20987	15.20222	3.21518	4.72826	**0.00081**	1.48260	0.13818	3.35062	**0.00081**
180421	62	13	3.85	-24.58014	7.16158	-3.43222	**0.00100**	-1.87036	9.24480	-0.20231	0.84373	-2.46701	**0.01362**	-3.29175	**0.00100**
4347159	35	40	67.45	3.59414	5.77001	0.62290	0.53532	-5.15779	1.46392	-3.52328	**0.00115**	-1.85993	0.06290	-3.25025	**0.00115**
539647	60	15	19.27	-0.77631	7.21163	-0.10765	0.91458	7.98557	2.00165	3.98950	**0.00180**	2.13185	**0.03302**	3.12216	**0.00180**
760544	63	12	4.67	-21.43375	7.65218	-2.80100	**0.00654**	-12.16997	6.65957	-1.82744	0.10090	-3.08298	**0.00205**	-3.08298	**0.00205**
348304	53	22	3.86	-20.19467	6.35729	-3.17661	**0.00219**	9.33380	4.16605	2.24045	**0.03721**	-0.69228	0.48876	-3.06251	**0.00219**
194374	70	5	2.00	-19.76244	11.36347	-1.73912	0.08629	-62.98963	7.35475	-8.56448	**0.01336**	-2.96230	**0.00305**	-2.96230	**0.00305**
157424	48	27	265.70	-7.05658	5.98033	-1.17996	0.24190	3.60379	1.12006	3.21749	**0.00368**	1.22612	0.22015	2.90425	**0.00368**
196957	22	53	9.45	-9.89474	6.23576	-1.58677	0.11695	-6.68415	2.53652	-2.63517	**0.01117**	-2.90275	**0.00370**	-2.90275	**0.00370**
3236435	37	38	40.37	-3.70670	5.78750	-0.64047	0.52390	6.23049	2.04063	3.05322	**0.00431**	1.56798	0.11689	2.85480	**0.00431**
193654	59	16	4.25	-19.60743	6.70559	-2.92404	**0.00462**	-4.16101	6.95905	-0.59793	0.56016	-2.41495	**0.01574**	-2.83265	**0.00462**
740158	66	9	3.44	24.50788	8.39309	2.92001	**0.00467**	-2.62862	5.90241	-0.44535	0.67169	1.70067	0.08900	2.82895	**0.00467**
311718	71	4	3.25	17.87391	12.70858	1.40644	0.16389	-8.77365	0.06714	-130.66925	**0.00487**	-1.00642	0.31421	-2.81538	**0.00487**
4296763	70	5	102.80	-13.56549	11.47719	-1.18195	0.24111	-6.21280	0.44219	-14.05010	**0.00503**	-2.81250	**0.00492**	-2.81250	**0.00492**
4383922	69	6	2.00	-29.13657	10.10697	-2.88282	**0.00519**	-12.01821	8.30465	-1.44717	0.24366	-2.80066	**0.00510**	-2.80066	**0.00510**
177792	32	43	6.26	-4.36808	5.83940	-0.74803	0.45688	7.83040	2.64419	2.96136	**0.00513**	1.45284	0.14627	2.79862	**0.00513**
1044419	70	5	155.80	-31.41750	10.95516	-2.86783	**0.00542**	-2.25102	3.68229	-0.61131	0.60322	-2.33404	**0.01959**	-2.78105	**0.00542**
4464173	25	50	6.98	-16.60061	5.79693	-2.86369	**0.00548**	0.33690	2.37784	0.14168	0.88794	-1.86417	0.06230	-2.77725	**0.00548**
216599	20	55	35.02	0.88005	6.57290	0.13389	0.89386	-5.07634	1.76883	-2.86988	**0.00592**	-1.85159	0.06408	-2.75196	**0.00592**
174943	70	5	4.60	31.14306	11.00374	2.83023	**0.00602**	2.40381	5.20527	0.46180	0.68959	2.22447	**0.02612**	2.74646	**0.00602**
4469032	70	5	5.80	18.66595	11.36758	1.64203	0.10494	15.58326	2.46071	6.33283	**0.02404**	2.74205	**0.00611**	2.74205	**0.00611**
3318103	59	16	2.38	-0.09158	7.04933	-0.01299	0.98967	17.71583	5.44881	3.25132	**0.00631**	1.92203	0.05460	2.73111	**0.00631**
515299	70	5	14.60	-16.04482	11.40830	-1.40642	0.16390	-17.75530	2.09920	-8.45813	**0.01369**	-2.72754	**0.00638**	-2.72754	**0.00638**
199710	50	25	5.20	-10.74935	6.00832	-1.78908	0.07781	-7.43506	3.36680	-2.20834	**0.03794**	-2.71459	**0.00664**	-2.71459	**0.00664**
3907189	67	8	1.88	24.88858	8.90699	2.79428	**0.00666**	-10.76851	10.95066	-0.98337	0.37059	1.28548	0.19863	2.71331	**0.00666**
297385	68	7	2.57	-20.03704	9.74438	-2.05627	**0.04338**	-22.54206	9.36102	-2.40808	0.07371	-2.69297	**0.00708**	-2.69297	**0.00708**
4309301	13	62	21.26	20.07851	7.24730	2.77048	**0.00712**	-0.68737	1.72327	-0.39887	0.69143	1.62240	0.10472	2.69134	**0.00712**
196061	68	7	5.57	-27.06290	9.83047	-2.75296	**0.00747**	-7.10729	9.18274	-0.77398	0.48214	-2.38861	**0.01691**	-2.67515	**0.00747**
2157225	65	10	2.40	5.90157	8.45699	0.69783	0.48753	20.39533	5.57924	3.65557	**0.00812**	2.36271	**0.01814**	2.64713	**0.00812**
300235	60	15	1.60	-18.65437	6.86683	-2.71659	**0.00825**	6.39341	11.25001	0.56830	0.58031	-1.47683	0.13972	-2.64149	**0.00825**
1952	7	68	202.57	-1.18978	10.56638	-0.11260	0.91066	3.72990	1.36942	2.72370	**0.00828**	1.78768	0.07383	2.64037	**0.00828**
177758	64	11	4.82	-9.97263	8.06702	-1.23622	0.22039	-16.64450	5.15971	-3.22586	**0.01213**	-2.64017	**0.00829**	-2.64017	**0.00829**
851733	59	16	70.75	10.34719	6.93609	1.49179	0.14012	3.78745	1.48194	2.55573	**0.02393**	2.64002	**0.00829**	2.64002	**0.00829**
357930	64	11	110.91	-8.61168	8.09150	-1.06429	0.29075	-2.59847	0.74745	-3.47643	**0.00836**	-2.61170	**0.00901**	-2.63704	**0.00836**
184525	65	10	1.70	-20.70228	8.15344	-2.53909	**0.01328**	-23.53975	17.28289	-1.36203	0.21539	-2.62702	**0.00861**	-2.62702	**0.00861**
174516	43	32	22.66	11.25842	5.81506	1.93608	0.05678	4.51292	2.40327	1.87783	0.07050	2.62597	**0.00864**	2.62597	**0.00864**
34789	63	12	3.83	-20.33586	7.53596	-2.69851	**0.00867**	-8.24459	8.98557	-0.91754	0.38279	-2.47310	**0.01339**	-2.62472	**0.00867**
193477	38	37	5.78	-13.59704	5.58404	-2.43498	**0.01737**	-4.25673	3.14968	-1.35148	0.18547	-2.61832	**0.00884**	-2.61832	**0.00884**
182797	69	6	2.83	-26.09489	10.39567	-2.51017	**0.01432**	-57.03296	35.94635	-1.58661	0.21079	-2.61675	**0.00888**	-2.61675	**0.00888**
197581	69	6	2.00	-2.60991	10.62638	-0.24561	0.80669	11.03465	1.82183	6.05692	**0.00903**	1.67318	0.09429	2.61094	**0.00903**
105287	64	11	3.09	-20.87598	7.93603	-2.63053	**0.01042**	-9.78927	8.15517	-1.20038	0.26433	-2.60060	**0.00931**	-2.60060	**0.00931**
349123	70	5	2.60	-29.52283	11.04806	-2.67222	**0.00931**	6.15478	20.66671	0.29781	0.79394	-1.65401	0.09813	-2.60033	**0.00931**
194654	58	17	3.06	-4.01863	6.94065	-0.57900	0.56440	-10.42257	3.46151	-3.01099	**0.00935**	-2.24539	**0.02474**	-2.59914	**0.00935**
315223	62	13	6.62	-6.95411	7.58096	-0.91731	0.36204	-15.52809	4.87290	-3.18662	**0.00971**	-2.47309	**0.01339**	-2.58600	**0.00971**
4479443	65	10	2.90	8.68126	8.42458	1.03047	0.30624	19.09898	5.43480	3.51420	**0.00980**	2.54970	**0.01078**	2.58267	**0.00980**
182289	34	41	10.37	-10.07184	5.67096	-1.77604	0.07995	-4.25545	2.19417	-1.93944	0.05990	-2.56857	**0.01021**	-2.56857	**0.01021**
179267	45	30	6.03	-14.79331	5.62719	-2.62890	**0.01047**	-2.12136	3.17941	-0.66722	0.51029	-2.27578	**0.02286**	-2.56006	**0.01047**
195436	37	38	4.76	-14.56108	5.54448	-2.62623	**0.01054**	3.81049	2.75232	1.38446	0.17498	-0.84938	0.39567	-2.55758	**0.01054**
2046330	67	8	5.88	23.42965	8.92596	2.62489	**0.01058**	0.76831	6.65277	0.11549	0.91255	1.88525	0.05940	2.55633	**0.01058**
519880	70	5	3.80	-29.37026	11.20750	-2.62059	**0.01070**	9.04333	50.96898	0.17743	0.87552	-1.69399	0.09027	-2.55233	**0.01070**
320786	67	8	3.38	-23.40178	8.93187	-2.62003	**0.01072**	4.41092	4.92304	0.89598	0.41132	-1.22346	0.22115	-2.55181	**0.01072**
158625	70	5	2.80	-29.03759	11.08341	-2.61991	**0.01072**	-14.53078	15.66249	-0.92774	0.45148	-2.33674	**0.01945**	-2.55170	**0.01072**
591118	71	4	3.25	-4.44682	12.88000	-0.34525	0.73091	-32.64721	0.56302	-57.98563	**0.01098**	-2.04164	**0.04119**	-2.54340	**0.01098**
4379646	67	8	1.75	-23.28756	8.93797	-2.60547	**0.01114**	57.52187	41.02106	1.40225	0.21977	-0.92709	0.35388	-2.53824	**0.01114**
197179	69	6	3.83	26.47442	10.18549	2.59923	**0.01133**	-5.49181	6.26266	-0.87691	0.44509	1.25073	0.21103	2.53243	**0.01133**
562410	69	6	1.50	-16.97317	10.48403	-1.61896	0.10983	-21.25801	6.65907	-3.19234	**0.04962**	-2.51883	**0.01177**	-2.51883	**0.01177**
3946926	69	6	3.17	26.43685	10.27261	2.57353	**0.01213**	2.51636	14.91550	0.16871	0.87676	1.88341	0.05965	2.50846	**0.01213**
187517	61	14	14.14	18.11422	7.08718	2.55591	**0.01270**	1.63510	4.48943	0.36421	0.72261	2.01313	**0.04410**	2.49202	**0.01270**
197696	68	7	3.71	1.59393	9.97400	0.15981	0.87348	16.72011	3.89366	4.29418	**0.01270**	1.87468	0.06084	2.49196	**0.01270**
4318284	69	6	28.83	7.15038	10.60342	0.67435	0.50225	-7.90968	1.48529	-5.32533	**0.01294**	-1.28305	0.19947	-2.48546	**0.01294**
4457268	67	8	4.25	10.25587	9.33144	1.09907	0.27540	17.21897	4.76907	3.61055	**0.01537**	2.48493	**0.01296**	2.48493	**0.01296**
183598	70	5	2.60	-26.16156	11.14316	-2.34777	**0.02164**	-13.96977	8.30400	-1.68229	0.23454	-2.46456	**0.01372**	-2.46456	**0.01372**
194488	71	4	2.50	-31.33784	12.42411	-2.52234	**0.01387**	-39.29670	53.09788	-0.74008	0.59440	-2.11645	**0.03431**	-2.46064	**0.01387**
4405104	39	36	18.58	-9.66556	5.65975	-1.70777	0.09199	-4.07045	2.19556	-1.85395	0.07270	-2.46052	**0.01387**	-2.46052	**0.01387**
165257	71	4	4.75	1.57965	12.86550	0.12278	0.90262	-9.79677	0.21849	-44.83877	**0.01420**	-1.64751	0.09945	-2.45227	**0.01420**
113654	67	8	2.88	-17.78722	9.10487	-1.95359	0.05463	-32.65153	17.61453	-1.85367	0.12297	-2.44956	**0.01430**	-2.44956	**0.01430**
193053	18	57	11.40	16.12775	6.47972	2.48896	**0.01512**	0.34524	2.22496	0.15517	0.87727	1.82704	0.06769	2.42939	**0.01512**
189647	63	12	3.33	-18.87538	7.59093	-2.48657	**0.01522**	6.55902	6.42973	1.02011	0.33431	-1.03357	0.30134	-2.42715	**0.01522**
4369434	69	6	4.00	-4.39919	10.66674	-0.41242	0.68126	-24.43354	4.91368	-4.97255	**0.01563**	-1.99989	**0.04551**	-2.41753	**0.01563**
189356	67	8	2.50	7.63049	9.46697	0.80601	0.42289	-19.53090	5.43857	-3.59118	**0.01569**	-1.14174	0.25356	-2.41608	**0.01569**

Table S4e. OTUs associated with Total cholesterol (TC) at FDR < 0.05 level [a] (Cont.)

	Summary of OTUs reads			Binary model				Quantitative model				Meta analysis		Final association	
OTU	No. Absent	No. Present	mean counts in presents	Estimate	s.e.	*t* value	*P* value	Estimate	s.e.	*t* value	*P* value	Meta z value	*Meta P* value	*z*	*P value*
178478	34	41	4.98	-9.75791	5.74026	-1.69991	0.09346	-4.65850	2.61399	-1.78214	0.08272	-2.41302	**0.01582**	-2.41302	**0.01582**
192676	69	6	3.83	-1.19380	10.74543	-0.11110	0.91185	-18.54316	3.75226	-4.94186	**0.01589**	-1.78337	0.07453	-2.41135	**0.01589**
337161	62	13	9.46	-1.49092	7.61805	-0.19571	0.84539	10.84224	3.74320	2.89652	**0.01593**	1.56663	0.11720	2.41055	**0.01593**
179201	52	23	5.70	-14.85216	6.02522	-2.46500	**0.01609**	-3.88865	4.59863	-0.84561	0.40777	-2.28731	**0.02218**	-2.40693	**0.01609**
2949328	63	12	3.42	0.17037	7.87047	0.02165	0.98279	16.75681	5.68582	2.94712	**0.01630**	1.71386	0.08655	2.40220	**0.01630**
808794	71	4	3.25	-8.64693	12.80201	-0.67544	0.50156	-30.73374	0.78940	-38.93293	**0.01635**	-2.17300	**0.02978**	-2.40105	**0.01635**
315982	71	4	2.25	30.30829	12.36861	2.45042	**0.01670**	5.55113	16.90290	0.32841	0.79799	1.87326	0.06103	2.39325	**0.01670**
181452	66	9	3.00	-20.92043	8.56771	-2.44178	**0.01707**	5.20614	14.34388	0.36295	0.72908	-1.44164	0.14940	-2.38513	**0.01707**
191928	65	10	1.80	-19.92159	8.17091	-2.43811	**0.01723**	1.37879	9.79716	0.14073	0.89204	-1.58814	0.11225	-2.38169	**0.01723**
925131	69	6	76.17	-24.89420	10.21799	-2.43631	**0.01731**	6.67263	6.50091	1.02641	0.38022	-1.06244	0.28804	-2.38000	**0.01731**
188262	71	4	1.00	31.16998	12.80096	2.43497	**0.01737**	0.00000	0.00000	0.00000	1.00000	1.68203	0.09256	2.37874	**0.01737**
191874	48	27	3.07	-4.74986	6.03417	-0.78716	0.43377	-12.60701	4.95984	-2.54182	**0.01790**	-2.22767	**0.02590**	-2.36765	**0.01790**
341730	67	8	2.50	-19.24887	9.07267	-2.12163	**0.03731**	-16.89469	11.63935	-1.45151	0.20635	-2.36599	**0.01798**	-2.36599	**0.01798**
175761	58	17	2.41	-0.98717	6.90710	-0.14292	0.88675	-20.60406	7.71999	-2.66892	**0.01834**	-1.76856	0.07697	-2.35871	**0.01834**
310633	71	4	10.00	-16.15987	12.75873	-1.26657	0.20939	-14.71017	0.87027	-16.90301	**0.03762**	-2.35765	**0.01839**	-2.35765	**0.01839**
4094866	57	18	5.89	2.88451	6.74686	0.42753	0.67027	-10.35846	3.92012	-2.64238	**0.01847**	-1.36491	0.17228	-2.35605	**0.01847**
158423	60	15	79.60	-7.56135	7.16915	-1.05471	0.29509	5.86865	2.15747	2.72015	**0.01860**	0.92375	0.35562	2.35340	**0.01860**
190913	30	45	9.82	-0.54321	6.14181	-0.08844	0.92977	5.70763	2.33971	2.43946	**0.01901**	1.59603	0.11048	2.34526	**0.01901**
329798	65	10	5.80	19.58336	8.16403	2.39874	**0.01904**	4.03780	5.81663	0.69418	0.50994	2.12387	**0.03368**	2.34468	**0.01904**
730906	53	22	2.91	-14.87998	6.23168	-2.38780	**0.01958**	-3.79768	4.93909	-0.76890	0.45140	-2.18316	**0.02902**	-2.33438	**0.01958**
176108	64	11	2.18	-18.89827	7.92605	-2.38432	**0.01975**	15.00937	12.48698	1.20200	0.26373	-0.85807	0.39085	-2.33111	**0.01975**
187196	71	4	2.25	-29.40984	12.35899	-2.37963	**0.01998**	0.83969	16.15505	0.05198	0.96694	-1.61592	0.10611	-2.32670	**0.01998**
178151	71	4	3.25	-18.91848	12.64449	-1.49618	0.13898	-8.37890	0.93065	-9.00329	0.07042	-2.32554	**0.02004**	-2.32554	**0.02004**
851323	47	28	57.61	-10.69397	5.84376	-1.82998	0.07139	-3.58044	2.33293	-1.53474	0.13741	-2.32531	**0.02006**	-2.32531	**0.02006**
197988	42	33	14.00	-13.28355	5.59736	-2.37318	**0.02031**	0.26346	2.46296	0.10697	0.91552	-1.56592	0.11737	-2.32062	**0.02031**
182033	56	19	3.68	-10.33662	6.77714	-1.52522	0.13159	-10.26423	5.41752	-1.89464	0.07636	-2.31937	**0.02037**	-2.31937	**0.02037**
288651	26	49	32.88	-3.49789	6.05907	-0.57730	0.56554	-3.63627	1.51606	-2.39849	**0.02057**	-2.04377	**0.04098**	-2.31570	**0.02057**
176077	66	9	2.67	20.21362	8.55052	2.36402	**0.02078**	0.02050	8.06401	0.00254	0.99805	1.63655	0.10172	2.31199	**0.02078**
3141342	60	15	4.33	4.69473	7.28299	0.64462	0.52122	-17.84493	6.70793	-2.66027	**0.02078**	-1.18121	0.23752	-2.31194	**0.02078**
553080	28	47	17.00	-13.61482	5.77471	-2.35766	**0.02111**	2.65357	2.38546	1.11240	0.27201	-0.85387	0.39318	-2.30600	**0.02111**
4311621	67	8	2.50	21.26075	9.04133	2.35151	**0.02144**	-7.55005	5.96474	-1.26578	0.26137	0.83228	0.40525	2.30019	**0.02144**
4438999	61	14	3.93	0.51318	7.40209	0.06933	0.94492	-14.33736	5.35490	-2.67743	**0.02151**	-1.57676	0.11485	-2.29897	**0.02151**
180107	61	14	3.93	0.51318	7.40209	0.06933	0.94492	-14.33736	5.35490	-2.67743	**0.02151**	-1.57676	0.11485	-2.29897	**0.02151**
180312	51	24	4.92	-14.01917	5.97179	-2.34757	**0.02165**	-0.93061	4.63320	-0.20086	0.84274	-1.76413	0.07771	-2.29648	**0.02165**
3709990	63	12	3.08	-10.95802	7.93429	-1.38110	0.17152	-14.01236	6.54866	-2.13973	0.06105	-2.29137	**0.02194**	-2.29137	**0.02194**
302333	63	12	2.50	-8.23645	7.82857	-1.05210	0.29627	-24.60621	9.48717	-2.59363	**0.02904**	-2.28212	**0.02248**	-2.28212	**0.02248**
303320	70	5	2.40	26.46978	11.35304	2.33151	**0.02253**	-1.87917	6.32334	-0.29718	0.79435	1.42883	0.15305	2.28133	**0.02253**
2996838	46	29	9.14	-5.81087	5.96127	-0.97477	0.33294	-5.04090	2.10231	-2.39779	**0.02397**	-2.28103	**0.02255**	-2.28103	**0.02255**
182577	43	32	3.63	-10.88096	5.68850	-1.91280	0.05975	-5.11298	3.72207	-1.37369	0.18006	-2.27914	**0.02266**	-2.27914	**0.02266**
4462107	69	6	2.67	5.11967	10.61726	0.48220	0.63112	-38.83900	9.02298	-4.30445	**0.02308**	-1.26715	0.20510	-2.27216	**0.02308**
197970	27	48	8.15	-13.52053	5.84982	-2.31127	**0.02368**	-0.51752	2.45194	-0.21107	0.83379	-1.74802	0.08046	-2.26222	**0.02368**
189478	66	9	1.44	-9.63102	8.81755	-1.09226	0.27836	-25.35312	9.30843	-2.72367	**0.03447**	-2.26167	**0.02372**	-2.26167	**0.02372**
176947	69	6	3.83	-22.05129	10.38990	-2.12238	**0.03725**	-8.58882	6.27766	-1.36816	0.26473	-2.26157	**0.02372**	-2.26157	**0.02372**
2774254	69	6	13.50	-19.75752	10.37282	-1.90474	0.06081	-11.84969	6.94798	-1.70549	0.18665	-2.25952	**0.02385**	-2.25952	**0.02385**
4060501	46	29	13.69	-0.25794	5.95116	-0.04334	0.96555	-7.63167	3.19898	-2.38566	**0.02462**	-1.61961	0.10532	-2.24728	**0.02462**
186687	60	15	2.60	-16.02744	6.98471	-2.29465	**0.02467**	-4.00832	5.98827	-0.66936	0.51594	-2.04788	**0.04057**	-2.24651	**0.02467**
180352	70	5	4.00	-25.62911	11.17757	-2.29291	**0.02478**	-7.47438	13.30091	-0.56194	0.63073	-1.92727	0.05395	-2.24487	**0.02478**
4306262	33	42	261.55	-8.97558	5.71544	-1.57041	0.12070	-2.40477	1.45076	-1.65759	0.10542	-2.24219	**0.02495**	-2.24219	**0.02495**
294791	69	6	2.67	-4.76407	10.63079	-0.44814	0.65540	-26.37107	6.31319	-4.17714	**0.02499**	-1.90058	0.05736	-2.24155	**0.02499**
4398588	63	12	4.00	17.40154	7.61729	2.28448	**0.02529**	-3.42502	6.29547	-0.54405	0.59963	1.21054	0.22607	2.23690	**0.02529**
322835	38	37	7.84	-12.75940	5.58732	-2.28363	**0.02535**	4.54343	3.07070	1.47961	0.14819	-0.55870	0.57636	-2.23610	**0.02535**
125270	61	14	3.50	4.83975	7.42466	0.65185	0.51657	-7.48035	2.89493	-2.58395	**0.02541**	-1.12180	0.26195	-2.23510	**0.02541**
1679707	62	13	3.23	-16.80224	7.37182	-2.27925	**0.02562**	6.08068	5.86905	1.03606	0.32458	-0.88166	0.37796	-2.23195	**0.02562**
3609545	45	30	6.00	-13.10806	5.76529	-2.27362	**0.02597**	-0.73576	3.90986	-0.18818	0.85214	-1.70626	0.08796	-2.22662	**0.02597**
305318	37	38	6.50	-12.69619	5.58487	-2.27332	**0.02599**	3.92794	2.67778	1.46686	0.15134	-0.55970	0.57569	-2.22634	**0.02599**
4483570	71	4	5.25	-26.81850	12.48823	-2.14750	**0.03512**	-16.22480	8.19172	-1.98064	0.29765	-2.22629	**0.02599**	-2.22629	**0.02599**
3138798	31	44	219.93	-9.75340	5.74307	-1.69829	0.09377	-1.70766	1.13815	-1.50039	0.14118	-2.22545	**0.02605**	-2.22545	**0.02605**
337538	67	8	1.63	-10.21361	9.29994	-1.09824	0.27576	-15.46566	5.63557	-2.74430	**0.04058**	-2.21866	**0.02651**	-2.21866	**0.02651**
189450	66	9	2.44	-19.58847	8.65648	-2.26287	**0.02666**	-4.69062	7.99431	-0.58674	0.57877	-1.95984	0.05001	-2.21645	**0.02666**
193534	68	7	2.43	5.45579	9.89320	0.55147	0.58302	31.33216	9.18755	3.41029	**0.02702**	1.95176	0.05097	2.21122	**0.02702**
346253	69	6	2.67	-13.78278	10.62288	-1.29746	0.19861	-28.64492	10.13936	-2.82512	0.06646	-2.20677	**0.02733**	-2.20677	**0.02733**
189007	67	8	2.00	-12.47547	9.24722	-1.34910	0.18153	-18.99121	8.47508	-2.24083	0.07514	-2.20312	**0.02759**	-2.20312	**0.02759**
174818	20	55	10.71	4.87704	6.50307	0.74996	0.45572	-4.89853	2.16583	-2.26173	**0.02792**	-1.02705	0.30440	-2.19838	**0.02792**
175922	69	6	2.67	23.03253	10.29373	2.23753	**0.02835**	3.60844	14.18708	0.25435	0.81567	1.71514	0.08632	2.19245	**0.02835**
4442459	63	12	4.58	-17.02474	7.61727	-2.23502	**0.02852**	4.32539	5.06654	0.85372	0.41541	-0.97273	0.33069	-2.19007	**0.02852**
4347652	70	5	2.20	-25.13730	11.32236	-2.22015	**0.02956**	-16.47632	15.92920	-1.03435	0.40965	-2.12165	**0.03387**	-2.17597	**0.02956**
4429981	61	14	38.07	10.86885	7.29292	1.49033	0.14051	-8.66154	3.46908	-2.49678	**0.02968**	-0.49532	0.62038	-2.17439	**0.02968**
4103317	17	58	56.57	14.76989	6.66558	2.21584	**0.02986**	-0.33087	1.45545	-0.22733	0.82101	1.37578	0.16889	2.17189	**0.02986**
187126	57	18	3.22	-14.46392	6.53409	-2.21361	**0.03002**	7.24551	3.76510	1.92439	0.07349	-0.26869	0.78817	-2.16977	**0.03002**
552235	68	7	4.57	-21.10516	9.59864	-2.19877	**0.03111**	-0.91157	8.35234	-0.10914	0.91835	-1.59679	0.11031	-2.15568	**0.03111**
297266	67	8	7.38	-11.19396	9.25280	-1.20979	0.23032	7.97933	2.69040	2.96585	**0.03131**	0.67430	0.50012	2.15315	**0.03131**
180552	60	15	4.27	-9.69567	7.13150	-1.35956	0.17821	-12.93708	7.00468	-1.84692	0.08955	-2.15246	**0.03136**	-2.15246	**0.03136**
157631	71	4	11.25	-27.34829	12.46407	-2.19417	**0.03145**	11.09732	10.97248	1.01138	0.49640	-1.04026	0.29822	-2.15132	**0.03145**
307113	63	12	1.67	-16.66465	7.62580	-2.18530	**0.03212**	-6.04089	11.28364	-0.53537	0.60537	-1.88061	0.06002	-2.14290	**0.03212**
4045882	63	12	1.67	-16.66465	7.62580	-2.18530	**0.03212**	-6.04089	11.28364	-0.53537	0.60537	-1.88061	0.06002	-2.14290	**0.03212**
275237	42	33	229.64	10.93765	5.66604	1.93039	0.05750	1.64449	1.43445	1.14642	0.26068	2.13850	**0.03248**	2.13850	**0.03248**
1607319	64	11	1.27	-11.67123	8.26564	-1.41202	0.16225	-10.63693	5.80475	-1.83245	0.10424	-2.13698	**0.03260**	-2.13698	**0.03260**

Table S4e. OTUs associated with Total cholesterol (TC) at FDR < 0.05 level [a] (Cont.)

	Summary of OTUs reads			Binary model				Quantitative model				Meta analysis		Final association	
OTU	No. Absent	No. Present	mean counts in presents	Estimate	s.e.	*t* value	*P* value	Estimate	s.e.	*t* value	*P* value	Meta z value	*Meta P* value	*z*	*P value*
181756	70	5	4.20	-11.92971	11.48241	-1.03896	0.30230	-13.00165	2.90890	-4.46961	**0.04659**	-2.13656	**0.03263**	-2.13656	**0.03263**
4411875	67	8	2.75	-0.24781	9.36333	-0.02647	0.97896	14.19238	4.84527	2.92912	**0.03267**	1.49185	0.13574	2.13616	**0.03267**
842193	51	24	70.96	-13.17286	6.06049	-2.17356	**0.03303**	1.00164	2.28998	0.43740	0.66628	-1.20243	0.22920	-2.13175	**0.03303**
180468	12	63	15.57	-4.74880	7.84952	-0.60498	0.54709	4.65638	2.13631	2.17964	**0.03322**	1.07996	0.28016	2.12942	**0.03322**
4414420	71	4	1.75	5.86402	12.85548	0.45615	0.64966	15.23606	0.81136	18.77853	**0.03387**	1.82141	0.06854	2.12162	**0.03387**
4020046	64	11	7.64	-6.76508	8.16074	-0.82898	0.40986	14.05703	5.50812	2.55206	**0.03407**	0.91581	0.35977	2.11930	**0.03407**
272886	71	4	2.25	-14.01511	12.74402	-1.09974	0.27511	45.97852	2.48434	18.50736	**0.03436**	0.72435	0.46885	2.11577	**0.03436**
196139	69	6	2.33	1.18886	10.73568	0.11074	0.91213	67.78870	18.35715	3.69277	**0.03445**	1.57339	0.11563	2.11476	**0.03445**
258099	64	11	4.64	-17.08436	7.93939	-2.15185	**0.03476**	1.02887	6.38459	0.16115	0.87597	-1.38241	0.16685	-2.11110	**0.03476**
4381555	62	13	3.92	-11.30755	7.70170	-1.46819	0.14641	-7.00063	4.20287	-1.66568	0.12675	-2.10675	**0.03514**	-2.10675	**0.03514**
4393532	60	15	5.53	-15.04464	7.00762	-2.14690	**0.03517**	-0.64027	4.78001	-0.13395	0.89567	-1.58217	0.11361	-2.10639	**0.03517**
194320	44	31	3.90	12.18770	5.67925	2.14600	**0.03524**	-3.09034	4.04773	-0.76348	0.45157	0.95653	0.33881	2.10554	**0.03524**
187569	64	11	2.36	-16.91932	7.90854	-2.13937	**0.03580**	5.96508	6.78705	0.87889	0.40508	-0.89566	0.37043	-2.09923	**0.03580**
4466707	63	12	14.75	-13.78011	7.77887	-1.77148	0.08071	-5.95251	4.55424	-1.30703	0.22361	-2.09555	**0.03612**	-2.09555	**0.03612**
2438203	43	32	7.66	-8.15308	5.75984	-1.41550	0.16123	-4.55476	2.83458	-1.60685	0.11892	-2.09322	**0.03633**	-2.09322	**0.03633**
558839	70	5	1.80	2.79406	11.55793	0.24174	0.80967	16.82027	3.29738	5.10110	**0.03635**	1.65030	0.09888	2.09301	**0.03635**
189292	64	11	6.00	-16.88374	7.93858	-2.12680	**0.03686**	2.58791	5.68822	0.45496	0.66123	-1.16605	0.24359	-2.08726	**0.03686**
178713	63	12	34.50	0.15867	7.87168	0.02016	0.98397	-7.21391	2.95424	-2.44188	**0.03725**	-1.45871	0.14464	-2.08302	**0.03725**
183576	66	9	3.56	-14.18300	8.73528	-1.62365	0.10882	-10.67633	7.06943	-1.51021	0.18173	-2.07815	**0.03770**	-2.07815	**0.03770**
183604	65	10	3.50	-17.47602	8.25768	-2.11634	**0.03777**	2.44284	4.76861	0.51227	0.62422	-1.12248	0.26166	-2.07730	**0.03777**
192912	68	7	4.00	-16.15085	9.73008	-1.65989	0.10129	-9.38830	6.06632	-1.54761	0.19663	-2.07173	**0.03829**	-2.07173	**0.03829**
157162	70	5	3.00	1.88571	11.55943	0.16313	0.87087	-13.34827	2.69467	-4.95358	**0.03842**	-1.34901	0.17733	-2.07035	**0.03842**
556126	63	12	4.17	6.00632	7.83474	0.76663	0.44581	13.77797	5.70122	2.41667	**0.03882**	2.00004	**0.04550**	2.06607	**0.03882**
182911	39	36	7.17	-7.27268	5.71367	-1.27286	0.20716	-4.67100	2.76136	-1.69156	0.10015	-2.05451	**0.03993**	-2.05451	**0.03993**
2497335	29	46	20.37	4.54098	5.89824	0.76989	0.44389	3.92621	1.85492	2.11664	**0.04012**	1.99277	**0.04629**	2.05255	**0.04012**
535955	64	11	2.36	1.39975	8.16458	0.17144	0.86436	15.94772	6.52342	2.44469	**0.04027**	1.57106	0.11617	2.05098	**0.04027**
4381430	38	37	3.92	-11.74236	5.62697	-2.08680	**0.04045**	-0.21595	2.98657	-0.07231	0.94278	-1.49972	0.13369	-2.04915	**0.04045**
178331	70	5	3.80	-24.02767	11.53625	-2.08280	**0.04082**	29.46669	27.34630	1.07754	0.39394	-0.84346	0.39897	-2.04533	**0.04082**
184342	68	7	1.86	-20.17123	9.68574	-2.08257	**0.04084**	26.44693	12.40327	2.13225	0.09995	-0.28287	0.77728	-2.04511	**0.04084**
208739	32	43	3.77	-1.25038	5.86674	-0.21313	0.83183	-5.20414	2.46283	-2.11308	**0.04088**	-1.59600	0.11049	-2.04472	**0.04088**
179384	68	7	1.86	20.03844	9.63435	2.07990	**0.04110**	-2.33202	9.92321	-0.23501	0.82574	1.28863	0.19753	2.04256	**0.04110**
185607	60	15	2.07	-14.56056	7.00931	-2.07732	**0.04134**	10.93694	9.99470	1.09427	0.29531	-0.70256	0.48233	-2.04010	**0.04134**
173965	60	15	2.40	-14.53509	7.00583	-2.07471	**0.04159**	6.87750	7.48061	0.91938	0.37601	-0.81483	0.41517	-2.03762	**0.04159**
187883	64	11	2.45	-12.67475	8.09080	-1.56656	0.12160	-10.71882	7.34360	-1.45962	0.18252	-2.03726	**0.04162**	-2.03726	**0.04162**
189384	71	4	1.00	26.45629	12.78350	2.06956	**0.04208**	0.00000	0.00000	0.00000	1.00000	1.43734	0.15062	2.03270	**0.04208**
4425512	71	4	3.50	-1.33977	12.86587	-0.10413	0.91735	-20.25496	1.34915	-15.01311	**0.04234**	-1.50891	0.13132	-2.03015	**0.04234**
4388068	69	6	18.83	14.29651	10.51443	1.35970	0.17817	4.30248	2.05826	2.09035	0.12773	2.02906	**0.04245**	2.02906	**0.04245**
4326573	70	5	11.00	21.09246	11.31825	1.86358	0.06646	11.41105	8.27877	1.37835	0.30203	2.02756	**0.04261**	2.02756	**0.04261**
573061	69	6	3.50	4.13811	10.63443	0.38912	0.69833	50.63745	14.95752	3.38542	**0.04292**	1.70559	0.08808	2.02449	**0.04292**
110192	60	15	20.60	-5.24261	7.20199	-0.72794	0.46901	-7.44671	3.29346	-2.26106	**0.04313**	-1.94211	0.05212	-2.02247	**0.04313**
178799	69	6	4.00	-2.04063	10.64219	-0.19175	0.84848	-7.03765	2.09034	-3.36675	**0.04352**	-1.56256	0.11816	-2.01873	**0.04352**
177024	70	5	9.40	-3.92276	11.62042	-0.33757	0.73667	-23.33428	5.03988	-4.62993	**0.04362**	-1.66452	0.09601	-2.01772	**0.04362**
4337970	57	18	10.00	0.08319	6.75276	0.01232	0.99020	-9.14735	4.15568	-2.20117	**0.04380**	-1.41686	0.15652	-2.01602	**0.04380**
4473509	24	51	20.41	-2.59374	6.17925	-0.41975	0.67592	4.22386	2.04320	2.06728	**0.04412**	1.12775	0.25942	2.01292	**0.04412**
16054	56	19	19.47	-6.28479	6.59098	-0.95354	0.34350	-4.17527	2.04062	-2.04608	0.05755	-2.01270	**0.04415**	-2.01270	**0.04415**
134671	68	7	4.43	-7.67474	9.87713	-0.77702	0.43969	-12.37322	4.27745	-2.89267	**0.04444**	-1.96759	**0.04912**	-2.00988	**0.04444**
663500	55	20	69.10	7.53020	6.46113	1.16546	0.24768	4.05092	2.27884	1.77762	0.09336	2.00390	**0.04508**	2.00390	**0.04508**
4364405	41	34	10.06	3.01978	5.90734	0.51119	0.61078	-7.13128	3.41718	-2.08689	**0.04521**	-1.05621	0.29087	-2.00266	**0.04521**
4354103	66	9	34.22	17.57991	8.63193	2.03661	**0.04537**	-0.37822	2.63523	-0.14352	0.89057	1.31781	0.18757	2.00123	**0.04537**
340960	69	6	20.83	11.35225	10.57778	1.07322	0.28676	8.46282	3.20694	2.63891	0.07773	2.00060	**0.04544**	2.00060	**0.04544**
110060	70	5	2.00	-23.16109	11.38075	-2.03511	**0.04552**	30.28886	31.91999	0.94890	0.44283	-0.87142	0.38353	-1.99980	**0.04552**
4370025	59	16	7.63	-7.86759	7.16301	-1.09836	0.27571	-6.38082	3.39897	-1.87728	0.08310	-1.99616	**0.04592**	-1.99616	**0.04592**
4294457	64	11	4.64	4.62176	8.30712	0.55636	0.57969	11.05265	4.68512	2.35910	**0.04602**	1.80243	0.07148	1.99518	**0.04602**
33112	70	5	3.60	-4.08595	11.90918	-0.34309	0.73253	18.01051	4.00461	4.49745	**0.04605**	1.16896	0.24242	1.99493	**0.04605**
367946	69	6	7.50	3.32992	10.94231	0.30432	0.76176	-24.09126	7.32276	-3.28992	**0.04608**	-1.19606	0.23167	-1.99465	**0.04608**
193968	17	58	13.78	3.02549	6.87911	0.43981	0.66139	-3.93056	1.92907	-2.03755	**0.04642**	-1.09855	0.27196	-1.99158	**0.04642**
4458227	66	9	16.11	-5.55195	8.85201	-0.62720	0.53251	-11.58563	4.63551	-2.49932	**0.04657**	-1.84863	0.06451	-1.99018	**0.04657**
4358921	59	16	1.69	-13.86867	6.85693	-2.02258	**0.04683**	1.15544	9.29572	0.12430	0.90298	-1.31940	0.18703	-1.98782	**0.04683**
4318177	67	8	268.38	-18.39584	9.10755	-2.01984	**0.04712**	0.85841	0.90988	0.94343	0.38878	-0.79434	0.42700	-1.98520	**0.04712**
365717	62	13	3.69	-15.05745	7.46295	-2.01763	**0.04736**	5.30989	5.49118	0.96699	0.35636	-0.75006	0.45322	-1.98308	**0.04736**
367814	54	21	2.24	-12.67384	6.28268	-2.01727	**0.04740**	13.76642	6.70365	2.05357	0.05484	-0.04426	0.96470	-1.98274	**0.04740**
146554	20	55	91.85	4.17387	6.51845	0.64032	0.52400	-3.50296	1.72831	-2.02681	**0.04782**	-0.94876	0.34274	-1.97894	**0.04782**
175520	69	6	2.33	-21.19602	10.53273	-2.01240	**0.04792**	6.09738	11.16824	0.54596	0.62308	-1.05117	0.29318	-1.97808	**0.04792**
183579	57	18	3.67	-10.18264	6.70845	-1.51788	0.13342	8.83997	4.10929	2.15122	**0.04816**	0.33600	0.73687	1.97592	**0.04816**
182653	70	5	8.80	-22.82301	11.35417	-2.01010	**0.04817**	11.78824	3.10277	3.79927	0.06282	-0.08162	0.93495	-1.97588	**0.04817**
177520	44	31	6.06	-11.48136	5.71953	-2.00740	**0.04846**	3.50370	3.56412	0.98305	0.33400	-0.71221	0.47634	-1.97330	**0.04846**
192127	38	37	4.57	-11.25158	5.61487	-2.00389	**0.04884**	1.35493	3.04026	0.44566	0.65867	-1.08059	0.27988	-1.96994	**0.04884**
326482	30	45	534.71	11.48164	5.73014	2.00373	**0.04886**	-0.23410	0.83908	-0.27899	0.78162	1.19684	0.23137	1.96979	**0.04886**
192015	64	11	2.09	-12.80557	8.07239	-1.58634	0.11704	14.61068	6.32559	2.30977	**0.04971**	0.27943	0.77991	1.96248	**0.04971**

[a] Statistics associated to the two part model for association analysis between the trait and OTUs adjusting for age and gender. The binomial analysis that tests for association of detecting a microbe represented by an OTU with the trait, where s.e. represents the standard error of the estimate, *t* -value of a *t* test for the estimate and its associated probability (*P* value). The quantitative analysis tests for association between the trait and the abundance of the OTU for the subjects where that OTU is present, where s.e. represents the standard error of the estimate, *t* -value of a *t* test for the estimate and its associated probability (*P* value). The Meta analysis combine the effect of both binary and quantitative analysis, a meta *P* value was derived using an unweighted Z method. The final association *P* value per OTU-trait pair was assigned from the minimum of *P* values from the binary analysis, quantitative analysis, and meta-analysis.
