# Supplementary material for: Intestinal Microbiota Is Influenced by Gender and Body Mass Index
Source: PLoS One. 2016 May 26;11(5):e0154090. doi: 10.1371/journal.pone.0154090 (PMC4881937; doi:10.1371/journal.pone.0154090)
Supplement: S5 Table — (PDF) [file pone.0154090.s009.pdf]

Table S5a. Associated taxonomies with Body mass index (BMI) at FDR < 0.05 level [a]

	Summary of OTUs reads			Binary model				Quantitative model				Meta analysis		Final association	
Taxonomie	No. Absent	No. Present	mean counts in presents	Estimate	s.e.	*t* value	*P* value	Estimate	s.e.	*t* value	*P* value	Meta z value	*Meta P* value	*z*	*P value*
k__Bacteria p__Proteobacteria c__Gammaproteobacteria o__Cardiobacteriales f__Cardiobacteriaceae g__Cardiobacterium	71	4	1.75	-4.24195	2.37290	-1.78766	0.07804	-5.52718	0.11621	-47.56072	**0.01338**	-2.99501	**0.00274**	-2.99501	**0.00274**
k__Bacteria p__Bacteroidetes c__Bacteroidia o__Bacteroidales f__Rikenellaceae g__Alistipes	16	59	53.17	-3.67338	1.25130	-2.93565	**0.00447**	-0.10021	0.26910	-0.37238	0.71102	-2.27248	**0.02306**	-2.84328	**0.00447**
k__Bacteria p__Bacteroidetes c__Bacteroidia o__Bacteroidales f__[Paraprevotellaceae] g__Paraprevotella	21	54	130.56	3.25642	1.12945	2.88319	**0.00519**	0.03801	0.34127	0.11138	0.91175	2.05485	**0.03989**	2.79517	**0.00519**
k__Bacteria p__Firmicutes c__Clostridia o__Clostridiales f__Clostridiaceae g__Clostridium	5	70	129.89	2.61207	2.15636	1.21133	0.22973	0.72489	0.27335	2.65187	**0.00998**	2.67109	**0.00756**	2.67109	**0.00756**
k__Bacteria p__Bacteroidetes c__Bacteroidia o__Bacteroidales f__Prevotellaceae g__	71	4	2.25	-0.27906	2.41506	-0.11555	0.90833	-2.24329	0.02838	-79.03470	**0.00805**	-1.95510	0.05057	-2.64978	**0.00805**
k__Bacteria p__Bacteroidetes c__Bacteroidia o__Bacteroidales f__Prevotellaceae g__Prevotella	1	74	1607.95	0.00000	0.00000	0.00000	1.00000	0.33719	0.12479	2.70211	**0.00861**	1.85762	0.06322	2.62708	**0.00861**
k__Archaea p__Euryarchaeota c__Methanobacteria o__Methanobacteriales f__Methanobacteriaceae g__Methanobrevibacter	49	26	31.38	-0.44387	1.13651	-0.39056	0.69728	-1.12528	0.39786	-2.82833	**0.00953**	-2.10823	**0.03501**	-2.59250	**0.00953**
k__Bacteria p__Proteobacteria c__Gammaproteobacteria o__Enterobacteriales f__Enterobacteriaceae g__Citrobacter	66	9	9.33	4.18009	1.57318	2.65710	**0.00970**	0.09168	1.37712	0.06657	0.94909	1.87393	0.06094	2.58628	**0.00970**
k__Bacteria p__Firmicutes c__Clostridia o__Clostridiales f__Clostridiaceae g__	6	69	38.58	-0.13521	1.98847	-0.06800	0.94598	0.70845	0.26806	2.64287	**0.01026**	1.76725	0.07719	2.56703	**0.01026**
k__Bacteria p__Firmicutes c__Clostridia o__Clostridiales f__Veillonellaceae g__Acidaminococcus	38	37	331.81	2.70483	1.06660	2.53594	**0.01338**	0.23528	0.22186	1.06049	0.29640	2.48728	**0.01287**	2.48728	**0.01287**
k__Bacteria p__Bacteroidetes c__Bacteroidia o__Bacteroidales f__[Odoribacteraceae] g__Odoribacter	3	72	46.57	0.82753	2.78051	0.29762	0.76685	-0.75739	0.29845	-2.53775	**0.01342**	-1.53863	0.12390	-2.47244	**0.01342**
k__Bacteria p__Proteobacteria c__Gammaproteobacteria o__Enterobacteriales f__Enterobacteriaceae g__Enterobacter	66	9	2.67	2.75462	1.63425	1.68555	0.09621	2.65084	1.19708	2.21443	0.06872	2.46338	**0.01376**	2.46338	**0.01376**
k__Bacteria p__Firmicutes c__Erysipelotrichi o__Erysipelotrichales f__Erysipelotrichaceae g__[Eubacterium]	27	48	66.77	-0.07356	1.11868	-0.06575	0.94776	0.47227	0.18553	2.54549	**0.01441**	1.68389	0.09220	2.44691	**0.01441**
k__Bacteria p__Cyanobacteria c__Chloroplast o__Streptophyta f__ g__	51	24	10.63	2.78753	1.11435	2.50149	**0.01464**	0.48019	0.63407	0.75731	0.45728	2.25176	**0.02434**	2.44113	**0.01464**
k__Bacteria p__Firmicutes c__Clostridia o__Clostridiales f__Veillonellaceae g__Megamonas	61	14	178.79	0.30809	1.37636	0.22384	0.82352	0.85183	0.29729	2.86532	**0.01537**	1.87141	0.06129	2.42355	**0.01537**
k__Bacteria p__Cyanobacteria c__4C0d-2 o__YS2 f__ g__	49	26	26.58	-1.16649	1.11858	-1.04283	0.30051	-0.97098	0.40491	-2.39800	**0.02499**	-2.31708	**0.02050**	-2.31708	**0.02050**
k__Bacteria p__Firmicutes c__Bacilli o__Turicibacterales f__Turicibacteraceae g__Turicibacter	59	16	13.88	1.10087	1.30605	0.84290	0.40208	1.12218	0.43689	2.56858	**0.02336**	2.19590	**0.02810**	2.26755	**0.02336**
k__Bacteria p__Firmicutes c__Clostridia o__Clostridiales f__Christensenellaceae g__Christensenella	64	11	4.45	-3.23294	1.46582	-2.20556	**0.03061**	-1.04834	1.23857	-0.84640	0.42192	-2.09673	**0.03602**	-2.16213	**0.03061**
k__Bacteria p__Firmicutes c__Clostridia o__Clostridiales f__Veillonellaceae g__Veillonella	14	61	66.15	-0.20052	1.39277	-0.14398	0.88592	0.53177	0.24093	2.20713	**0.03128**	1.42134	0.15522	2.15355	**0.03128**
k__Bacteria p__Firmicutes c__Bacilli o__Lactobacillales f__Lactobacillaceae g__	70	5	29.00	-1.21748	2.14394	-0.56787	0.57189	-0.77258	0.14000	-5.51839	**0.03130**	-1.92224	0.05458	-2.15319	**0.03130**
k__Bacteria p__Firmicutes c__Bacilli o__Lactobacillales f__Lactobacillaceae g__Lactobacillus	29	46	161.57	2.24965	1.07070	2.10110	**0.03913**	0.02393	0.27279	0.08772	0.93051	1.52027	0.12844	2.06278	**0.03913**
k__Bacteria p__Proteobacteria c__Gammaproteobacteria o__Enterobacteriales f__Enterobacteriaceae g__	6	69	305.32	0.24939	2.08736	0.11947	0.90523	0.36601	0.17720	2.06558	**0.04280**	1.51655	0.12938	2.02567	**0.04280**
k__Bacteria p__Proteobacteria c__Gammaproteobacteria o__Enterobacteriales f__Enterobacteriaceae g__Pantoea	32	43	269.12	1.37740	1.07161	1.28535	0.20279	0.37846	0.24448	1.54802	0.12949	1.97265	**0.04854**	1.97265	**0.04854**

[a] Statistics associated to the two part model for association analysis between the trait and microbes adjusting for age and gender. The binomial analysis that tests for association of detecting a microbe with the trait, where s.e. represents the standard error of the estimate, *t* -value of a *t* test for the estimate and its associated probability (*P* value). The quantitative analysis tests for association between the trait and the abundance of the microbe for the subjects where that microbe is present, where s.e. represents the standard error of the estimate, *t* -value of a *t* test for the estimate and its associated probability (*P* value). The Meta analysis combine the effect of both binary and quantitative analysis, a meta *P* value was derived using an unweighted Z method. The final association *P* value per microbe-trait pair was assigned from the minimum of *P* values from the binary analysis, quantitative analysis, and meta-analysis.

Table S5b. Associated taxonomies with Triglycerides at FDR < 0.05 level [a]

	Summary of OTUs reads			Binary model				Quantitative model				Meta analysis		Final association	
Taxonomie	No. Absent	No. Present	mean counts in presents	Estimate	s.e.	t value	P value	Estimate	s.e.	t value	P value	Meta z value	Meta P value	*z*	*P value*
k__Bacteria p__Firmicutes c__Clostridia o__Clostridiales f__Veillonellaceae g__Dialister	17	58	268.71	-75.53458	22.52918	-3.35274	**0.00128**	-0.42802	2.06444	-0.20733	0.83652	-2.42339	**0.01538**	-3.22084	**0.00128**
k__Bacteria p__Firmicutes c__Bacilli o__Lactobacillales f__ g__	67	8	5.63	101.77620	30.59668	3.32638	**0.00139**	-82.60462	58.64759	-1.40849	0.21803	1.38978	0.16459	3.19725	**0.00139**
k__Bacteria p__Fusobacteria c__Fusobacteriia o__Fusobacteriales f__Leptotrichiaceae g__Leptotrichia	69	6	2.67	33.97669	37.24747	0.91219	0.36471	-58.45030	9.29023	-6.29159	**0.00811**	-1.23109	0.21829	-2.64744	**0.00811**
k__Bacteria p__Firmicutes c__Clostridia o__Clostridiales f__Lachnospiraceae g__Oribacterium	59	16	2.88	62.41962	23.64636	2.63971	**0.01017**	16.93305	52.48570	0.32262	0.75211	2.04069	**0.04128**	2.57012	**0.01017**
k__Bacteria p__Firmicutes c__Clostridia o__Clostridiales f__Lachnospiraceae g__Lachnospira	0	75	288.77	0.00000	0.00000	0.00000	1.00000	-9.43567	3.79208	-2.48826	**0.01515**	-1.71737	0.08591	-2.42873	**0.01515**
k__Bacteria p__Firmicutes c__Clostridia o__Clostridiales f__[Mogibacteriaceae] g__Mogibacterium	67	8	2.13	29.00754	32.78823	0.88469	0.37927	-66.69096	20.12746	-3.31343	**0.02116**	-1.00824	0.31334	-2.30511	**0.02116**
k__Bacteria p__TM7 c__TM7-3 o__CW040 f__ g__	25	50	155.90	-40.58598	21.04268	-1.92875	0.05770	-3.95866	3.02501	-1.30864	0.19702	-2.25427	**0.02418**	-2.25427	**0.02418**
k__Bacteria p__Firmicutes c__Clostridia o__Clostridiales f__Veillonellaceae g__Phascolarctobacterium	12	63	301.17	13.04554	27.79236	0.46939	0.64021	8.48866	3.75927	2.25806	**0.02759**	1.88829	0.05899	2.20304	**0.02759**
k__Bacteria p__Firmicutes c__Bacilli o__Gemellales f__Gemellaceae g__	36	39	7.87	40.40409	19.77700	2.04298	**0.04472**	12.83030	11.63355	1.10287	0.27740	2.18743	**0.02871**	2.18743	**0.02871**
k__Bacteria p__Actinobacteria c__Coriobacteriia o__Coriobacteriales f__Coriobacteriaceae g__Atopobium	52	23	5.39	47.54758	21.39399	2.22247	**0.02939**	-18.08059	20.05529	-0.90154	0.37803	0.91687	0.35921	2.17818	**0.02939**
k__Bacteria p__Firmicutes c__Erysipelotrichi o__Erysipelotrichales f__Erysipelotrichaceae g__Coprobacillus	60	15	4.93	54.46843	24.72433	2.20303	**0.03079**	-18.83968	31.35802	-0.60079	0.55915	1.11414	0.26522	2.15973	**0.03079**
k__Bacteria p__Firmicutes c__Clostridia o__Clostridiales f__[Tissierellaceae] g__WAL_1855D	65	10	4.00	63.21708	28.94208	2.18426	**0.03220**	-49.29793	61.30630	-0.80413	0.44777	0.97777	0.32819	2.14191	**0.03220**
k__Bacteria p__Firmicutes c__Bacilli o__Lactobacillales f__Lactobacillaceae g__Lactobacillus	29	46	161.57	-39.88948	20.35123	-1.96005	0.05386	-3.66895	3.38111	-1.08513	0.28391	-2.12099	**0.03392**	-2.12099	**0.03392**
k__Bacteria p__Firmicutes c__Bacilli o__Lactobacillales f__Leuconostocaceae g__Weissella	61	14	7.00	15.93906	26.21858	0.60793	0.54515	12.11592	5.09175	2.37952	**0.03653**	1.90636	0.05660	2.09096	**0.03653**
k__Bacteria p__Firmicutes c__Clostridia o__Clostridiales f__Ruminococcaceae g__Ruminococcus	0	75	308.87	0.00000	0.00000	0.00000	1.00000	-12.14391	5.72785	-2.12015	**0.03744**	-1.47144	0.14117	-2.08093	**0.03744**
k__Bacteria p__Proteobacteria c__Epsilonproteobacteria o__Campylobacterales f__Campylobacteraceae g__Campylobacter	62	13	3.92	53.26154	26.04491	2.04499	**0.04451**	-103.67951	45.88597	-2.25950	**0.04741**	0.01881	0.98499	2.00924	**0.04451**
k__Bacteria p__Firmicutes c__Erysipelotrichi o__Erysipelotrichales f__Erysipelotrichaceae g__Catenibacterium	48	27	61.48	-41.98303	20.62267	-2.03577	**0.04545**	-0.50362	4.92345	-0.10229	0.91938	-1.48609	0.13725	-2.00043	**0.04545**
k__Bacteria p__Proteobacteria c__Gammaproteobacteria o__Enterobacteriales f__Enterobacteriaceae g__Erwinia	49	26	13.69	-6.62846	21.29569	-0.31126	0.75650	11.25878	5.36355	2.09913	**0.04698**	1.18539	0.23586	1.98647	**0.04698**

[a] Statistics associated to the two part model for association analysis between the trait and microbes adjusting for age and gender. The binomial analysis that tests for association of detecting a microbe with the trait, where s.e. represents the standard error of the estimate, *t* -value of a *t* test for the estimate and its associated probability (*P* value). The quantitative analysis tests for association between the trait and the abundance of the microbe for the subjects where that microbe is present, where s.e. represents the standard error of the estimate, *t* -value of a *t* test for the estimate and its associated probability (*P* value). The Meta analysis combine the effect of both binary and quantitative analysis, a meta *P* value was derived using an unweighted Z method. The final association *P* value per microbe-trait pair was assigned from the minimum of *P* values from the binary analysis, quantitative analysis, and meta-analysis.

Table S5c. Associated taxonomies with High-density lipoprotein (HDL) at FDR < 0.05 level [a]

	Summary of OTUs reads			Binary model				Quantitative model				Meta analysis		Final association	
Taxonomie	No. Absent	No. Present	mean counts in presents	Estimate	s.e.	t value	P value	Estimate	s.e.	t value	P value	Meta z value	Meta P value	*z*	*P value*
k__Bacteria p__Tenericutes c__Mollicutes o__Anaeroplasmatales f__Anaeroplasmataceae g__	71	4	687.50	7.70278	6.47966	1.18876	0.23844	-10.39396	0.01165	-892.05595	**0.00071**	-1.55944	0.11889	-3.38428	**0.00071**
k__Bacteria p__Firmicutes c__Clostridia o__Clostridiales f__Lachnospiraceae g__Oribacterium	59	16	2.88	1.71378	3.53338	0.48503	0.62913	-19.03891	5.11684	-3.72084	**0.00257**	-1.79074	0.07334	-3.01544	**0.00257**
k__Bacteria p__Firmicutes c__Bacilli o__Bacillales f__Staphylococcaceae g__Staphylococcus	71	4	2.75	18.30074	6.17725	2.96260	**0.00413**	-8.84490	15.41236	-0.57388	0.66832	1.72499	0.08453	2.86795	**0.00413**
k__Bacteria p__Proteobacteria c__Betaproteobacteria o__Burkholderiales f__Oxalobacteraceae g__Oxalobacter	42	33	9.12	6.31233	2.82518	2.23431	**0.02857**	4.51120	2.97952	1.51407	0.14047	2.59043	**0.00959**	2.59043	**0.00959**
k__Bacteria p__Firmicutes c__Erysipelotrichi o__Erysipelotrichales f__Erysipelotrichaceae g__Catenibacterium	48	27	61.48	7.56556	2.89679	2.61170	**0.01096**	1.05257	1.54711	0.68035	0.50279	2.27275	**0.02304**	2.54405	**0.01096**
k__Bacteria p__Firmicutes c__Erysipelotrichi o__Erysipelotrichales f__Erysipelotrichaceae g__	17	58	36.62	-8.19344	3.32906	-2.46119	**0.01625**	-0.03991	0.52314	-0.07630	0.93946	-1.75313	0.07958	-2.40335	**0.01625**
k__Bacteria p__Bacteroidetes c__Bacteroidia o__Bacteroidales f__Rikenellaceae g__Alistipes	16	59	53.17	-0.59856	3.58254	-0.16708	0.86778	-1.87315	0.80143	-2.33727	**0.02303**	-1.72498	0.08453	-2.27301	**0.02303**
k__Bacteria p__Firmicutes c__Bacilli o__Gemellales f__Gemellaceae g__	36	39	7.87	-5.03986	2.84586	-1.77094	0.08080	-1.58707	1.07656	-1.47421	0.14912	-2.25475	**0.02415**	-2.25475	**0.02415**
k__Bacteria p__Proteobacteria c__Gammaproteobacteria o__Cardiobacteriales f__Cardiobacteriaceae g__Cardiobacterium	71	4	1.75	9.78147	6.46054	1.51403	0.13440	21.38645	3.19541	6.69286	0.09442	2.24119	**0.02501**	2.24119	**0.02501**
k__Bacteria p__Bacteroidetes c__Bacteroidia o__Bacteroidales f__Bacteroidaceae g__Bacteroides	0	75	4087.83	0.00000	0.00000	0.00000	1.00000	-2.47105	1.10913	-2.22791	**0.02901**	-1.54385	0.12262	-2.18334	**0.02901**
k__Bacteria p__Actinobacteria c__Actinobacteria o__Bifidobacteriales f__Bifidobacteriaceae g__	68	7	5.00	10.66025	4.83020	2.20700	**0.03050**	-12.34710	4.71863	-2.61667	0.05901	0.19470	0.84563	2.16350	**0.03050**
k__Bacteria p__Proteobacteria c__Gammaproteobacteria o__Pseudomonadales f__Moraxellaceae g__Acinetobacter	64	11	2.45	8.08179	3.98966	2.02568	**0.04650**	10.40523	9.35575	1.11217	0.29836	2.14305	**0.03211**	2.14305	**0.03211**
k__Bacteria p__Actinobacteria c__Actinobacteria o__Bifidobacteriales f__Bifidobacteriaceae g__Alloscardovia	67	8	15.25	-4.62636	4.67216	-0.99020	0.32539	-2.71062	1.13746	-2.38304	0.06293	-2.01040	**0.04439**	-2.01040	**0.04439**
k__Bacteria p__Proteobacteria c__Gammaproteobacteria o__Enterobacteriales f__Enterobacteriaceae g__Escherichia	54	21	8.67	-0.97245	3.29060	-0.29552	0.76844	2.38835	1.12297	2.12681	**0.04752**	1.19304	0.23285	1.98162	**0.04752**
k__Bacteria p__Firmicutes c__Bacilli o__Bacillales f__Planococcaceae g__	62	13	2.46	-1.60995	3.82460	-0.42095	0.67505	8.09224	3.58647	2.25632	**0.04767**	1.10387	0.26965	1.98034	**0.04767**
k__Bacteria p__Actinobacteria c__Coriobacteriia o__Coriobacteriales f__Coriobacteriaceae g__Atopobium	52	23	5.39	-6.18905	3.07532	-2.01249	**0.04791**	-0.12069	1.53345	-0.07870	0.93805	-1.45373	0.14602	-1.97817	**0.04791**
k__Bacteria p__Firmicutes c__Clostridia o__Clostridiales f__Dehalobacteriaceae g__Dehalobacterium	48	27	6.04	3.98383	2.98866	1.33298	0.18674	3.92576	2.59107	1.51511	0.14280	1.96979	**0.04886**	1.96979	**0.04886**

[a] Statistics associated to the two part model for association analysis between the trait and microbes adjusting for age and gender. The binomial analysis that tests for association of detecting a microbe with the trait, where s.e. represents the standard error of the estimate, *t* -value of a *t* test for the estimate and its associated probability (*P* value). The quantitative analysis tests for association between the trait and the abundance of the microbe for the subjects where that microbe is present, where s.e. represents the standard error of the estimate, *t* -value of a *t* test for the estimate and its associated probability (*P* value). The Meta analysis combine the effect of both binary and quantitative analysis, a meta *P* value was derived using an unweighted Z method. The final association *P* value per microbe-trait pair was assigned from the minimum of *P* values from the binary analysis, quantitative analysis, and meta-analysis.

Table S5d. Associated taxonomies with Low-density lipoprotein (HDL) at FDR < 0.05 level [a]

	Summary of OTUs reads			Binary model				Quantitative model				Meta analysis		Final association	
Taxonomie	No. Absent	No. Present	mean counts in presents	Estimate	s.e.	t value	P value	Estimate	s.e.	t value	P value	Meta z value	Meta P value	*z*	*P value*
k__Bacteria p__Actinobacteria c__Coriobacteriia o__Coriobacteriales f__Coriobacteriaceae g__	23	52	14.87	0.79347	5.15124	0.15403	0.87801	5.00216	1.69927	2.94371	**0.00495**	2.09589	**0.03609**	2.81054	**0.00495**
k__Bacteria p__Proteobacteria c__Gammaproteobacteria o__Enterobacteriales f__Enterobacteriaceae g__	6	69	305.32	-22.70412	8.82754	-2.57196	**0.01218**	-1.08496	0.75551	-1.43606	0.15571	-2.77657	**0.00549**	-2.77657	**0.00549**
k__Bacteria p__Bacteroidetes c__Bacteroidia o__Bacteroidales f__Prevotellaceae g__	71	4	2.25	27.77277	10.15788	2.73411	**0.00787**	-12.63501	20.75306	-0.60883	0.65184	1.56023	0.11870	2.65771	**0.00787**
k__Bacteria p__Proteobacteria c__Gammaproteobacteria o__Enterobacteriales f__Enterobacteriaceae g__Escherichia	54	21	8.67	-12.87293	5.15750	-2.49596	**0.01485**	0.11916	2.16502	0.05504	0.95671	-1.68410	0.09216	-2.43595	**0.01485**
k__Bacteria p__Firmicutes c__Bacilli o__Lactobacillales f__ g__	67	8	5.63	17.84628	7.37449	2.42000	**0.01805**	5.25760	7.90853	0.66480	0.53561	2.11011	**0.03485**	2.36468	**0.01805**
k__Bacteria p__Firmicutes c__Clostridia o__Clostridiales f__Ruminococcaceae g__Clostridium	71	4	1.25	-20.64467	10.40515	-1.98408	0.05106	-29.27143	12.88989	-2.27088	0.26407	-2.16926	**0.03006**	-2.16926	**0.03006**
k__Bacteria p__Firmicutes c__Erysipelotrichi o__Erysipelotrichales f__Erysipelotrichaceae g__Clostridium	68	7	31.43	-0.87960	8.20209	-0.10724	0.91490	-5.75344	1.76883	-3.25267	**0.03130**	-1.59816	0.11001	-2.15328	**0.03130**
k__Bacteria p__Firmicutes c__Clostridia o__Clostridiales f__Clostridiaceae g__SMB53	53	22	3.14	-0.05586	5.21953	-0.01070	0.99149	10.33790	4.45099	2.32261	**0.03146**	1.51361	0.13013	2.15123	**0.03146**
k__Bacteria p__Firmicutes c__Bacilli o__Lactobacillales f__Leuconostocaceae g__Weissella	61	14	7.00	8.17949	6.05740	1.35033	0.18114	8.24201	4.40721	1.87012	0.08830	2.15081	**0.03149**	2.15081	**0.03149**
k__Bacteria p__Proteobacteria c__Gammaproteobacteria o__Pseudomonadales f__Pseudomonadaceae g__Pseudomonas	61	14	27.07	-5.70772	6.05318	-0.94293	0.34887	-4.01931	1.71127	-2.34873	**0.03857**	-2.12520	**0.03357**	-2.12520	**0.03357**
k__Bacteria p__Firmicutes c__Clostridia o__Clostridiales f__Veillonellaceae g__Dialister	17	58	268.71	-0.24483	5.65247	-0.04331	0.96557	-1.57944	0.74002	-2.13433	**0.03729**	-1.50313	0.13280	-2.08259	**0.03729**
k__Bacteria p__Bacteroidetes c__Bacteroidia o__Bacteroidales f__ g__	54	21	223.33	1.72619	5.27233	0.32741	0.74431	2.43587	1.09260	2.22942	**0.03876**	1.69200	0.09065	2.06670	**0.03876**
k__Bacteria p__Firmicutes c__Clostridia o__Clostridiales f__[Tissierellaceae] g__Peptoniphilus	68	7	7.14	-0.32745	8.21295	-0.03987	0.96831	-9.50340	3.25620	-2.91856	**0.04331**	-1.45698	0.14512	-2.02075	**0.04331**
k__Bacteria p__Proteobacteria c__Gammaproteobacteria o__Pasteurellales f__Pasteurellaceae g__Actinobacillus	65	10	3.10	2.55452	7.11844	0.35886	0.72075	-13.90841	5.87645	-2.36680	**0.04984**	-1.13411	0.25675	-1.96134	**0.04984**

[a] Statistics associated to the two part model for association analysis between the trait and microbes adjusting for age and gender. The binomial analysis that tests for association of detecting a microbe with the trait, where s.e. represents the standard error of the estimate, *t* -value of a *t* test for the estimate and its associated probability (*P* value). The quantitative analysis tests for association between the trait and the abundance of the microbe for the subjects where that microbe is present, where s.e. represents the standard error of the estimate, *t* -value of a *t* test for the estimate and its associated probability (*P* value). The Meta analysis combine the effect of both binary and quantitative analysis, a meta *P* value was derived using an unweighted Z method. The final association *P* value per microbe-trait pair was assigned from the minimum of *P* values from the binary analysis, quantitative analysis, and meta-analysis.

Table S5e. Associated taxonomies with Total cholesterol (TC) at FDR < 0.05 level [a]

	Summary of OTUs reads			Binary model				Quantitative model				Meta analysis		Final association	
Taxonomie	No. Absent	No. Present	mean counts in presents	Estimate	s.e.	t value	P value	Estimate	s.e.	t value	P value	Meta z value	Meta P value	*z*	*P value*
k__Bacteria p__Firmicutes c__Clostridia o__Clostridiales f__Ruminococcaceae g__Anaerotruncus	35	40	5.48	-9.27950	5.74866	-1.61420	0.11086	-6.72385	2.77878	-2.41972	**0.02056**	-2.76497	**0.00569**	-2.76497	**0.00569**
k__Bacteria p__Proteobacteria c__Gammaproteobacteria o__Pasteurellales f__Pasteurellaceae g__	63	12	4.42	-18.95427	7.59835	-2.49453	**0.01491**	1.12192	6.19121	0.18121	0.86022	-1.59700	0.11026	-2.43460	**0.01491**
k__Bacteria p__Firmicutes c__Bacilli o__Gemellales f__Gemellaceae g__	36	39	7.87	4.76046	5.75766	0.82681	0.41108	-5.97766	2.53353	-2.35942	**0.02385**	-1.01652	0.30938	-2.25958	**0.02385**
k__Bacteria p__Firmicutes c__Clostridia o__Clostridiales f__Christensenellaceae g__Christensenella	64	11	4.45	-17.72210	7.88383	-2.24790	**0.02765**	9.16884	8.21083	1.11668	0.29655	-0.81912	0.41272	-2.20228	**0.02765**
k__Bacteria p__Bacteroidetes c__Bacteroidia o__Bacteroidales f__ g__	54	21	223.33	-3.93402	6.41354	-0.61339	0.54155	2.98066	1.24762	2.38909	**0.02805**	1.12158	0.26204	2.19662	**0.02805**
k__Bacteria p__Firmicutes c__Clostridia o__Clostridiales f__Clostridiaceae g__SMB53	53	22	3.14	-3.42593	6.34833	-0.53966	0.59110	10.51976	4.45741	2.36006	**0.02912**	1.16292	0.24486	2.18186	**0.02912**
k__Bacteria p__Proteobacteria c__Gammaproteobacteria o__Enterobacteriales f__Enterobacteriaceae g__	6	69	305.32	-23.61437	10.89174	-2.16810	**0.03346**	0.39100	0.95821	0.40805	0.68456	-1.21644	0.22382	-2.12655	**0.03346**
k__Bacteria p__Proteobacteria c__Deltaproteobacteria o__Desulfovibrionales f__Desulfovibrionaceae g__Desulfovibrio	28	47	205.96	6.29885	5.95658	1.05746	0.29384	2.52163	1.25830	2.00401	0.05125	2.12070	**0.03395**	2.12070	**0.03395**
k__Bacteria p__Bacteroidetes c__Bacteroidia o__Bacteroidales f__[Paraprevotellaceae] g__Paraprevotella	21	54	130.56	10.14829	6.31136	1.60794	0.11223	2.44311	1.70817	1.43025	0.15874	2.11961	**0.03404**	2.11961	**0.03404**
k__Bacteria p__Actinobacteria c__Coriobacteriia o__Coriobacteriales f__Coriobacteriaceae g__Eggerthella	60	15	5.53	-15.04464	7.00762	-2.14690	**0.03517**	-0.64027	4.78001	-0.13395	0.89567	-1.58217	0.11361	-2.10639	**0.03517**
k__Bacteria p__Firmicutes c__Bacilli o__Lactobacillales f__Leuconostocaceae g__Weissella	61	14	7.00	-0.06516	7.47518	-0.00872	0.99307	10.41354	4.43221	2.34952	**0.03852**	1.45706	0.14510	2.06928	**0.03852**
k__Bacteria p__Actinobacteria c__Actinobacteria o__Bifidobacteriales f__Bifidobacteriaceae g__Bifidobacterium	7	68	91.18	-2.49938	9.90983	-0.25221	0.80160	-2.91829	1.39534	-2.09145	**0.04040**	-1.62700	0.10374	-2.04964	**0.04040**
k__Bacteria p__Firmicutes c__Clostridia o__Clostridiales f__Ruminococcaceae g__Ruminococcus	0	75	308.87	0.00000	0.00000	0.00000	1.00000	-3.38723	1.63083	-2.07700	**0.04137**	-1.44236	0.14920	-2.03980	**0.04137**
k__Bacteria p__Actinobacteria c__Coriobacteriia o__Coriobacteriales f__Coriobacteriaceae g__Adlercreutzia	60	15	3.47	-14.53509	7.00583	-2.07471	**0.04159**	5.99994	5.26070	1.14052	0.27632	-0.67104	0.50219	-2.03762	**0.04159**
k__Bacteria p__Firmicutes c__Clostridia o__Clostridiales f__Peptococcaceae g__	40	35	13.69	-3.96541	5.83952	-0.67906	0.49927	-5.88691	2.77386	-2.12228	**0.04165**	-1.91813	0.05509	-2.03701	**0.04165**
k__Bacteria p__Firmicutes c__Clostridia o__Clostridiales f__Ruminococcaceae g__Clostridium	71	4	1.25	-20.34598	12.80038	-1.58948	0.11633	-36.28571	11.20860	-3.23731	0.19073	-2.03560	**0.04179**	-2.03560	**0.04179**
k__Bacteria p__Firmicutes c__Clostridia o__Clostridiales f__Clostridiaceae g__Sarcina	68	7	30.14	6.51830	9.90905	0.65781	0.51276	-10.72036	3.80527	-2.81724	**0.04796**	-0.93561	0.34948	-1.97770	**0.04796**
k__Bacteria p__Actinobacteria c__Coriobacteriia o__Coriobacteriales f__Coriobacteriaceae g__	23	52	14.87	0.42868	6.27876	0.06827	0.94576	4.22936	2.10125	2.01278	**0.04965**	1.43614	0.15096	1.96297	**0.04965**

[a] Statistics associated to the two part model for association analysis between the trait and microbes adjusting for age and gender. The binomial analysis that tests for association of detecting a microbe with the trait, where s.e. represents the standard error of the estimate, *t* -value of a *t* test for the estimate and its associated probability (*P* value). The quantitative analysis tests for association between the trait and the abundance of the microbe for the subjects where that microbe is present, where s.e. represents the standard error of the estimate, *t* -value of a *t* test for the estimate and its associated probability (*P* value). The Meta analysis combine the effect of both binary and quantitative analysis, a meta *P* value was derived using an unweighted Z method. The final association *P* value per microbe-trait pair was assigned from the minimum of *P* values from the binary analysis, quantitative analysis, and meta-analysis.
